# Inhibition of Vascular Smooth Muscle and Cancer Cell Proliferation by New VEGFR Inhibitors and Their Immunomodulator Effect: Design, Synthesis, and Biological Evaluation

**DOI:** 10.1155/2021/8321400

**Published:** 2021-10-28

**Authors:** Feng Ran, Wendong Li, Yi Qin, Tong Yu, Zhao Liu, Min Zhou, Cheng Liu, Tong Qiao, Xiaoqiang Li, Reda G. Yousef, Ibrahim H. Eissa, Mohamed M. Khalifa

**Affiliations:** ^1^Department of Vascular Surgery, Nanjing Drum Tower Hospital Affiliated to Nanjing University Medical School, Nanjing 210008, China; ^2^Pharmaceutical Medicinal Chemistry & Drug Design Department, Faculty of Pharmacy (Boys), Al-Azhar University, Cairo 11884, Egypt

## Abstract

Abnormal vascular smooth muscle cell (VSMC) proliferation has an important role in the pathogenesis of both atherosclerosis restenosis and hypertension. Vascular endothelial growth factor (VEGF) has been shown to stimulate VSMC proliferation. In addition, angiogenesis is one of the hallmarks of cancerous growth. VEGF is the key modulator for the initial stages of angiogenesis that acts through the endothelial-specific receptor tyrosine kinases (VEGFRs). VEGFR-2 blockage is a good approach for suppression of angiogenesis. In order to discover novel VEGFR-2 TK inhibitors, we have designed and synthesized three new series of pyridine-containing compounds. The new compounds were all screened against a panel of three cell lines (HepG-2, HCT-116, and MCF-7). Promising results encouraged us to additionally evaluate the most active members for their *in vitro* VEGFR-2 inhibitory effect. Compound 7a, which is the most potent candidate, revealed a significant increase in caspase-3 level by 7.80-fold when compared to the control. In addition, Bax and Bcl-2 concentration levels showed an increase in the proapoptotic protein Bax (261.4 Pg/ml) and a decrease of the antiapoptotic protein Bcl-2 (1.25 Pg/ml) compared to the untreated cells. Furthermore, compound 7a arrested the cell cycle in the G2/M phase with induction of apoptosis. The immunomodulatory effect of compound 7a, the most active member, showed a reduction in TNF-*α* by 87%. Also, compound 7a caused a potent inhibitory effect on smooth muscle proliferation. Docking studies were also performed to get better insights into the possible binding mode of the target compounds with VEGFR-2 active sites.

## 1. Introduction

Smooth muscles are located in many organs of the body. It performs many biological roles such as digestion and nutrient collection in the stomach and intestines. In the urinary system, it participates in toxin clearance and electrolyte balance. Also, it plays a critical role in the regulation of blood pressure and tissue oxygenation in arteries and veins. These important roles enable the body to maintain its most basic functions [1].

Abnormal vascular smooth muscle cell (VSMC) proliferation is thought to play an important role in the pathogenesis of both atherosclerosis restenosis and hypertension [2, 3]. Many growth factors and cytokines have been shown to stimulate VSMC proliferation such as vascular endothelial growth factor (VEGF) [4, 5], platelet-derived growth factors (PDGF) [6], basic fibroblast growth factor (bFGF) [7], tumor necrosis factor-*α* (TNF-*α*) [8], and interleukin-1 [9]. VEGF is overexpressed by VSMC in the intima of atherosclerotic human coronary arteries [10].

One of the hallmarks of cancerous growth is angiogenesis, the process of development of new capillaries from preexisting blood vessels [11–13]. The newly formed blood vessels supply oxygen and nutrients to the tumor cells that augments their growth and metastasis [14]. Tumors that lack a sufficient vasculature become necrotic or apoptotic and cannot grow beyond a limited size [15]. Thus, identifying new antiangiogenic molecules is still one of the main strategies for cancer treatment.

Regarding the molecular basis of angiogenesis, vascular endothelial growth factor (VEGF) is the key modulator for the initial stages of angiogenesis that acts through the endothelial-specific receptor tyrosine kinases (VEGFRs) [16]. VEGF has been proven to play pivotal roles in almost all aspects of cellular physiology [17]. Downregulation of kinase activity has been involved in many pathological cases ranging from neuronal complaints to cellular transformation in leukemias [18]. Over a quarter of all pharmaceutical drugs is currently estimated to target protein kinases [19], an assessment that encourages an ambitious search for discovery of new chemical scaffolds that have the potential to become drugs. VEGF is secreted by tumors and motivates a mitogenic response via its binding to one of three tyrosine kinase receptors (VEGFR 1-3) on nearby endothelial cells [20].

Among the redundancy in function and ligand receptor interactions, VEGFR-2 is considered the main receptor involved in mediating the permeability and in initiating cascades of transduction signals in response to interaction with VEGF [20]. Therefore, hindering this signaling pathway should block angiogenesis with subsequent starvation and reduction of tumor growth [21, 22].

Analyzing the VEGFR-2 kinase domain revealed that it comprises of a bilobed structure with Mg-ATP placed in a deep cleft located between the N- and C-terminal lobes [23]. Most of the historically developed kinase inhibitors (type I) target the ATP binding site (the active conformation) in a way that is almost identical to that of ATP [24]. Type I kinase inhibitors bind to the ATP binding site via development of hydrogen bonds to the kinase “hinge” residues in addition to hydrophobic interactions in and around the region occupied by the adenine ring of ATP [25]. Recently, a second class of tyrosine kinase inhibitors (type II) was identified [26]. This type binds not only to ATP binding site but also to an adjacent hydrophobic pocket created by the activation loop [27]. This pocket contains the conserved DFG motif. Type II inhibitors use also the ATP binding site in a similar way to type I, but they further interact with the DFG residues through unique hydrogen bonding and hydrophobic interactions [27]. Type II kinase inhibitors were proven to possess higher efficacy and selectivity compared to type I inhibitors.

In the past two decades, several patents and researches reported the discovery and design of numerous new type II kinase inhibitors that have the ability to form extensive hydrophobic and hydrogen bonding interactions with both the adenine and allosteric binding site [13, 16, 26, 28–31]. Although all VEGFR-2 tyrosine kinase inhibitors target VEGF signaling, they are structurally varied and differ in pharmacological efficacy and clinical performance.

On the other side, many antiangiogenic drugs targeting the VEGF/VEGFR pathway appear to be capable of modulating immune responses. Such immunomodulatory activity has a positive effect in cancer treatment; however, it has a harmful impact in some cases [32]. In some cancer patients, there is an increase in the level of MDSCF which interferes with antigen presenting cells (APC) and effector T-cell functions. Hence, it is necessary to reduce these cells during the clinical treatment using immunotherapeutic modalities [33–35]. Sunitinib, which is one of the VEGFRIs, was reported to have a potential to modulate antitumor immunity by reversing MDSC accumulation [36, 37]. Additionally, the treatment using sunitinib can reduce expression of interleukin-10, tumor growth factor-beta, and Foxp3. In the same time, it can enhance expression of Th1 cytokine interferon-gamma (IFN-*γ*) [37]. Imatinib, as another example of VEGFRI, was proven to exert significant immunomodulatory changes in APC and T cells during clinical treatment of cancer patients [32].

The neovascularization (angiogenesis) process is the initial step for cancer development and metastasis [38]. One of the key elements in such a step is vascular smooth muscle cells (SMCs) which consist of the majority of the cells forming the wall of blood vessels. It is well known that SMCs have a critical contribution in neovascularization and transformation of the blood vessels [2, 39]. It was confirmed that VEGF isoforms are involved in the normal formation of SMCs surrounded by arteries [5]. Additionally, the *in vitro* angiogenesis assay described by Sini et al. confirmed that the epidermal growth factor can potentiate the SMCs to release VEGF, leading to a dramatic development in angiogenesis. Such process can be blocked by VEGFIs [40]. Thus, the class of VEGFIs may be a promising approach that can inhibit SMC proliferation and consequently tumor growth.

The previous findings encouraged our team to design a novel series of VEGFR-2 tyrosine kinase inhibitors that could tackle both tumor angiogenesis and SMC proliferation in addition to enhancement of the immune responses of the metastatic cells.

### 1.1. Rationale of Molecular Design

The main target of our work is the discovery of new VEGFR-2 inhibitors. These new compounds should comprise the essential pharmacophoric feature of the reported and FDA-approved VEGFR-2 inhibitors. The new compounds were assessed for their inhibitory effect against vascular smooth muscle and cancer cell proliferation. As well, their potential activity against VEGFR-2 was also investigated. In order to prove the immunomodulatory action of the synthesized compounds, different mediators such as caspase-3, BAX, BCl-2, and TNF-*α* were investigated *in vitro* on HepG-2 cells after application of the most active compounds.

**Figure 1:**
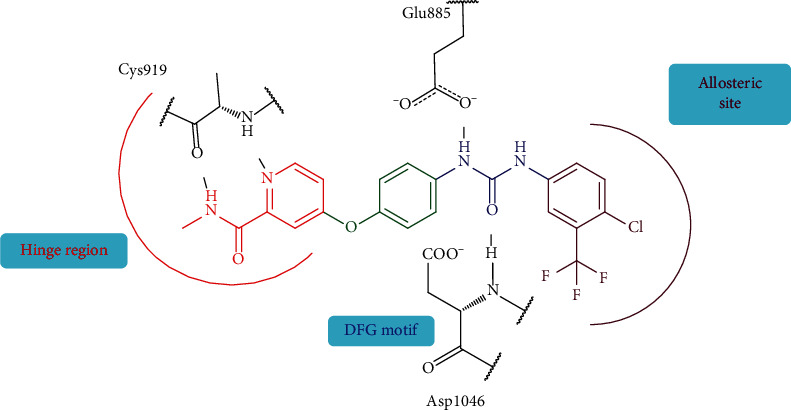
The essential pharmacophoric features of VEGFR-2 inhibitors. Red: hinge head; green: linker; blue: hydrogen-bonding moiety; maroon: hydrophobic tail.

Over the last few years, much efforts have been made by our research members aimed at developing drugs and optimize the effects of VEGFR-2 tyrosine kinase inhibitors via using the X-ray crystal structures of VEGFR-2 protein with various ligands [13, 16–18, 31, 41]. Examination of the binding features of known type II inhibitors led us to a conclusion regarding the main pharmacophoric requirements needed for the generation of new members that strongly hinder the action of VEGF on VEGFR-2 receptors. These requirements were demonstrated to be four main moieties. The first one is a “hinge-binding” head segment which is typically a flat heteroaromatic ring with at least one H-bond acceptor that allows the compound to bind to the ATP pocket via a hydrogen bond with the backbone NH of Cys919 residue. The second requirement is a 3-6 chemical bond length “linker” or “spacer.” The linker is either a rigid aromatic ring system or a highly flexible aliphatic chain. Its role is to traverse the kinase gatekeeper residues linking the hinge-binding segment with the hydrogen-bonding moiety. The “hydrogen-bonding moiety” is the third pharmacophoric feature needed for the design of VEGFR-2 inhibitors. It interacts via hydrogen bonding with Glu885 and/or Asp1046 residues in the DFG motif of the enzyme. The fourth part is the “tail” segment that consists of a hydrophobic moiety. It occupies the allosteric hydrophobic region binding through different hydrophobic interactions (Figure 1).

The previous investigation gave us the opportunities to design novel VEGFR-2 inhibitors with different spacers, hydrogen-bonding moieties, and/or various substituents around the terminal hydrophobic ring. Sorafenib (Figure 1) was used as a lead compound. Thus, three main scaffolds were designed and biologically evaluated for their VEGFR-2 inhibitory effects. Similarly as sorafenib, the hinge-binding head of all scaffolds was fixed to be a pyridine ring.

**Figure 2:**
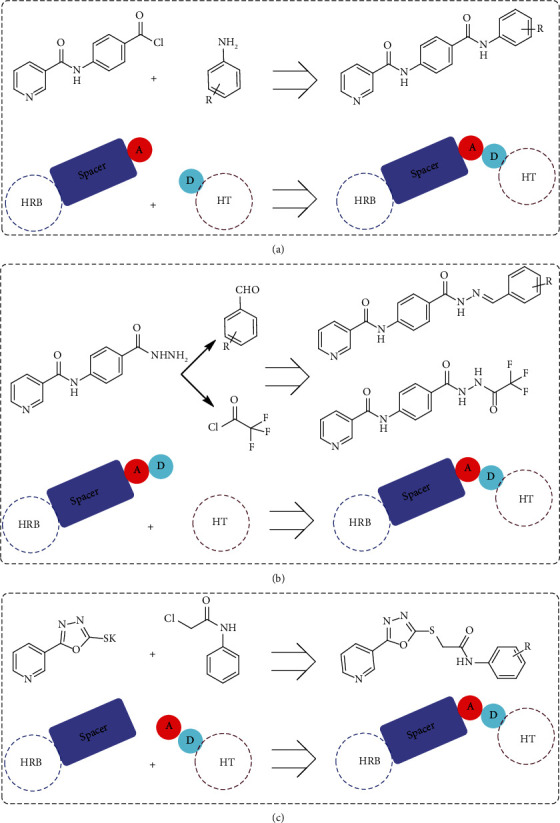
Design of the newly proposed VEGFR-2 inhibitors. A: hydrogen bond acceptor; D: hydrogen bond donor; HRB: hinge-region binding; HT: hydrophobic tail.

Scaffolds 1 and 2 were developed by structure optimization of sorafenib through increasing the length of the linker portion to be a phenyl carbamoyl group instead of the sorafenib phenoxy group. Another modification was made via replacement of the sorafenib urea HBA/HBD portion either by an amide moiety (scaffold 1) or a hydrazone moiety (scaffold 2). The hydrophobic tail was also modified to have an aliphatic structure 10 or aromatic moiety with different substitutions 5, 6, 7a-l, and 11a-h (Figure 2).

On the other side, many published articles reported that an oxadiazole moiety plays a vital role in the antiproliferative activity when it is used as a spacer in numerous synthetic molecules to join different anticancer scaffolds [42–44]. Consequently, linking the pyridine ring, via the oxadiazole moiety, to different hydrophobic tails bearing an amide group afforded the potent scaffold 3 VEGFR-2 inhibitors 18a-c (Figure 2).

The wide variety of modifications allowed us to examine the SAR of the designed compounds as effective antiproliferative agents with potential VEGFR-2 inhibitory effects. To confirm such design, silicomolecular docking studies of the synthesized compounds were performed against the prospective biological target (VEGFR-2).

## 2. Results and Discussion

### 2.1. Chemistry

**Scheme 1:**
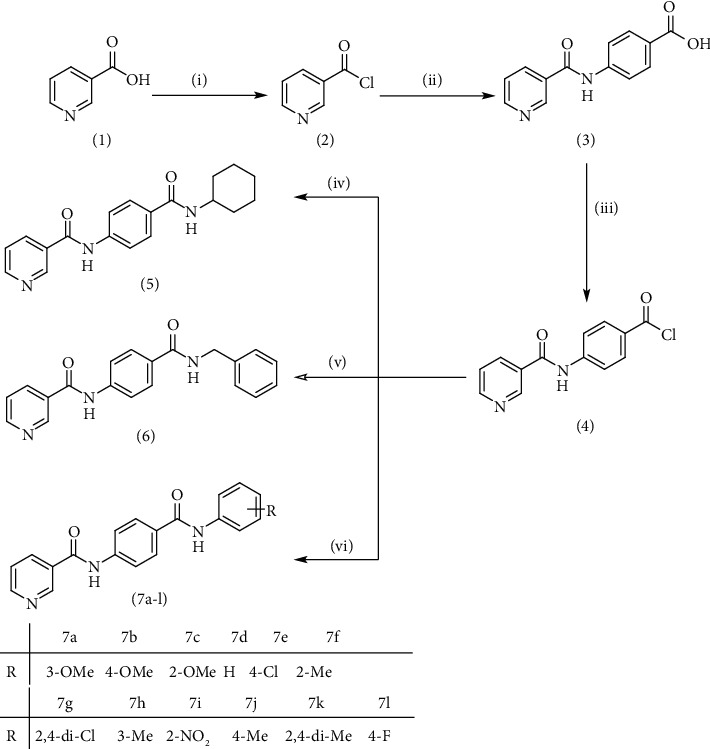
General procedure for the synthesis of target compounds 5, 6, and 7a-l. Reagents and conditions: (i) thionyl chloride (SOCl_2_), dicloroethane, reflux, 2 h; (ii) 4-aminibenzoic acid, triethylamine (TEA), acetonitrile, stirring, rt; (iii) thionyl chloride (SOCl_2_), dichloroethane, reflux, 2 h; (iv) cyclohexylamine, treithylamine (TEA), acetonitrile, reflux, 2 h; (v) benzylamine, treithylamine (TEA), acetonitrile, reflux, 2 h; (vi) app. amine, treithylamine (TEA), acetonitrile, reflux, 2 h.

**Scheme 2:**
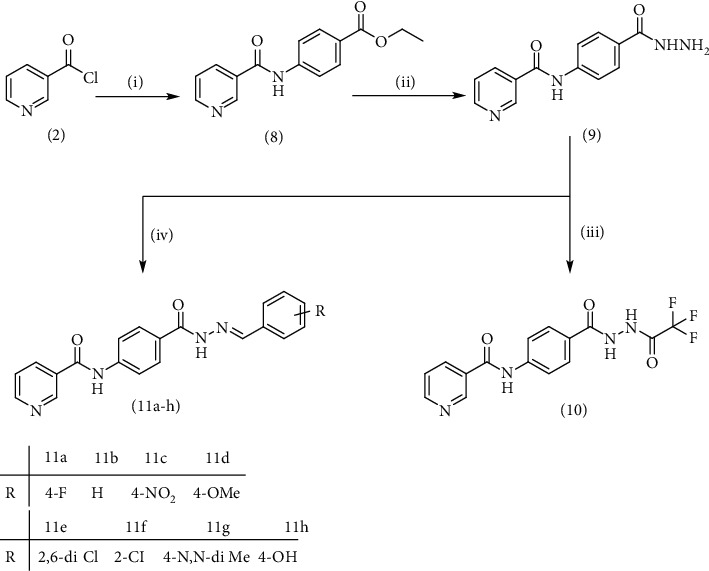
General procedure for synthesis of target compounds 10 and 11a-h. Reagents and conditions: (i) ethyl 4-aminobenzoate, treithylamine (TEA), acetonitrile, stirring, rt; (ii) hydrazine hydrate, EtOH, reflux, 12 h; (iii) triflouroacetic anhydride, DCM, stirring, rt; (iv) app. aldehyde, EtOH, g. acetic acid, reflux, 2 h.

Nicotinamide derivatives 5, 6, 7a-l, 10, and 11a-h were prepared following the reactions shown in Schemes 1 and 2. Compounds 5, 6, and 7a-l bearing various carbamoyl groups were synthesized starting from the commercially available nicotinic acid 1. Compound 1 was chlorinated using thionyl chloride in dichloroethane to afford nicotinoyl chloride 2 [45]. S_N_Ar displacement reaction of 2 with 4-aminobenzoic acid in the presence of TEA gave 4-(nicotinamido)benzoic acid 3. Subsequently, compound 3 was treated with thionyl chloride in dichloroethane to produce compound 4 in 85% yield. Treating compound 4 with cyclohexylamine, benzylamine, and different aniline derivatives in acetonitrile yielded the corresponding targeted compounds 5, 6, and 7a-l, respectively. The structures of compounds 5, 6, and 7a-l were confirmed by ^1^H NMR, which showed the presence of a new singlet signal attributed to a NH proton at a range of *δ* 9.13 to 10.76 ppm (Scheme 1).

Compound 2, moreover, underwent another S_N_Ar displacement route using ethyl 4-aminobenzoate with a catalytic amount of TEA resulting in synthesis of ethyl 4-(nicotinamido)benzoate 8 as white crystals [46]. The reflux of 8 with hydrazine hydrate in boiling ethanol for 12 h yielded *N*-(4-(hydrazinecarbonyl)phenyl)nicotinamide 9 [47]. The condensation of 9 with trifluoroacetic anhydride (TFAA) in DCM at room temperature afforded compound 10. Furthermore, a nucleophilic addition reaction was performed through the reaction of compound 9 with different benzaldehyde derivatives in the presence of a catalytic amount of glacial acetic acid to give the designed derivatives 11a-h. ^1^H NMR of 11a-h exhibited the disappearance of the characteristic NH_2_ signal of 9 and the appearance of a new benzylidene proton signal at *δ* values ranging from 8.34 to 8.89 ppm (Scheme 2).

**Scheme 3:**
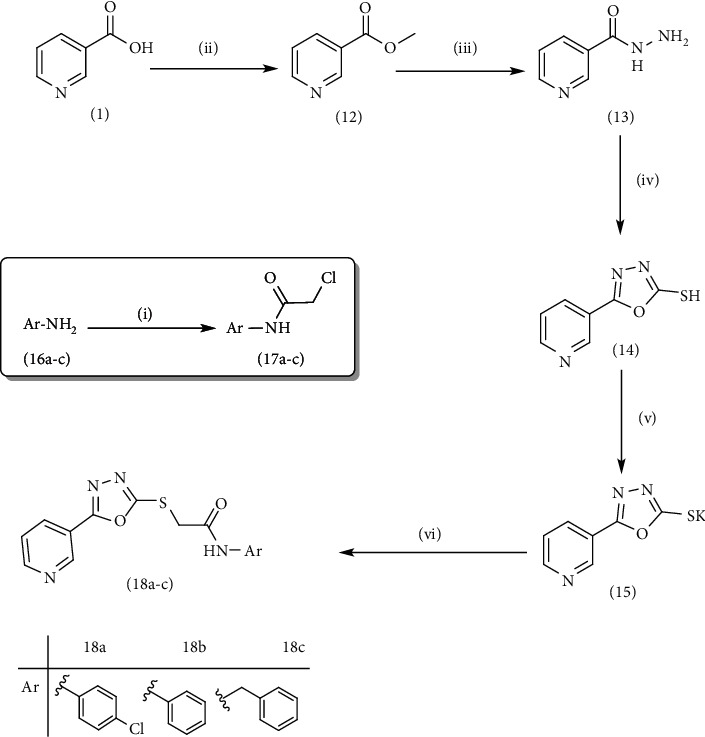
General procedure for synthesis of target compounds 18a-c. Reagents and conditions: (i) chloroacetylchloride, DMF, stirring, 12 h; (ii) MeOH, sulfuric acid, reflux, 4 h; (iii) hydrazine hydrate, EtOH, reflux, 8 h; (iv) (1) carbon disulfide, KOH, EtOh, reflux, 12 h, (2) HCl; (v) KOH, absoluteEtOH, reflux, 0.5 h; (vi) Comp 17a-c, DMF, Kl, heating, 4 h.

On the other side, the employed synthetic pathway used for the preparation of the pyridinyl derivatives 18a-c was outlined in Scheme 3. Scheme 3 began with the esterification of nicotinic acid 1 using methyl alcohol and sulfuric acid to afford methyl nicotinate 12 in a good yield. Compound 12 was then refluxed with hydrazine hydrate in ethanol to give nicotinohydrazide 13. A cyclization reaction was efficiently carried out via treatment of 13 with carbon disulfide in alcoholic potassium hydroxide followed by neutralization with HCl yielding 5-(pyridin-3-yl)-1,3,4-oxadiazole-2-thiol 14. A subsequent reflux of 14 with potassium hydroxide in absolute ethanol afforded the potassium salt 15. The later salt was then reacted with different acetanilide derivatives 17a-c, formed via the reaction of different amines 16a-c with chloroacetylchloride, to produce the corresponding final compounds 18a-c. IR of 18a-c were characterized by the appearance of carbonyl absorption bands at 1635–1647 cm^−1^ (Scheme 3).

### 2.2. Biological Evaluation

#### 2.2.1. In Vitro Antiproliferative Activity against MCF-7, HepG-2, and HCT-116

The *in vitro* antiproliferative activities of the newly synthesized compounds were evaluated against a panel of three human tumor cell lines, namely, breast cancer (MCF-7), hepatocellular carcinoma (HepG-2), and colorectal carcinoma (HCT-116) using the standard MTT method. The tested cell lines were selected depending on their VEGF overexpression. Sorafenib, the lead drug, was coassayed as a positive control. The cytotoxicity results are presented in Table 1.

Regarding the *N*-[4-(substituted carbamoyl)phenyl]nicotinamide 5, 6, and 7a-l series, it was noticed that the cytotoxicity IC_50_ of these members ranged from 1.25 to 42.10 *μ*M (MCF-7), 1.05 to 31.45 *μ*M (HepG-2), and 1.46 to 31.70 *μ*M (HCT-116) in comparison to the reference drug, sorafenib, with IC_50_ values of 4.32 (MCF-7), 3.76 (HepG-2), and 5.28 *μ*M (HCT-116). Results revealed that derivatives including a substituted phenyl ring were more potent than those including an unsubstituted one. Derivatives with *meta*- or *para*-methoxy groups 7a and 7b, particularly, exhibited the highest cytotoxic activity throughout the series (IC_50_ = 1.37, 1.05, and 1.46 *μ*M for 7a and 1.25, 2.12, and 2.54 *μ*M for 7b) that assessed the importance of the terminal hydrophobic tail in increasing the cytotoxic activity. The potency of cytotoxic activity was, however, decreased by the introduction of electron donating groups, e.g., *ortho-*, *meta*-, or *para*-methyl 7f, 7h, and 7j as well as the *di*-methyl substituted derivatives 7k (IC_50_ ranging from 14.08 to 31.45 *μ*M). Contrariwise, substitution of the phenyl ring with a strong electron withdrawing flouro group 7l dramatically potentiated the cytotoxic activity (IC_50_ = 3.50, 2.64, and 2.75 *μ*M). On the other hand, incorporation of a cyclohexyl 5 moiety increased the activity (IC_50_ = 4.66, 3.29, and 1.60 *μ*M), whereas the bulky benzyl group of compound 6 lowered the cytotoxic activity (IC_50_ = 18.32, 15.45, and 16.40 *μ*M).

Investigation of the cytotoxicity results of the hydrazone containing series 11a-h revealed that the compound with the *para*-methoxy phenyl moiety 11d was, interestingly, the most active among this series with IC_50_ = 4.15, 2.23, and 1.94 *μ*M. Members with *para*-*N*,*N*-di-methyl 11 g, *para*-chloro 11f, or *para*-nitro 11c substituents on the phenyl ring were reported to possess high cytotoxic effects with IC_50_ ranging from 3.47 to 7.31 *μ*M. Inversely, the activity was strongly decreased to 20.17, 31.12, and 34.9 *μ*M in the case of the *para*-hydroxy substituted compound 11h.

The *in vitro* antiproliferative effect of the trifluoroacetyl derivative 10 was better than that of sorafenib considering the MCF-7 and HepG-2 cell lines with IC_50_ values of 3.55 3.16 *μ*M, respectively, while its activity was almost equal to sorafenib regarding the HCT-116 cell line with an IC_50_ value of 5.49 *μ*M.

Concerning the oxadiazole linker containing series 18a-c, it was noticed that the compound bearing an unsubstituted phenyl ring as a hydrophobic tail 18b was the most effective against the three tested cancer cell lines with IC_50_ values of 2.67, 6.11, and 4.19 *μ*M. The cytotoxic activity was lowered with the *para*-chloro substituted phenyl moiety 18a (IC_50_ = 5.81, 8.29, and 5.13 *μ*M), while, it was the least in the member with a benzyl moiety 18c (IC50 = 19.37, 8.59, and 21.72 *μ*M).

The cytotoxicity values of the synthesized compounds against the WI-38 cell line (normal human lung fibroblasts) were also evaluated *in vitro*. The results revealed that the tested compounds have low toxicity against WI-38 with IC_50_ values ranging from 50.28 to 157.19 *μ*M.

#### 2.2.2. In Vitro VEGFR-2 Enzyme Assay Inhibition

In order to determine the potency of the design compounds as VEGFR-2 inhibitors, we decided to investigate the inhibitory effect of the most active cytotoxic members against VEGFR-2 with sorafenib as positive control [48]. Results are illustrated in Table 2. Most of the tested compounds showed moderate to potent inhibition of VEGFR-2 in comparison to the reference drug, sorafenib (IC_50_ = 2.36 *μ*M). The IC_50_ values of the tested compounds ranged from 2.17 to 8.09 *μ*M. In light of the VEGFR-2 inhibitory results, it was obvious that compound 7a significantly inhibited VEGFR-2 tyrosine kinase with an IC_50_ value of 2.17 *μ*M, a value which was lower than that of sorafenib, indicating the ability of the designed compound to block the VEGFR-2 signaling pathway with subsequent inhibition of angiogenesis. The cyclohexyl-containing compound 5 exhibited an inhibitory effect (IC_50_ = 2.46 *μ*M) that was almost like sorafenib. Members 11d and 7b, with a *para*-methoxy substituted phenyl moiety, possessed potent VEGFR-2 inhibitory activity with IC_50_ values of 2.74 and 3.45 *μ*M, respectively. The halogenated derivatives 7l, 11f, and 18a showed strong inhibitory effects with IC_50_ values of 3.84, 4.17, and 5.23 *μ*M, respectively. The rest of the tested compounds exhibited moderate inhibitory activities with IC_50_ values ranging from 6.12 to 8.09 *μ*M.

#### 2.2.3. Apoptotic Marker Analysis

**Figure 3:**
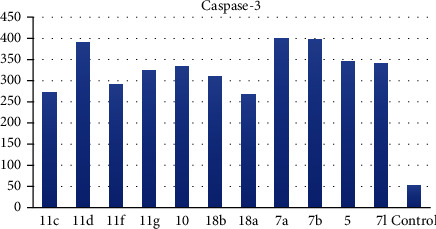
Levels of caspase-3 in the cell supernatant after exposure to the synthesized compounds on HepG-2 cells.

*(1) Effects on the Levels of Active Caspase-3*. A significant mechanism by which anticancer agents can affect cancer cells is apoptosis. Apoptosis is a process of automated cell death. The apoptosis mechanism is typically mediated by a group of proteases called caspases. Caspase-3 is activated in every mammalian cell type provoking the cell to die, considered one of the apoptosis marks. Therefore, the level of caspase-3 was analyzed following exposure of the HepG-2 cell to the most active cytotoxic candidates. The tested compounds caused a potent increase in caspase-3 level to a concentration ranging from 267.64 to 397.61 Pg/ml (Table 3). Compounds 7a, 7b, and 11d exhibited more than 7-fold increases in the caspase-3 level when compared with the control indicating their ability to activate caspase-3 enzymes and dramatically reinforce apoptosis of cancer cells (Figure 3).

**Figure 4:**
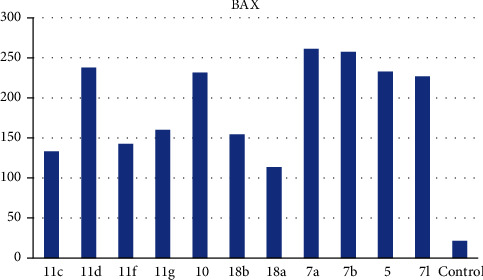
BAX levels caused by the tested compounds on HepG-2.

**Figure 5:**
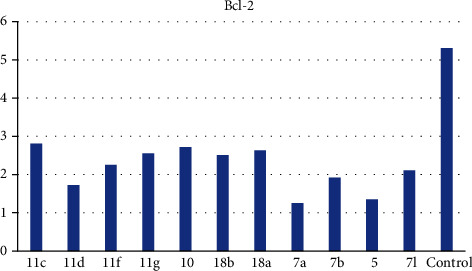
Bcl-2 levels caused by the tested compounds on HepG-2.

*(2) Effects on Bcl-2 Family Proteins*. The Bcl-2 family proteins are vital regulators of apoptosis cell death. Members of this family include the proapoptotic factor Bax which promotes cell death and the prosurvival (antiapoptotic) Bcl-2 which suppresses cell death. Bax and Bcl-2 cellular levels were analyzed for the most potent cytotoxic compounds in HepG-2 cells. Results are expressed in Table 3. The tested compounds caused an increase in the proapoptotic factor Bax by 5.25- to 12.11-fold (Figure 4), while a decrease in the antiapoptotic protein Bcl-2 concentration was observed by 0.23- to 0.52-fold in comparison to the control (Figure 5) indicating that the tested members promote apoptosis *via* both the intrinsic and extrinsic pathways.

**Figure 6:**
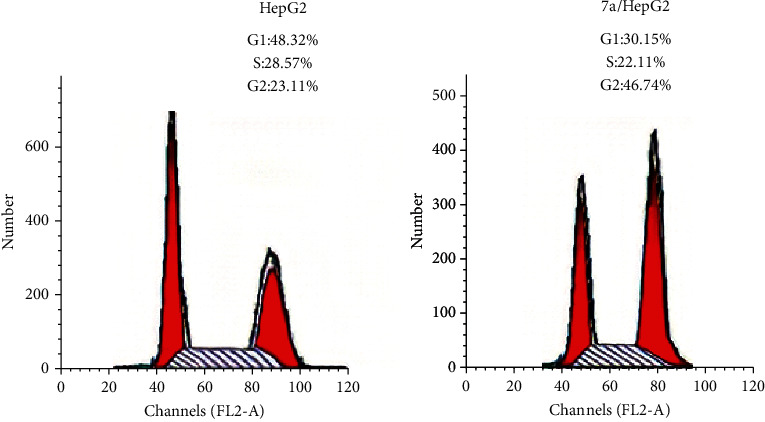
Cell cycle analysis of HepG-2 cells treated with compound 7a.

*(3) Cell Cycle Analysis*. For further investigation of the apoptotic capacity of compound 7a, flow cytometric analysis was carried out on the HepG-2 cancer cell line treated with the tested compound at its IC_50_ concentrations using untreated HepG-2 cancer cells as a negative control. An elevation of apoptotic cells at the pre-G1 phase was observed (10.5%) in comparison to the control HepG-2 cells (2.0%). Accumulation of cells at the G2-M phase for 7a was estimated to be 46.74% versus 23.11% accumulation for control HepG-2 cells. On the contrary, the percentage at the S phase of treated cells was reduced (22.11%) compared to untreated cells (28.57%) (Figure 6).

**Figure 7:**
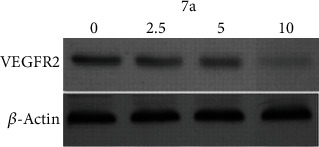
Effect of 7a on VEGFR-2 protein expression.

*(4) Effect of Compound 7a on VEGFR-2 Protein Expression*. Western blotting found that 10 h exposures to 7a in HepG-2 cells downregulated the expression of VEGFR-2 protein in comparison to the blank (Figure 7).

**Figure 8:**
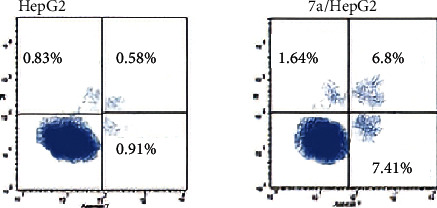
Effect of compound 7a on the percentage of annexin V-FITC-positive staining in HepG-2 cells.

*(5) Apoptotic Cell Subpopulation Determination*. To support the fact that derivative 7a induces apoptosis in the HepG-2 cancer cell line, annexin V-FITC and PI double-staining assay protocol was used.  Figure 8 displays the percentage of early and late apoptotic stages following the treatment of HepG-2 cells with the IC_50_ dose of compound 7a (2.17 *μ*M) for 24 h. The percentage of early and late apoptotic populations increased from 0.91% and 0.58%, respectively, in control untreated cells to 7.41% and 6.80% in 7a-treated cells. This is evidence that the antiproliferative activities are associated with a proapoptotic effect of the tested compound.

#### 2.2.4. In Vitro Immunomodulatory Assay

*(1) Estimation of Human Tumor Necrosis Factor Alpha (TNF-α) in HepG-2 Supernatant*. The effect of the most active cytotoxic compounds on TNF-*α* was also examined aiming to study their immunomodulatory effect on HepG-2 cells. Celecoxib, a potent inhibitor of TNF-*α-*induced cytokine expressions, was used as a positive control [49]. The data represented in Table 3 revealed that production of TNF-*α* was significantly decreased in HepG-2 cells exposed to celecoxib and the synthesized compounds compared to the untreated HepG-2 cells. Among the tested compounds, compound 7a caused a remarkably significant reduction in TNF-*α* levels (87%) in comparison to celecoxib (86%). The rest of the tested compounds exhibited moderate to potent TNF-*α* level reduction in relation to the reference drug.

#### 2.2.5. Effect on Vascular Smooth Muscle Cell Proliferation in a Concentration-Dependent Manner

**Figure 9:**
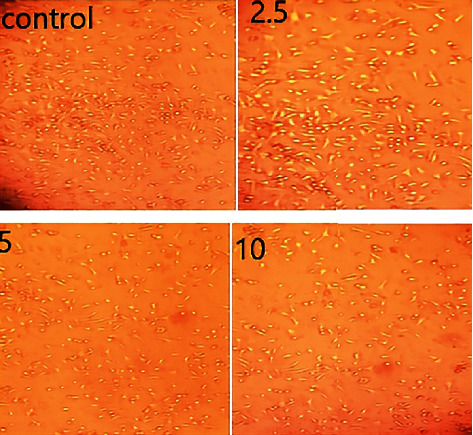
Effect of compound 7a on VSMC proliferation.

In order to examine its effect on the proliferation of vascular smooth muscle cells (VSMCs), VSMCs were treated with increasing concentrations of compound 7a (2.5, 5, and 10 *μ*M) for 48 h. The cellular proliferation results are illustrated in Figure 9. Results revealed that compound 7a had a significant antiproliferative effect at all concentrations tested, although the effect was more profound with concentrations of 5 *μ*M or higher.

### 2.3. Docking Study

**Figure 10:**
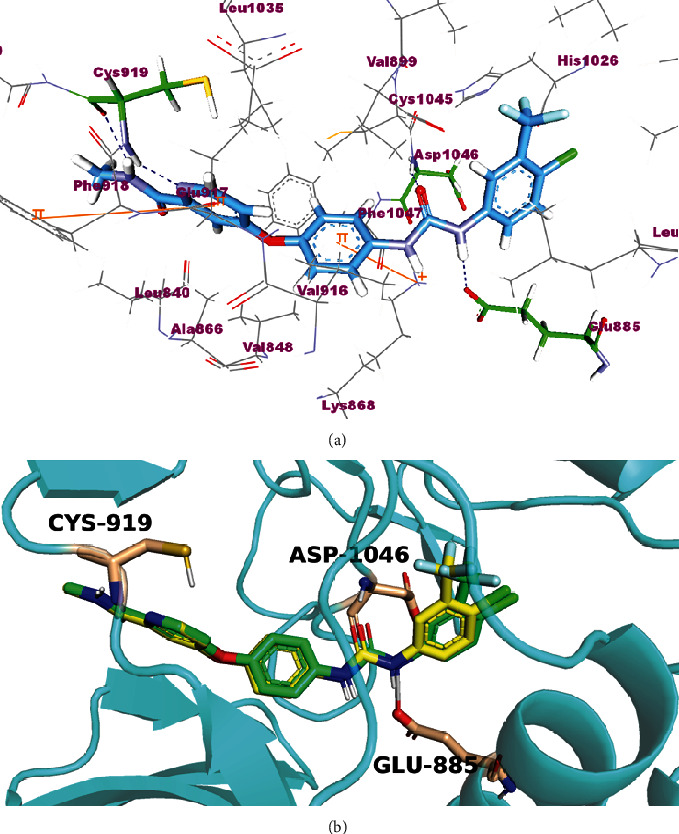
(a) Docking pose of sorafenib. (b) Superimposition of the cocrystallized pose (green) and the docking pose (yellow) of the same ligand.

The newly synthesized compounds underwent molecular docking studies in order to shed light on their interaction with the ATP pocket of VEGFR-2 using Molecular Operating Environment (MOE, 2014) software. The X-ray crystallographic structure of VEGFR-2 (PDB ID: 4ASD) with its cocrystalized type II PK inhibitor, sorafenib, was downloaded from the Protein Data Bank (PDB). Redocking of the cocrystalized ligand, sorafenib, was initially performed aiming at validating the used docking protocol. The simulation of the redocked ligand successfully regenerated the same binding mode of the cocrystalized one inside the VEGFR-2 active site with an RMSD of 0.65 Å. The redocked poses reproduced all crucial interactions carried out by the cocrystallized ligand with the VEGFR-2 active site including Cys919, Glu885, and Asp1046 residues. Results of the validation step signaled that the used docking protocol is suitable for a molecular docking study of the designed compounds in the VEGFR-2 active pocket (Figure 10).

A general examination of docking results revealed that the designed compounds exhibited a binding pattern comparable to that of the native ligand with predicted binding energy scores ranging from -27.18 to -56.91 kcal/mol.

**Figure 11:**
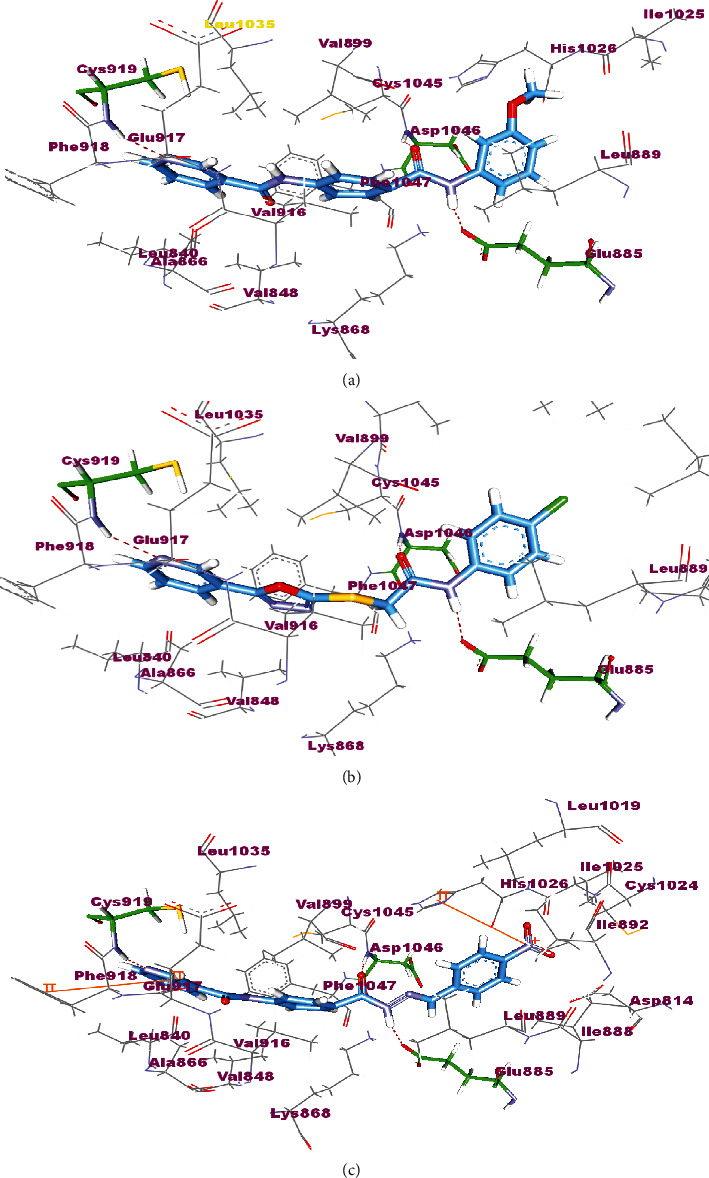
The proposed binding pose for the interaction of 7a (a), 18a (b), and 18c (c) in the active site of VEGFR-2 (PDB ID: 4ASD).

The synthesized compounds occupied the same orientation of sorafenib. They interacted with the key amino acids in the active site of VEGFR-2 in the same manner achieved by sorafenib. The compounds nicotinamide or pyridin-3-yl-oxadiazol moieties were directed toward the hinge region of the active side forming a hydrogen bond between their pyridinyl nitrogen and Cys919 residue. On the other side, the carbamoyl phenyl, hydrazine-carbonyl phenyl, and thioacetamide scaffolds of the designed compounds were accommodated in the pocket central area, i.e., the gate area, interacting via a hydrogen bond with the carboxylate side chain of Glu885 and another one with the NH moiety of Asp1046 of the conserved DFG motif in VEGFR-2. The orientation of the later moieties fitted the compounds' hydrophobic substituents in the hydrophobic allosteric pocket in the active site allowing these hydrophobic substituents to interact with hydrophobic side chains of Ile888, Leu889, Ile892, Val898, Val899, Leu1019, and Ile1044 amino acids lining the back pocket of VEGFR-2 (Figure 11).

## 3. Conclusion

Three series of pyridine-containing compounds have been synthesized and tested for their *in vitro* cytotoxic activity against HepG-2, MCF-7, and HCT-116 cancer cell lines by an MTT assay. In vitro IC_50_ determination of the best eleven derivatives were evaluated against the VEGFR-2 enzyme. Compound 7a was found to be the most potent especially against HepG-2 cells. A caspase-3 activation assay was performed for the most active members in HepG-2 cells. Compound 7a significantly increased the caspase-3 level by 7.80-fold (401.15 Pg/ml) compared to the control (51.38 Pg/ml). In addition, Bax and Bcl-2 concentration levels were estimated. Compound 7a results showed a titer increase of the proapoptotic protein Bax (261.4 Pg/ml) and a decrease of the antiapoptotic protein Bcl-2 (1.25 Pg/ml) compared to the untreated HepG-2 cells. Furthermore, compound 7a arrested the cell cycle in the G2/M phase with induction of apoptosis in HepG-2 cells. The immunomodulatory effect of the most active members was also evaluated with a significant reduction in TNF-*α* by 87% for compound 7a. Compound 7a also caused a potent inhibitory effect on smooth muscle proliferation. The biological results were supported by docking studies via prediction of the possible binding pattern of the target compounds with VEGFR-2 active sites.

## 4. Experimental Section

### 4.1. Chemistry

Determination of melting points was carried out using a Gallen lamp melting point apparatus and is uncorrected. Reaction progress was monitored by TLC (Merck, Germany), and the spots were detected by exposure to UV lamp at *λ* 254 nm. IR spectra were recorded on a Pye Unicam SP 1000 IR spectrophotometer using KBr discs and expressed in wave number (cm^−1^). ^1^H and ^13^C NMR spectra were recorded with a Bruker Advance 400 spectrophotometer operating at 400 MHz and 100 MHz, respectively, and the chemical shifts were given in *δ* as parts per million (ppm) downfield from tetramethylsilane (TMS) as internal standard. The mass spectra were recorded on a Varian MAT 311-A (70 eV).

The following compounds were prepared according to the reported procedures: nicotinoyl chloride 2, 4-(nicotinamido)benzoic acid 3, 4-(nicotinamido)benzoyl chloride 4, ethyl 4-(nicotinamido)benzoate 8, N-(4-(hydrazine-carbonyl)phenyl)nicotinamide 9, methyl nicotinate 12, nicotinohydrazide 13, 5-(pyridin-3-yl)-1,3,4-oxadiazole-2-thiol 14, potassium 5-(pyridin-3-yl)-1,3,4-oxa-diazole-2-thiolate 15, and *N*-aryl-2-chloroacetamides 17a-c.

#### 4.1.1. General Procedure for Synthesis of Compounds 5, 6, and 7a-l

To a solution of 4-(nicotinamido)benzoyl chloride 4 (0.26 g, 0.001 mol) in acetonitrile (25 ml), the appropriate alkyl amine, namely, cyclohexylamine or benzylamine, or the appropriate aniline derivative, namely, 3-methoxyaniline, 4-methoxyaniline, 2-methoxyaniline, aniline, 4-chloroaniline, *o*-toluidine, 2,4-dichloroaniline, *m*-toluidine, 2-nitroaniline, *p*-toluidine, 2,4-dimethylaniline, or 4-fluoroaniline was added. TEA was added to the mixture in a catalytic proportion. The reaction mixture was then refluxed for 2 h. The reaction mixture was poured onto ice water, and the residue was filtered off and crystallized from ethanol.

*(1) N-(4-(Cyclohexylcarbamoyl)phenyl)nicotinamide (5)*. Yield: 87%; melting point: 239-242°C; HPLC purity 96.93%; IR *υ*_max_/cm^−1^: 3321 (NH), 1651, 1630 (C=O); ^1^H NMR (DMSO-*d*6, 400 MHz) *δ* ppm: 1.13 (m, 1H), 1.26-1.37 (m, 4H), 1.61 (m, 1H), 1.75 (m, 2H), 2.84 (m, 2H), 3.77 (m, 1H), 7.59 (dd, *J* = 8.0, 8.0 Hz, 1H), 7.85 (m, 4H), 8.15 (d, *J* = 8.0 Hz, 1H), 8.31 (t, *J* = 8.0 Hz, 1H), 8.78 (d, *J* = 6.8 Hz, 1H), 9.13 (s, 1H), 10.67 (s, 1H); ^13^C NMR (DMSO-*d*6) *δ* (ppm): 25.47 (2C), 25.77, 32.97 (2C), 48.76, 119.83 (2C), 124.00, 128.51 (2C), 130.48, 130.87, 136.04, 141.74, 149.23, 152.74, 164.78, 165.20.

*(2) N-(4-(Benzylcarbamoyl)phenyl)nicotinamide (6)*. Yield: 74%; melting point: 229-232°C; HPLC purity 98.17%; IR *υ*_max_/cm^−1^: 3313, 3174 (NH), 1654, 1632 (C=O); ^1^H NMR (DMSO-*d*6, 400 MHz) *δ* ppm: 4.5 (s, 2H), 7.26 (s, 1H), 7.34 (m, 4H), 7.59 (dd, *J* = 8.0, 7.6 Hz, 1H), 7.89 (d, *J* = 8.4 Hz, 2H), 7.94 (d, *J* = 8.4 Hz, 2H), 8.32 (d, *J* = 7.6 Hz, 1H), 8.79 (s, 1H), 9.02 (t, *J* = 6.4 Hz, 1H), 9.14 (s, 1H), 10.68 (s, 1H); ^13^C NMR (DMSO-*d*6) *δ* (ppm): 43.05, 119.97 (2C), 124.02, 127.20, 127.71 (2C), 128.54 (2C), 128.76 (2C), 129.98, 130.87, 136.05, 140.27, 141.98, 149.23, 152.77, 164.84, 166.12.

*(3) N-(4-((3-Methoxyphenyl)carbamoyl)phenyl)nicotinamide (7a)*. Yield: 84%; melting point: 188-191°C; HPLC purity 96.21%; IR *υ*_max_/cm^−1^: 3348 (NH), 1665, 1651 (C=O); ^1^H NMR (DMSO-*d*6, 400 MHz) *δ* ppm: 3.77 (s, 3H), 6.68 (d, *J* = 7.2 Hz, 1H), 7.24 (t, *J* = 8.0 Hz, 1H), 7.40 (d, *J* = 8.0 Hz, 1H), 7.51 (s, 1H), 7.59 (dd, *J* = 8.0, 8.0 Hz, 1H), 7.95 (d, *J* = 8.4 Hz, 2H), 8.01 (d, *J* = 8.4 Hz, 2H), 8.33 (d, *J* = 7.6 Hz, 1H), 8.79 (s, 1H), 9.16 (s, 1H), 10.18 (s, 1H), 10.74 (s, 1H); ^13^C NMR (DMSO-*d*6) *δ* (ppm): 55.46, 106.46, 109.51, 113.01, 119.97 (2C), 124.02, 129.03 (2C), 130.39, 130.60, 130.84, 136.08, 140.94, 142.35, 149.26, 152.80, 159.89, 164.91, 165.38.

*(4) N-(4-((4-Methoxyphenyl)carbamoyl)phenyl)nicotinamide (7b)*. Yield: 71%; melting point: 249-251°C; HPLC purity 98.65%; IR *υ*_max_/cm^−1^: 3336 (NH), 1662, 1640 (C=O); ^1^H NMR (DMSO-*d*6, 400 MHz) *δ* ppm: 3.76 (s, 3H), 6.93 (d, *J* = 8.4 Hz, 2H), 7.59 (t, *J* = 6.0 Hz, 1H), 7.69 (d, *J* = 8.4 Hz, 2H), 7.93 (d, *J* = 8.0 Hz, 2H), 8.00 (d, *J* = 8.0 Hz, 2H), 8.33 (d, *J* = 7.2 Hz, 1H), 8.79 (s, 1H), 9.15 (s, 1H), 10.09 (s, 1H), 10.72 (s, 1H); mass (*m*/*z*): 347.06 (M^+^, 32%), 217.06 (100%).

*(5) N-(4-((2-Methoxyphenyl)carbamoyl)phenyl)nicotinamide (7c)*. Yield: 76%; melting point: 185-187°C; HPLC purity 98.53%; IR *υ*_max_/cm^−1^: 3433, 3305 (NH), 1681, 1685 (C=O); ^1^H NMR (DMSO-*d*6, 400 MHz) *δ* ppm: 3.86 (s, 3H), 6.99 (t, *J* = 8.4 Hz, 1H), 7.12 (d, *J* = 8.0 Hz, 1H), 7.19 (d, *J* = 7.6 Hz, 1H), 7.61 (s, 1H), 7.80 (d, *J* = 6.8 Hz, 1H), 7.95 (d, *J* = 8.4 Hz, 2H), 8.00 (d, *J* = 8.4 Hz, 2H), 8.33 (d, *J* = 6.4 Hz, 1H), 8.80 (s, 1H), 9.15 (s, 1H), 9.39 (s, 1H), 10.73 (s, 1H); ^13^C NMR (DMSO-*d*6) *δ* (ppm): 56.19, 111.82, 120.08 (2C), 120.69, 124.06, 124.69, 126.09, 127.35, 128.84 (2C), 129.99, 130.86, 136.10, 142.34, 149.23, 151.87, 152.81, 164.82, 164.93.

*(6) N-(4-(Phenylcarbamoyl)phenyl)nicotinamide (7d)*. Yield: 82%; melting point: 232-235°C; HPLC purity 98.53%; IR *υ*_max_/cm^−1^: 3344 (NH), 1665, 1647 (C=O); ^1^H NMR (DMSO-*d*6, 400 MHz) *δ* ppm: 7.09 (t, *J* = 7.6 Hz, 1H), 7.36 (t, *J* = 8.0 Hz, 2H), 7.62 (dd, *J* = 7.6, 7.6 Hz, 1H), 7.79 (d, *J* = 8.0 Hz, 2H), 7.95 (d, *J* = 8.8 Hz, 2H), 8.02 (d, *J* = 8.8 Hz, 2H), 8.36 (d, *J* = 6.4 Hz, 1H), 8.81 (s, 1H), 9.17 (s, 1H), 10.22 (s, 1H), 10.77 (s, 1H); mass (*m*/*z*): 317.33 (M^+^, 11%), 240.05 (100%).

*(7) N-(4-((4-Chlorophenyl)carbamoyl)phenyl)nicotinamide (7e)*. Yield: 79%; melting point: 272-274°C; HPLC purity 98.40%; IR *υ*_max_/cm^−1^: 3325 (NH), 1663, 1647 (C=O); ^1^H NMR (DMSO-*d*6, 400 MHz) *δ* ppm: 7.40 (d, *J* = 8.4 Hz, 2H), 7.59 (t, *J* = 6.4 Hz, 1H), 7.85 (d, *J* = 8.4 Hz, 2H), 7.96 (d, *J* = 8.0 Hz, 2H), 8.02 (d, *J* = 8.0 Hz, 2H), 8.36 (d, *J* = 7.2 Hz, 1H), 8.78 (s, 1H), 9.17 (s, 1H), 10.45 (s, 1H), 10.94 (s, 1H); mass (*m*/*z*): 351.12 (M^+^, 26%), 352.19 (M^+1^, 8%), 197 (100%).

*(8) N-(4-(o-Tolylcarbamoyl)phenyl)nicotinamide (7f)*. Yield: 88%; melting point: 194-197°C; HPLC purity 97.21%; IR *υ*_max_/cm^−1^: 3275, 3252 (NH), 1681, 1643 (C=O); ^1^H NMR (DMSO-*d*6, 400 MHz) *δ* ppm: 2.26 (s, 3H), 7.18 (dt, *J* = 7.2, 7.2 Hz, 1H), 7.23 (dt, *J* = 7.6, 7.6 Hz, 1H), 7.28 (dd, *J* = 7.6, 7.2 Hz, 1H), 7.35 (dd, *J* = 7.2, 8.0 Hz, 1H), 7.59 (dd, *J* = 6.8, 8.0 Hz, 1H), 7.95 (d, *J* = 8.8 Hz, 2H), 8.03 (d, *J* = 8.8 Hz, 2H), 8.34 (d, *J* = 8.0 Hz, 1H), 8.79 (s, 1H), 9.16 (s, 1H), 9.86 (s, 1H), 10.76 (s, 1H); ^13^C NMR (DMSO-*d*6) *δ* (ppm): 18.44, 120.04 (2C), 124.03, 126.39, 126.47, 127.10, 128.97 (2C), 130.05, 130.78, 130.86, 134.19, 136.09, 136.98, 142.31, 149.27, 152.79, 164.90, 165.16.

*(9) N-(4-((2,4-Dichlorophenyl)carbamoyl)phenyl)nicotinamide (7g)*. Yield: 80%; melting point: 189-192°C; HPLC purity 97.56%; IR *υ*_max_/cm^−1^: 3352, 3313 (NH), 1681 (C=O); ^1^H NMR (DMSO-*d*6, 400 MHz) *δ* ppm: 7.18 (d, *J* = 8.0 Hz, 1H), 7.37 (d, *J* = 8.0 Hz, 1H), 7.60 (d, *J* = 8.4 Hz, 2H), 7.97 (d, *J* = 7.6 Hz, 2H), 8.04 (d, *J* = 7.6 Hz, 2H), 8.37 (d, *J* = 7.6 Hz, 1H), 8.82 (s, 1H), 9.18 (s, 1H), 10.10 (s, 1H), 10.79 (s, 1H); mass (*m*/*z*): 386.79 (M^+^, 41%), 388.14 (M^+2^, 17%), 199 (100%).

*(10) N-(4-(m-Tolylcarbamoyl)phenyl)nicotinamide (7h)*. Yield: 85%; melting point: 217-219°C; HPLC purity 95.48%; IR *υ*_max_/cm^−1^: 3348 (NH), 1670, 1651 (C=O); ^1^H NMR (DMSO-*d*6, 400 MHz) *δ* ppm: 2.32 (s, 3H), 6.91 (d, *J* = 7.6 Hz, 1H), 7.22 (t, *J* = 8.0 Hz, 1H), 7.58 (m, 2H), 7.66 (s, 1H), 7.96 (d, *J* = 8.4 Hz, 2H), 8.02 (d, *J* = 8.4 Hz, 2H), 8.34 (d, *J* = 8.0 Hz, 1H), 8.80 (s, 1H), 9.17 (s, 1H), 10.14 (s, 1H), 10.75 (s, 1H); mass (*m*/*z*): 331.34 (M^+^, 14%), 123 (100%).

*(11) N-(4-((2-Nitrophenyl)carbamoyl)phenyl)nicotinamide (7i)*. Yield: 87%; melting point: 198-201°C; HPLC purity 95.60%; IR *υ*_max_/cm^−1^: 3329, 3190 (NH), 1670 (C=O), 1597, 1338 (NO_2_); ^1^H NMR (DMSO-*d*6, 400 MHz) *δ* ppm: 7.43 (t, *J* = 8.4 Hz, 1H), 7.61 (d, *J* = 8.0 Hz, 1H), 7.77 (d, *J* = 7.6 Hz, 2H), 8.01 (m, 5H), 8.34 (d, *J* = 7.2 Hz, 1H), 8.80 (s, 1H), 9.16 (s, 1H), 10.76 (s, 1H), 10.79 (s, 1H); ^13^C NMR (DMSO-*d*6) *δ* (ppm): 120.15 (2C), 124.04, 125.47, 125.88, 126.27, 128.94, 129.19 (2C), 130.79, 132.19, 134.52, 136.11, 142.97, 143.22, 149.28, 152.85, 164.99, 165.17.

*(12) N-(4-(p-Tolylcarbamoyl)phenyl)nicotinamide (7j)*. Yield: 76%; melting point: 219-222°C; HPLC purity 96.21%; IR *υ*_max_/cm^−1^: 3348 (NH), 1662, 1647 (C=O); ^1^H NMR (DMSO-*d*6, 400 MHz) *δ* ppm: 2.29 (s, 3H), 7.15 (d, *J* = 8.0 Hz, 2H), 7.58 (d, *J* = 7.2 Hz, 1H), 7.67 (d, *J* = 8.0 Hz, 2H), 7.95 (d, *J* = 8.4 Hz, 2H), 8.01 (d, *J* = 8.4 Hz, 2H), 8.35 (d, *J* = 7.6 Hz, 1H), 8.79 (s, 1H), 9.16 (s, 1H), 10.17 (s, 1H), 10.83 (s, 1H); mass (*m*/*z*): 332 (M^+^, 17%), 241 (100%).

*(13) N-(4-((2,4-Dimethylphenyl)carbamoyl)phenyl)nicotinamide (7k)*. Yield: 85%; melting point: 254-256°C; IR *υ*_max_/cm^−1^: 3278, 3182 (NH), 1665, 1635 (C=O); ^1^H NMR (DMSO-*d*6, 400 MHz) *δ* ppm: 2.21 (s, 6H), 7.14 (s, 3H), 7.59 (t, *J* = 6.0 Hz, 1H), 7.95 (d, *J* = 8.0 Hz, 2H), 8.05 (d, *J* = 8.0 Hz, 2H), 8.35 (d, *J* = 7.6 Hz, 1H), 8.80 (s, 1H), 9.17 (s, 1H), 9.75 (s, 1H), 10.75 (s, 1H); ^13^C NMR (DMSO-*d*6) *δ* (ppm): 18.15, 18.59, 120.11 (2C), 124.03, 127.11, 128.19 (2C), 128.84 (2C), 129.96, 130.83, 135.89, 136.09, 136.15 (2C), 142.21, 149.26, 152.80, 164.87, 164.93; mass (*m*/*z*): 345.11 (M^+^, 27%), 240 (100%).

*(14) N-(4-((4-Fluorophenyl)carbamoyl)phenyl)nicotinamide (7l)*. Yield: 81%; melting point: 259-261°C; HPLC purity 96.21%; IR *υ*_max_/cm^−1^: 3340 (NH), 1668, 1643 (C=O); ^1^H NMR (DMSO-*d*6, 400 MHz) *δ* ppm: 7.40 (dt, *J* = 6.8, 6.4 Hz, 2H), 7.59 (dd, *J* = 7.6, 8.0 Hz, 1H), 7.82 (dt, *J* = 7.6, 8.0 Hz, 2H), 7.95 (d, *J* = 8.0 Hz, 2H), 8.01 (d, *J* = 8.0 Hz, 2H), 8.33 (d, *J* = 8.0 Hz, 1H), 8.79 (s, 1H), 9.15 (s, 1H), 10.28 (s, 1H), 10.74 (s, 1H).

#### 4.1.2. *N*-(4-(2-(2,2,2-Trifluoroacetyl)hydrazine-1-carbonyl)phenyl)nicotinamide (10)

*N*-(4-(Hydrazinecarbonyl)phenyl)nicotinamide 9 (0.256 g, 0.001 mol) in DCM (25 ml) was stirred at room temperature with trifluoroacetic anhydride (0.001 mol) for 4 h. The formed precipitate was then collected and crystallized from ethanol.

Yield: 85%; melting point: 287-290°C; HPLC purity 96.38%; IR *υ*_max_/cm^−1^: 3348, 3232 (NH), 1751, 1662 (C=O); ^1^H NMR (DMSO-*d*6, 400 MHz) *δ* ppm: 7.59 (dd, *J* = 8.0, 8.0 Hz, 1H), 7.94 (d, *J* = 8.4 Hz, 2H), 8.00 (d, *J* = 8.4 Hz, 2H), 8.32 (d, *J* = 8.0 Hz, 1H), 8.79 (s, 1H), 9.14 (s, 1H), 10.75 (s, 2H), 11.75 (s, 1H); ^13^C NMR (DMSO-*d*6) *δ* (ppm): 120.15 (2C), 124.04, 127.18, 128.89 (2C), 130.81, 136.10 (2C), 142.91, 149.25 (2C), 152.83, 164.99, 165.13.

#### 4.1.3. General Procedure for Synthesis of Compounds 11a-h

A mixture of *N*-(4-(hydrazinecarbonyl)phenyl)nicotinamide 9 (0.256 g, 0.001 mol) and the appropriate aromatic aldehyde (0.001 mol), namely, 4-fluorobenzaldehyde, benzaldehyde, 4-nitrobenzaldehyde, 4-methoxybenzaldehyde, 2,6-dichloro-benzaldehyde, 2-chlorobenzaldehyde, 4-(dimethylamino)benzaldehyde, or 4-hydroxybenzaldehyde (0.001 mol) was refluxed in absolute ethanol (30 ml) containing a few drops of glacial acetic acid for 2 h. After reaction completion, the mixture was cooled to room temperature, and the formed precipitate was then filtered, dried, and recrystallized from ethanol.

*(1) (E)-N-(4-(2-(4-Fluorobenzylidene)hydrazine-1-carbonyl)phenyl)-nicotinamide (11a)*. Yield: 85%; melting point: 263-265°C; IR *υ*_max_ /cm^−1^: 3375, 3242 (NH), 1664 (C=O); ^1^H NMR (DMSO-*d*6, 400 MHz) *δ* ppm: 7.32 (d, *J* = 8.0 Hz, 2H), 7.59 (dd, *J* = 6.8, 7.2 Hz, 1H), 7.81 (d, *J* = 8.0 Hz, 2H), 7.96 (m, 4H), 8.33 (d, *J* = 8.0 Hz, 1H), 8.48 (s, 1H), 8.80 (s, 1H), 9.15 (s, 1H), 10.74 (s, 1H), 11.86 (s, 1H); ^13^C NMR (DMSO-*d*6) *δ* (ppm): 116.29 (2C), 120.05 (2C), 124.03 (2C), 128.99 (2C), 129.66, 130.82, 136.07 (2C), 142.50, 146.80, 149.25 (2C), 152.82, 162.99, 164.79, 164.91.

*(2) (E)-N-(4-(2-Benzylidenehydrazine-1-carbonyl)phenyl)-nicotinamide (11b)*. Yield: 81%; melting point: 260-262°C; HPLC purity 96.50%; IR *υ*_max_ /cm^−1^: 3332, 3259 (NH), 1647 (C=O); ^1^H NMR (DMSO-*d*6, 400 MHz) *δ* ppm: 7.47 (d, *J* = 6.4 Hz, 2H), 7.49 (d, *J* = 7.6 Hz, 1H), 7.59 (dd, *J* = 8.0, 8.0 Hz, 1H), 7.81 (d, *J* = 6.8 Hz, 2H), 7.94 (d, *J* = 8.8 Hz, 2H), 7.97 (d, *J* = 8.8 Hz, 2H), 8.32 (d, *J* = 8.0 Hz, 1H), 8.48 (s, 1H), 8.79 (s, 1H), 9.15 (s, 1H), 10.74 (s, 1H), 11.85 (s, 1H); ^13^C NMR (DMSO-*d*6) *δ* (ppm): 120.07 (2C), 124.06 (2C), 127.56 (2C), 129.00, 129.35 (2C), 130.82, 134.84, 136.09 (2C), 142.84, 147.98, 149.23 (2C), 152.82, 163.02, 164.93.

*(3) (E)-N-(4-(2-(4-Nitrobenzylidene)hydrazine-1-carbonyl)phenyl)-nicotinamide (11c)*. Yield: 89%; melting point: 291-293°C; HPLC purity 96.62%; IR *υ*_max_ /cm^−1^: 3360, 3294 (NH), 1670 (C=O); ^1^H NMR (DMSO-*d*6, 400 MHz) *δ* ppm: 7.60 (dd, *J* = 8.0, 7.2 Hz, 1H), 7.85 (s, 1H), 7.95 (d, *J* = 8.4 Hz, 2H), 7.99 (s, 1H), 8.01 (d, *J* = 8.4 Hz, 2H), 8.33 (m, 3H), 8.57 (s, 1H), 8.80 (s, 1H), 9.15 (s, 1H), 10.75 (s, 1H), 12.15 (s, 1H); mass (*m*/*z*): 390.18 (M^+^, 21%), 197 (100%).

*(4) (E)-N-(4-(2-(4-Methoxybenzylidene)hydrazine-1-carbonyl)phenyl)-nicotinamide (11d)*. Yield: 78%; melting point: 264-267°C; HPLC purity 97.33%; IR *υ*_max_ /cm^−1^: 3325, 3251 (NH), 1660, 1643 (C=O); ^1^H NMR (DMSO-*d*6, 400 MHz) *δ* ppm: 3.82 (s, 3H), 7.03 (d, *J* = 8.4 Hz, 2H), 7.59 (dd, *J* = 7.6, 7.6 Hz, 1H), 7.68 (d, *J* = 8.4 Hz, 2H), 7.93 (d, *J* = 8.8 Hz, 2H), 7.96 (d, *J* = 8.8 Hz, 2H), 8.32 (d, *J* = 8.0 Hz, 1H), 8.42 (s, 1H), 8.79 (s, 1H), 9.14 (s, 1H), 10.73 (s, 1H), 11.72 (s, 1H); ^13^C NMR (DMSO-*d*6) *δ* (ppm): 55.77, 114.82 (2C), 120.06 (2C), 124.05 (2C), 127.04, 128.92 (2C), 130.82, 136.08 (2C), 142.37, 147.89, 149.23 (2C), 152.81, 161.28, 162.85, 164.91.

*(5) (E)-N-(4-(2-(2,6-Dichlorobenzylidene)hydrazine-1-carbonyl)phenyl)-nicotinamide (11e)*. Yield: 74%; melting point: 283-285°C; HPLC purity 96.77%; IR *υ*_max_ /cm^−1^: IR (KBr) cm^−1^: 3294, 3170 (NH), 1651 (C=O); ^1^H NMR (DMSO-*d*6, 400 MHz) *δ* ppm: 7.44 (t, *J* = 8.0 Hz, 1H), 7.59 (m, 3H), 7.94 (d, *J* = 8.4 Hz, 2H), 8.00 (d, *J* = 8.4 Hz, 2H), 8.33 (d, *J* = 7.6 Hz, 1H), 8.69 (s, 1H), 8.79 (s, 1H), 9.15 (s, 1H), 10.75 (s, 1H), 12.14 (s, 1H); mass (*m*/*z*): 412.71 (M^+^, 29%), 414.02 (M^+1^, 17%), 344 (100%).

*(6) (E)-N-(4-(2-(2-Chlorobenzylidene)hydrazine-1-carbonyl)phenyl)-nicotinamide (11f)*. Yield: 77%; melting point: 191-193°C; HPLC purity 97.99%; IR *υ*_max_ /cm^−1^: 3248, 3178 (NH), 1674, 1635 (C=O); ^1^H NMR (DMSO-*d*6, 400 MHz) *δ* ppm: 7.44 (m, 2H), 7.54 (m, 1H), 7.59 (dd, *J* = 7.6, 8.0 Hz, 1H), 7.95 (d, *J* = 8.0 Hz, 2H), 7.99 (d, *J* = 8.0 Hz, 2H), 8.05 (s, 1H), 8.32 (d, *J* = 8.0 Hz, 1H), 8.79 (s, 1H), 8.89 (s, 1H), 9.15 (s, 1H), 10.74 (s, 1H), 12.08 (s, 1H); mass (*m*/*z*): 378.13 (M^+^, 21%), 379.53 (M^+1^, 8%), 345 (100%).

*(7) (E)-N-(4-(2-(4-(Dimethylamino)benzylidene)hydrazine-1-carbonyl)phenyl)-nicotinamide (11g)*. Yield: 82%; melting point: 273-275°C; HPLC purity 97.37%; IR *υ*_max_ /cm^−1^: 3321, 3255 (NH), 1651 (C=O); ^1^H NMR (DMSO-*d*6, 400 MHz) *δ* ppm: 2.99 (s, 6H), 6.76 (d, *J* = 8.4 Hz, 2H), 7.55 (d, *J* = 8.4 Hz, 2H), 7.59 (dd, *J* = 8.0, 7.6 Hz, 1H), 7.92 (d, *J* = 9.2 Hz, 2H), 7.95 (d, *J* = 9.2 Hz, 2H), 8.33 (s, 1H), 8.35 (t, *J* = 6.0 Hz, 1H), 8.79 (s, 1H), 9.15 (s, 1H), 10.72 (s, 1H), 11.54 (s, 1H); ^13^C NMR (DMSO-*d*6) *δ* (ppm): 40.26 (2C), 112.29 (2C), 120.03 (2C), 122.13, 124.04 (2C), 128.89 (2C), 130.84, 136.07 (2C), 142.21, 148.81, 149.24 (2C), 151.95, 152.80, 162.54, 164.87.

*(8) (E)-N-(4-(2-(4-Hydroxybenzylidene)hydrazine-1-carbonyl)phenyl)-nicotinamide (11h)*. Yield: 72%; melting point: 256-259°C; HPLC purity 97.49%; IR *υ*_max_ /cm^−1^: 3340 (OH), 3267, 3182 (NH), 1671, 1643 (C= O); ^1^H NMR (DMSO-*d*6, 400 MHz) *δ* ppm: 6.85 (d, *J* = 8.4 Hz, 2H), 7.57 (d, *J* = 8.4 Hz, 2H), 7.60 (dd, *J* = 8.0, 8.0 Hz, 1H), 7.92 (d, *J* = 8.8 Hz, 2H), 7.95 (d, *J* = 8.8 Hz, 2H), 8.32 (d, *J* = 8.0 Hz, 1H), 8.36 (s, 1H), 8.79 (s, 1H), 9.14 (s, 1H), 9.97 (s, 1H), 10.72 (s, 1H), 11.63 (s, 1H); ^13^C NMR (DMSO-*d*6) *δ* (ppm): 116.19 (2C), 120.03 (2C), 124.05 (2C), 125.83, 128.88 (2C), 130.83, 136.07 (2C), 142.31, 148.28, 149.23 (2C), 152.81, 159.86, 162.72, 164.89.

#### 4.1.4. General Procedure for Synthesis of Compounds 18a-c

A mixture of the potassium salt 15 (0.217 g, 0.001 mol) and the appropriate chloroacetanilides (0.001 mol), namely, 2-chloro-*N*-(4-chlorophenyl)acetamide 17a, 2-chloro-*N*-phenylacetamide 17b, or *N*-benzyl-2-chloroacetamide 17c (0.001 mol) in dry DMF (25 ml) with a catalytic amount of potassium iodide was heated over a water bath for 4 h. Then, the reaction mixture was cooled, poured into ice water (50 ml), and stirred well for 0.5 h. The residue was filtered off, and the crude product was crystallized from ethanol.

*(1) N-(4-Chlorophenyl)-2-((5-(pyridin-3-yl)-1,3,4-oxadiazol-2-yl)thio)acetamide (18a)*. Yield: 72%; melting point: 225-228°C; IR *υ*_max_ /cm^−1^: 3271 (NH), 1635 (C=O); ^1^H NMR (DMSO-*d*6, 400 MHz) *δ* ppm: 3.85 (s, 2H), 7.41 (d, *J* = 8.8 Hz, 2H), 7.61 (t, *J* = 8.0 Hz, 1H), 7.64 (d, *J* = 8.8 Hz, 2H), 8.24 (d, *J* = 8.0 Hz, 1H), 8.74 (dd, *J* = 4.8, 4.8 Hz, 1H), 9.07 (s, 1H), 10.37 (s, 1H); mass (*m*/*z*): 346.12 (M^+^, 31%), 347.34 (M^+1^, 11%), 312.51 (100%).

*(2) N-Phenyl-2-((5-(pyridin-3-yl)-1,3,4-oxadiazol-2-yl)thio)acetamide (18b)*. Yield: 72%; melting point: 232-234°C; IR *υ*_max_ /cm^−1^: 3209 (NH), 1635 (C=O); ^1^H NMR (DMSO-*d*6, 400 MHz) *δ* ppm: 3.58 (s, 2H), 7.04 (t, *J* = 7.6 Hz, 1H), 7.38 (t, *J* = 7.6 Hz, 2H), 7.61 (t, *J* = 7.6 Hz, 1H), 7.63 (d, *J* = 7.6 Hz, 2H), 8.25 (d, *J* = 8.0 Hz, 1H), 8.75 (dd, *J* = 4.8, 4.8 Hz, 1H), 9.08 (s, 1H), 10.90 (s, 1H); mass (*m*/*z*): 312.19 (M^+^, 100%).

*(3) N-Benzyl-2-((5-(pyridin-3-yl)-1,3,4-oxadiazol-2-yl)thio)acetamide (18c)*. Yield: 72%; melting point: 167-169°C; HPLC purity 96.89%; IR *υ*_max_ /cm^−1^: 3205 (NH), 1647 (C=O); ^1^H NMR (DMSO-*d*6, 400 MHz) *δ* ppm: 4.14 (s, 2H), 4.93 (s, 2H), 7.28 (t, *J* = 6.8 Hz, 1H), 7.35 (t, *J* = 7.2 Hz, 2H), 7.41 (t, *J* = 7.2 Hz, 2H), 7.51 (t, *J* = 8.0 Hz, 1H), 8.18 (d, *J* = 6.8 Hz, 1H), 8.70 (s, 1H), 9.00 (s, 1H); mass (*m*/*z*): 326.71 (M^+^, 29%), 147 (100%).

### 4.2. Biological Evaluation

#### 4.2.1. In Vitro Antiproliferative Activity against MCF-7, HepG-2, and HCT-116

The antiproliferative activity of the synthesized compounds was evaluated using MTT protocol as described [50–52] (Supplementary data (available here)).

#### 4.2.2. In Vitro VEGFR-2 Enzyme Assay Inhibition

The most potent cytotoxic compounds, 5, 7a, 7b, 7l, 10, 11c, 11d, 11f, 11g, 18a, and 18b, were further assessed to determine their inhibitory activities against the VEGFR-2 enzyme according to the protocol described in the Supplementary data.

#### 4.2.3. Apoptotic Marker Analysis

*(1) Effects on the Levels of Active Caspase-3*. Measurement of caspase-3 levels was evaluated for the most active members according to the protocol described by Andersson et al. [53] (Supplementary data).

*(2) Effects on Bcl-2 Family Proteins*. Bax and Bcl-2 cellular levels were analyzed for the most potent cytotoxic compounds in HepG-2 cells following the reported protocol [54] (Supplementary data).

*(3) Cell Cycle Analysis*. According to the method described by Léonce et al., the flow cytometric analysis for compound 7a was performed [55, 56] (Supplementary data).

*(4) Apoptotic Cell Subpopulation Determination*. The annexin-V-FITC assay for compound 7a on HepG-2 cells was performed according to the reported procedure [57–59] (Supplementary data).

#### 4.2.4. Estimation of Human Tumor Necrosis Factor-Alpha (TNF-*α*) in HepG-2 Supernatant

The levels of TNF-*α* in cell culture supernatants were estimated by the ELISA technique according to the reported procedure [60, 61].

#### 4.2.5. Vascular Smooth Muscle Cell Culture

VSMCs were isolated and cultured following the reported method [62] (Supplementary data).

### 4.3. Docking Study

Discovery Studio 2.5 software was used to perform docking and visualization according to the described protocol [63–66] (Supplementary data).

## Figures and Tables

**Table 1:** IC_50_ values of the MTT assayed compounds against the tested cell lines.

Comp.	Structure	MCF-7	HepG-2	HCT-116	WI-38
5	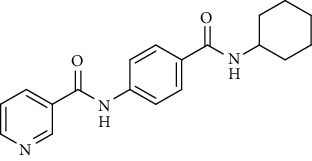	4.66	3.29	1.60	61.39
6	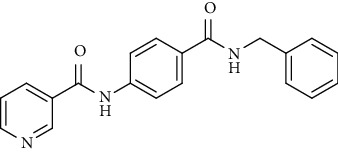	18.32	15.45	16.40	117.36
7a	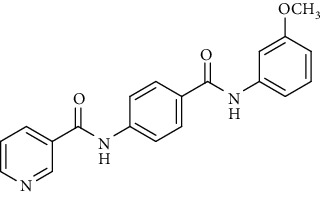	1.37	1.05	1.46	60.8
7b	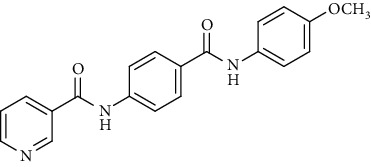	1.25	2.12	2.54	82.17
7c	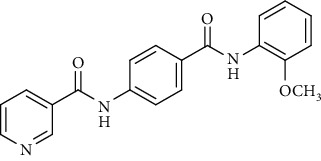	11.79	19.41	15.34	136.51
7d	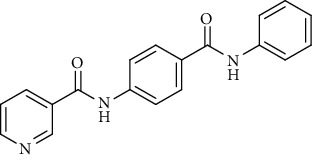	23.11	29.14	31.47	143.82
7e	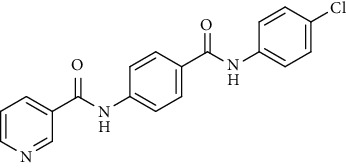	13.38	22.41	17.80	142.63
7f	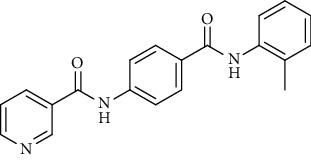	20.46	31.45	18.30	157.19
7 g	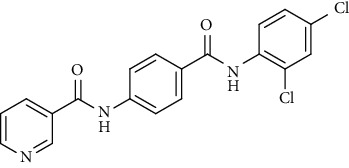	42.10	26.55	31.70	131.54
7 h	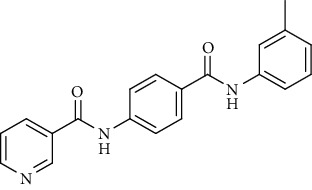	19.20	14.90	22.70	95.14
7i	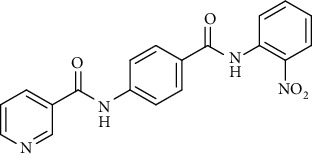	16.15	15.22	18.40	103.26
7j	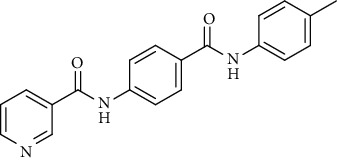	16.80	14.08	15.10	84.92
7 k	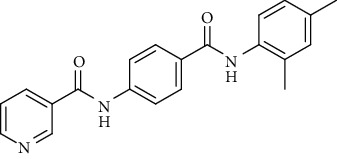	17.14	20.05	18.70	75.31
7 l	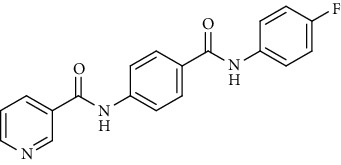	3.50	2.64	2.75	68.21
10	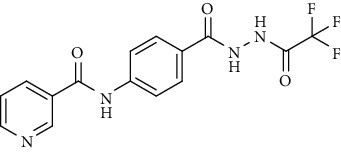	3.55	3.16	5.49	78.42
11a	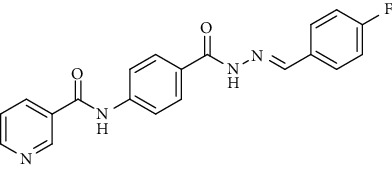	12.31	15.47	17.16	85.13
11b	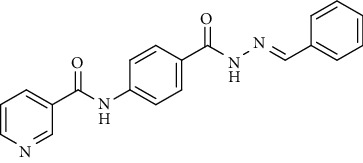	15.54	17.62	13.44	72.28
11c	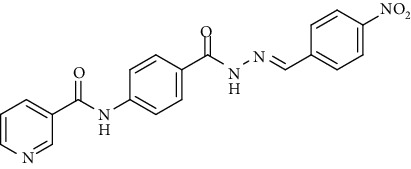	6.29	7.31	5.72	52.31
11d	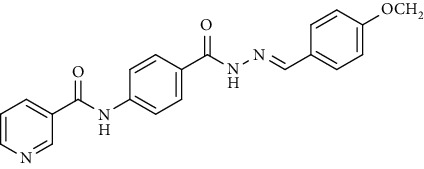	4.15	2.23	1.94	50.28
11e	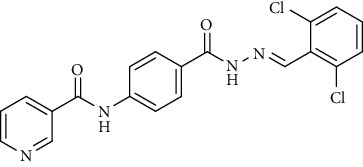	18.22	23.91	14.52	80.06
11f	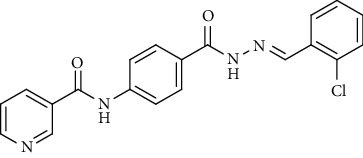	6.15	7.05	3.47	62.37
11 g	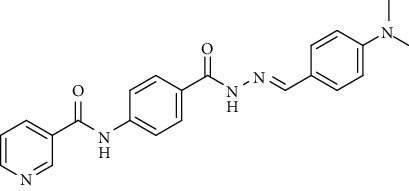	5.73	6.42	4.80	70.14
11 h	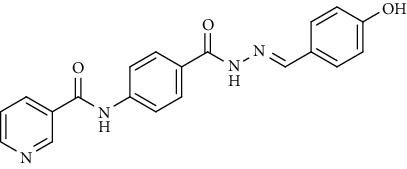	20.17	31.12	34.9	103.22
18a	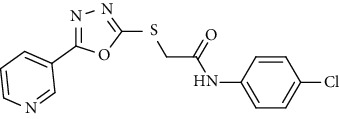	5.81	8.29	5.13	123.42
18b	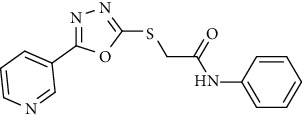	2.67	6.11	4.19	64.35
18c	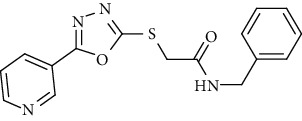	19.37	8.59	21.72	92.11
Sorafenib	—	4.32	3.76	5.28	—

**Table 2:** IC_50_ values of the tested compounds against VEGFR-2.

Comp.	IC_50_
5	2.46
7a	2.17
7b	3.45
7l	3.84
10	7.68
11c	8.09
11d	2.74
11f	4.17
11g	6.12
18a	5.23
18b	6.57
Sorafenib	2.36

**Table 3:** Caspase 3 concentrations, BAX and Bcl-2 expression levels, and TNF-*α* percent inhibition in HepG-2 cells treated with the tested compounds.

Comp.	Caspase 3		BAX		Bcl-2		TNF-*α* % inhibition
	Conc. (Pg/ml)	Fold	Conc. (Pg/ml)	Fold	Conc. (Pg/ml)	Fold	
11c	271.41	5.28	133.2	6.17	2.81	0.52	31%
11d	391.23	7.61	237.9	11.02	1.72	0.32	81%
11f	292.11	5.68	142.5	6.60	2.25	0.42	60%
11g	324.36	6.31	160.2	7.42	2.55	0.48	47%
10	334.57	6.51	231.6	10.73	2.72	0.51	40%
18b	310.75	6.04	154.4	7.15	2.51	0.47	54%
18a	267.64	5.20	113.5	5.26	2.63	0.49	45%
7a	401.15	7.80	261.4	12.11	1.25	0.23	87%
7b	397.61	7.73	257.5	11.93	1.92	0.36	72%
5	345.5	6.72	232.7	10.78	1.35	0.25	85%
7l	340.42	6.62	226.9	10.51	2.11	0.39	74%
Control	51.38	1.00	21.57	1.00	5.31	1.00	86%

## Data Availability

Data can be found in the supplementary information files.
