# Supplementary material for: Inhibition of Vascular Smooth Muscle and Cancer Cell Proliferation by New VEGFR Inhibitors and Their Immunomodulator Effect: Design, Synthesis, and Biological Evaluation
Source: Oxid Med Cell Longev. 2021 Oct 28;2021:8321400. doi: 10.1155/2021/8321400 (PMC8568530; doi:10.1155/2021/8321400)
Supplement: Supplementary Materials — Supplementary data related to this manuscript is found in a separate file. [file 8321400.f1.pdf]

Mohamed khalifa-R14-carbon-ES

5

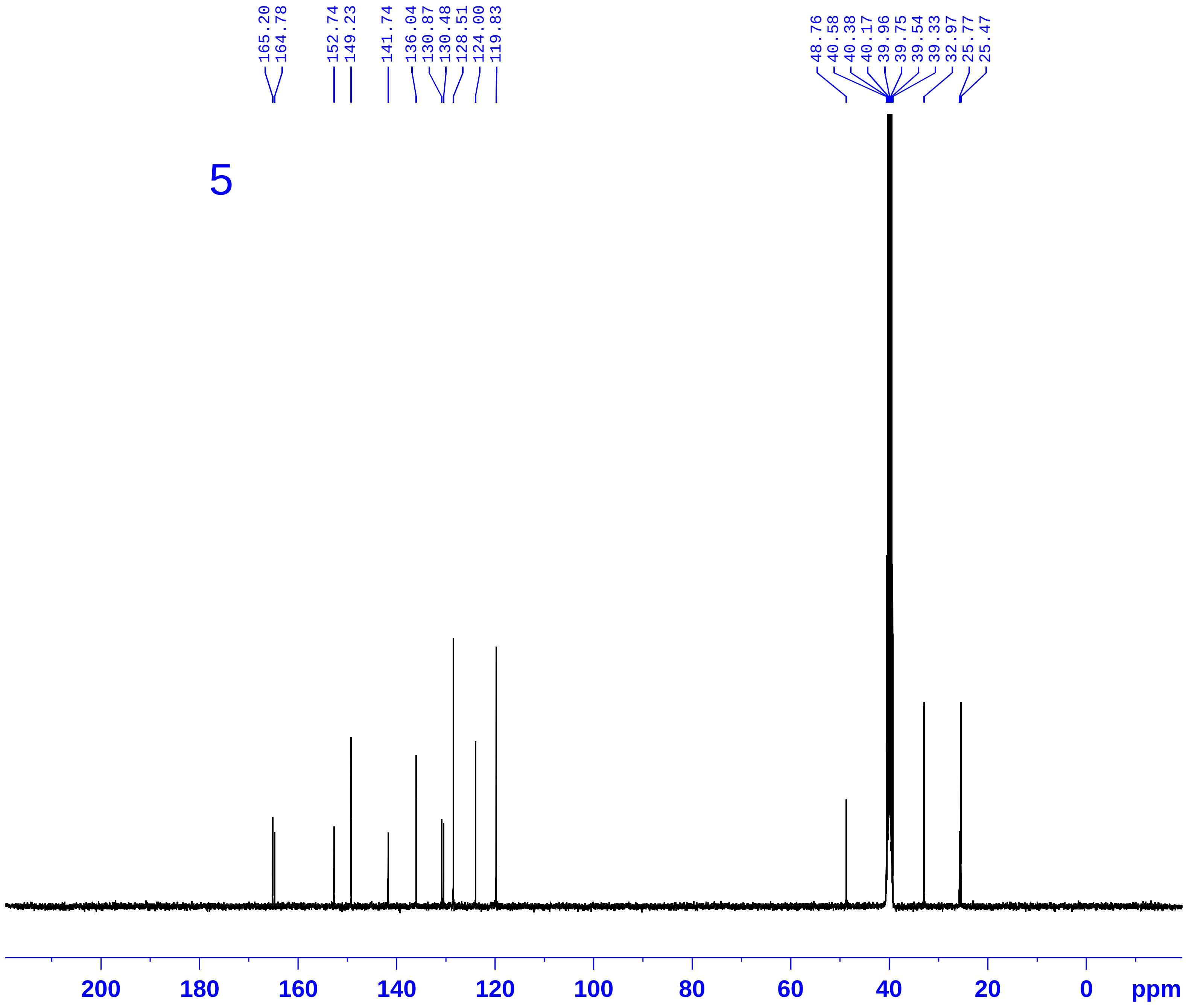

Faculty of Pharmacy

```
Current Data Parameters
NAME       Mohamed khalifa-R14-carbon-ES
EXPNO                     10
PROCNO                     1

F2 - Acquisition Parameters
Date_               20201202
Time                    7.01 h
INSTRUM                spect
PROBHD     Z108618_0945 (
PULPROG              zgpg30
TD                     65536
SOLVENT                 DMSO
NS                      2100
DS                         4
SWH                24038.461 Hz
FIDRES              0.733596 Hz
AQ                 1.3631488 sec
RG                    197.77
DW                    20.800 usec
DE                      6.50 usec
TE                     293.5 K
D1                2.00000000 sec
D11               0.03000000 sec
TD0                        1
SFO1             100.6404331 MHz
NUC1                     13C
P1                     10.00 usec
PLW1             47.00000000 W
SFO2             400.2016008 MHz
NUC2                      1H
CPDPRG[2             waltz16
PCPD2                  90.00 usec
PLW2             13.00000000 W
PLW12             0.29249999 W
PLW13             0.14713000 W

F2 - Processing parameters
SI                     32768
SF               100.6303700 MHz
WDW                       EM
SSB        0
LB                      1.00 Hz
GB         0
PC                      1.40
```

Mohamed khalifa-R14-carbon-ES

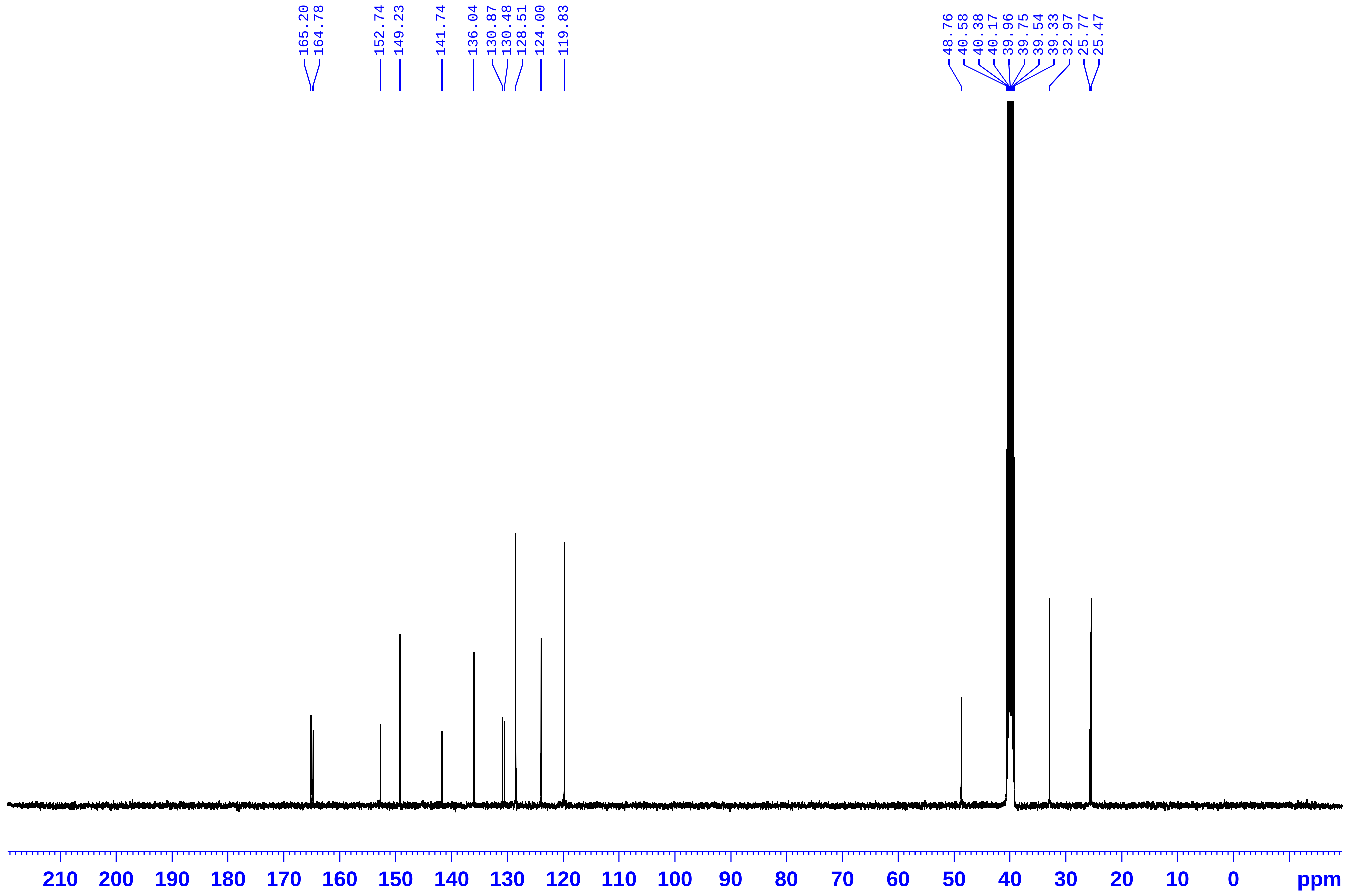

Mohamed khalifa-R14-carbon-ES

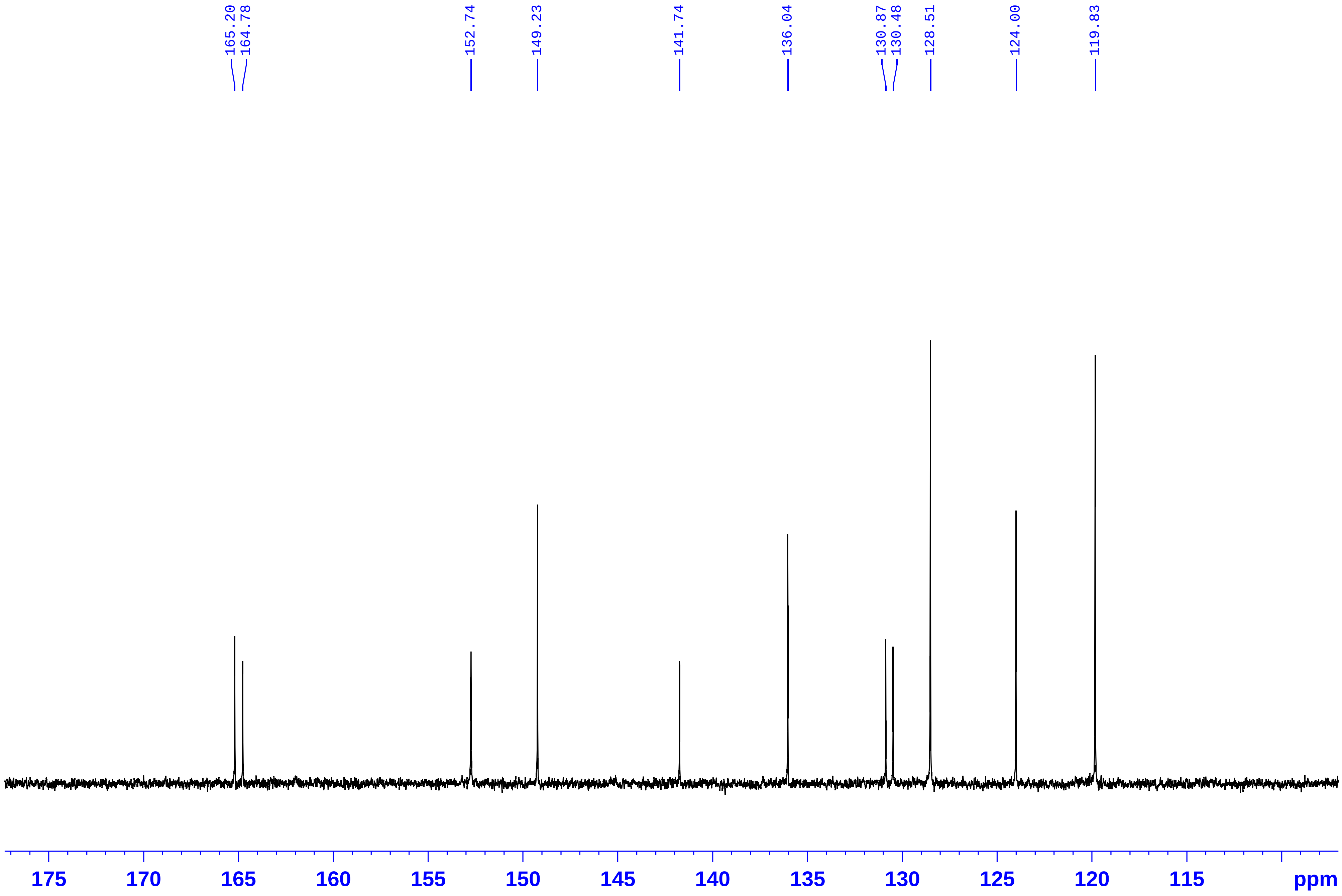

Mohamed khalifa-R14-carbon-ES

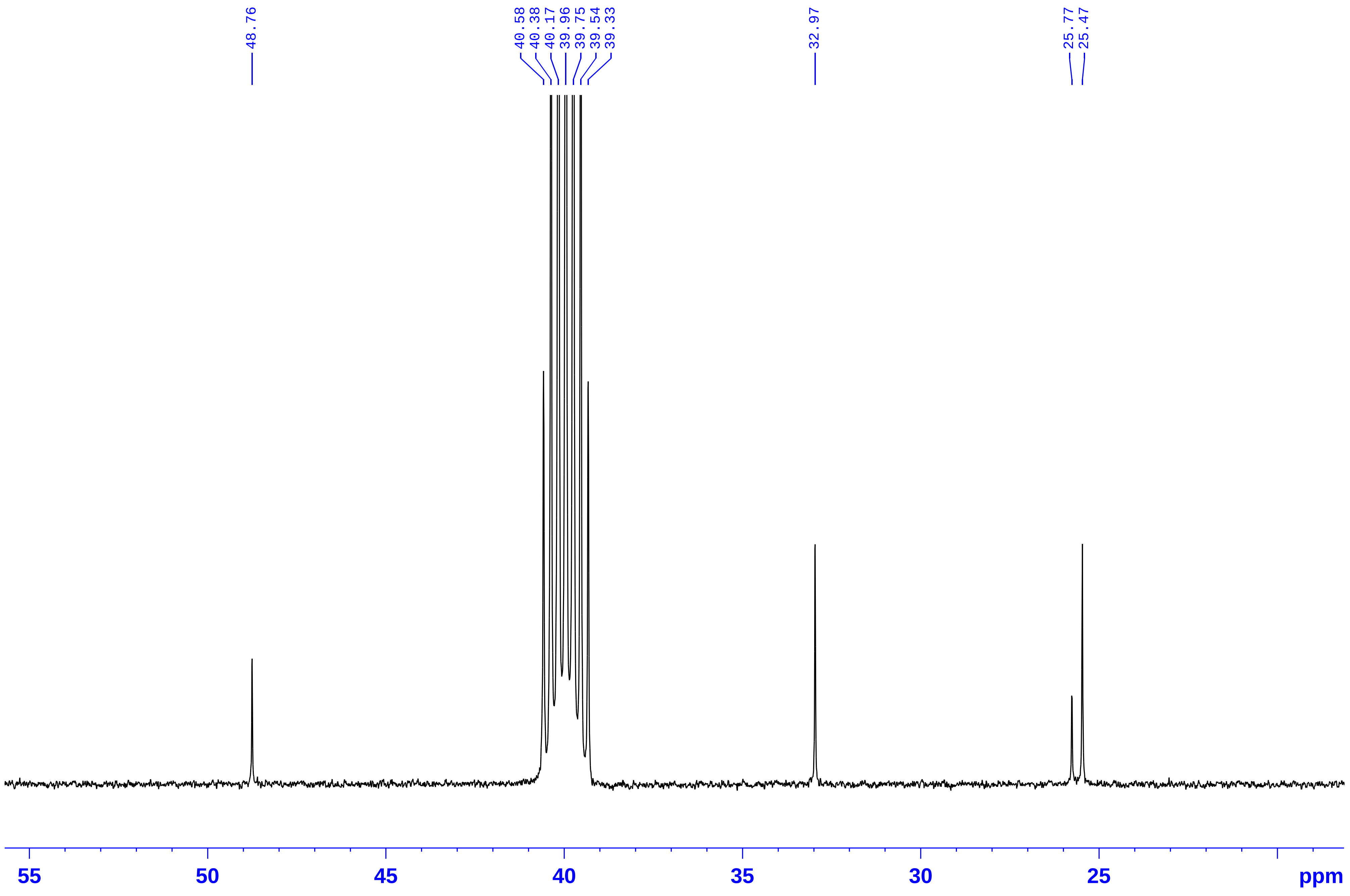

Mohamed khalifa-R5-carbon-ES

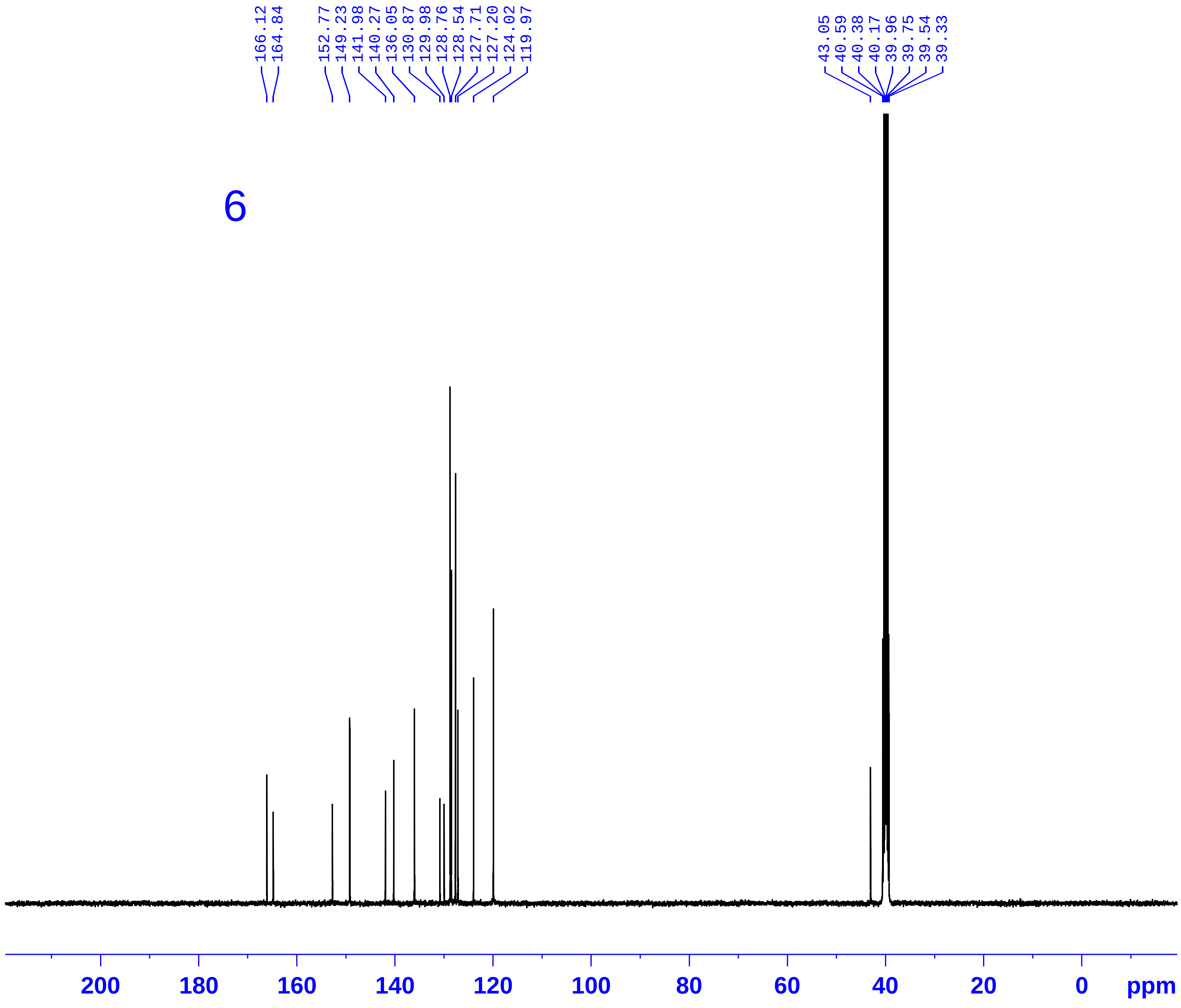

Faculty of Pharmacy

Current Data Parameters
NAME Mohamed khalifa-R5-carbon-ES
EXPNO 10
PROCNO 1

F2 - Acquisition Parameters
Date_ 20201202
Time 9.05 h
INSTRUM spect
PROBHD Z108618_0945 (
PULPROG zgpg30
TD 65536
SOLVENT DMSO
NS 2100
DS 4
SWH 24038.461 Hz
FIDRES 0.733596 Hz
AQ 1.3631488 sec
RG 197.77
DW 20.800 usec
DE 6.50 usec
TE 293.5 K
D1 2.00000000 sec
D11 0.03000000 sec
TD0 1
SFO1 100.6404331 MHz
NUC1 13C
P1 10.00 usec
PLW1 47.00000000 W
SFO2 400.2016008 MHz
NUC2 1H
CPDPRG[2 waltz16
PCPD2 90.00 usec
PLW2 13.00000000 W
PLW12 0.29249999 W
PLW13 0.14713000 W

F2 - Processing parameters
SI 32768
SF 100.6303700 MHz
WDW EM
SSB 0
LB 1.00 Hz
GB 0
PC 1.40

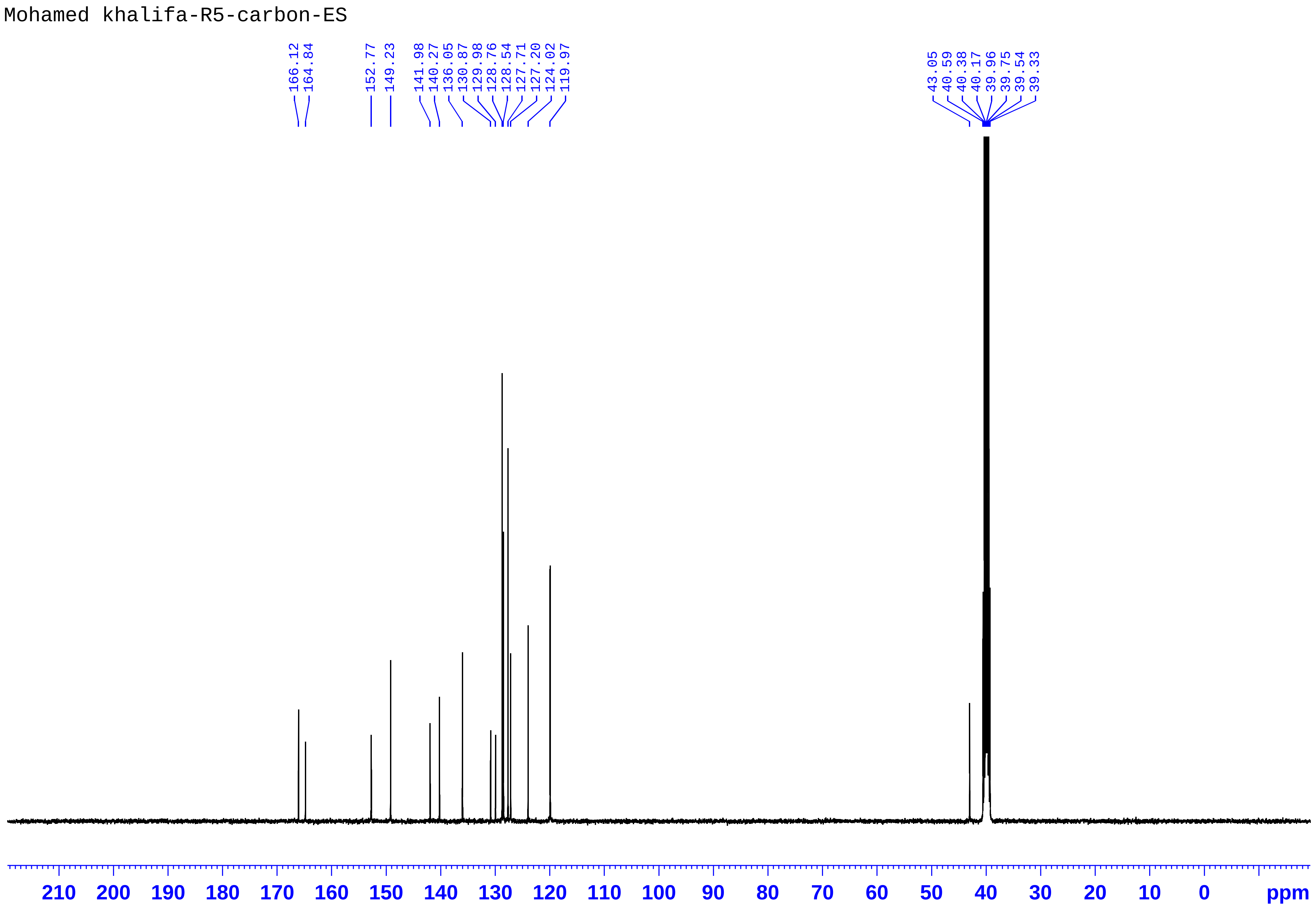
Mohamed khalifa-R5-carbon-ES
166.12
164.84
152.77
149.23
141.98
140.27
136.05
130.87
129.98
128.76
128.54
127.71
127.20
124.02
119.97
43.05
40.59
40.38
40.17
39.96
39.75
39.54
39.33
210
200
190
180
170
160
150
140
130
120
110
100
90
80
70
60
50
40
30
20
10
0
ppm

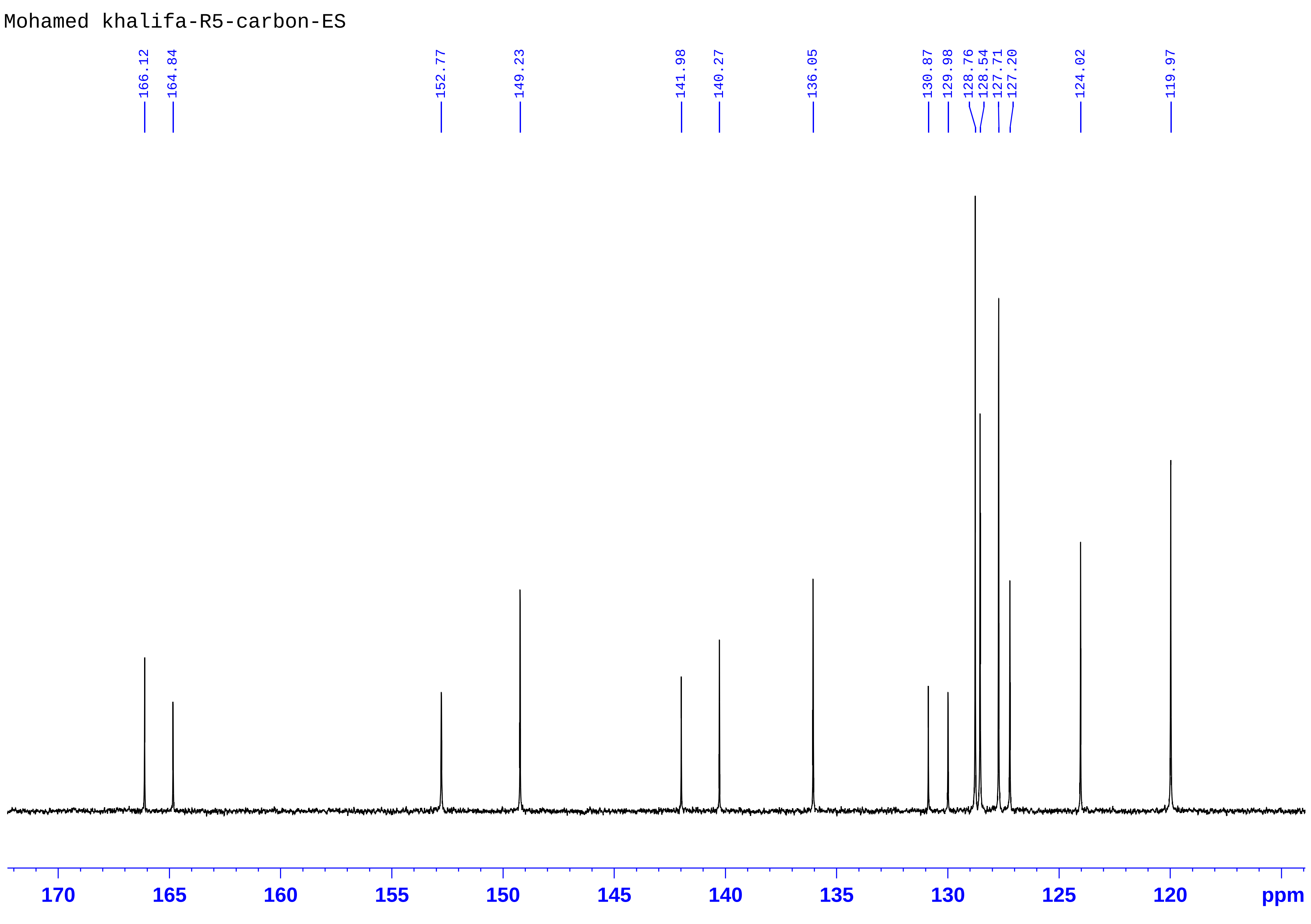
Mohamed khalifa-R5-carbon-ES
166.12
164.84
152.77
149.23
141.98
140.27
136.05
130.87
129.98
128.76
128.54
127.71
127.20
124.02
119.97
170
165
160
155
150
145
140
135
130
125
120
ppm

Mohamed khalifa-R5-carbon-ES

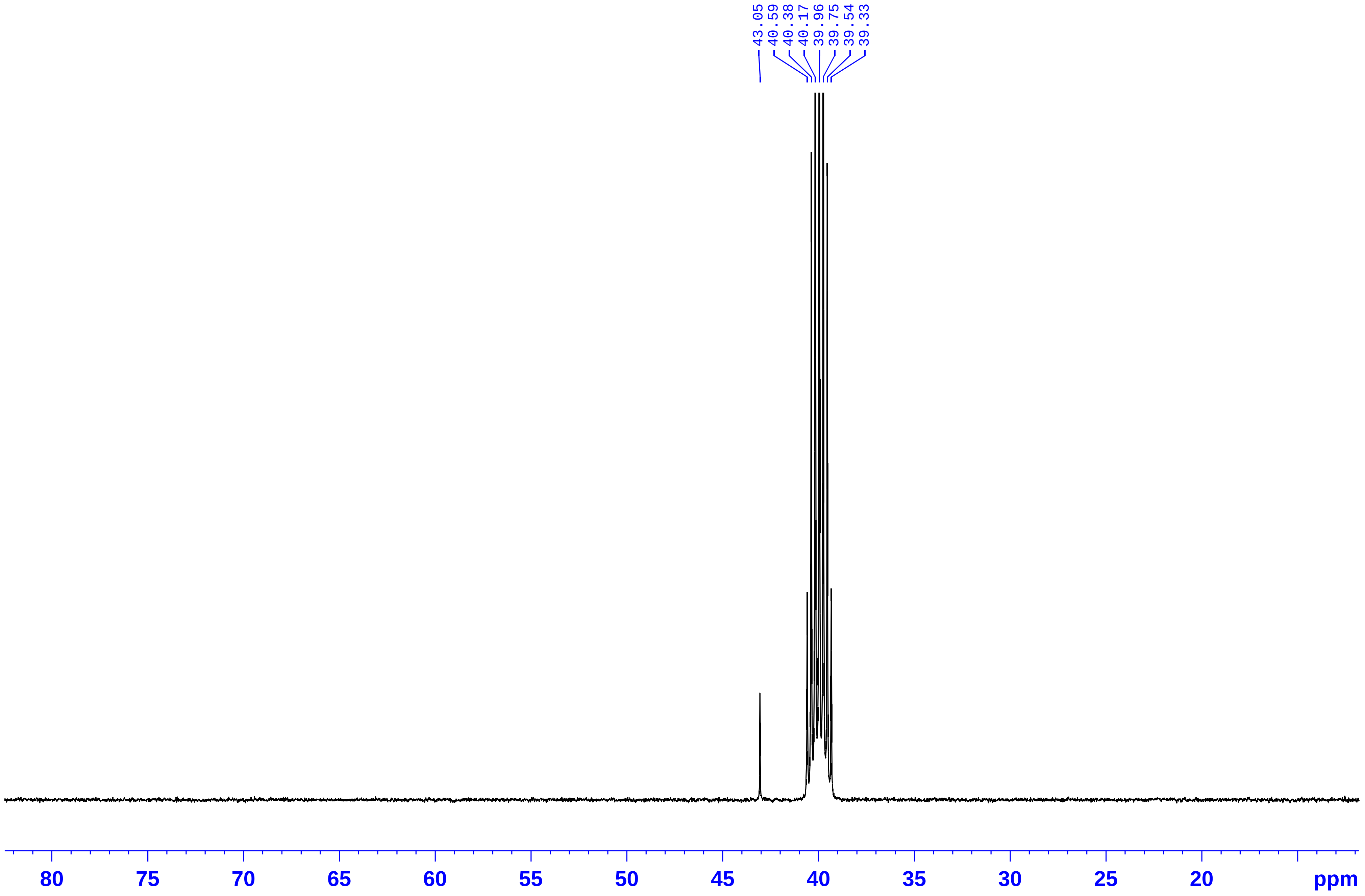

Mohamed khalifa-R1-carbon

7a

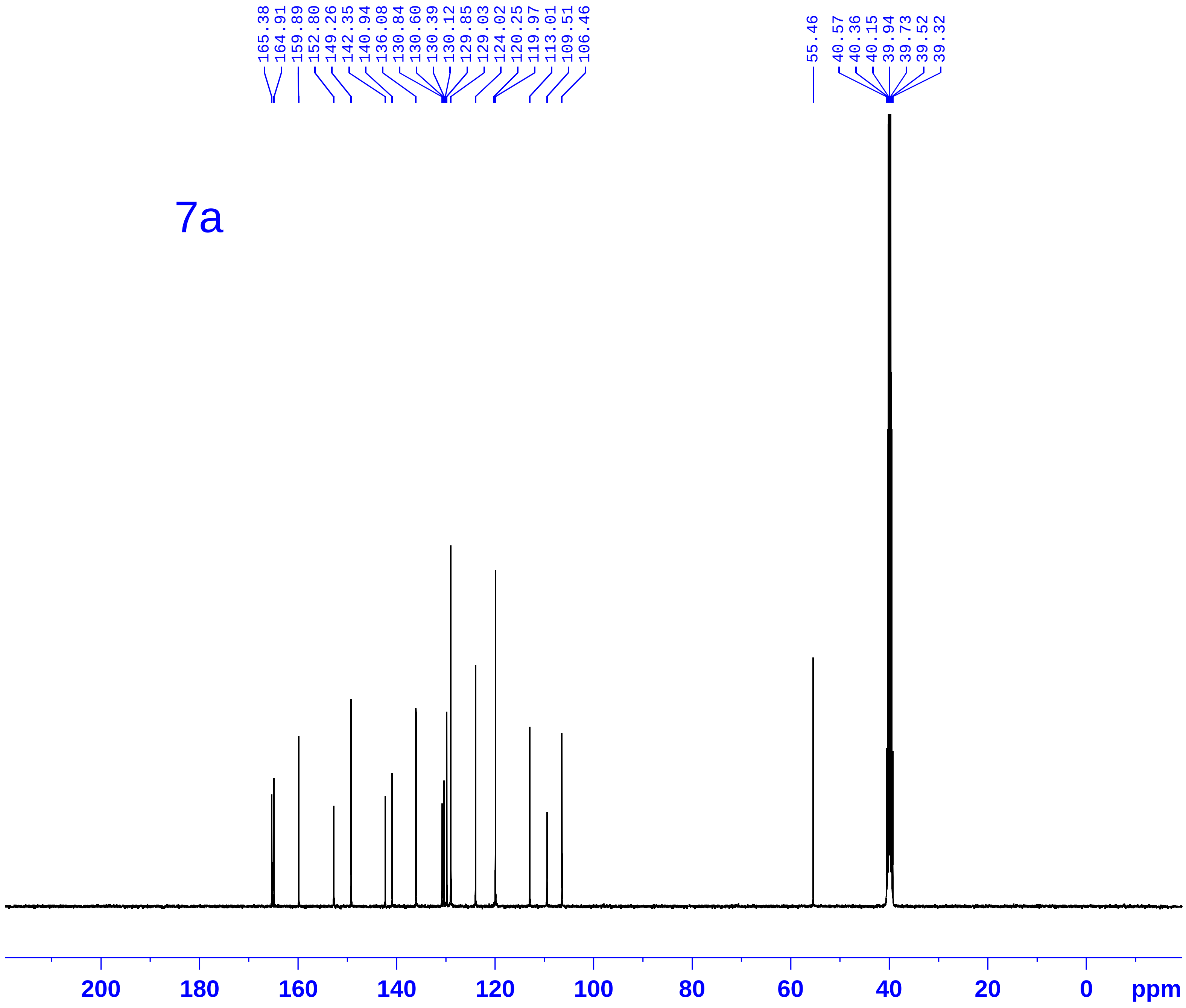

Current Data Parameters
NAME Mohamed khalifa-R1-carbon-ES
EXPNO 10
PROCNO 1

F2 - Acquisition Parameters
Date_ 20201201
Time 20.17 h
INSTRUM spect
PROBHD Z108618_0945 (
PULPROG zgpg30
TD 65536
SOLVENT DMSO
NS 2200
DS 4
SWH 24038.461 Hz
FIDRES 0.733596 Hz
AQ 1.3631488 sec
RG 197.77
DW 20.800 usec
DE 6.50 usec
TE 293.8 K
D1 2.00000000 sec
D11 0.03000000 sec
TD0 1
SFO1 100.6404331 MHz
NUC1 13C
P1 10.00 usec
PLW1 47.00000000 W
SFO2 400.2016008 MHz
NUC2 1H
CPDPRG[2 waltz16
PCPD2 90.00 usec
PLW2 13.00000000 W
PLW12 0.29249999 W
PLW13 0.14713000 W

F2 - Processing parameters
SI 32768
SF 100.6303700 MHz
WDW EM
SSB 0
LB 1.00 Hz
GB 0
PC 1.40

Mohamed khalifa-R1-carbon

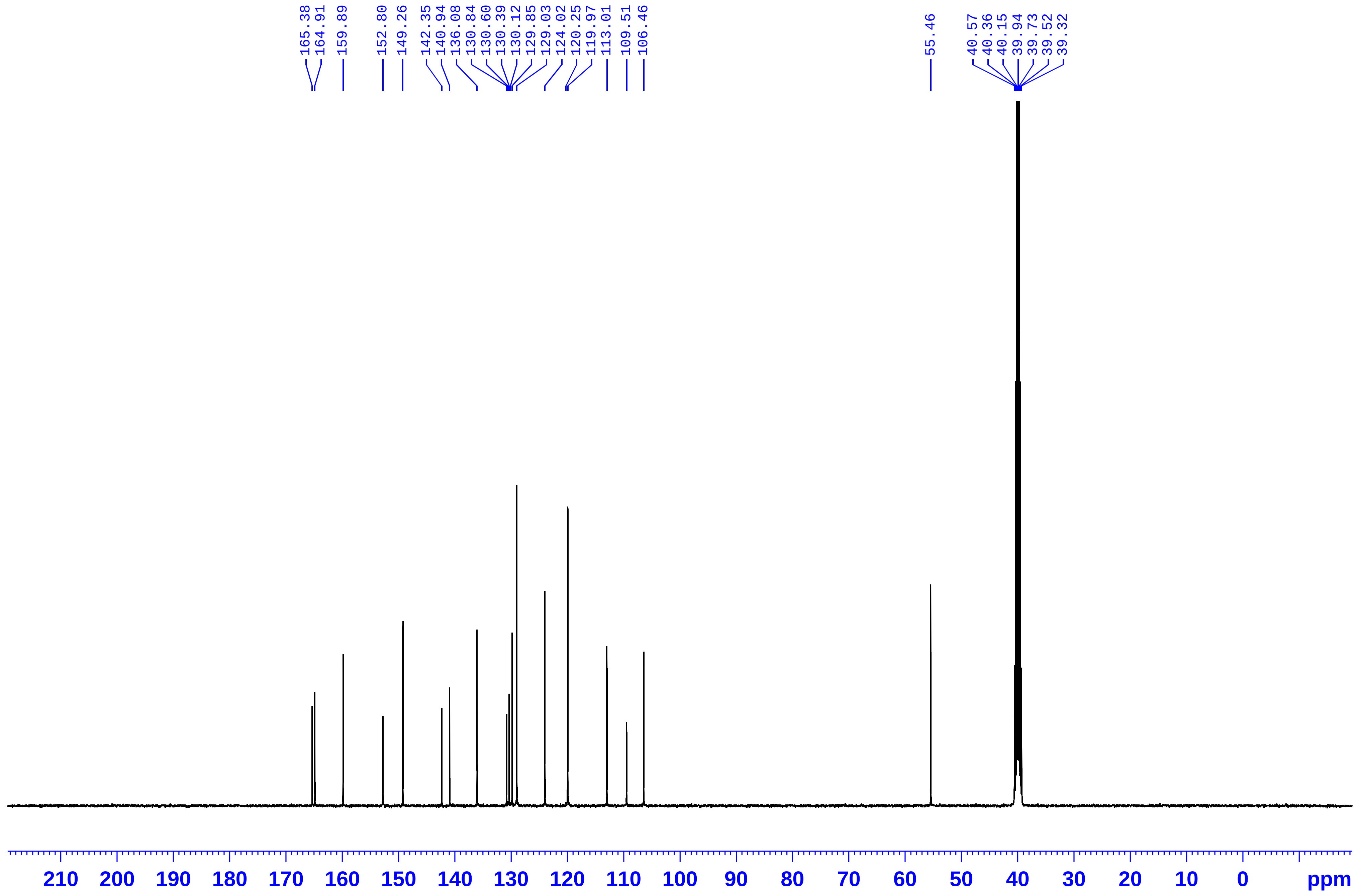

Mohamed khalifa-R1-carbon

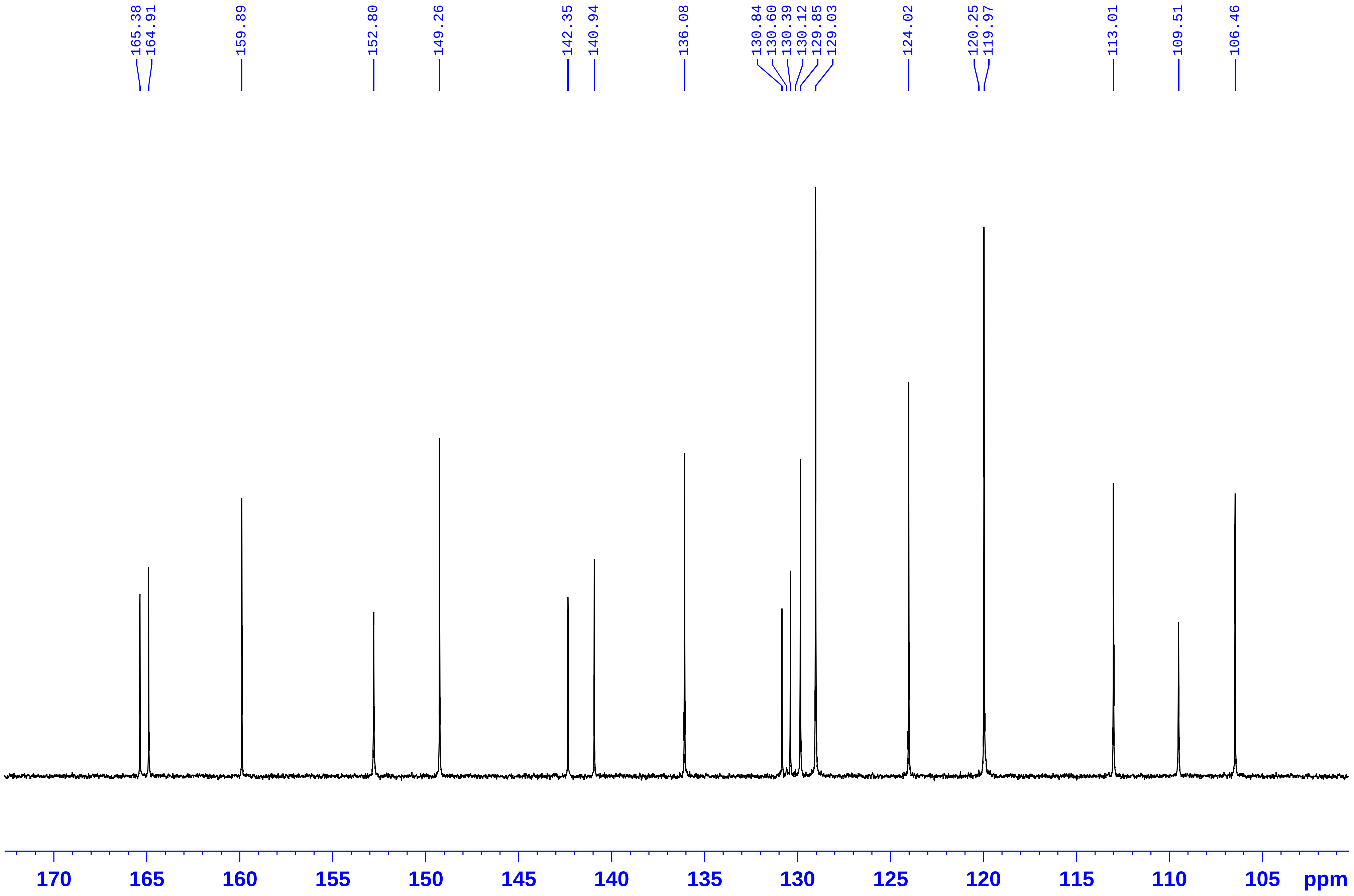

Mohamed khalifa-R3-carbon-ES

7c

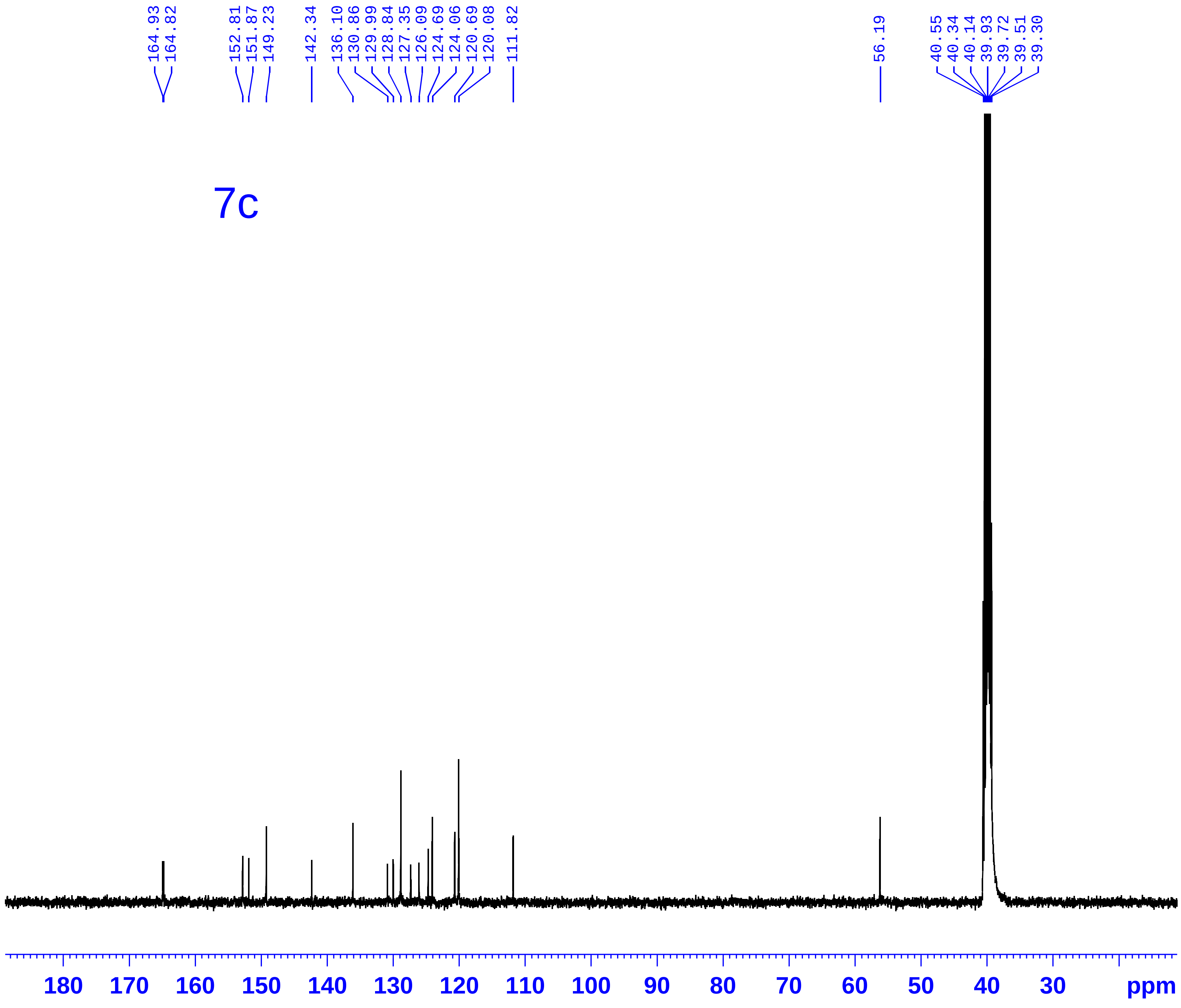

Faculty of Pharmacy

Current Data Parameters
NAME Mohamed khalifa-R3-carbon-ES
EXPNO 10
PROCNO 1

F2 - Acquisition Parameters
Date_ 20201202
Time 11.08 h
INSTRUM spect
PROBHD Z108618_0945 (
PULPROG zgpg30
TD 65536
SOLVENT DMSO
NS 2100
DS 4
SWH 24038.461 Hz
FIDRES 0.733596 Hz
AQ 1.3631488 sec
RG 197.77
DW 20.800 usec
DE 6.50 usec
TE 294.6 K
D1 2.00000000 sec
D11 0.03000000 sec
TD0 1
SFO1 100.6404331 MHz
NUC1 13C
P1 10.00 usec
PLW1 47.00000000 W
SFO2 400.2016008 MHz
NUC2 1H
CPDPRG[2 waltz16
PCPD2 90.00 usec
PLW2 13.00000000 W
PLW12 0.29249999 W
PLW13 0.14713000 W

F2 - Processing parameters
SI 32768
SF 100.6303700 MHz
WDW EM
SSB 0
LB 1.00 Hz
GB 0
PC 1.40

Mohamed khalifa-R3-carbon-ES

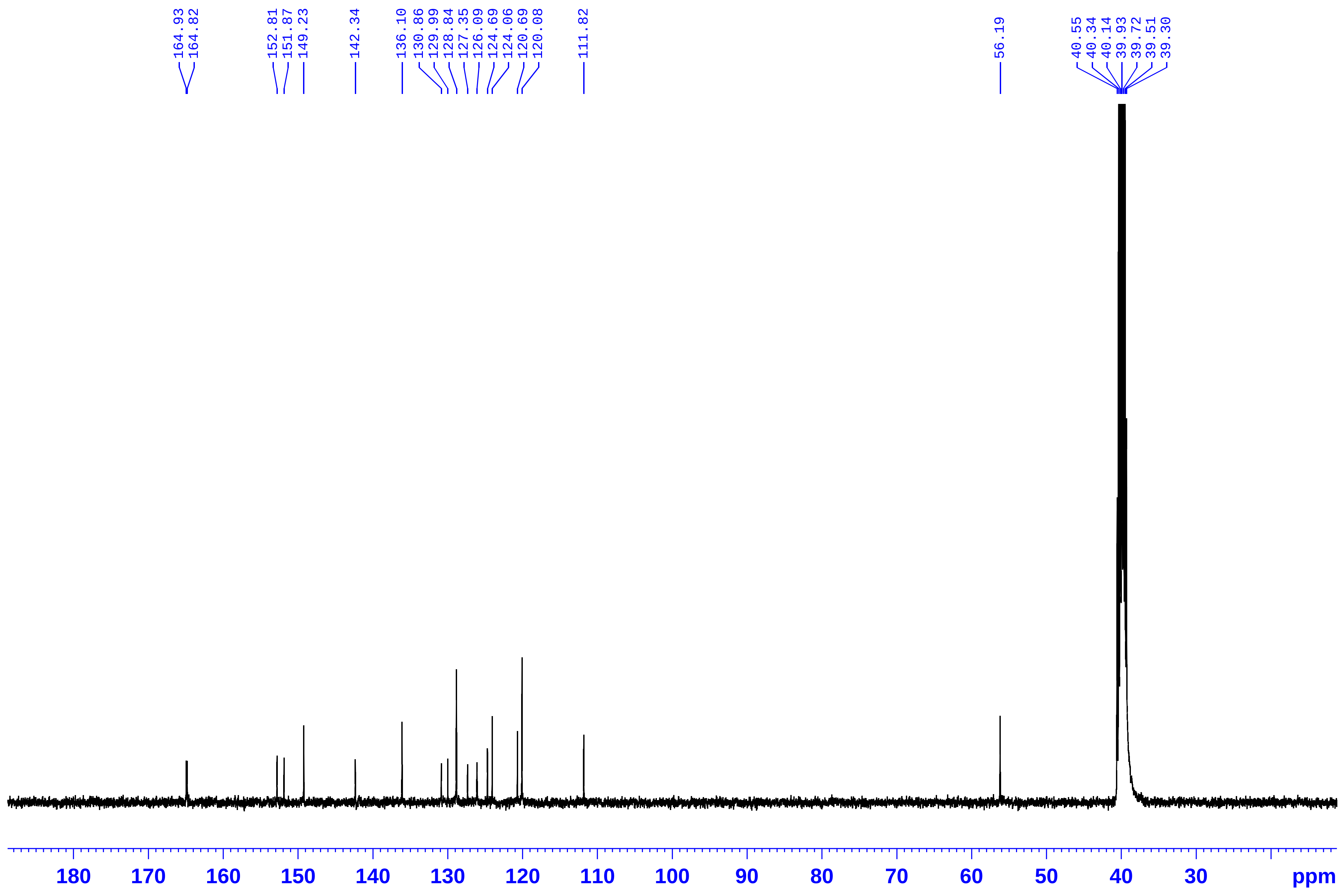

Mohamed khalifa-R3-carbon-ES

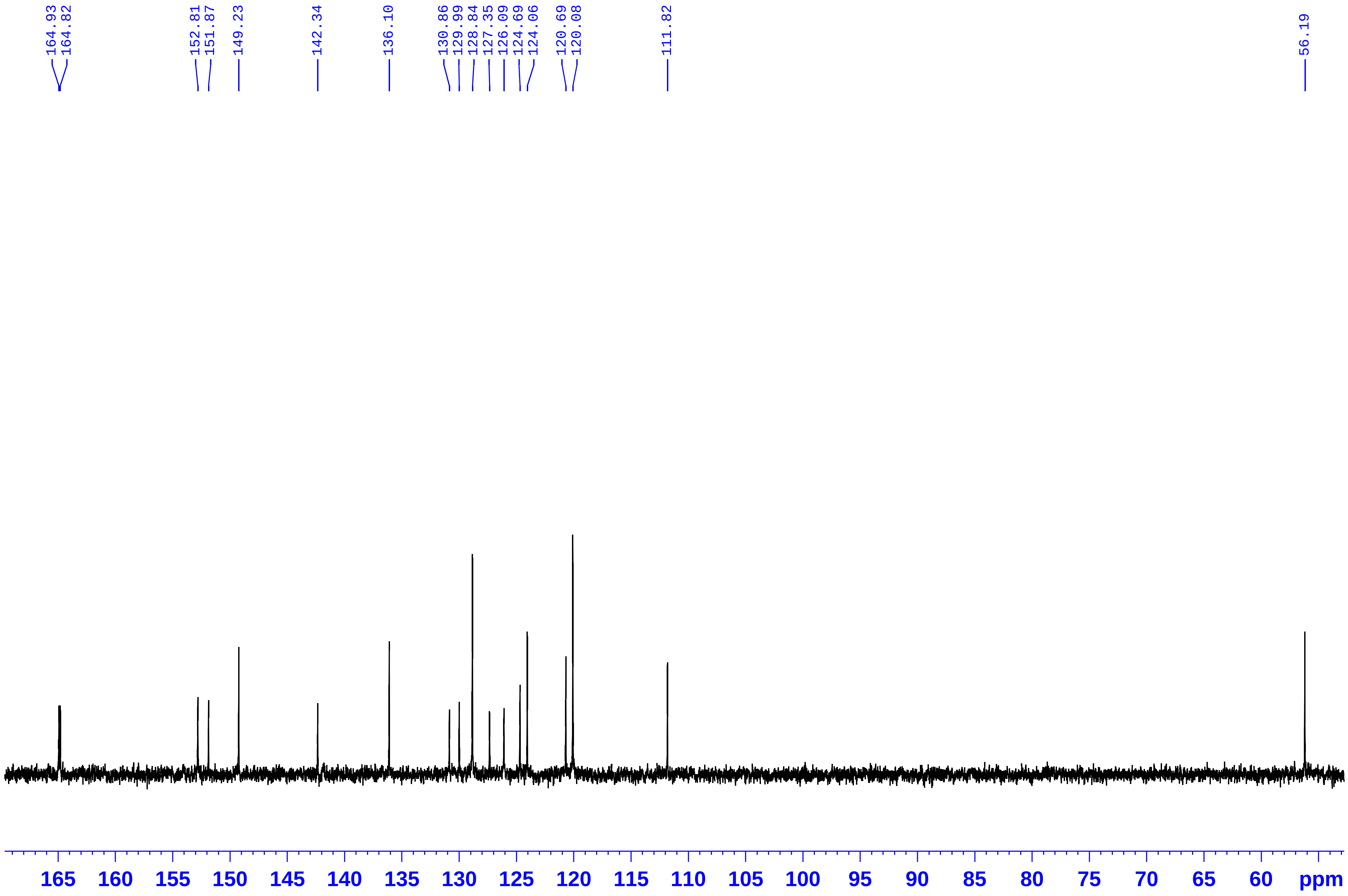

Mohamed khalifa-R3-carbon-ES

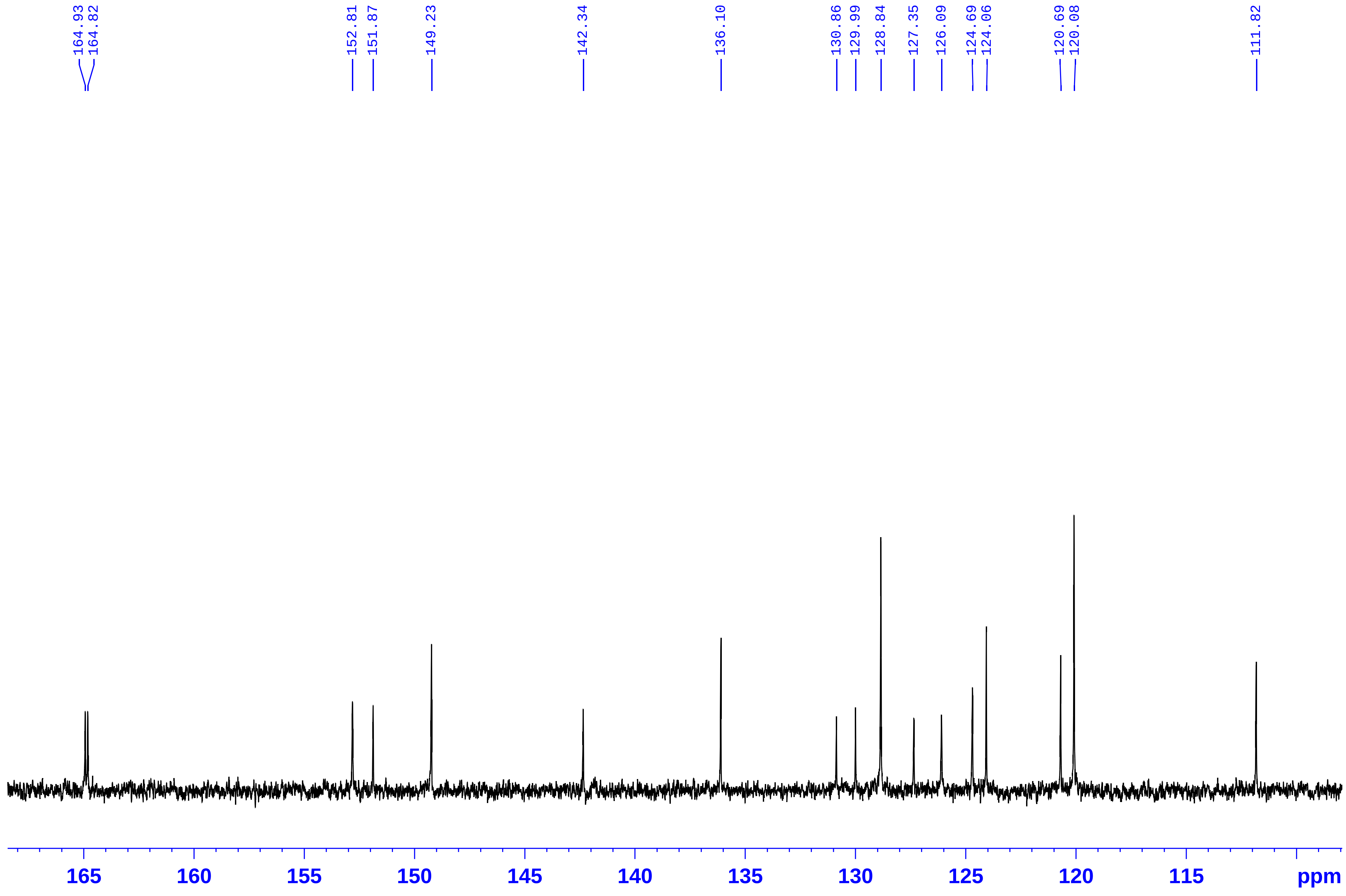

Mohamed khalifa-R8-carbon-ES

7f

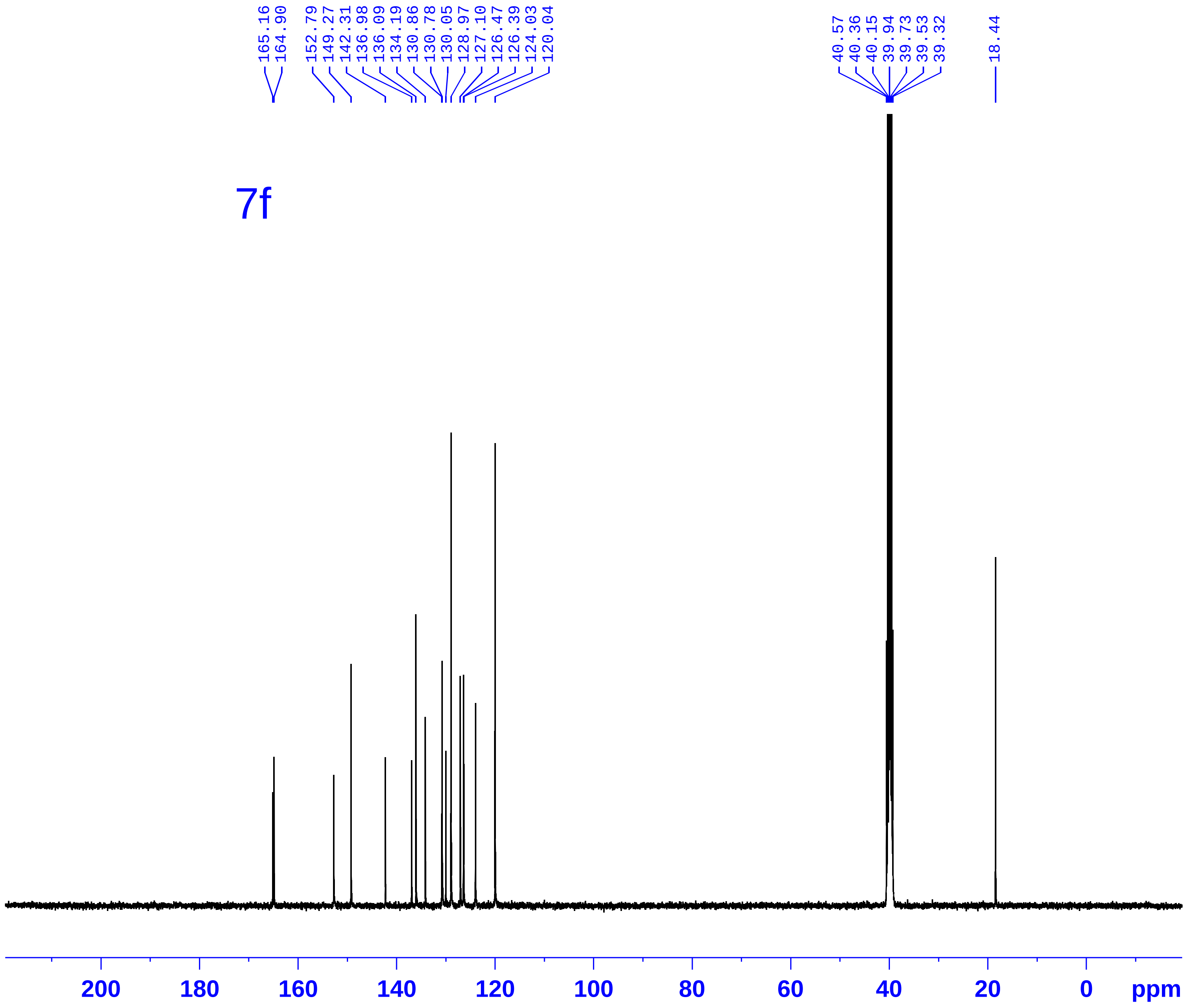

Faculty of Pharmacy

```
Current Data Parameters
NAME      Mohamed khalifa-R8-carbon-ES
EXPNO                     10
PROCNO                     1

F2 - Acquisition Parameters
Date_               20201202
Time                    0.45 h
INSTRUM                spect
PROBHD      Z108618_0945 (
PULPROG              zgpg30
TD                     65536
SOLVENT                 DMSO
NS                      2200
DS                         4
SWH                24038.461 Hz
FIDRES              0.733596 Hz
AQ                 1.3631488 sec
RG                    197.77
DW                    20.800 usec
DE                      6.50 usec
TE                     293.6 K
D1                2.00000000 sec
D11               0.03000000 sec
TD0                        1
SFO1             100.6404331 MHz
NUC1                     13C
P1                     10.00 usec
PLW1             47.00000000 W
SFO2             400.2016008 MHz
NUC2                      1H
CPDPRG[2             waltz16
PCPD2                  90.00 usec
PLW2             13.00000000 W
PLW12             0.29249999 W
PLW13             0.14713000 W

F2 - Processing parameters
SI                     32768
SF               100.6303700 MHz
WDW                       EM
SSB       0
LB                      1.00 Hz
GB        0
PC                      1.40
```

Mohamed khalifa-R8-carbon-ES

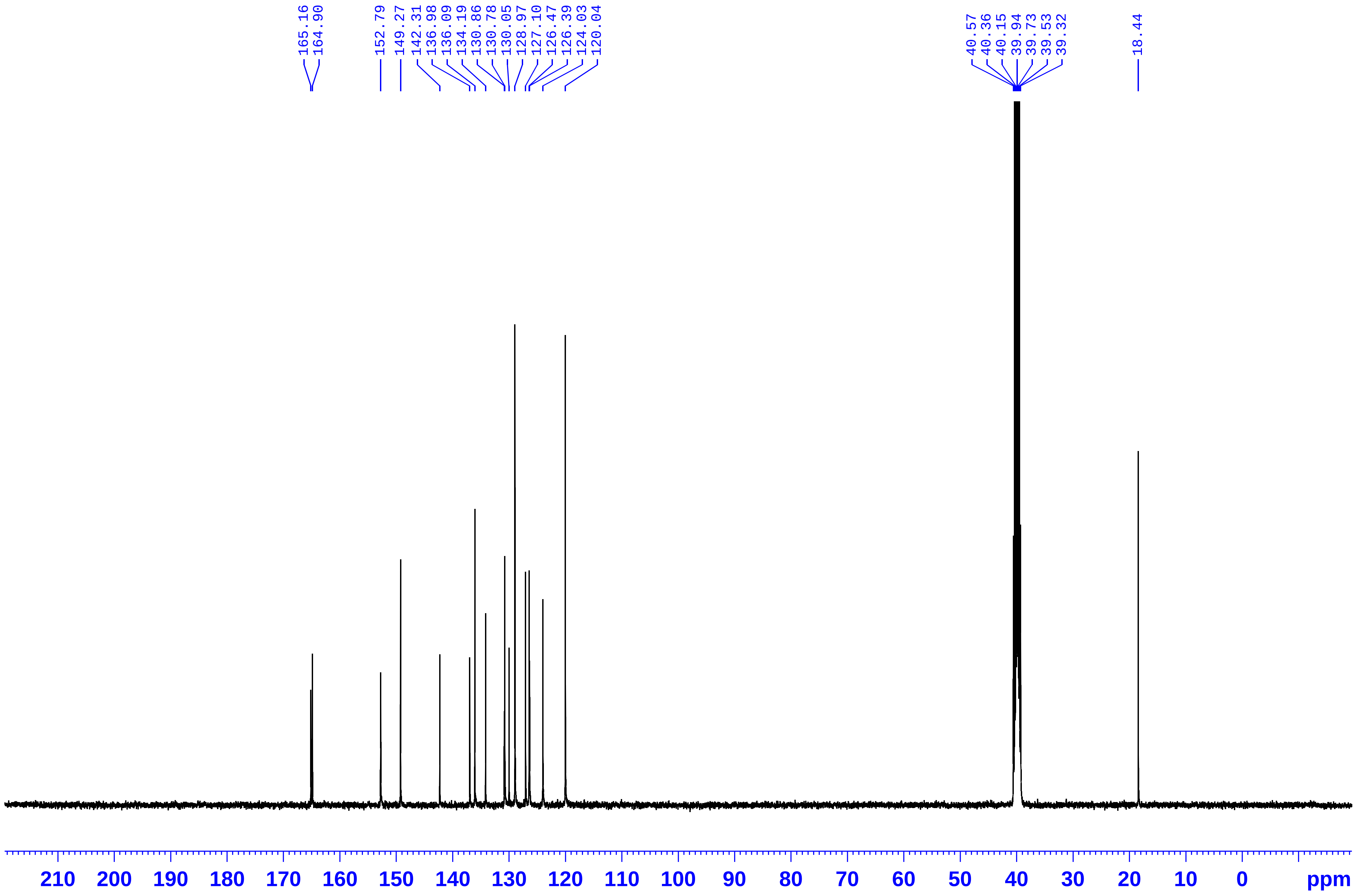

Mohamed khalifa-R8-carbon-ES

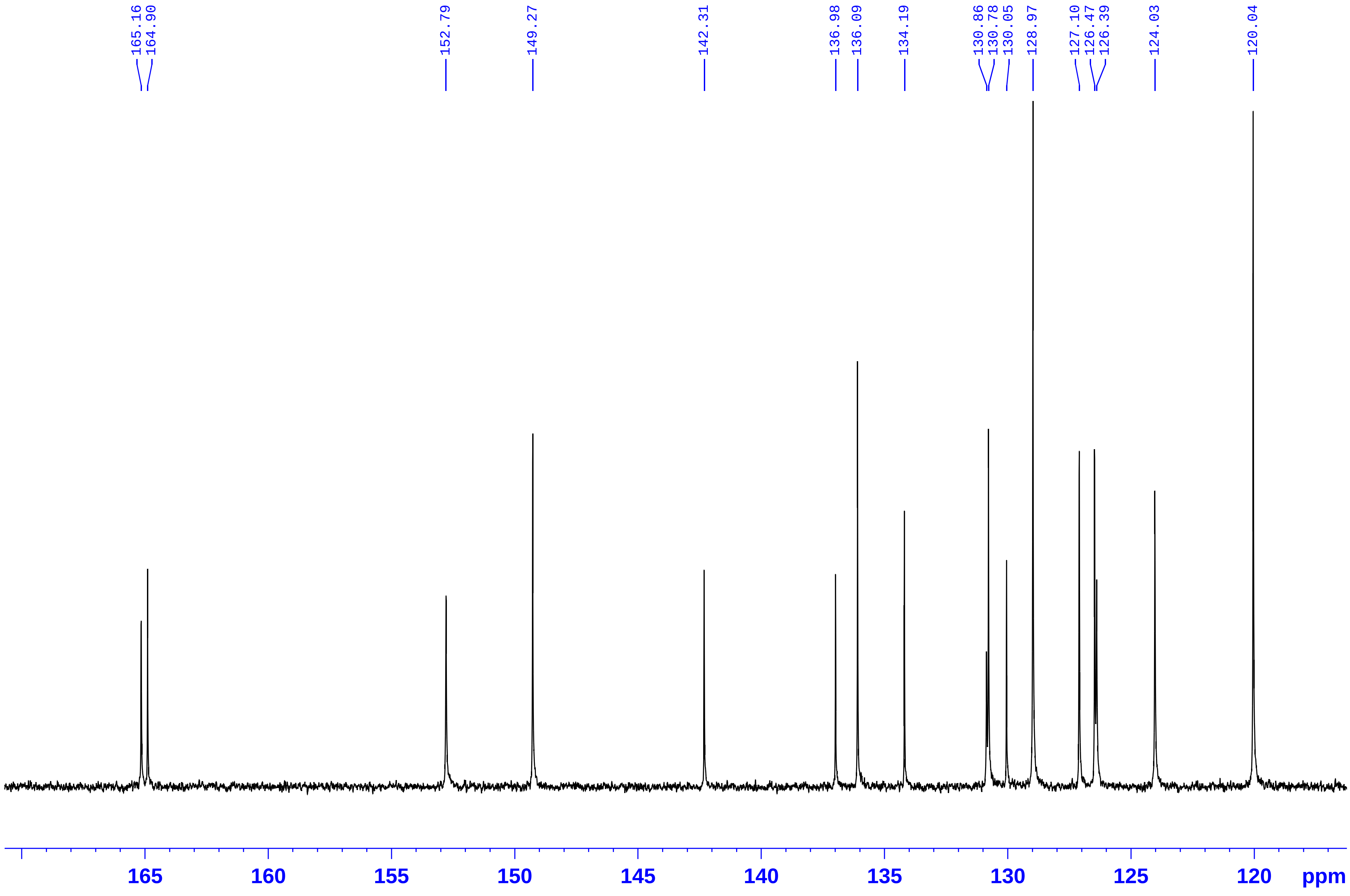

Mohamed khalifa-12-carbon

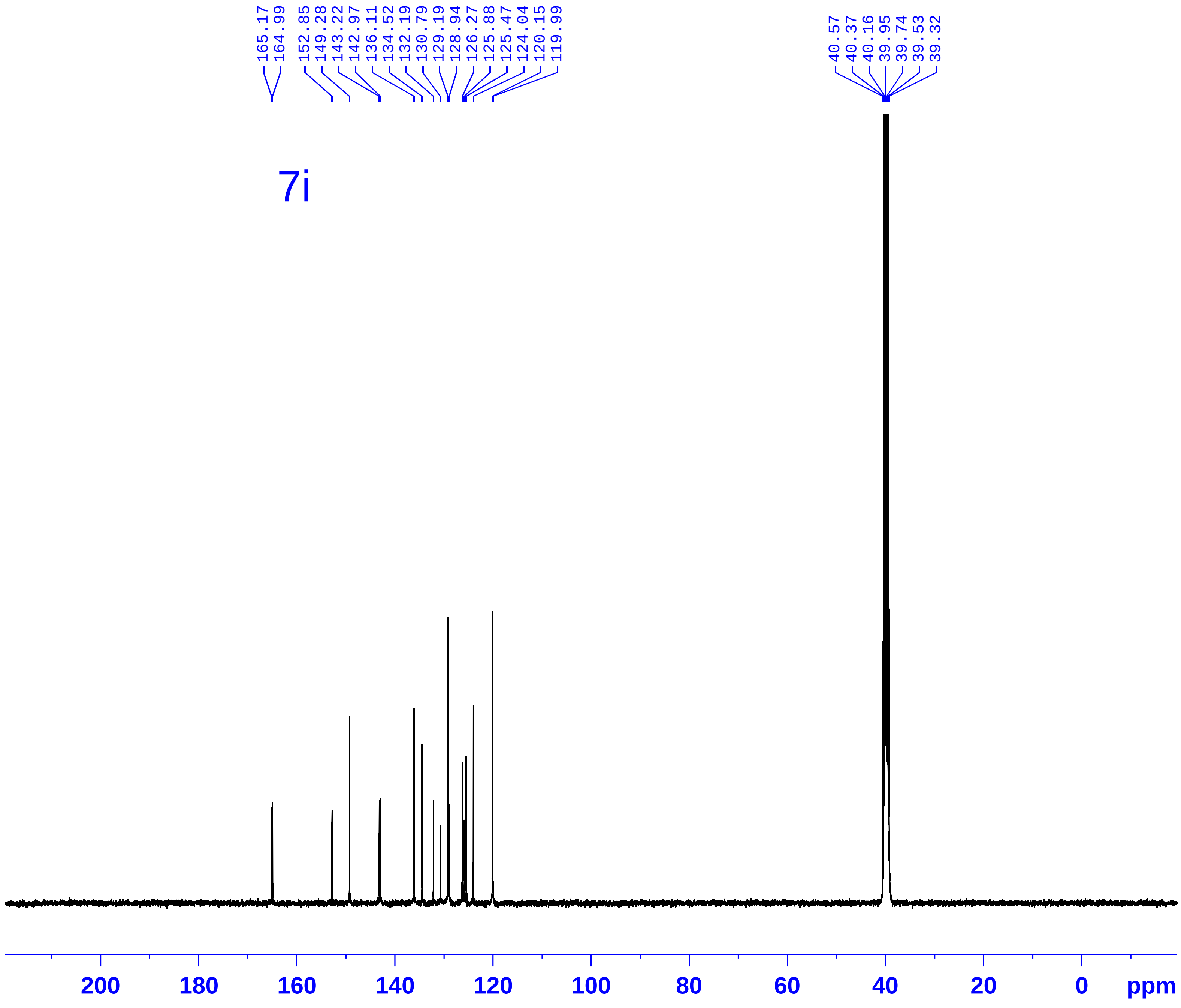


```
Current Data Parameters
NAME     Mohamed khalifa-R12-carbon-ES
EXPNO                  10
PROCNO                  1

F2 - Acquisition Parameters
Date_            20201202
Time                 4.58 h
INSTRUM             spect
PROBHD   Z108618_0945 (
PULPROG           zgpg30
TD                 65536
SOLVENT             DMSO
NS                  2100
DS                     4
SWH            24038.461 Hz
FIDRES          0.733596 Hz
AQ             1.3631488 sec
RG                197.77
DW                20.800 usec
DE                  6.50 usec
TE                 293.8 K
D1            2.00000000 sec
D11           0.03000000 sec
TD0                    1
SFO1         100.6404331 MHz
NUC1                 13C
P1                 10.00 usec
PLW1         47.00000000 W
SFO2         400.2016008 MHz
NUC2                  1H
CPDPRG[2         waltz16
PCPD2              90.00 usec
PLW2         13.00000000 W
PLW12         0.29249999 W
PLW13         0.14713000 W

F2 - Processing parameters
SI                 32768
SF           100.6303700 MHz
WDW                   EM
SSB      0
LB                  1.00 Hz
GB       0
PC                  1.40
```

Mohamed khalifa-12-carbon

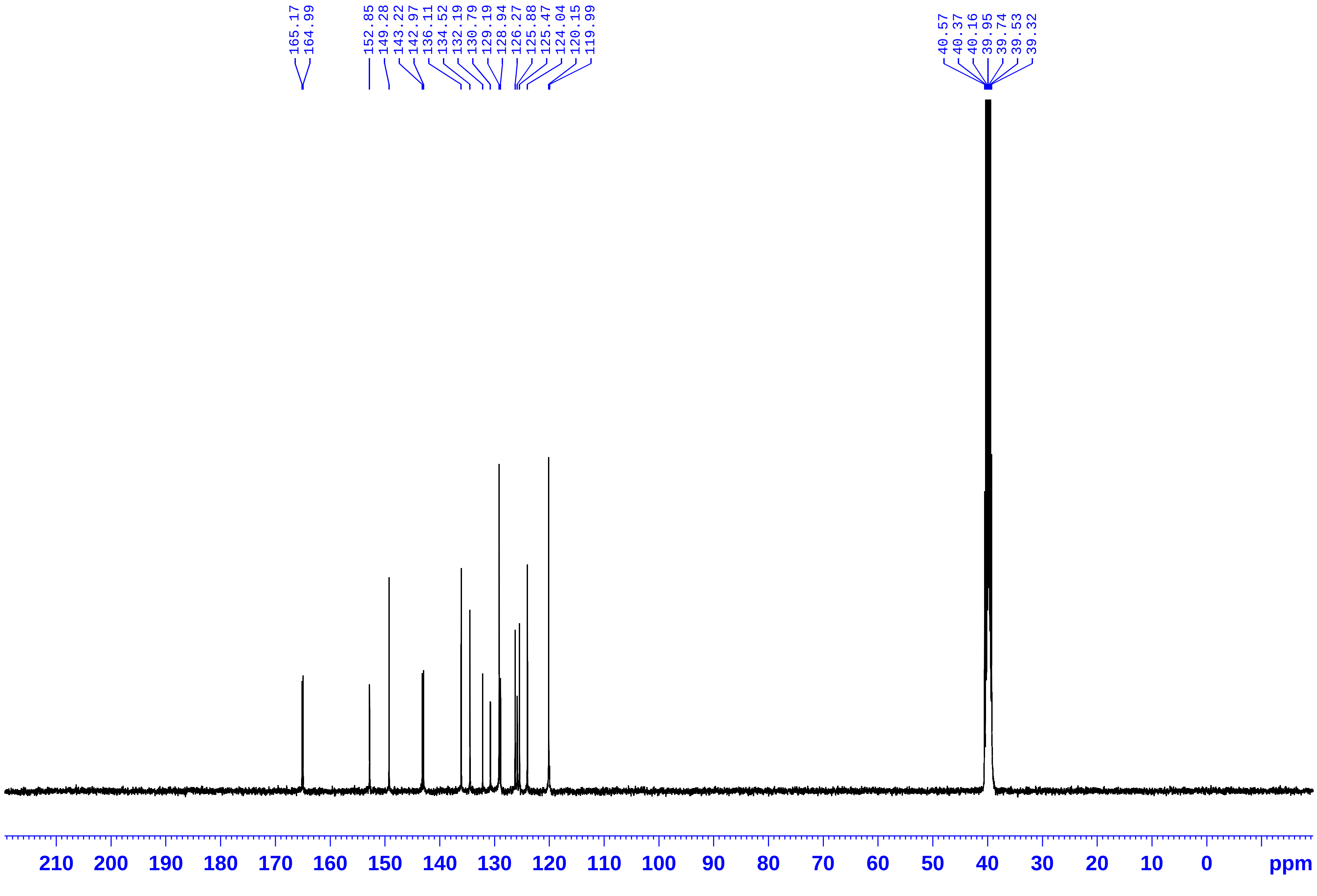

Mohamed khalifa-12-carbon

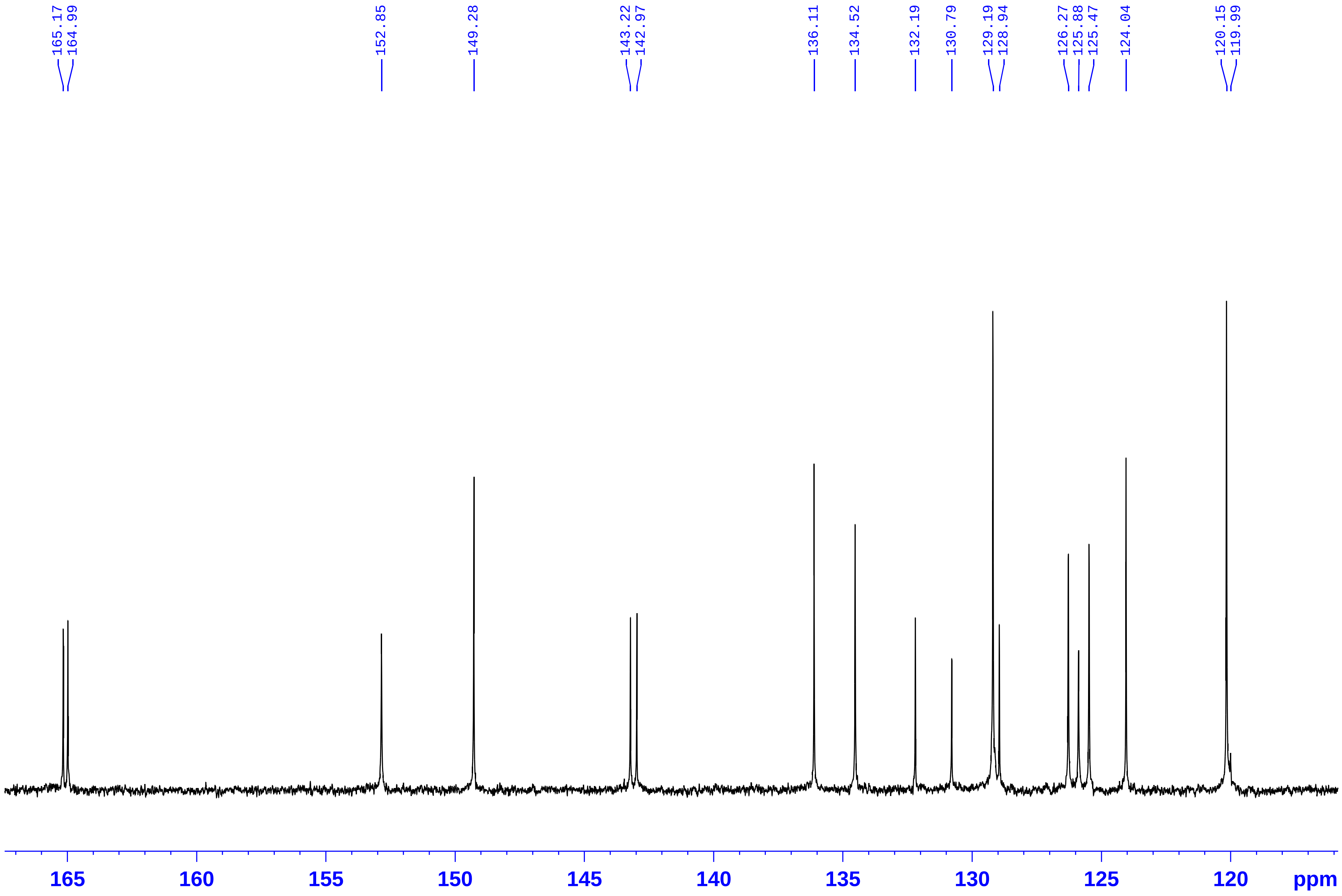

Mohamed khalifa-R20-carbon-ES

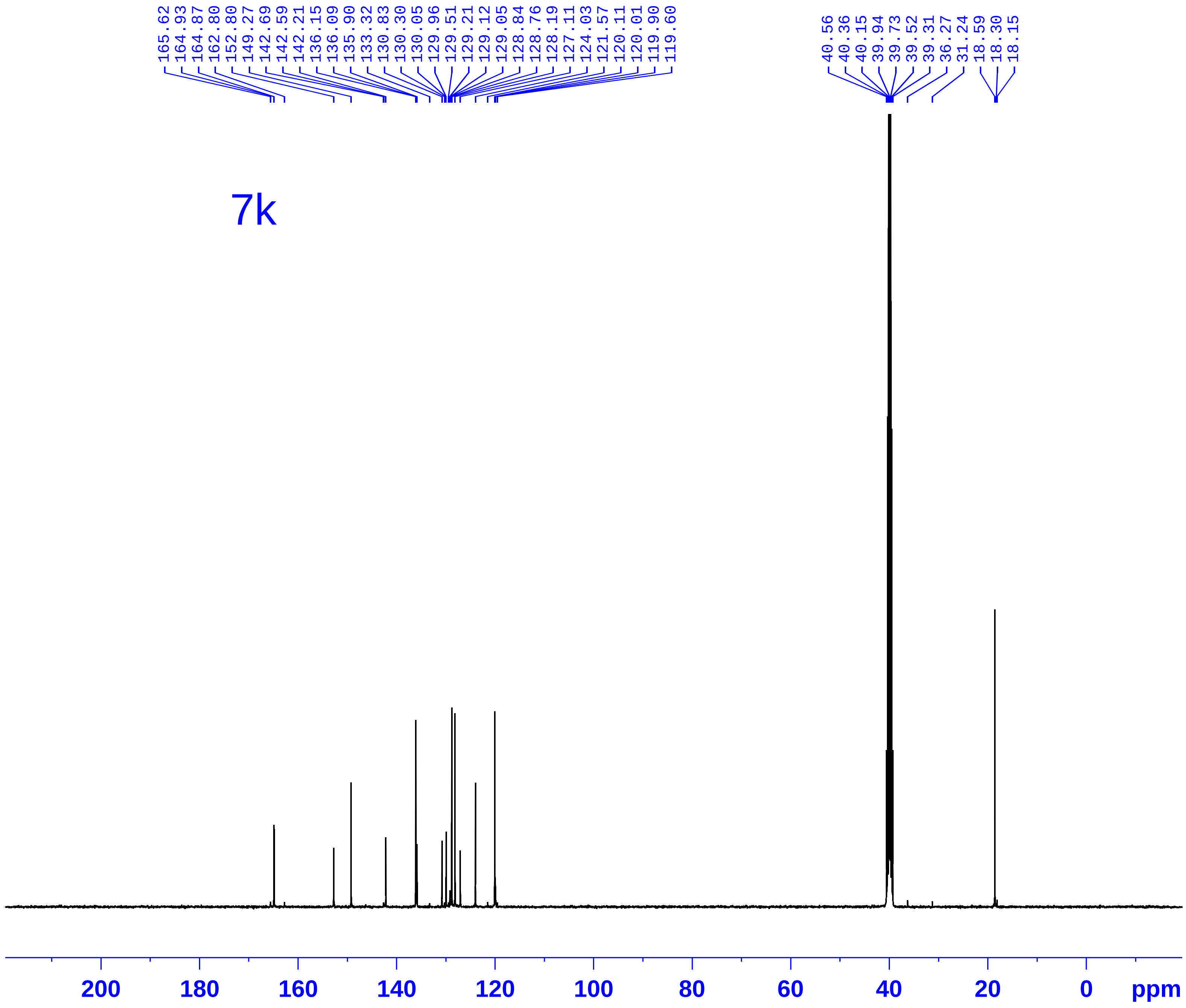

Faculty of Pharmacy

```
Current Data Parameters
NAME      Mohamed khalifa-R20-carbon-ES
EXPNO                    10
PROCNO                    1

F2 - Acquisition Parameters
Date_              20201202
Time                   2.54 h
INSTRUM               spect
PROBHD    Z108618_0945 (
PULPROG             zgpg30
TD                    65536
SOLVENT                DMSO
NS                     2200
DS                        4
SWH               24038.461 Hz
FIDRES             0.733596 Hz
AQ                1.3631488 sec
RG                   197.77
DW                   20.800 usec
DE                     6.50 usec
TE                    293.5 K
D1               2.00000000 sec
D11              0.03000000 sec
TD0                       1
SFO1            100.6404331 MHz
NUC1                    13C
P1                    10.00 usec
PLW1            47.00000000 W
SFO2            400.2016008 MHz
NUC2                     1H
CPDPRG[2            waltz16
PCPD2                 90.00 usec
PLW2            13.00000000 W
PLW12            0.29249999 W
PLW13            0.14713000 W

F2 - Processing parameters
SI                    32768
SF              100.6303700 MHz
WDW                      EM
SSB       0
LB                     1.00 Hz
GB        0
PC                     1.40
```

Mohamed khalifa-R20-carbon-ES

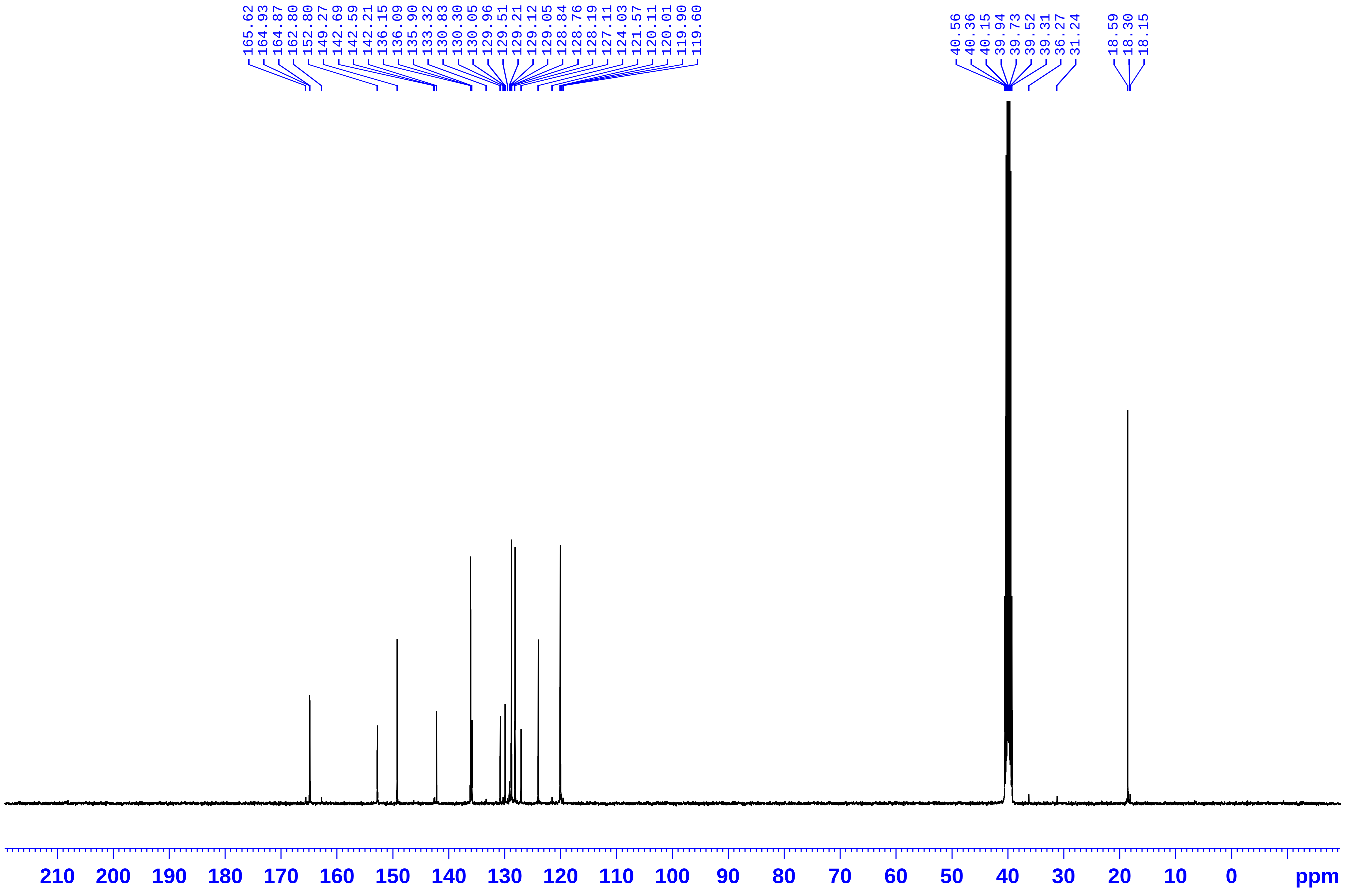

Mohamed khalifa-R20-carbon-ES

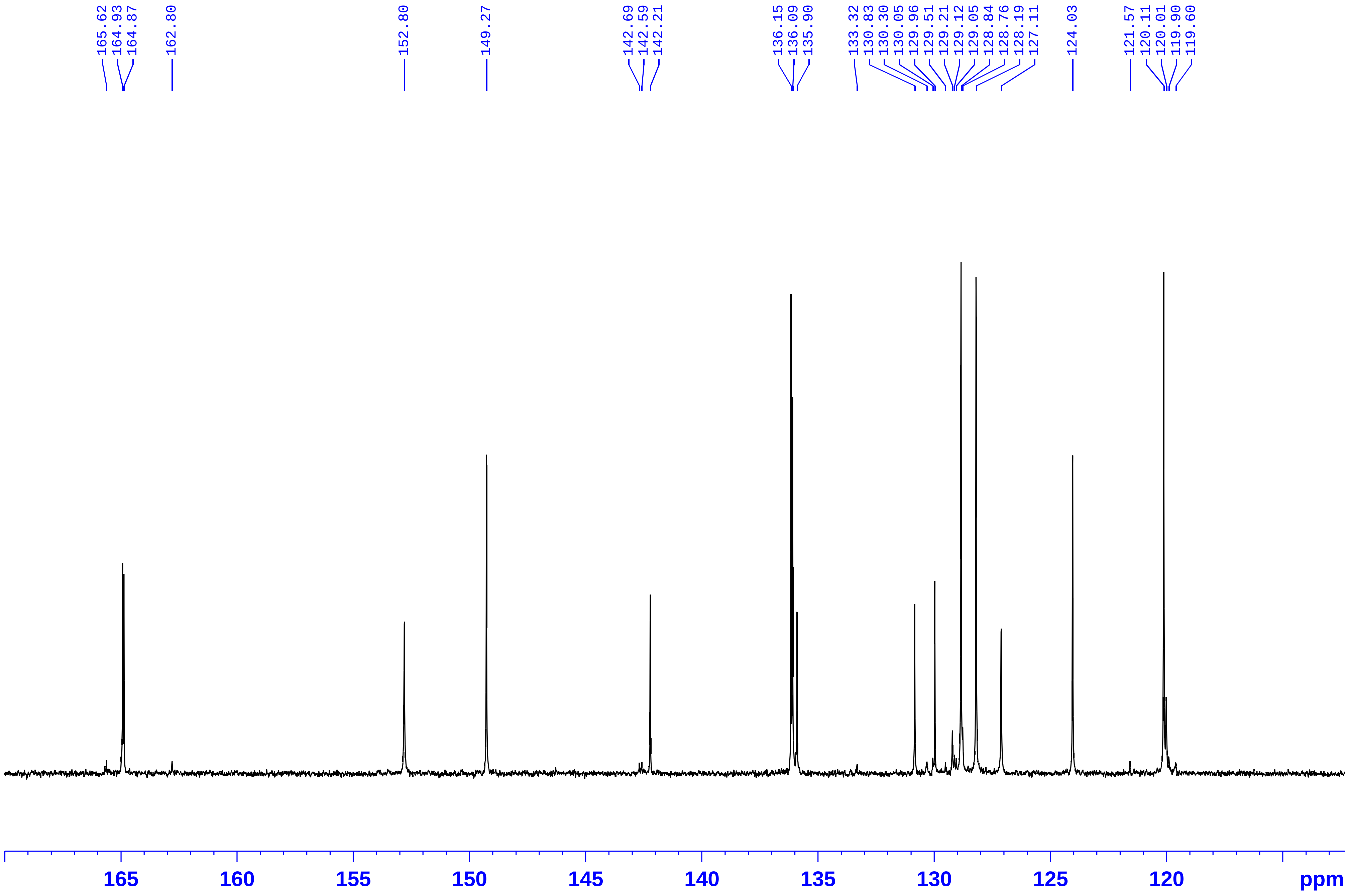

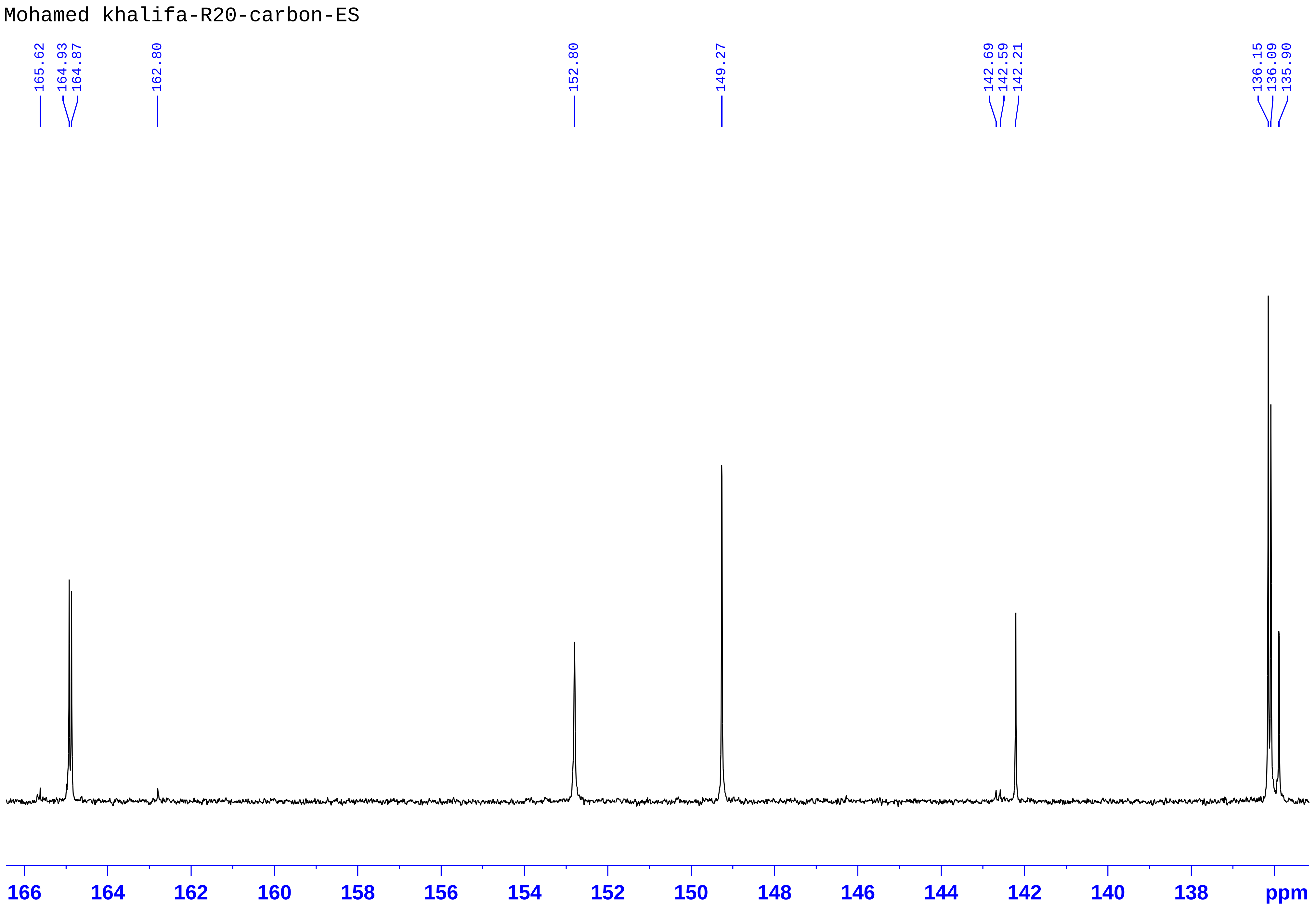
Mohamed khalifa-R20-carbon-ES
165.62
164.93
164.87
162.80
152.80
149.27
142.69
142.59
142.21
136.15
136.09
135.90
166
164
162
160
158
156
154
152
150
148
146
144
142
140
138
ppm

Mohamed khalifa-A1-carbon-ES

10

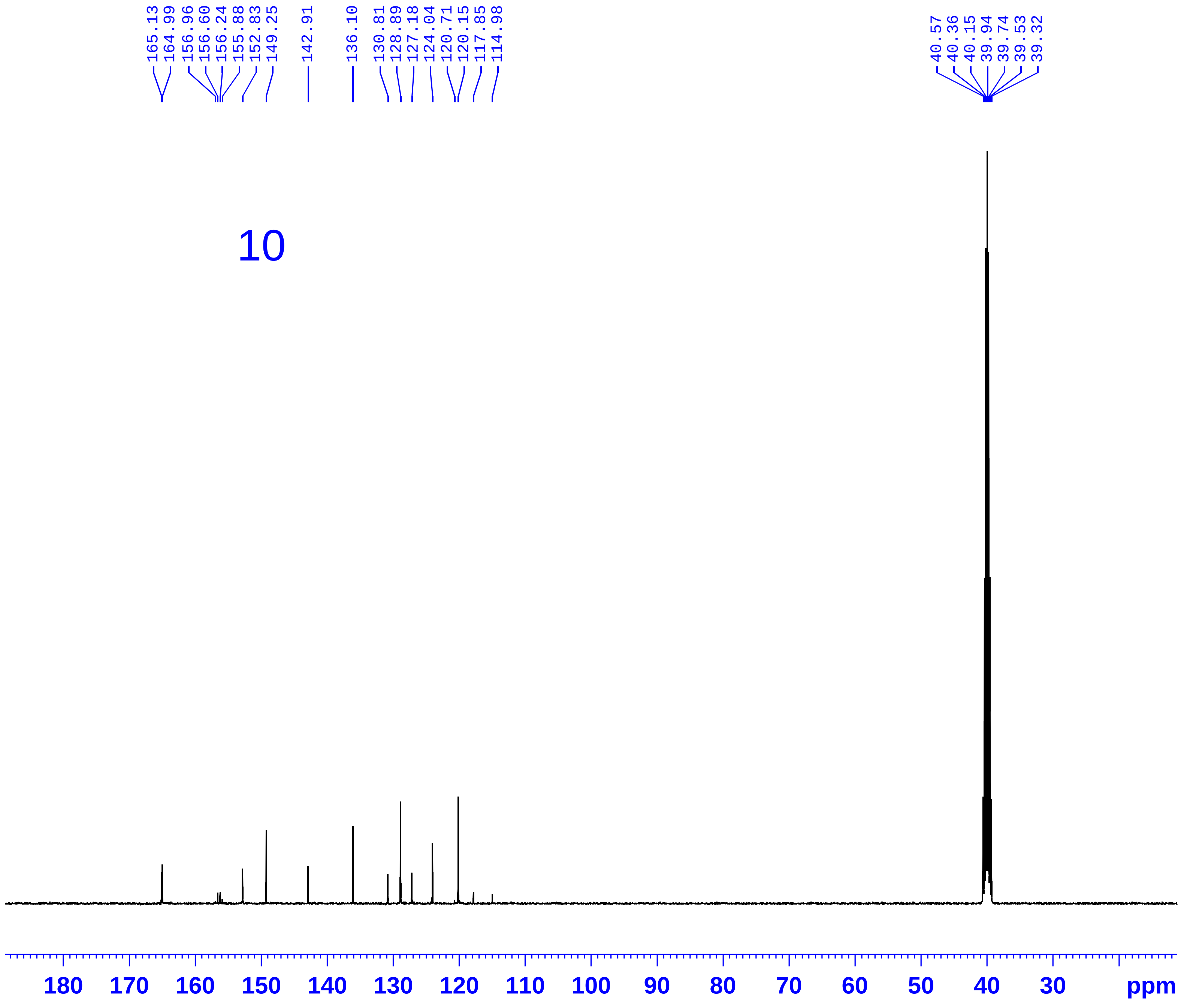

BRUKER

Faculty of Pharmacy

Current Data Parameters

NAME	Mohamed khalifa-A1-carbon-ES	
EXPNO	10	
PROCNO	1	

F2 - Acquisition Parameters

Date_	20201202	
Time	13.12	h
INSTRUM	spect	
PROBHD	Z108618_0945 (	
PULPROG	zgpg30	
TD	65536	
SOLVENT	DMSO	
NS	2100	
DS	4	
SWH	24038.461	Hz
FIDRES	0.733596	Hz
AQ	1.3631488	sec
RG	197.77	
DW	20.800	usec
DE	6.50	usec
TE	293.8	K
D1	2.00000000	sec
D11	0.03000000	sec
TD0	1	
SFO1	100.6404331	MHz
NUC1	13C	
P1	10.00	usec
PLW1	47.00000000	W
SFO2	400.2016008	MHz
NUC2	1H	
CPDPRG[2	waltz16	
PCPD2	90.00	usec
PLW2	13.00000000	W
PLW12	0.29249999	W
PLW13	0.14713000	W

F2 - Processing parameters

SI	32768	
SF	100.6303700	MHz
WDW	EM	
SSB	0	
LB	1.00	Hz
GB	0	
PC	1.40	

Mohamed khalifa-A1-carbon-ES

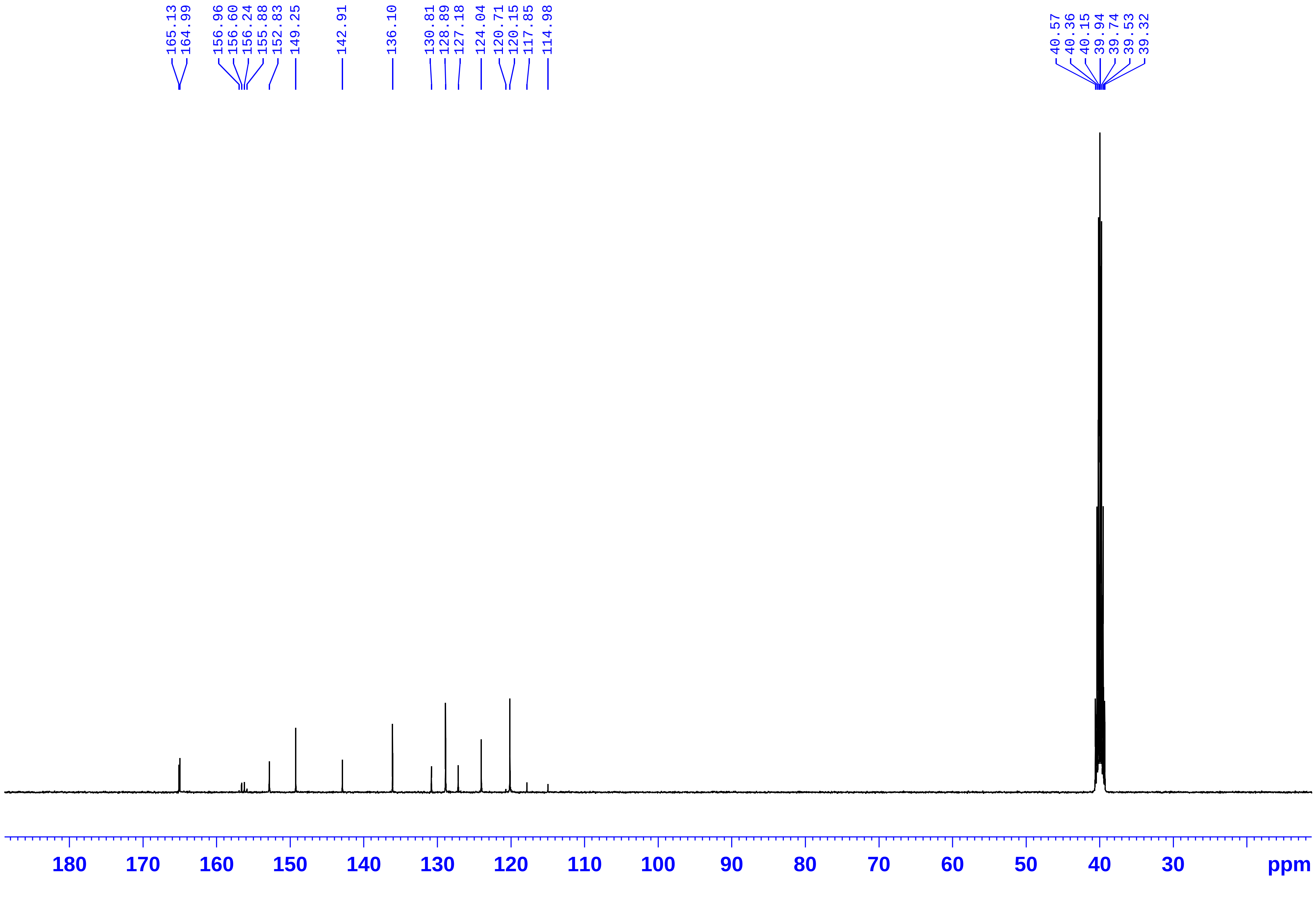

Mohamed khalifa-A1-carbon-ES

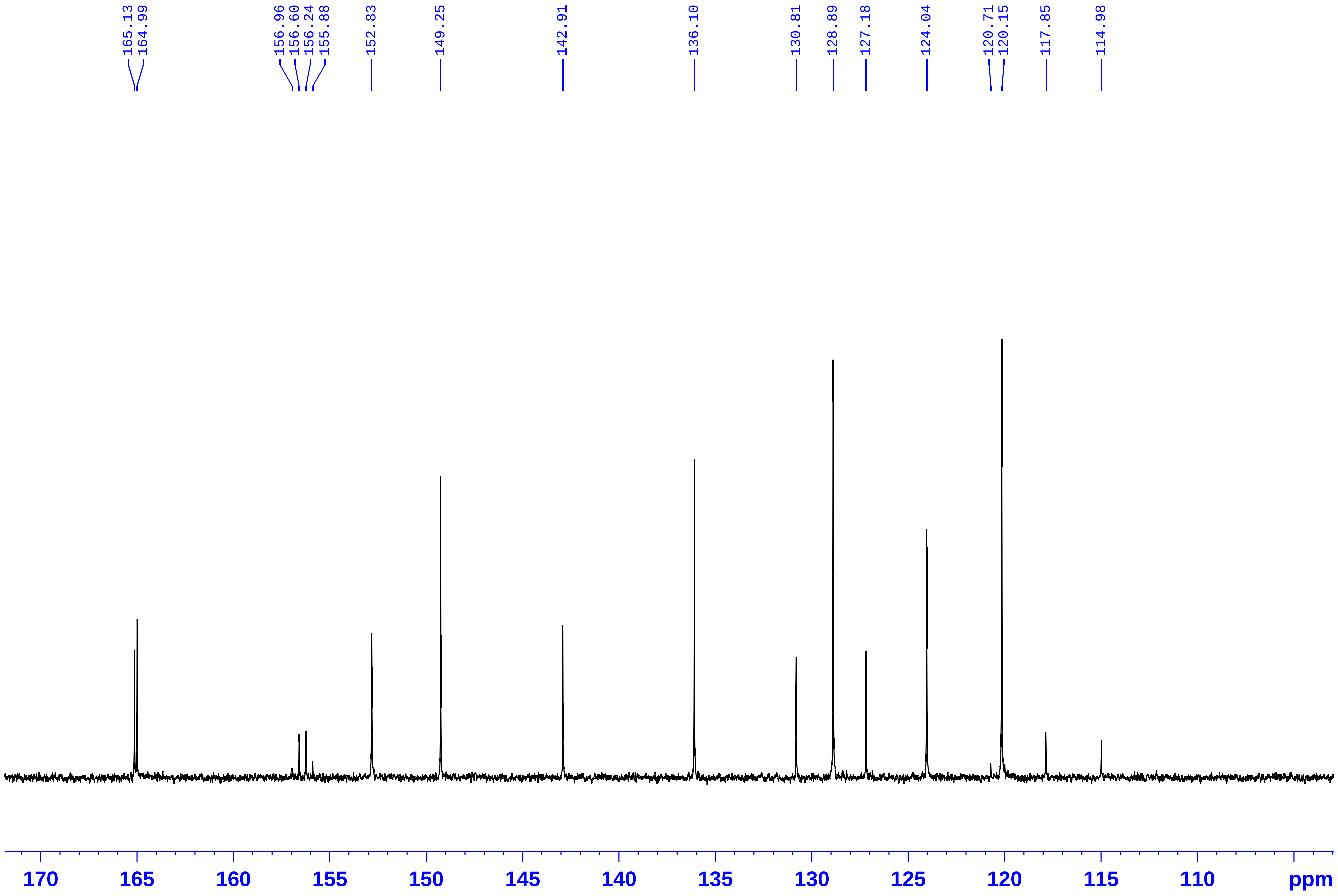

Mohamed khalifa-X1-carbon-ES

11a

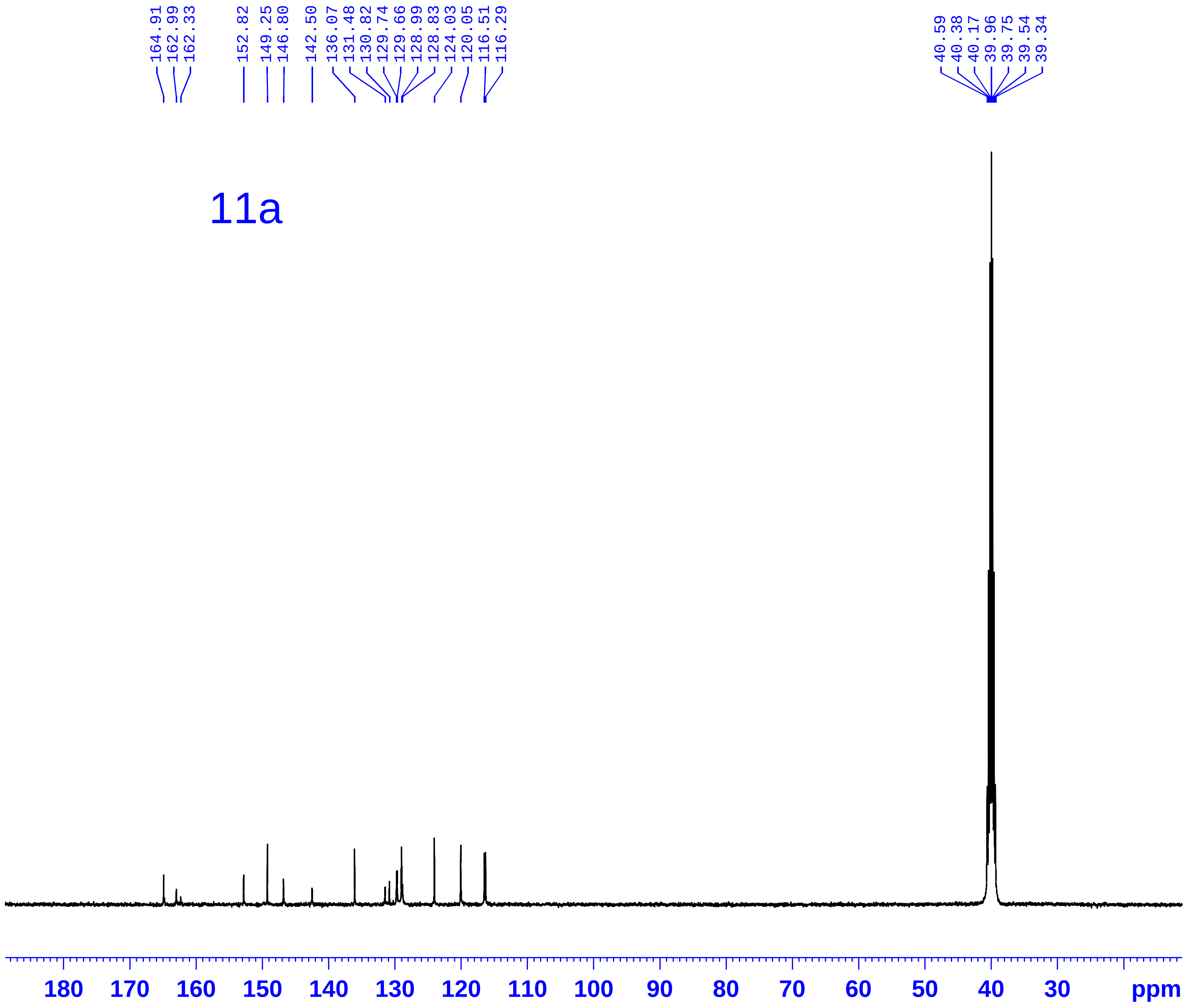

Faculty of Pharmacy

```
Current Data Parameters
NAME     Mohamed khalifa-X1-carbon-ES
EXPNO                 10
PROCNO                 1

F2 - Acquisition Parameters
Date_           20201202
Time               15.16 h
INSTRUM            spect
PROBHD   Z108618_0945 (
PULPROG          zgpg30
TD                 65536
SOLVENT             DMSO
NS                  2100
DS                     4
SWH            24038.461 Hz
FIDRES          0.733596 Hz
AQ             1.3631488 sec
RG                197.77
DW                20.800 usec
DE                  6.50 usec
TE                 295.1 K
D1            2.00000000 sec
D11           0.03000000 sec
TD0                    1
SFO1         100.6404331 MHz
NUC1                 13C
P1                 10.00 usec
PLW1         47.00000000 W
SFO2         400.2016008 MHz
NUC2                  1H
CPDPRG[2         waltz16
PCPD2              90.00 usec
PLW2         13.00000000 W
PLW12         0.29249999 W
PLW13         0.14713000 W

F2 - Processing parameters
SI                 32768
SF           100.6303700 MHz
WDW                   EM
SSB      0
LB                  1.00 Hz
GB       0
PC                  1.40
```

Mohamed khalifa-X1-carbon-ES

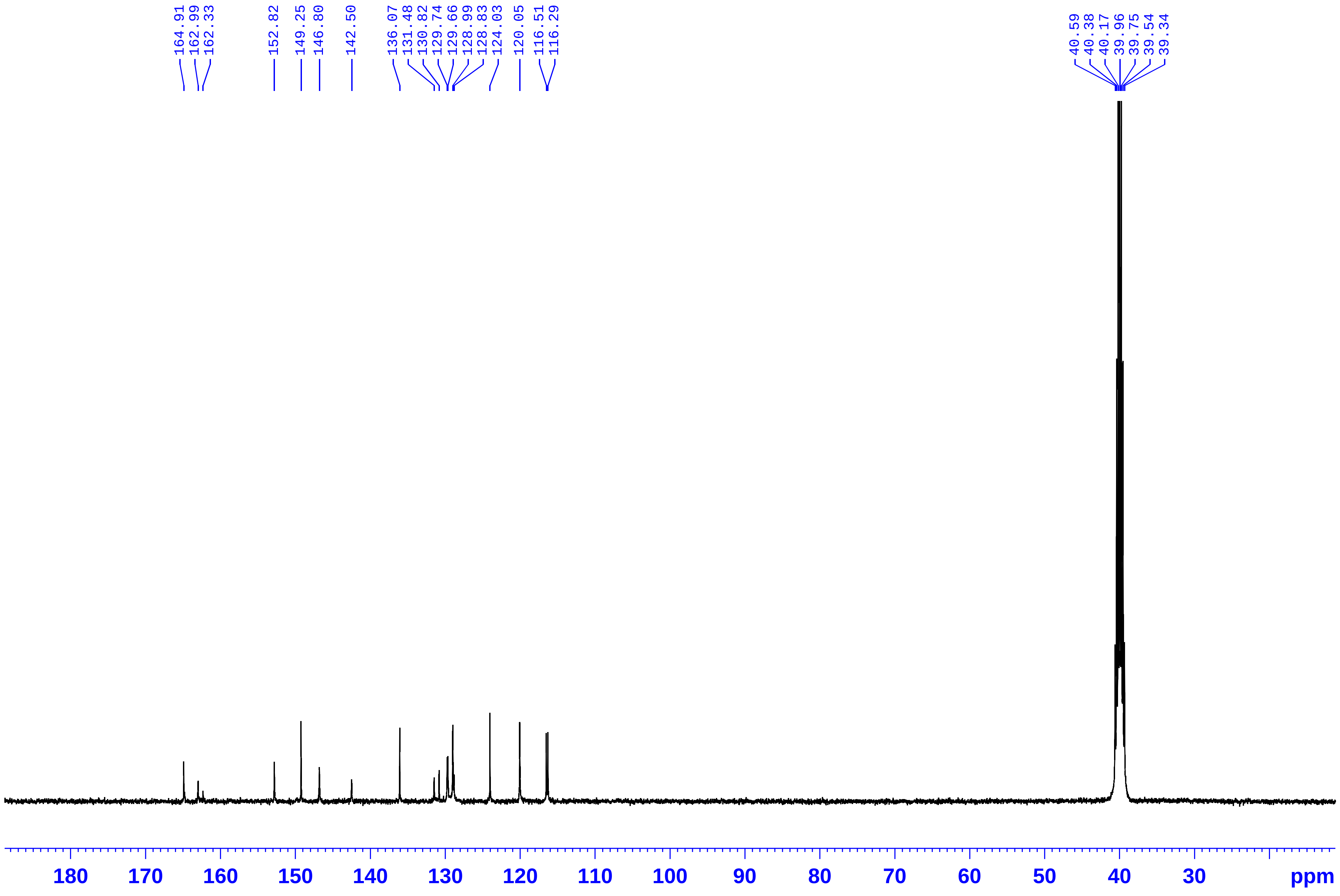

Mohamed khalifa-X1-carbon-ES

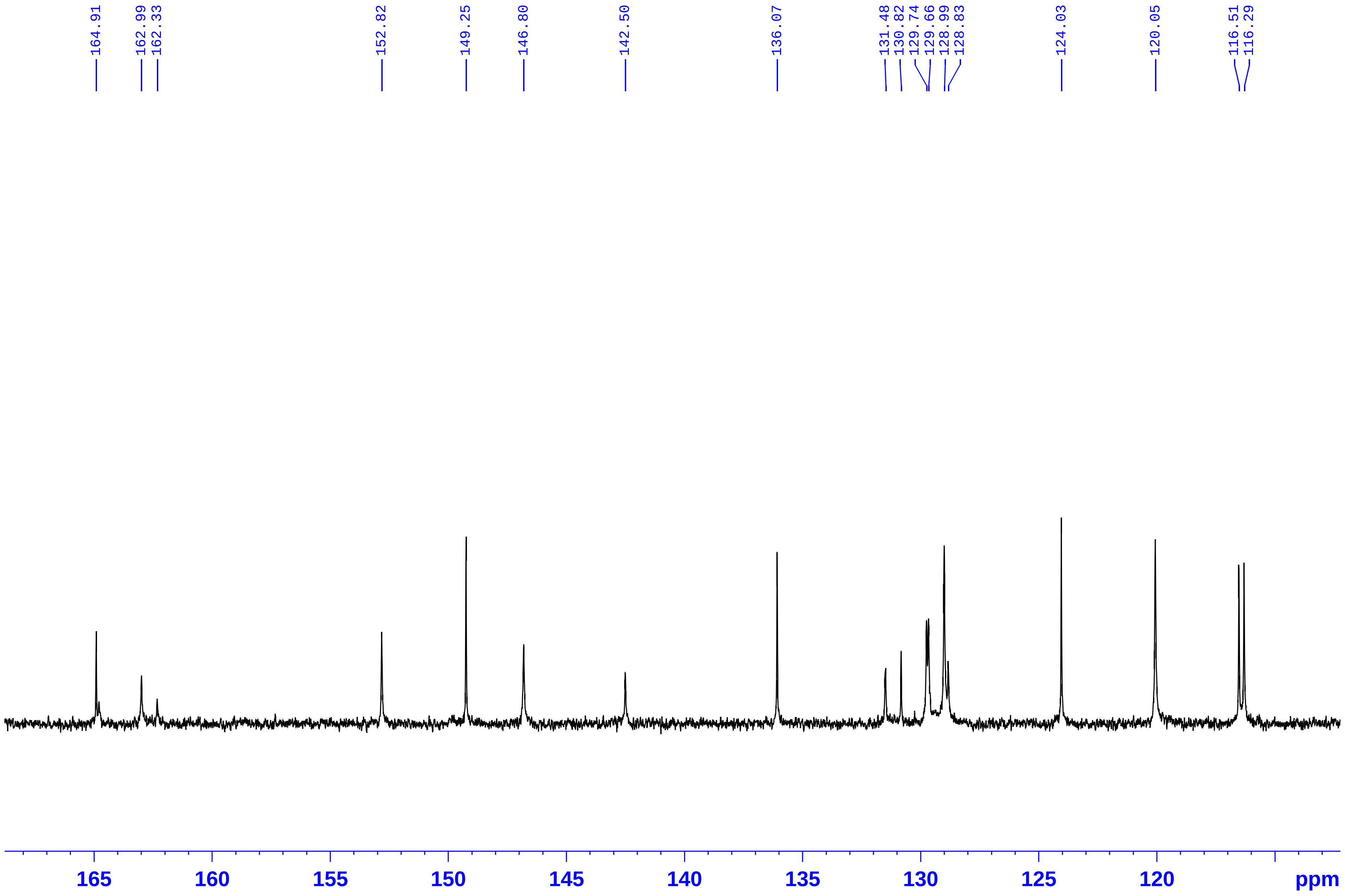

Mohamed khalifa-X2-carbon-ES

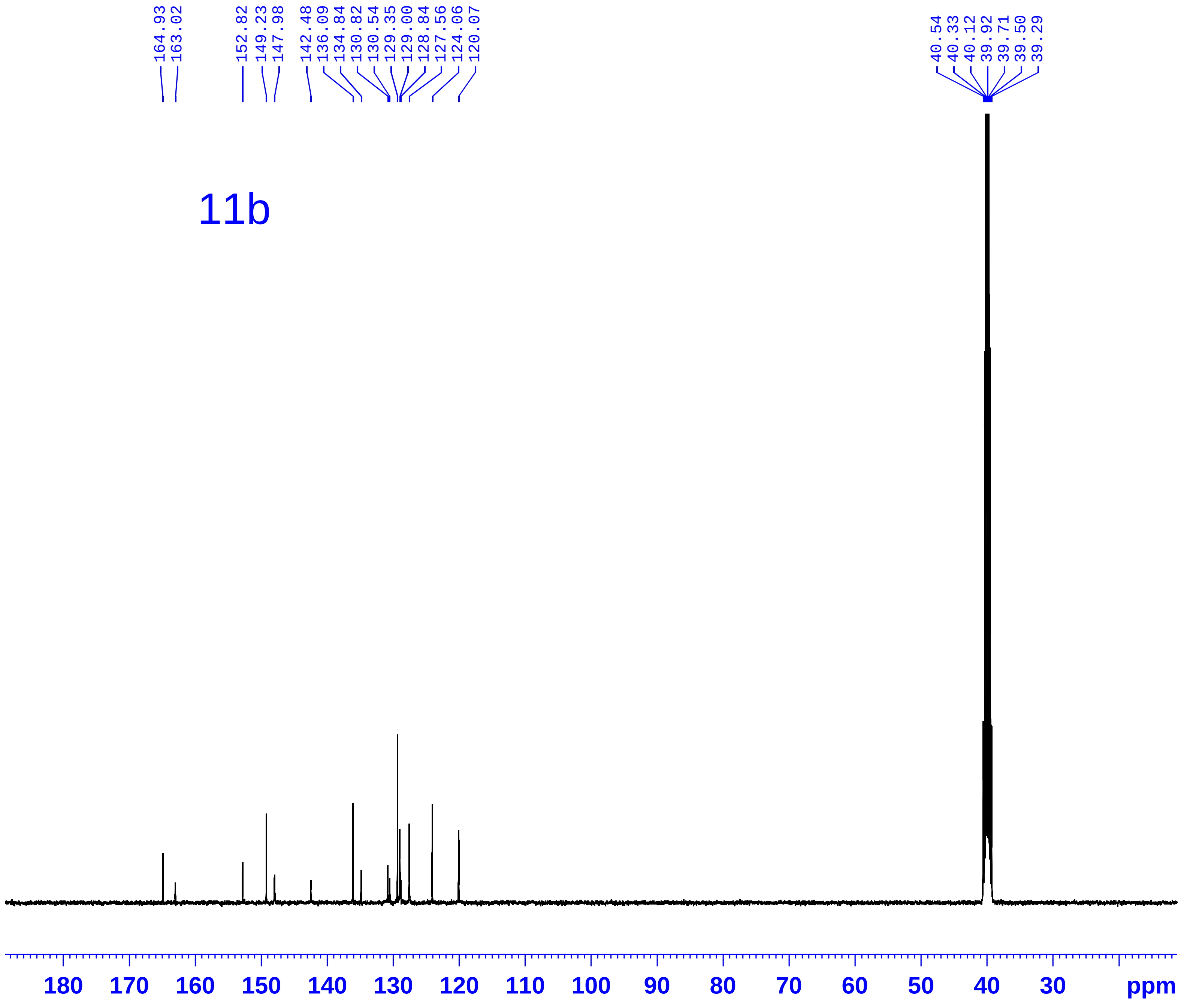

11b

```
Current Data Parameters
NAME      Mohamed khalifa-X2-carbon-ES
EXPNO                  10
PROCNO                  1

F2 - Acquisition Parameters
Date_            20201202
Time                17.20 h
INSTRUM             spect
PROBHD   Z108618_0945 (
PULPROG            zgpg30
TD                  65536
SOLVENT              DMSO
NS                   2100
DS                      4
SWH             24038.461 Hz
FIDRES           0.733596 Hz
AQ              1.3631488 sec
RG                 197.77
DW                 20.800 usec
DE                   6.50 usec
TE                  293.8 K
D1             2.00000000 sec
D11            0.03000000 sec
TD0                     1
SFO1          100.6404331 MHz
NUC1                  13C
P1                  10.00 usec
PLW1          47.00000000 W
SFO2          400.2016008 MHz
NUC2                   1H
CPDPRG[2          waltz16
PCPD2               90.00 usec
PLW2          13.00000000 W
PLW12          0.29249999 W
PLW13          0.14713000 W

F2 - Processing parameters
SI                  32768
SF            100.6303700 MHz
WDW                    EM
SSB      0
LB                   1.00 Hz
GB       0
PC                   1.40
```

Faculty of Pharmacy

Mohamed khalifa-X2-carbon-ES

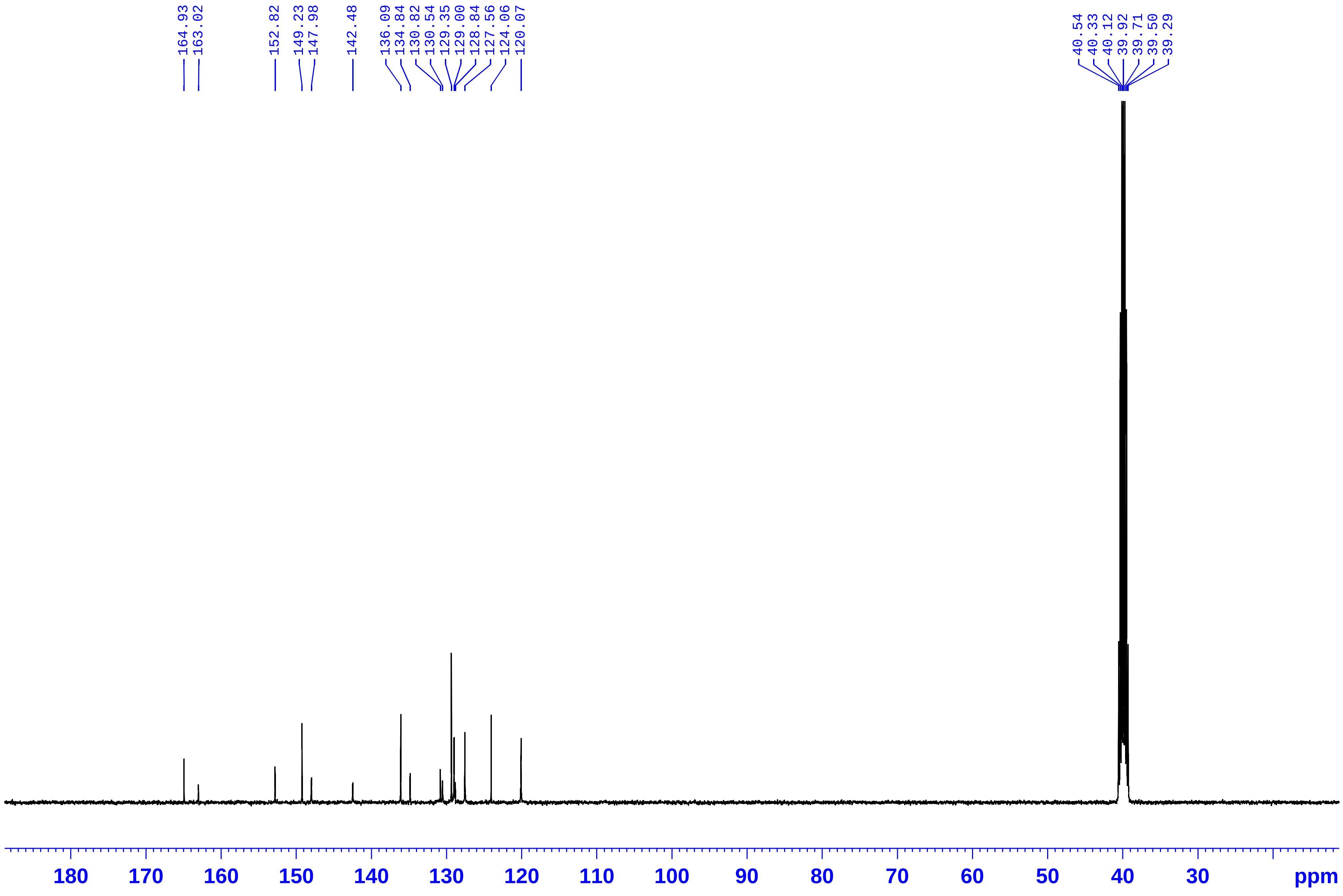

Mohamed khalifa-X2-carbon-ES

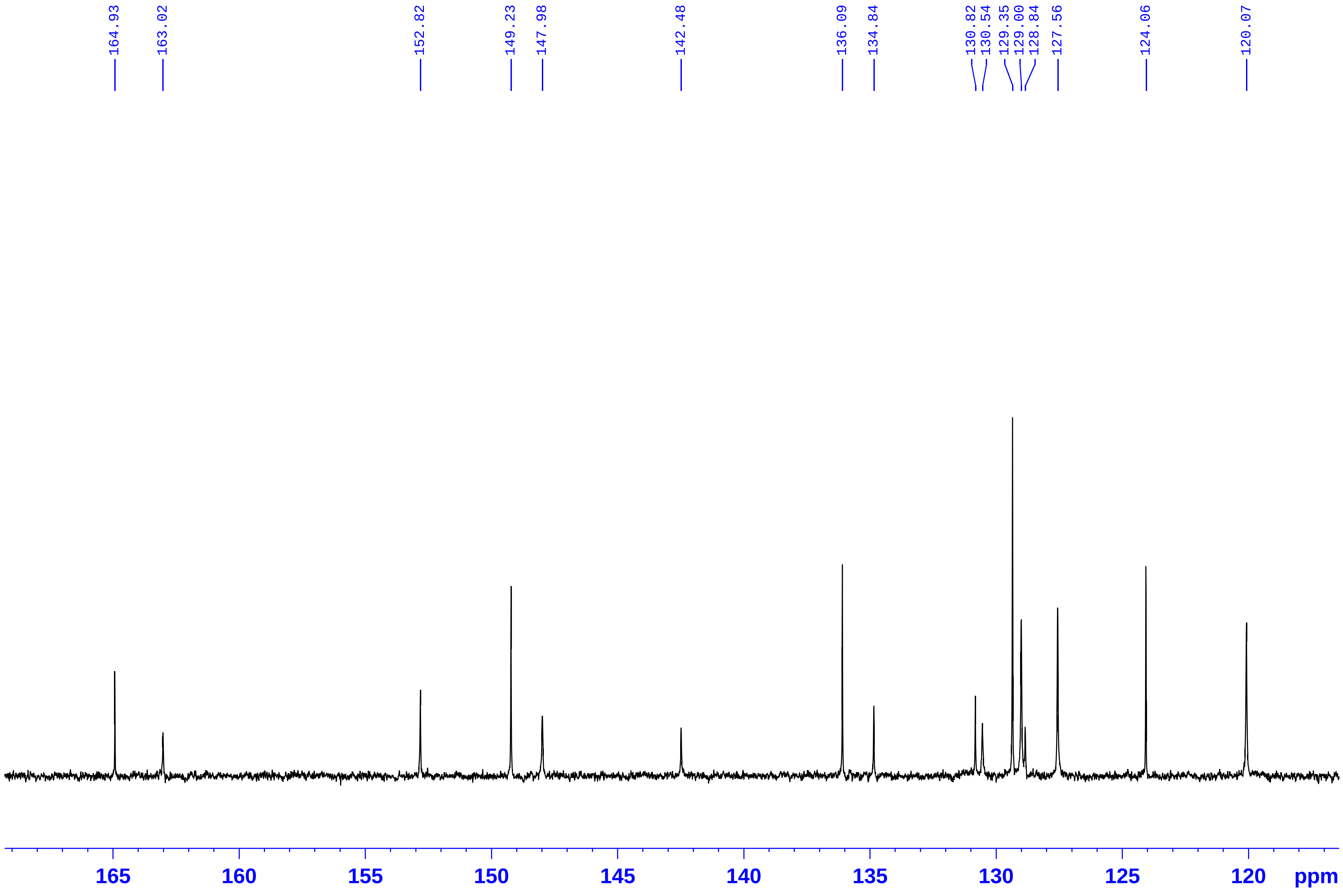

Mohamed khalifa-X4-carbon-ES

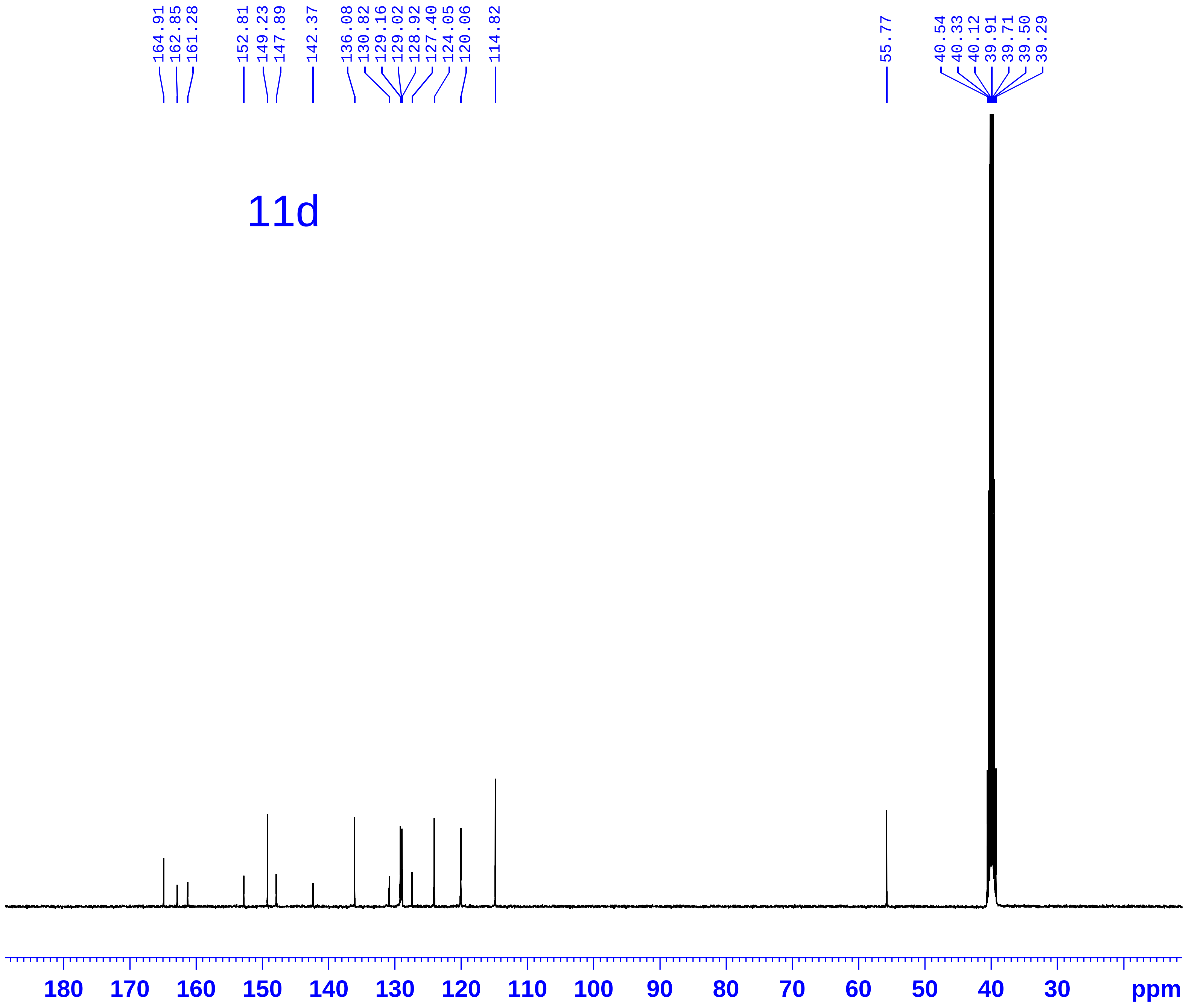

BRUKER

Faculty of Pharmacy

Current Data Parameters		
NAME	Mohamed khalifa-X4-carbon-ES	
EXPNO	10	
PROCNO	1	
F2 - Acquisition Parameters		
Date_	20201202	
Time	19.23	h
INSTRUM	spect	
PROBHD	Z108618_0945 (	
PULPROG	zgpg30	
TD	65536	
SOLVENT	DMSO	
NS	2100	
DS	4	
SWH	24038.461	Hz
FIDRES	0.733596	Hz
AQ	1.3631488	sec
RG	197.77	
DW	20.800	usec
DE	6.50	usec
TE	293.8	K
D1	2.00000000	sec
D11	0.03000000	sec
TD0	1	
SFO1	100.6404331	MHz
NUC1	13C	
P1	10.00	usec
PLW1	47.00000000	W
SFO2	400.2016008	MHz
NUC2	1H	
CPDPRG[2	waltz16	
PCPD2	90.00	usec
PLW2	13.00000000	W
PLW12	0.29249999	W
PLW13	0.14713000	W
F2 - Processing parameters		
SI	32768	
SF	100.6303700	MHz
WDW	EM	
SSB	0	
LB	1.00	Hz
GB	0	
PC	1.40	

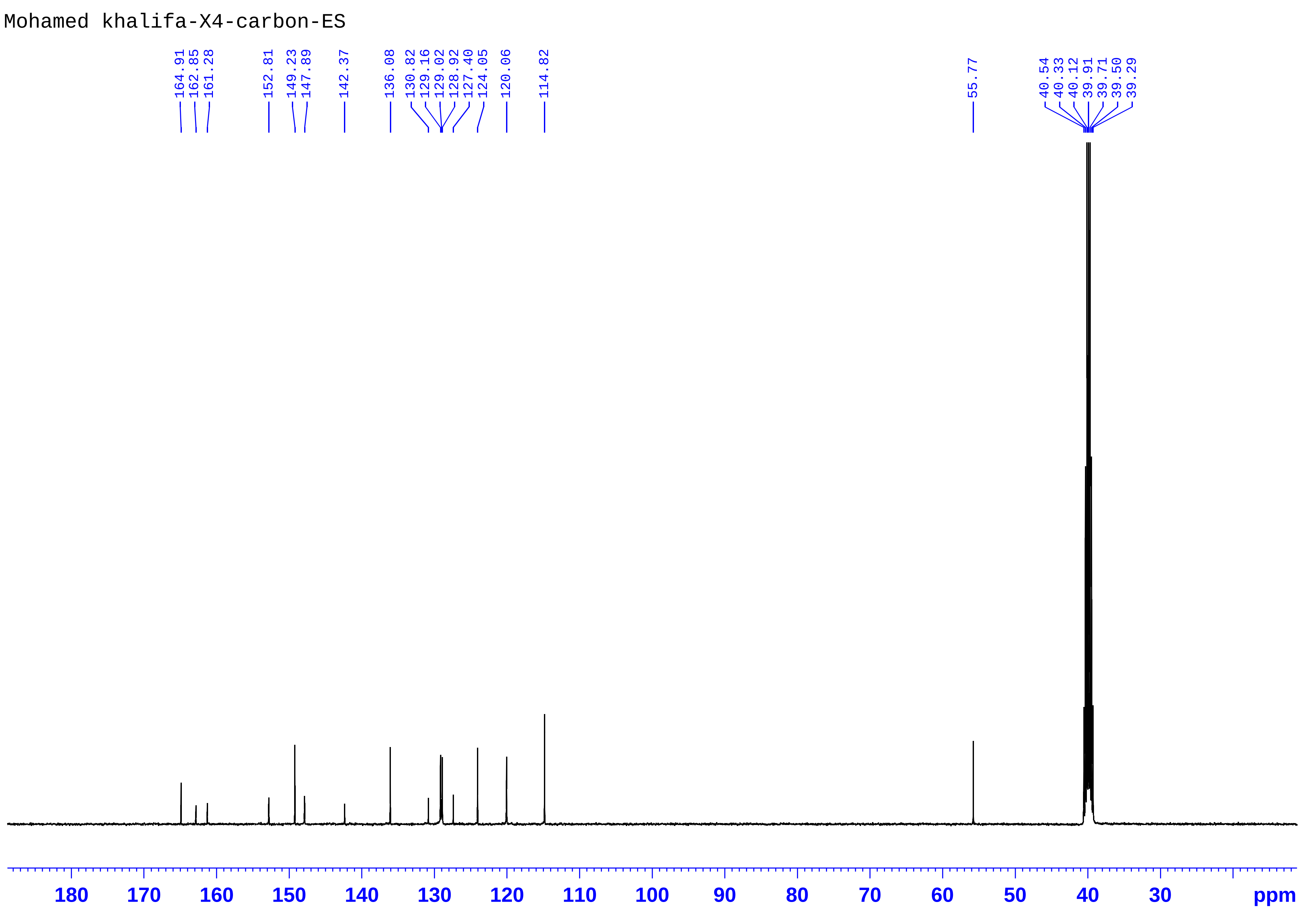
Mohamed khalifa-X4-carbon-ES
164.91
162.85
161.28
152.81
149.23
147.89
142.37
136.08
130.82
129.16
129.02
128.92
127.40
124.05
120.06
114.82
55.77
40.54
40.33
40.12
39.91
39.71
39.50
39.29
180
170
160
150
140
130
120
110
100
90
80
70
60
50
40
30
ppm

Mohamed khalifa-X4-carbon-ES

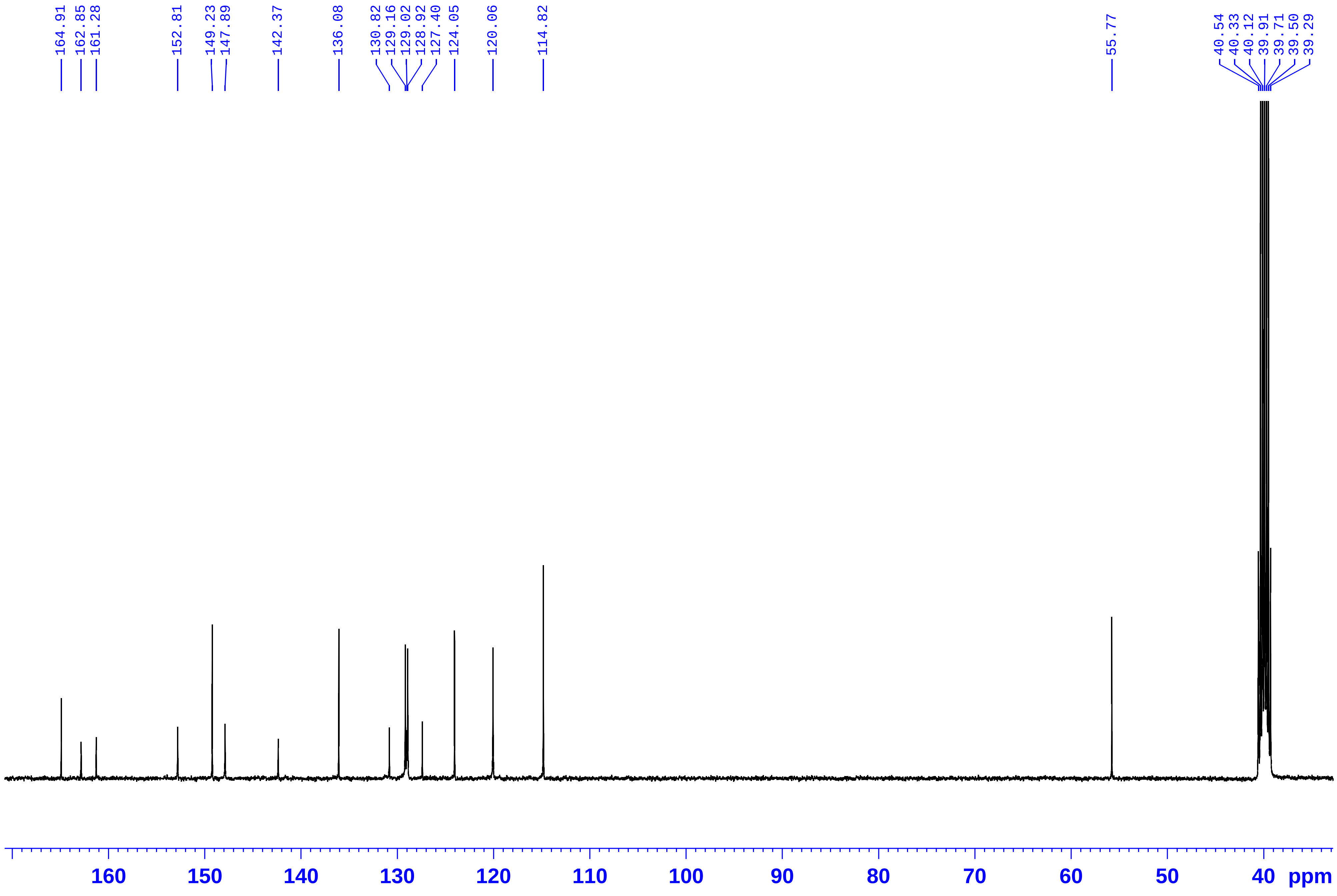

Mohamed khalifa-X4-carbon-ES

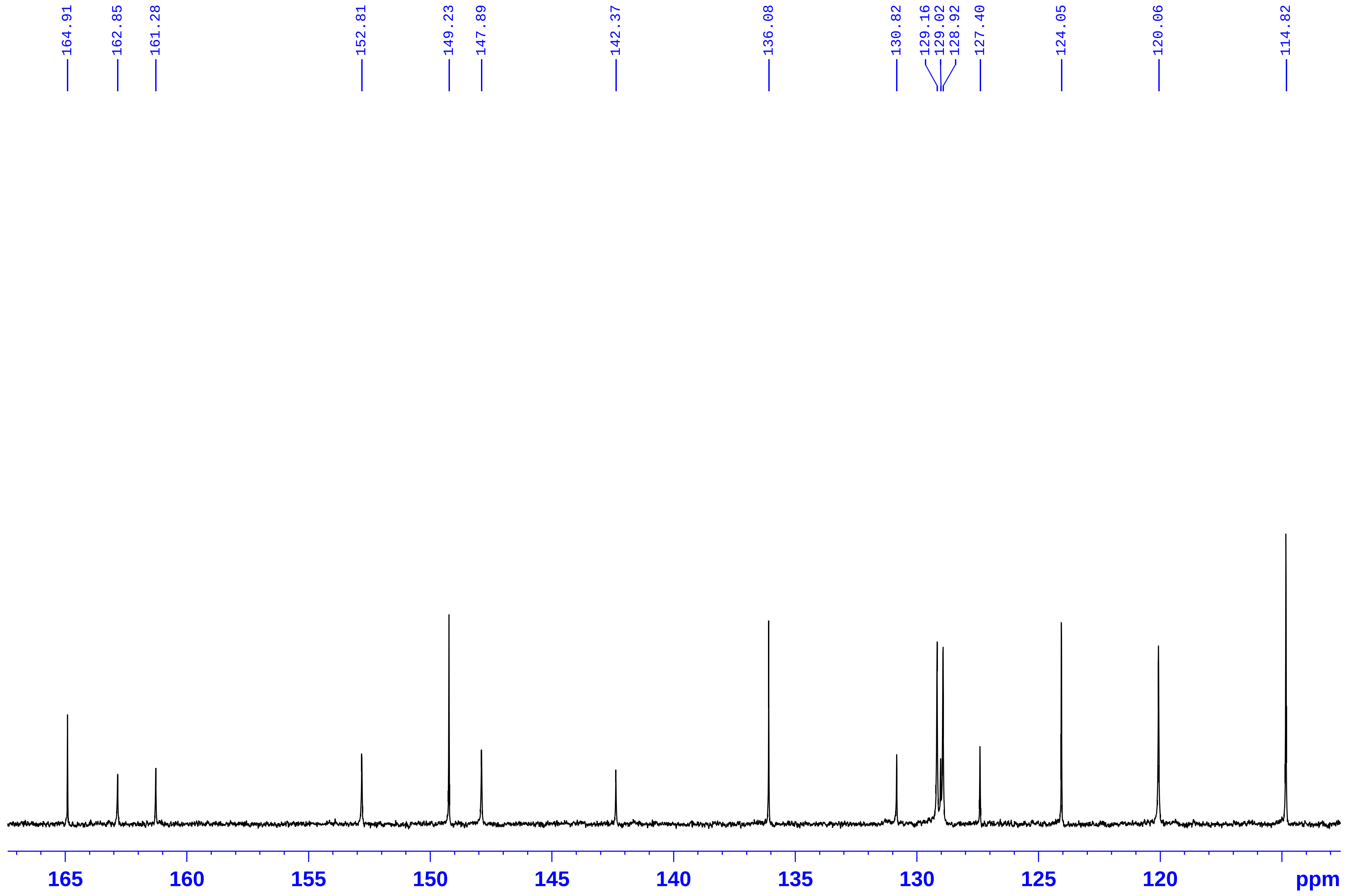

Mohamed khalifa-X7-carbon-ES

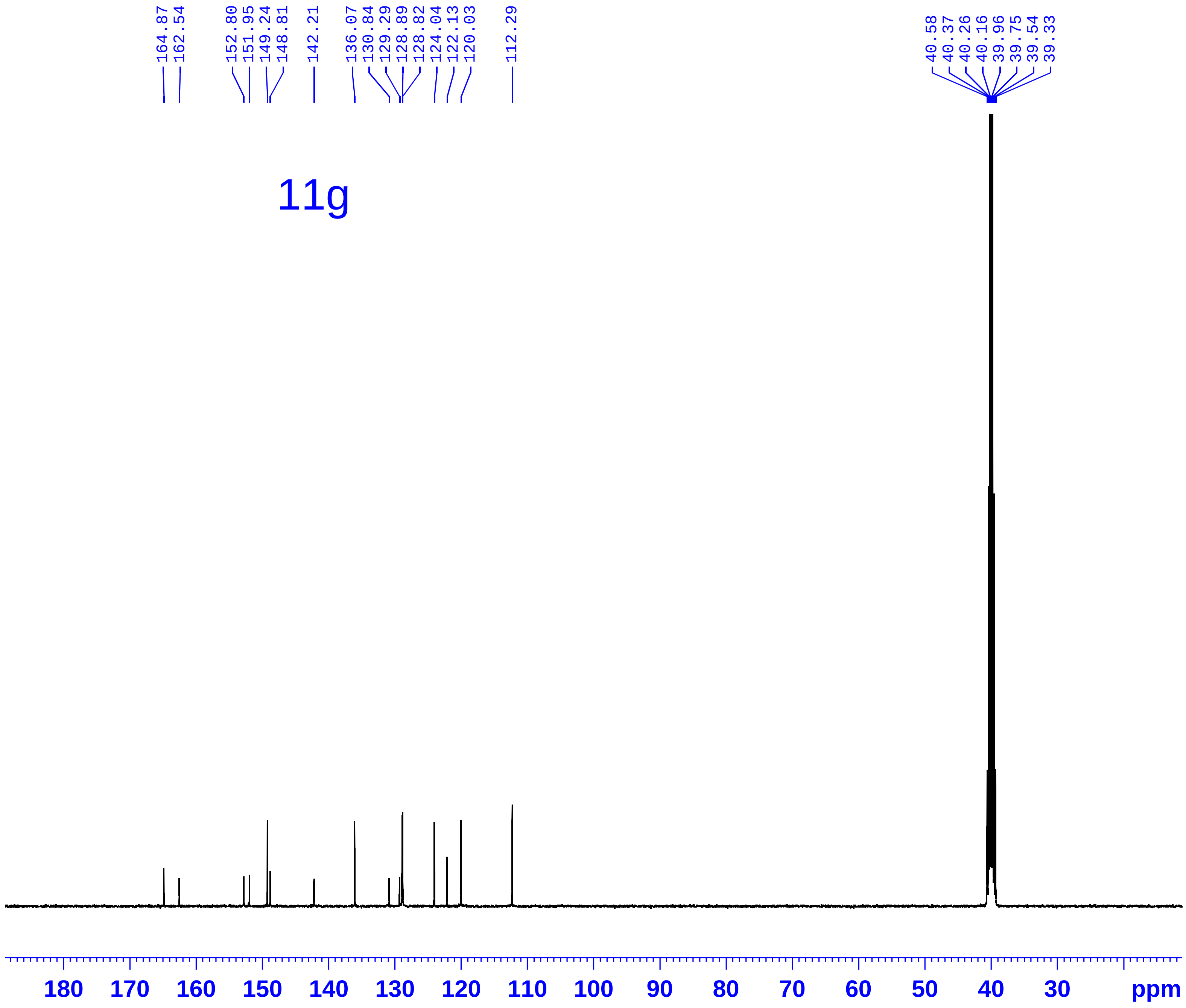

11g

Faculty of Pharmacy

```
Current Data Parameters
NAME      Mohamed khalifa-X7-carbon-ES
EXPNO                     10
PROCNO                     1

F2 - Acquisition Parameters
Date_               20201202
Time                   21.27 h
INSTRUM                spect
PROBHD     Z108618_0945 (
PULPROG              zgpg30
TD                     65536
SOLVENT                 DMSO
NS                      2100
DS                         4
SWH               24038.461 Hz
FIDRES             0.733596 Hz
AQ                1.3631488 sec
RG                    197.77
DW                    20.800 usec
DE                      6.50 usec
TE                     293.7 K
D1                2.00000000 sec
D11               0.03000000 sec
TD0                        1
SFO1             100.6404331 MHz
NUC1                     13C
P1                     10.00 usec
PLW1             47.00000000 W
SFO2             400.2016008 MHz
NUC2                      1H
CPDPRG[2             waltz16
PCPD2                  90.00 usec
PLW2             13.00000000 W
PLW12             0.29249999 W
PLW13             0.14713000 W

F2 - Processing parameters
SI                     32768
SF               100.6303700 MHz
WDW                       EM
SSB        0
LB                      1.00 Hz
GB         0
PC                      1.40
```

Mohamed khalifa-X7-carbon-ES

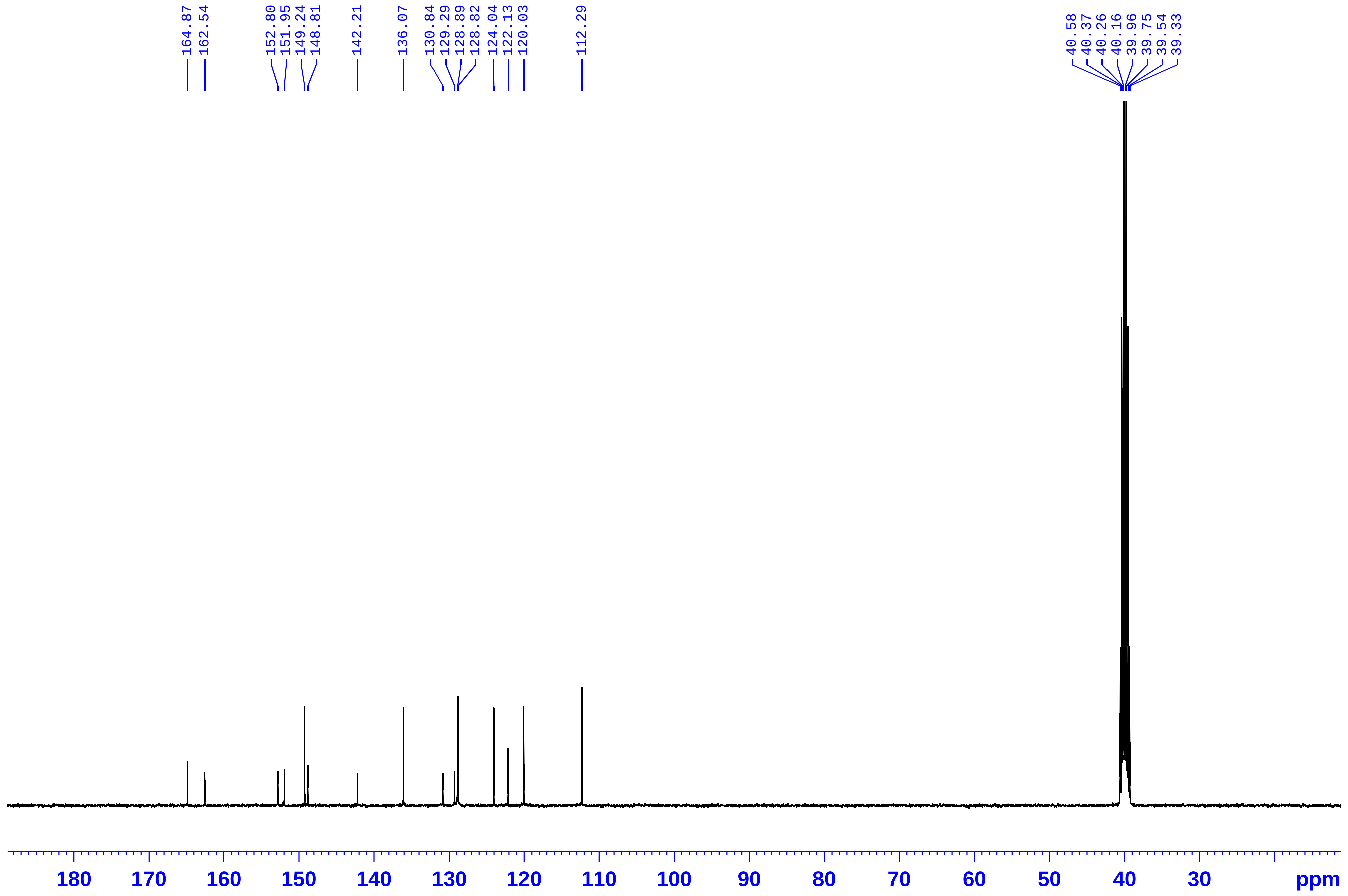

Mohamed khalifa-X7-carbon-ES

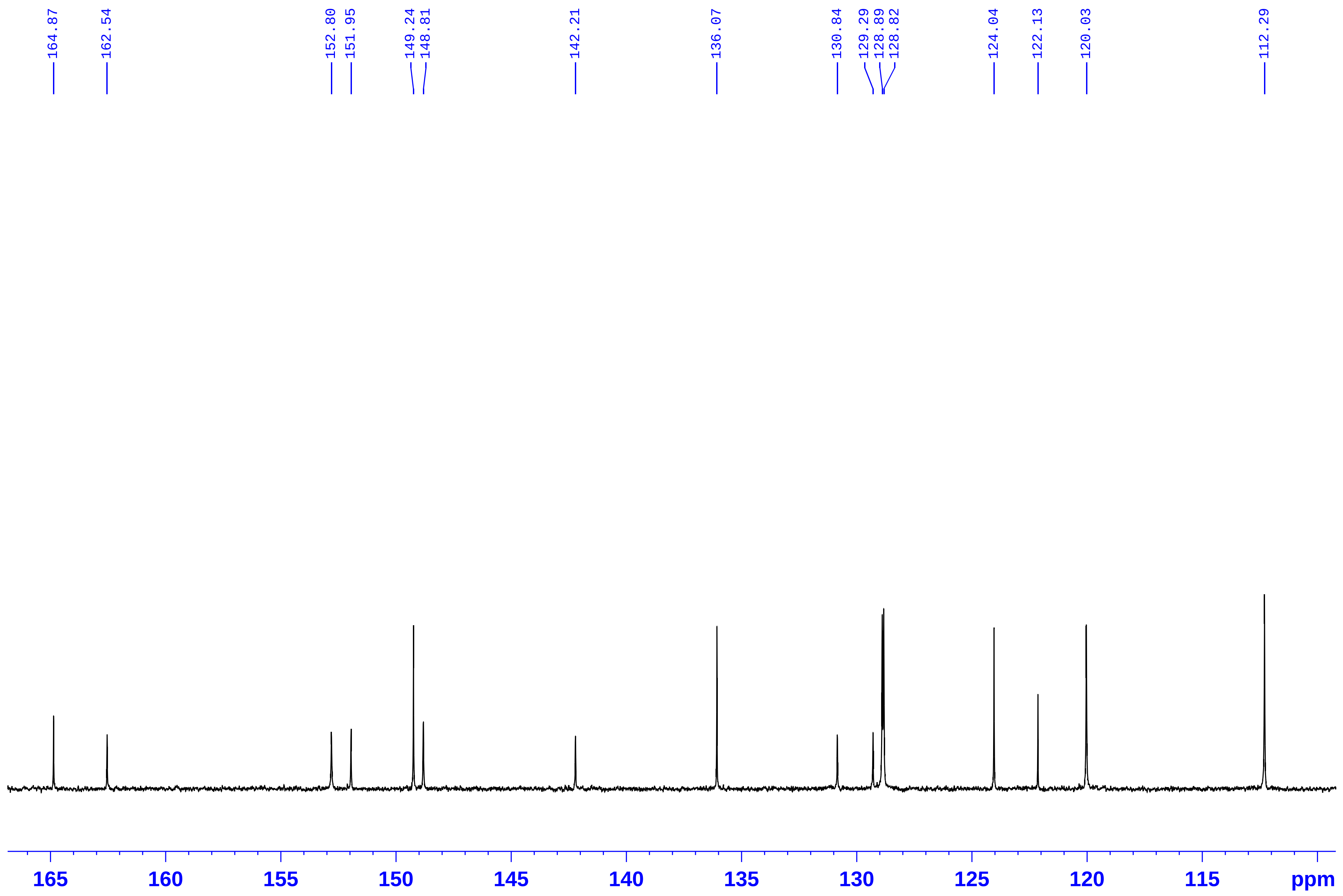

Mohamed khalifa-X8-carbon-ES

11h

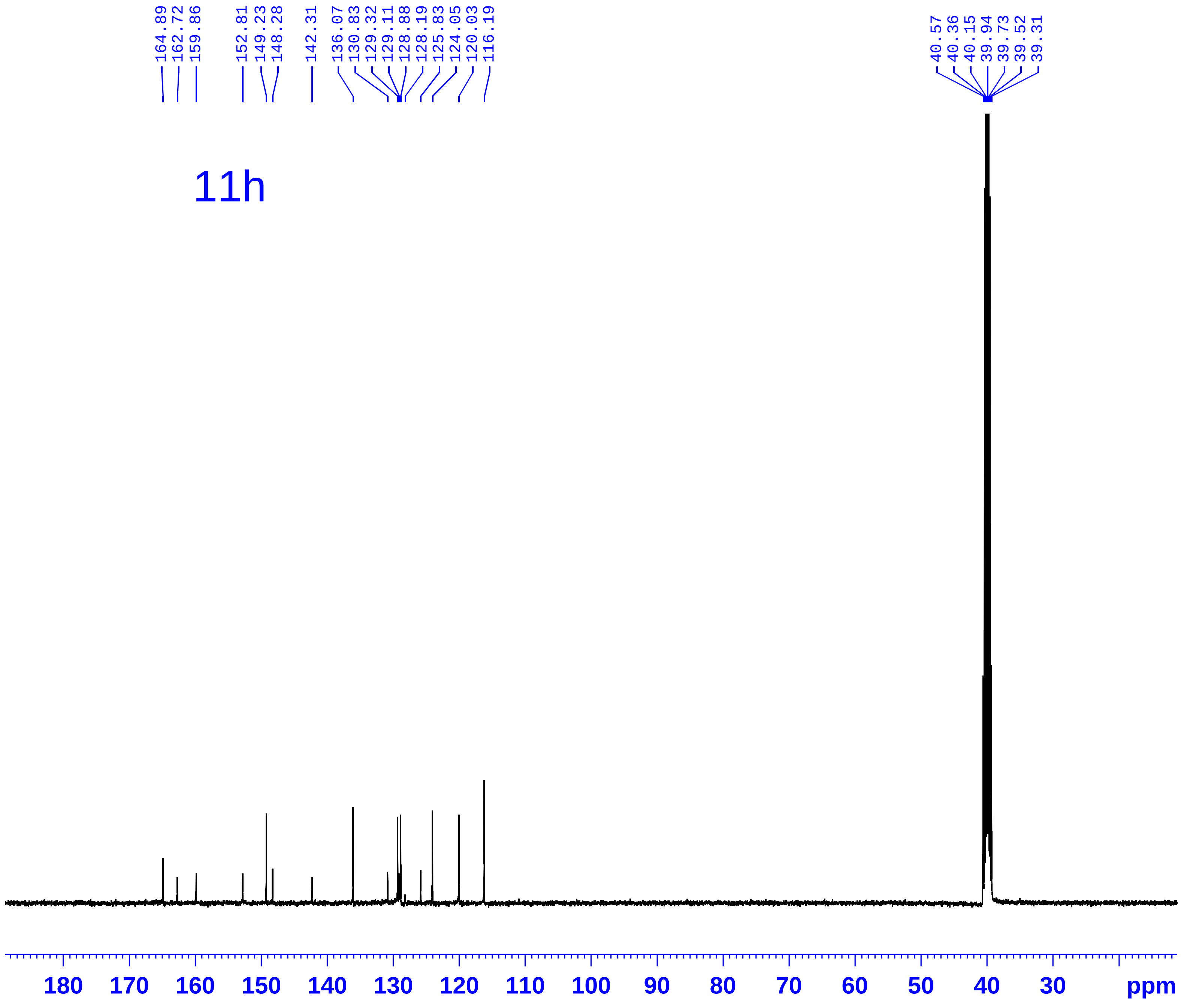

Faculty of Pharmacy

```
Current Data Parameters
NAME      Mohamed khalifa-X8-carbon-ES
EXPNO                    10
PROCNO                    1

F2 - Acquisition Parameters
Date_              20201202
Time                  23.36 h
INSTRUM               spect
PROBHD    Z108618_0945 (
PULPROG             zgpg30
TD                   65536
SOLVENT               DMSO
NS                    2200
DS                       4
SWH              24038.461 Hz
FIDRES            0.733596 Hz
AQ               1.3631488 sec
RG                  197.77
DW                  20.800 usec
DE                    6.50 usec
TE                   293.6 K
D1              2.00000000 sec
D11             0.03000000 sec
TD0                      1
SFO1           100.6404331 MHz
NUC1                   13C
P1                   10.00 usec
PLW1           47.00000000 W
SFO2           400.2016008 MHz
NUC2                    1H
CPDPRG[2           waltz16
PCPD2                90.00 usec
PLW2           13.00000000 W
PLW12           0.29249999 W
PLW13           0.14713000 W

F2 - Processing parameters
SI                   32768
SF             100.6303700 MHz
WDW                     EM
SSB       0
LB                    1.00 Hz
GB        0
PC                    1.40
```

Mohamed khalifa-X8-carbon-ES

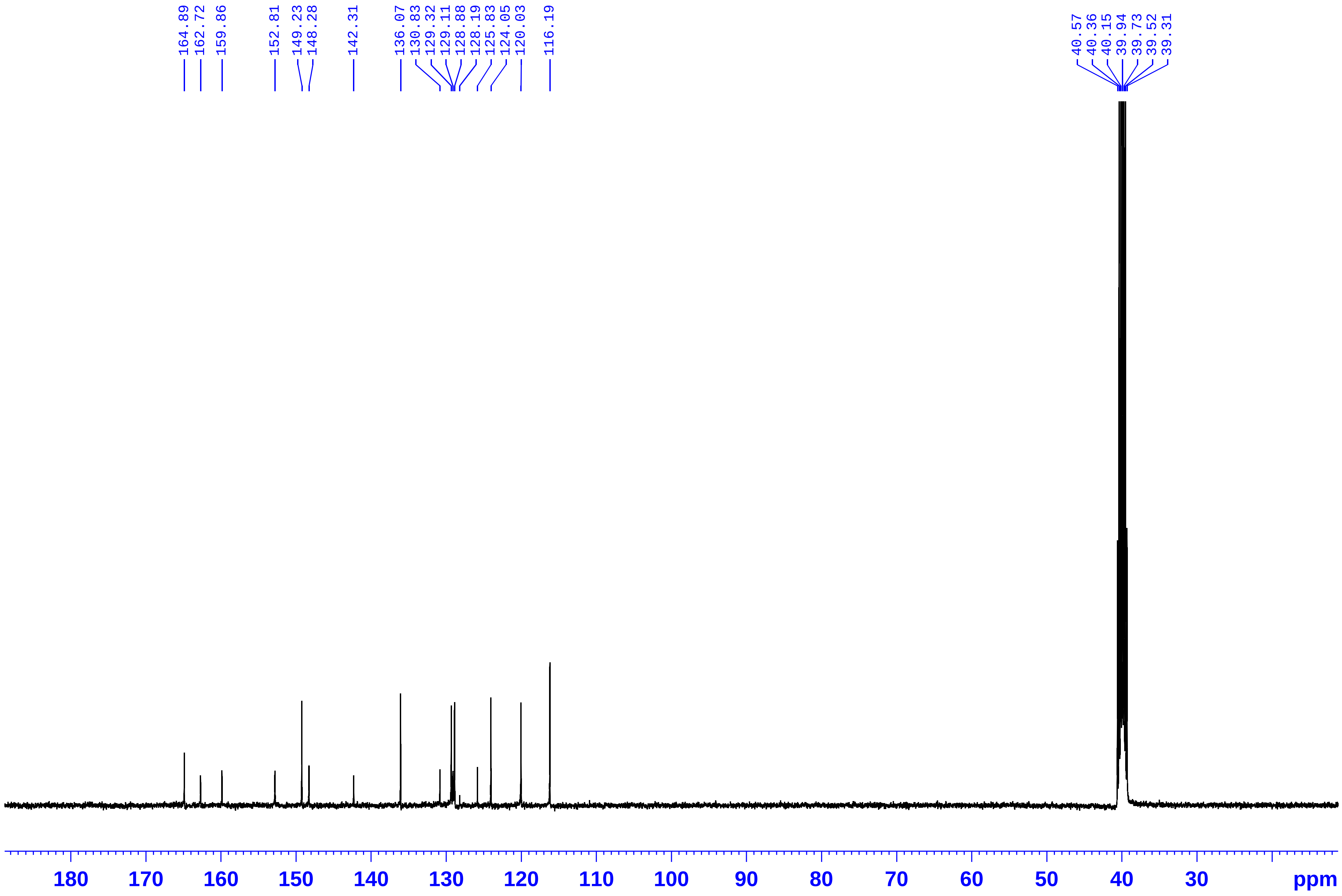

Mohamed khalifa-X8-carbon-ES

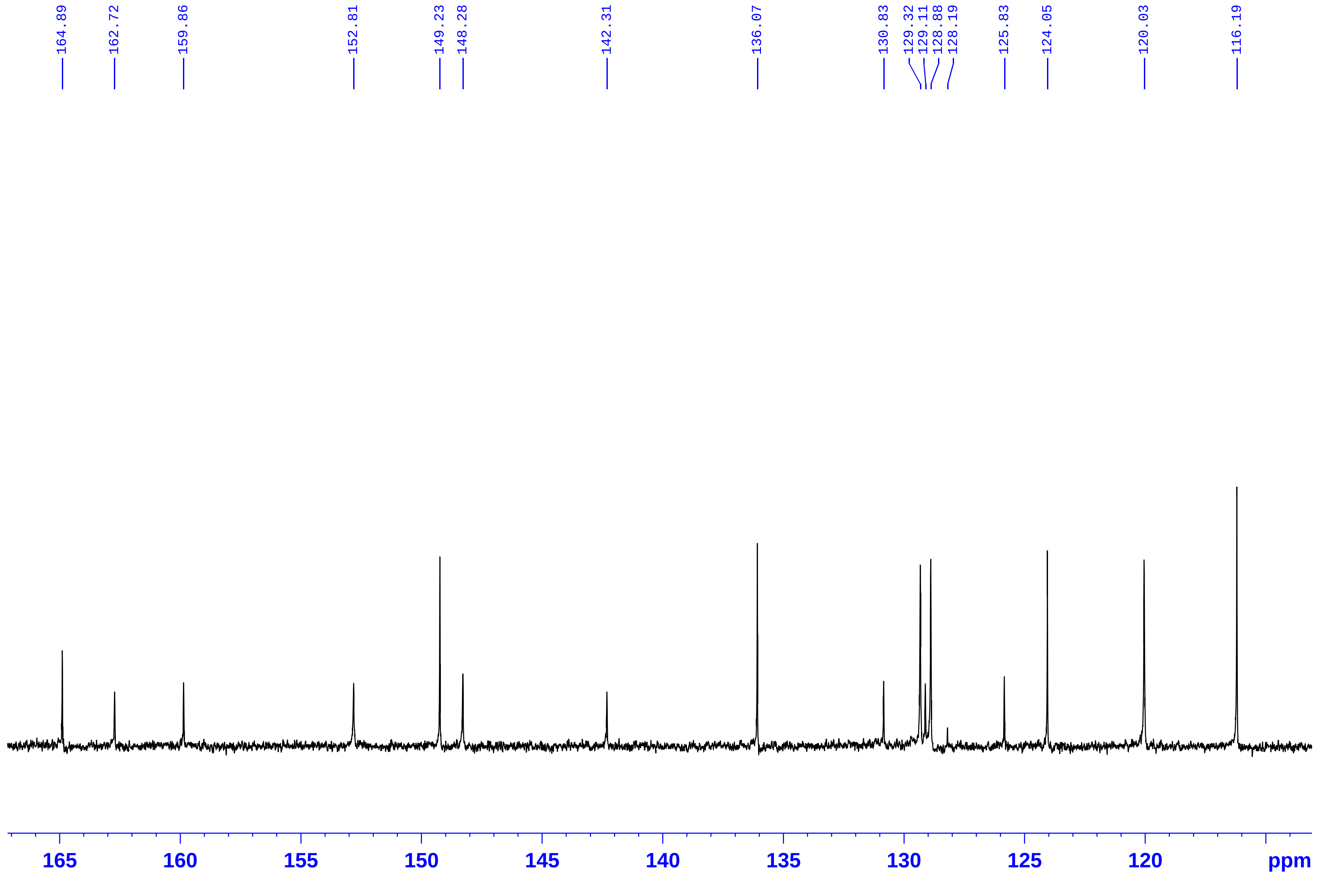

جامعة القاهرة

كلية الصيدلة

Microanalytical Unit-FOPCU

وحدة التحاليل الدقيقة

معمل الأشعة تحت الحمراء

IR Lab

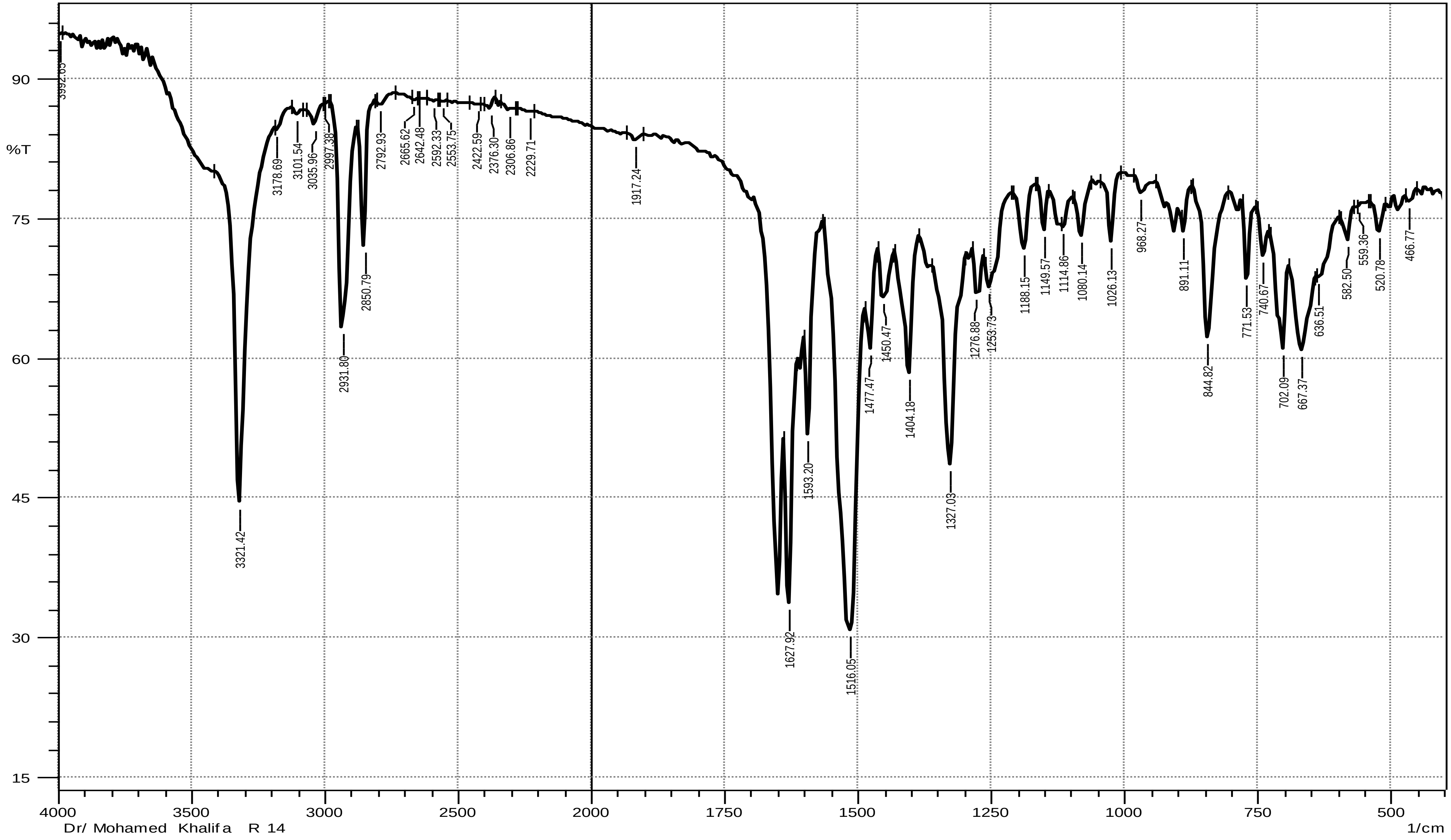

Dr/ Mohamed Khalifa 5

جامعة القاهرة

كلية الصيدلة

MAU

Microanalytical Unit-FOPCU

وحدة التحاليل الدقيقة

معمل الأشعة تحت الحمراء

IR Lab

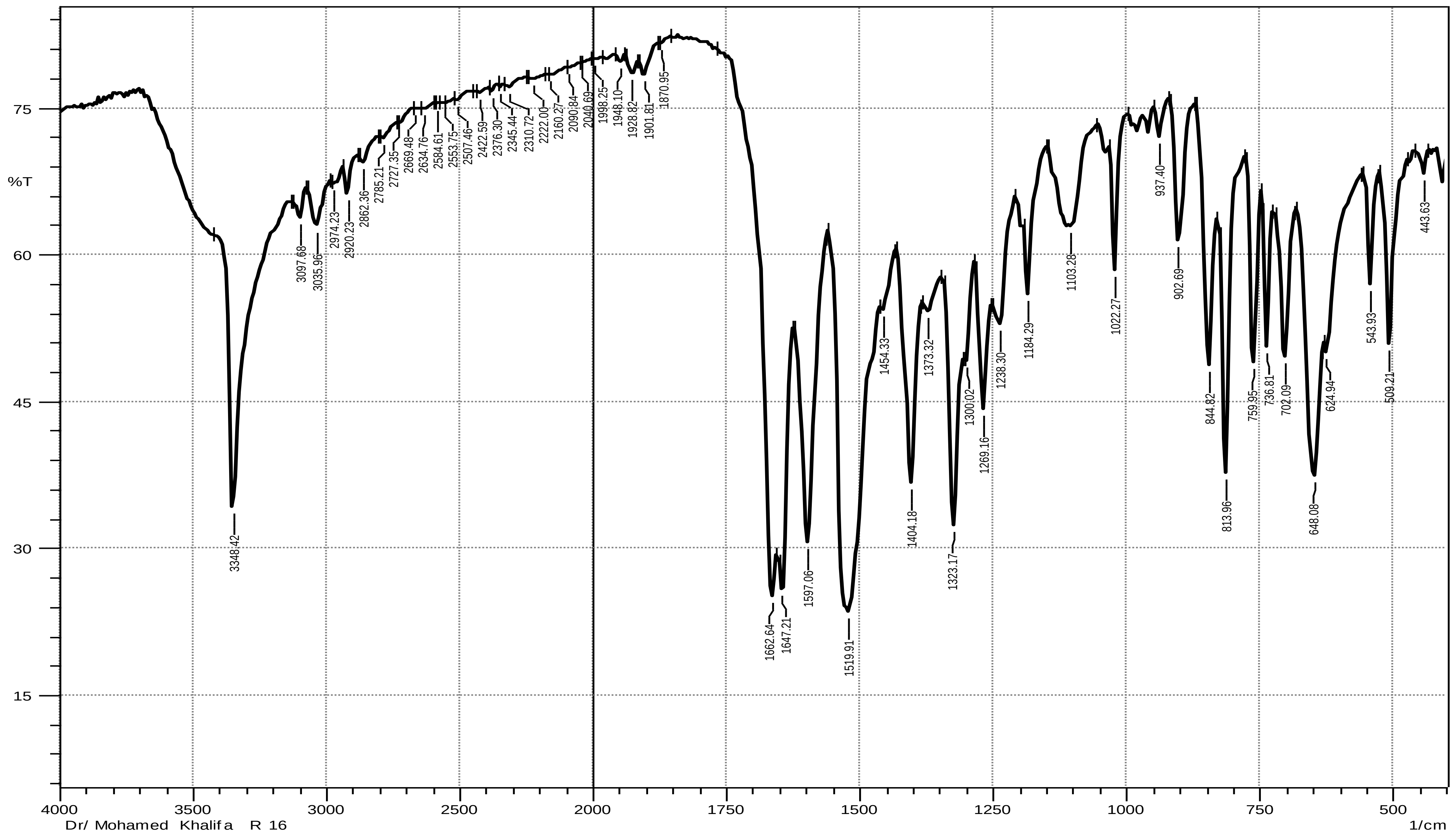

Dr/ Mohamed Khalifa 7j

جامعة القاهرة
كلية الصيدلة
MAU
Microanalytical Unit-FOPCU
وحدة التحاليل الدقيقة
معمل الأشعة تحت الحمراء
IR Lab

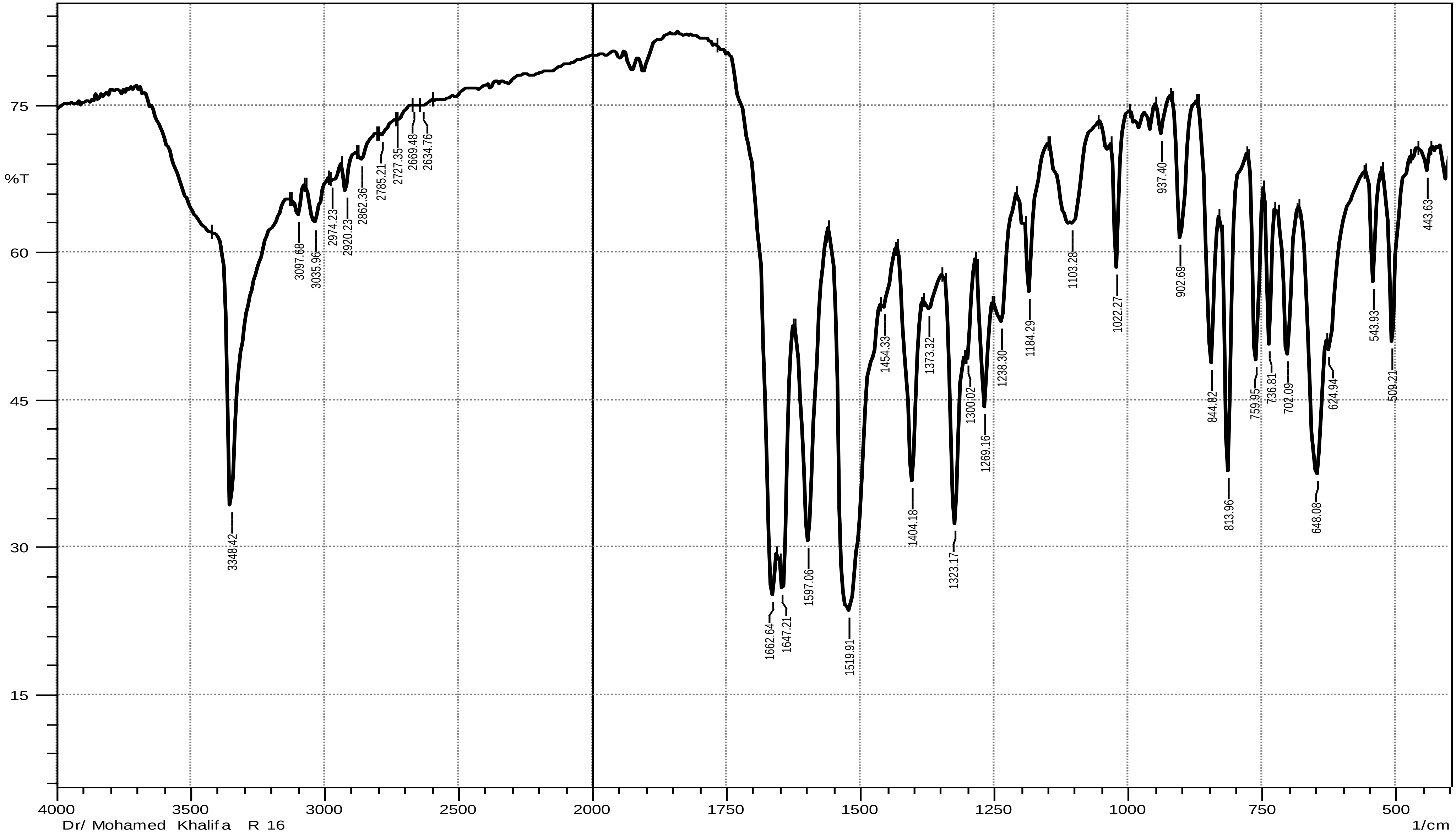

Dr/ Mohamed Khalifa 7j

جامعة القاهرة

كلية الصيدلة

MAU

Microanalytical Unit-FOPCU

وحدة التحاليل الدقيقة

معمل الأشعة تحت الحمراء

IR Lab

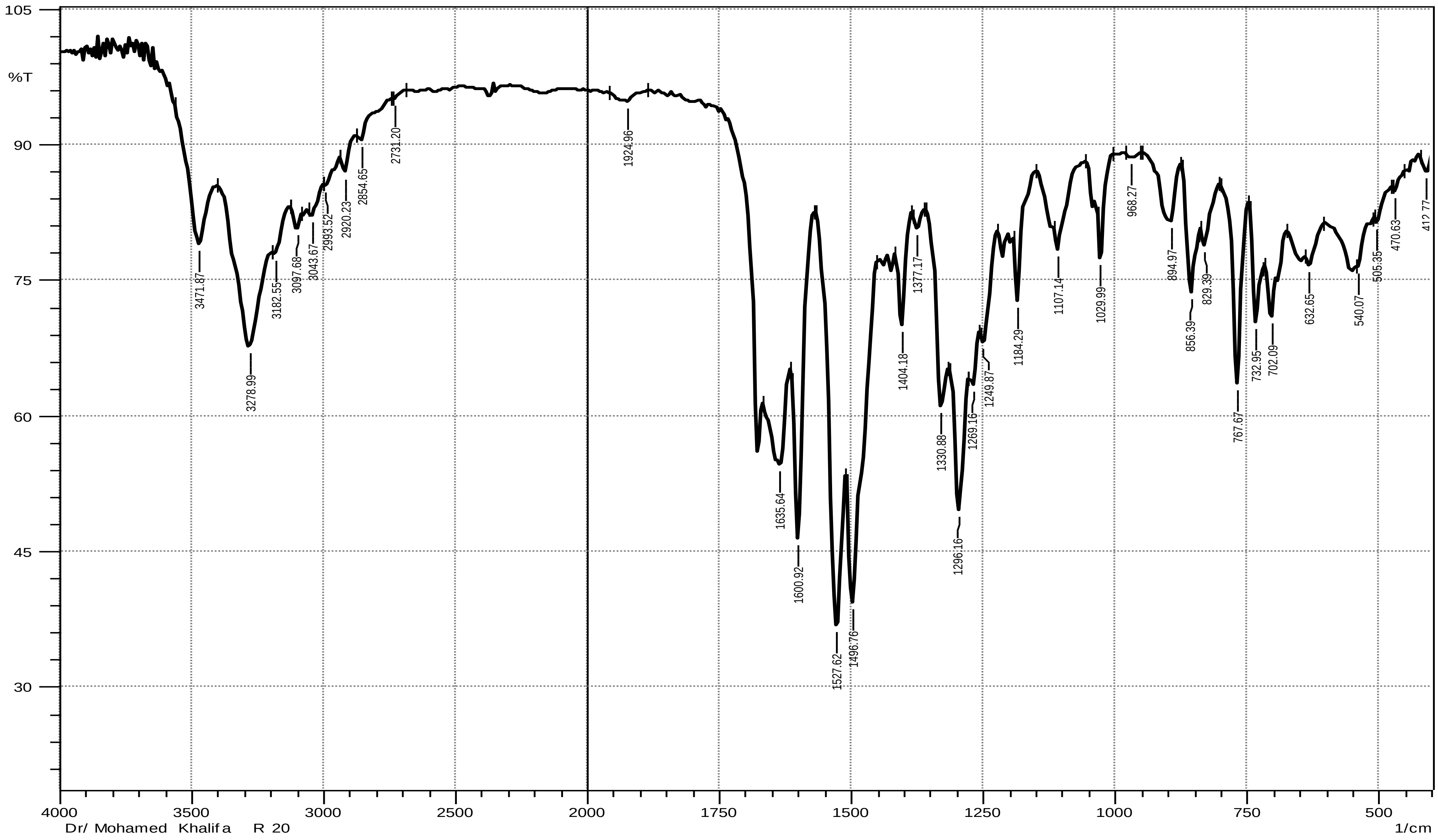

Dr/ Mohamed Khalifa 7k

جامعة القاهرة
كلية الصيدلة

MAU
Microanalytical Unit-FOPCU
وحدة التحاليل الدقيقة
معمل الأشعة تحت الحمراء

IR Lab

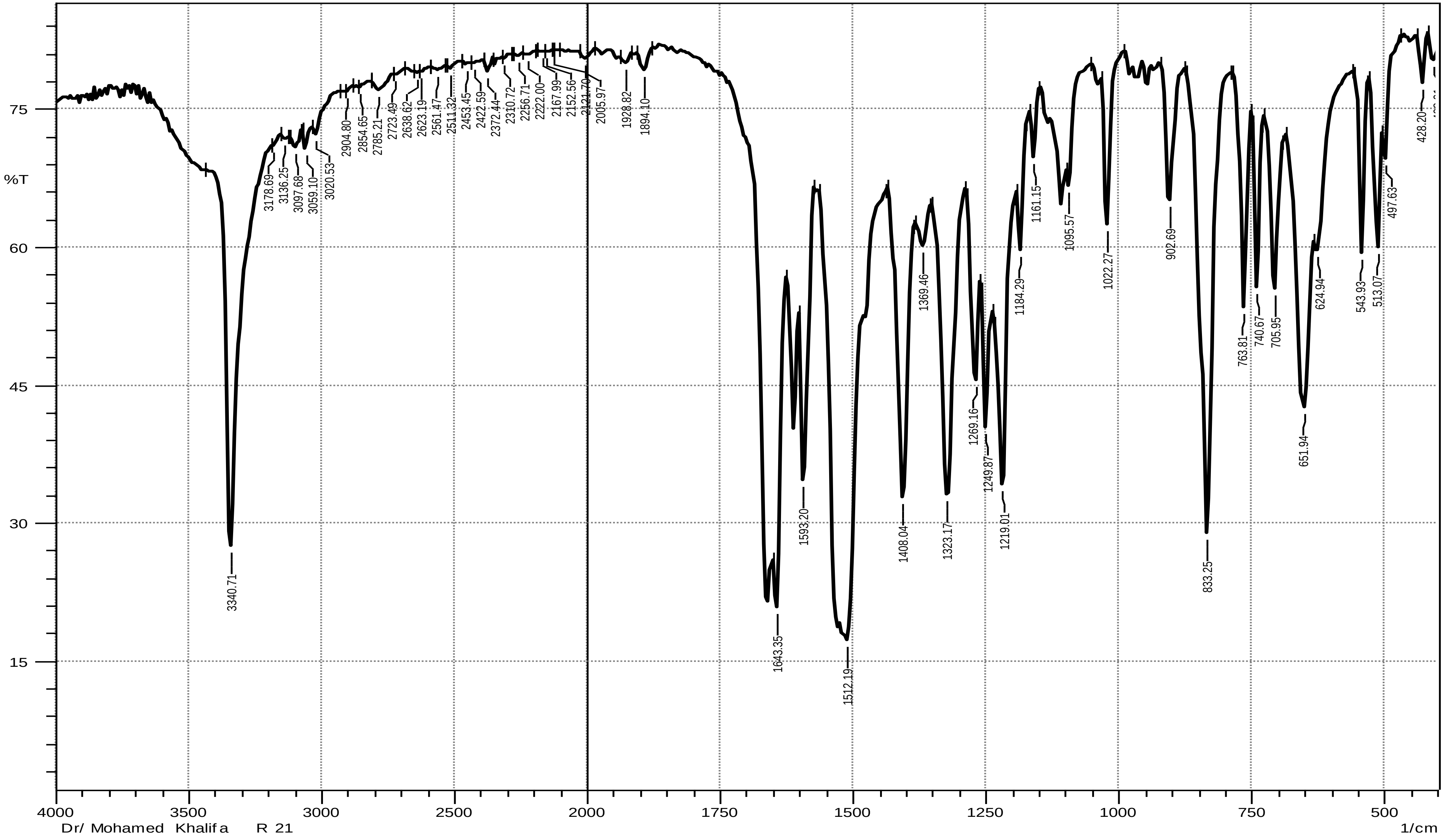

Dr/ Mohamed Khalifa 7l

جامعة القاهرة
كلية الصيدلة

MAU
Microanalytical Unit-FOPCU
وحدة التحاليل الدقيقة
معمل الأشعة تحت الحمراء

IR Lab

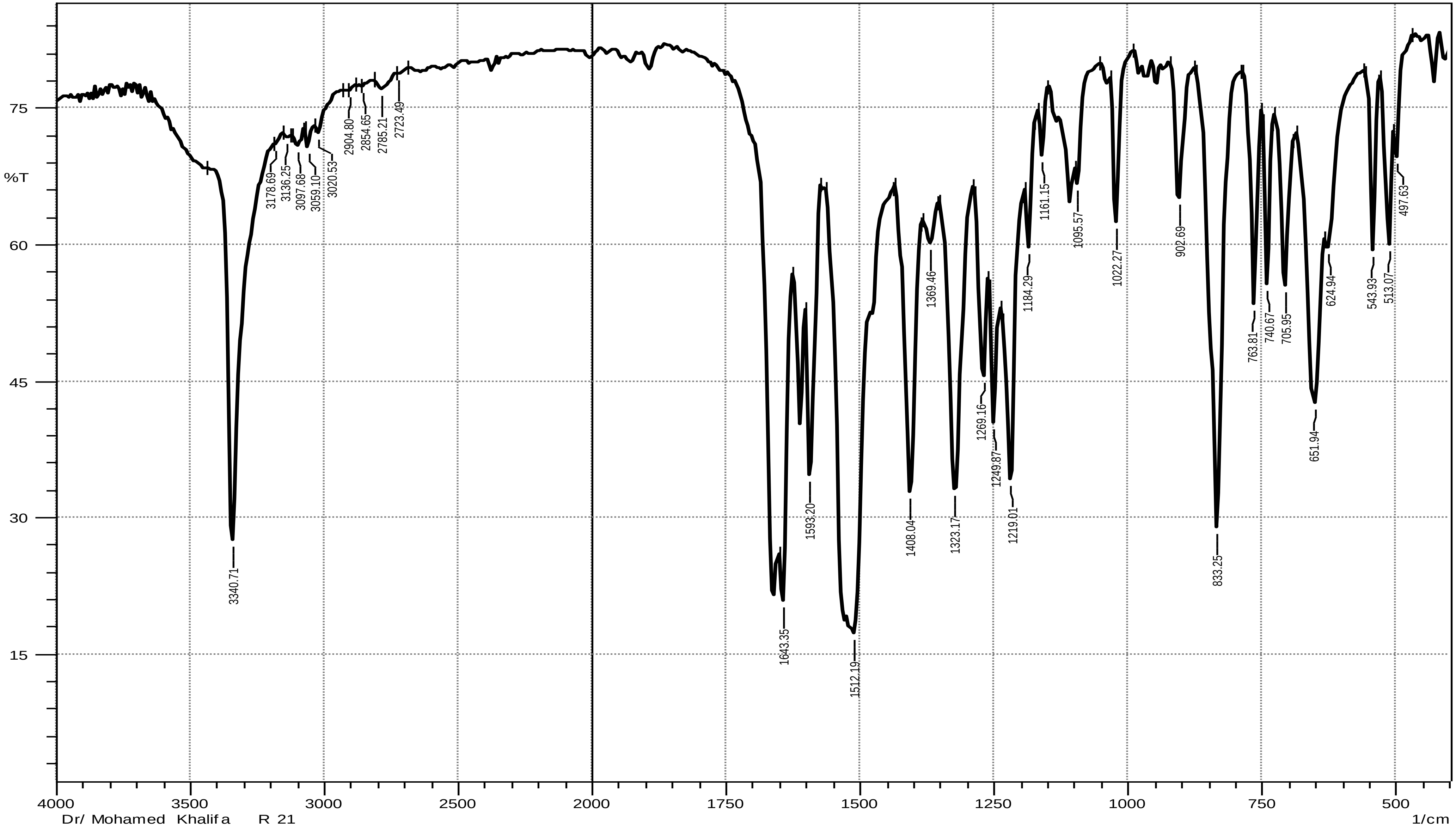

Dr/ Mohamed Khalifa 7l

جامعة القاهرة
كلية الصيدلة
MAU
Microanalytical Unit-FOPCU
وحدة التحاليل الدقيقة
معمل الأشعة تحت الحمراء
IR Lab

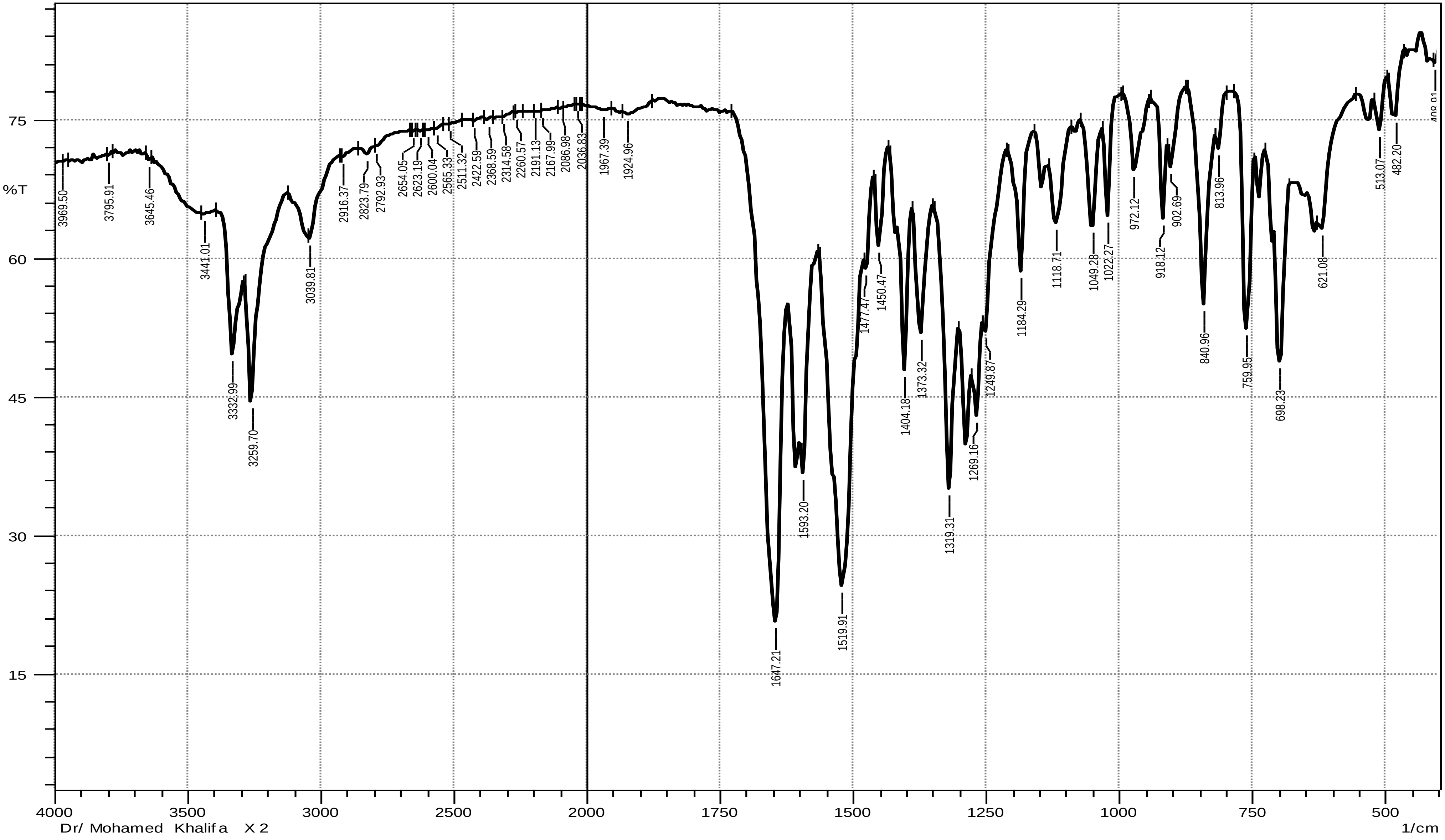

Dr/ Mohamed Khalifa 11b

جامعة القاهرة
كلية الصيدلة
MAU
Microanalytical Unit-FOPCU
وحدة التحاليل الدقيقة
معمل الأشعة تحت الحمراء
IR Lab

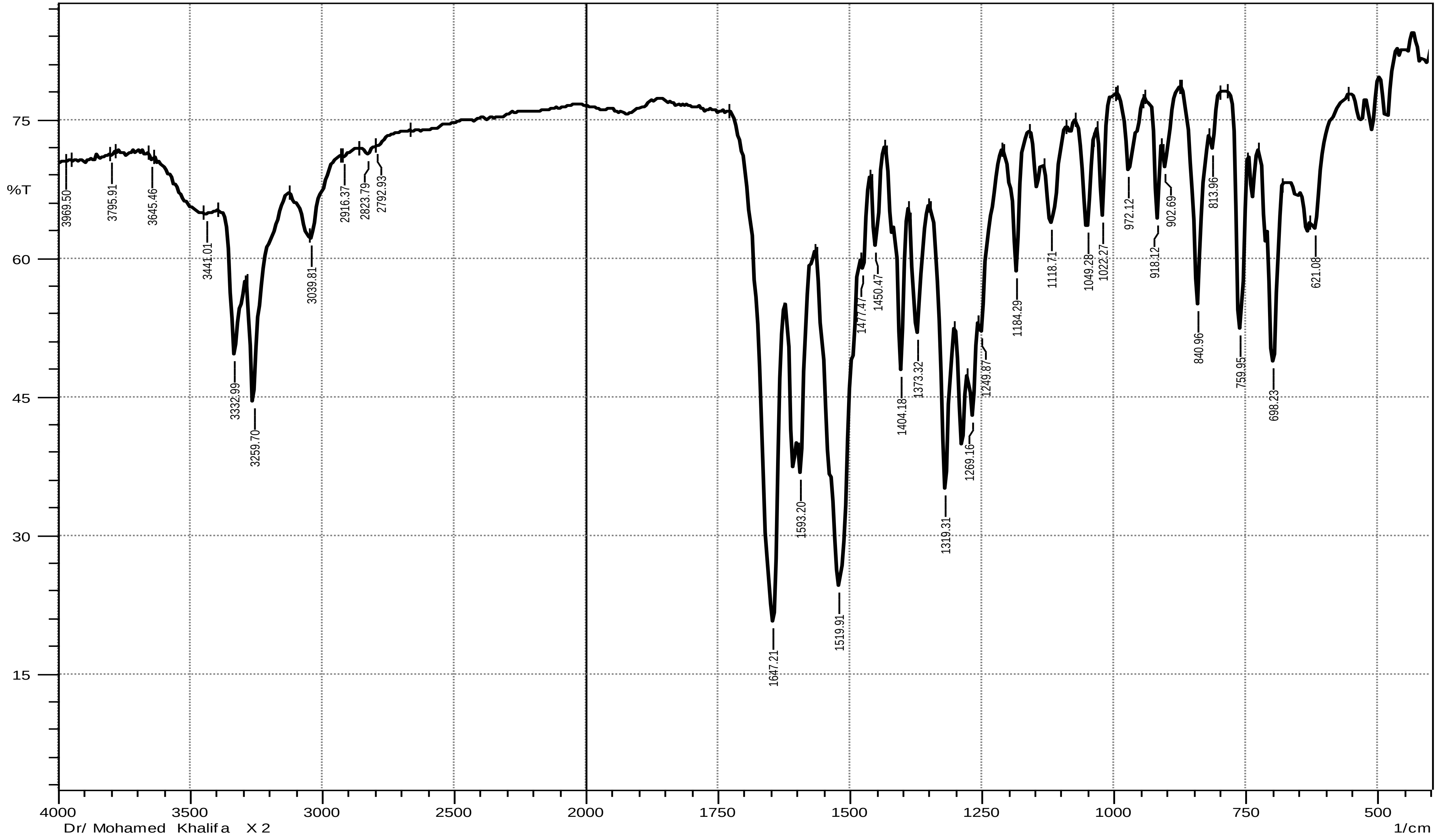

Dr/ Mohamed Khalifa 11b.

جامعة القاهرة
كلية الصيدلة
MAU
Microanalytical Unit-FOPCU
وحدة التحاليل الدقيقة
معمل الأشعة تحت الحمراء
IR Lab

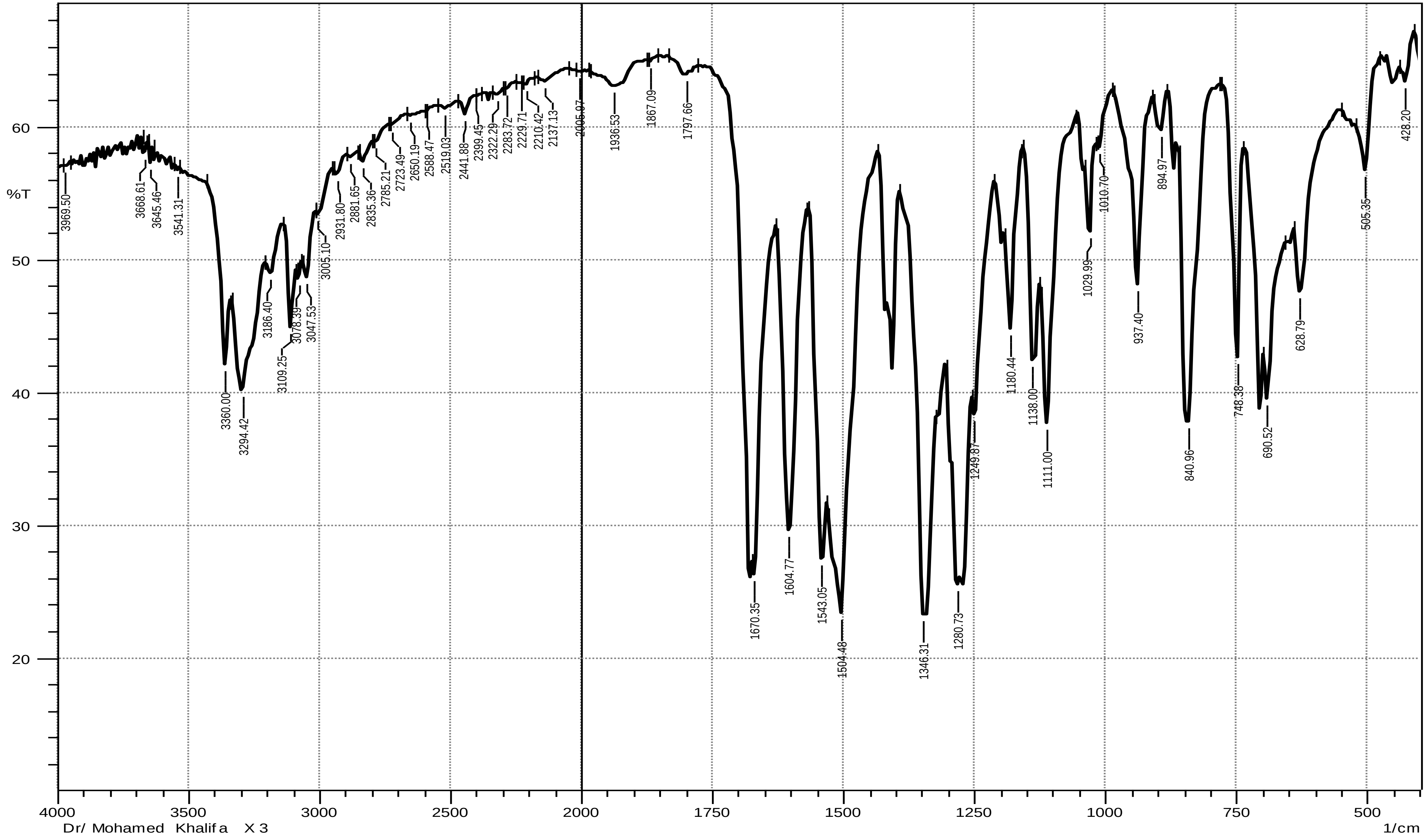

Dr/ Mohamed Khalifa 11c

جامعة القاهرة
كلية الصيدلة

MAU
Microanalytical Unit-FOPCU
وحدة التحاليل الدقيقة
معمل الأشعة تحت الحمراء

IR Lab

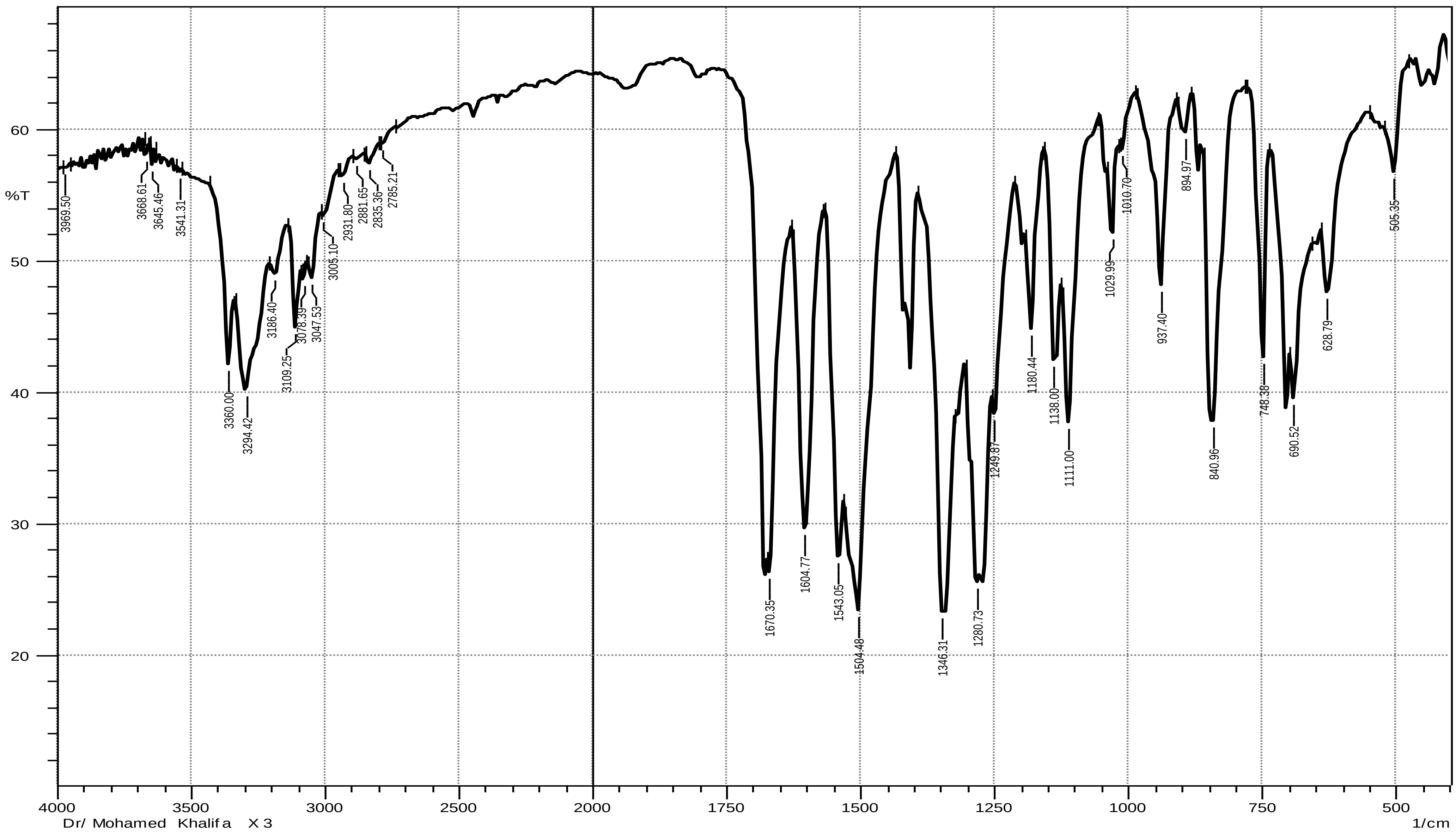

Dr/ Mohamed Khalifa 11c

جامعة القاهرة

MAU

Microanalytical Unit-FOPCU

وحدة التحاليل الدقيقة

معمل الأشعة تحت الحمراء

كلية الصيدلة

IR Lab

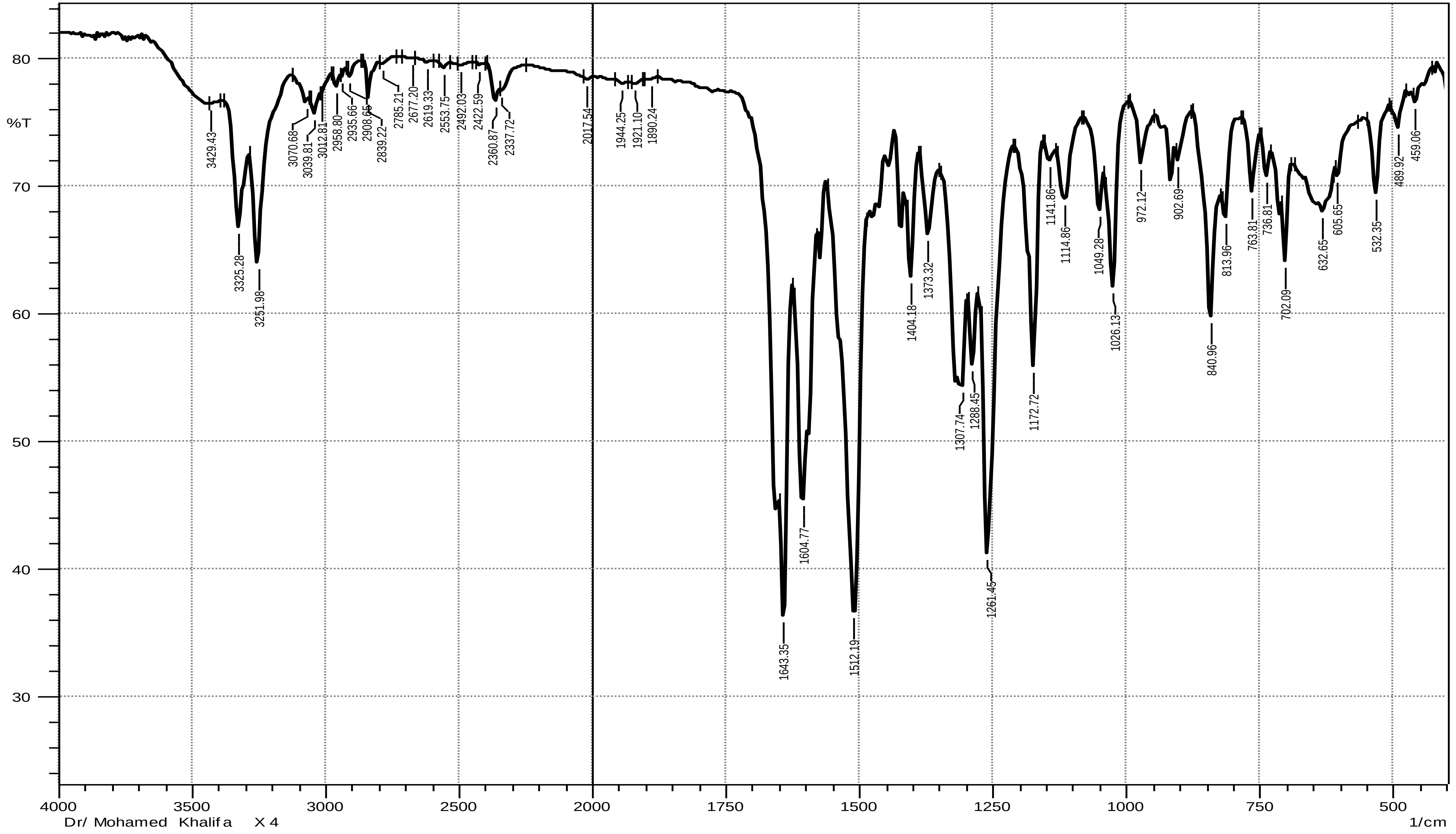

Dr/ Mohamed Khalifa 11d

جامعة القاهرة
كلية الصيدلة

MAU
Microanalytical Unit-FOPCU
وحدة التحاليل الدقيقة
معمل الأشعة تحت الحمراء

IR Lab

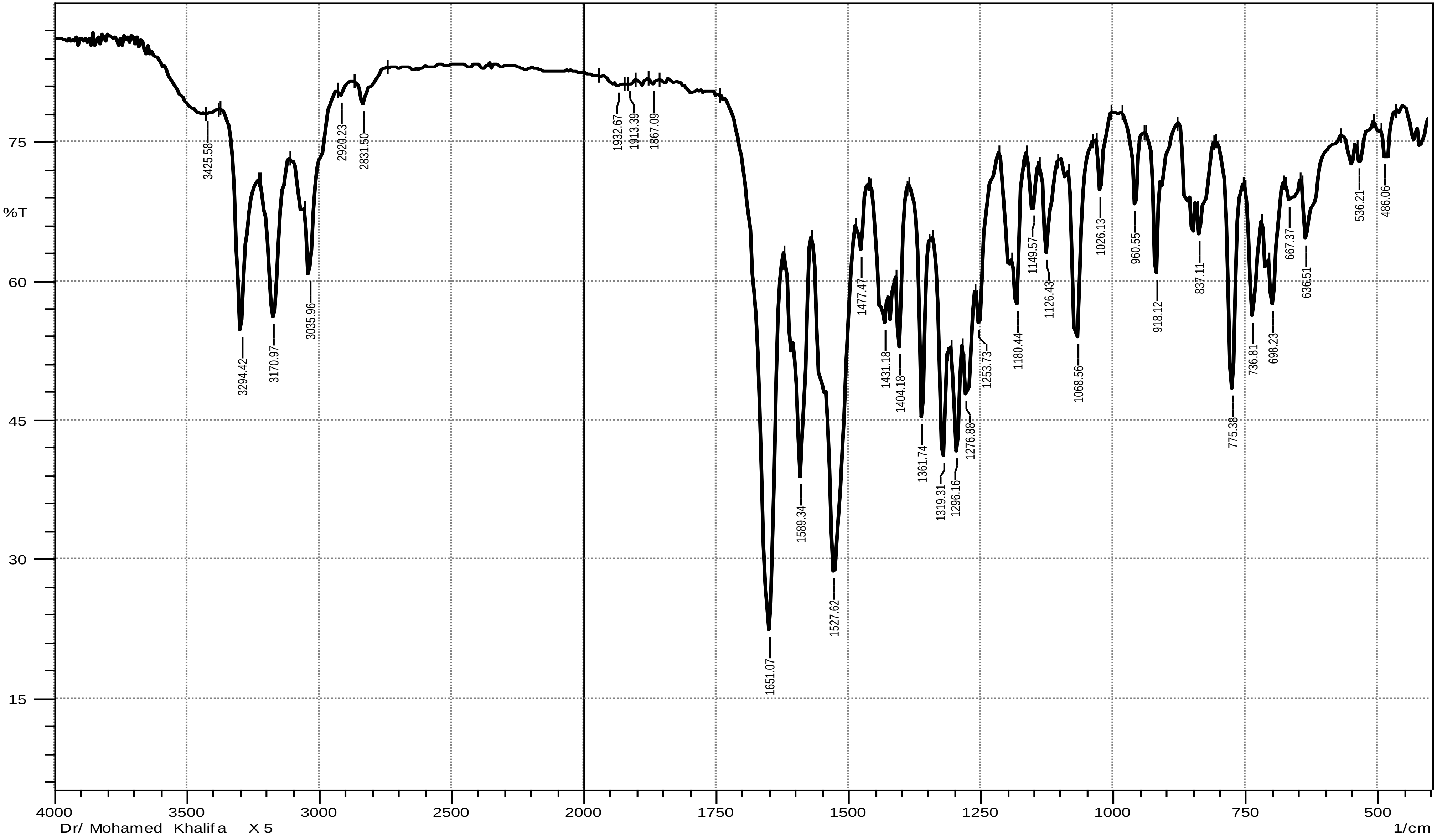

Dr/ Mohamed Khalifa 11e

جامعة القاهرة
كلية الصيدلة

MAU
Microanalytical Unit-FOPCU
وحدة التحاليل الدقيقة
معمل الأشعة تحت الحمراء

IR Lab

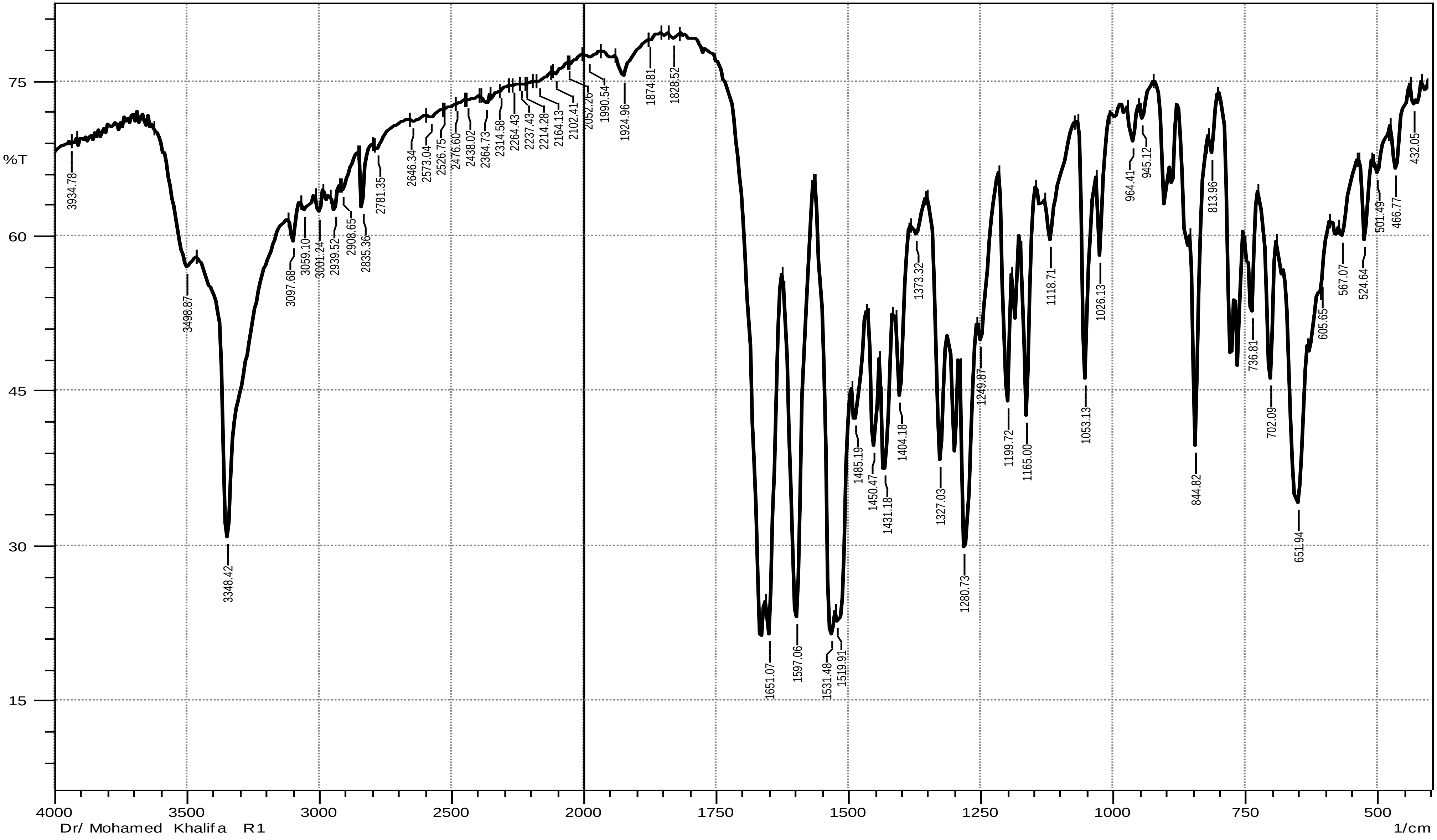

Dr/ Mohamed Khalifa 7a

جامعة القاهرة

كلية الصيدلة

MAU

Microanalytical Unit-FOPCU

وحدة التحاليل الدقيقة

معمل الأشعة تحت الحمراء

IR Lab

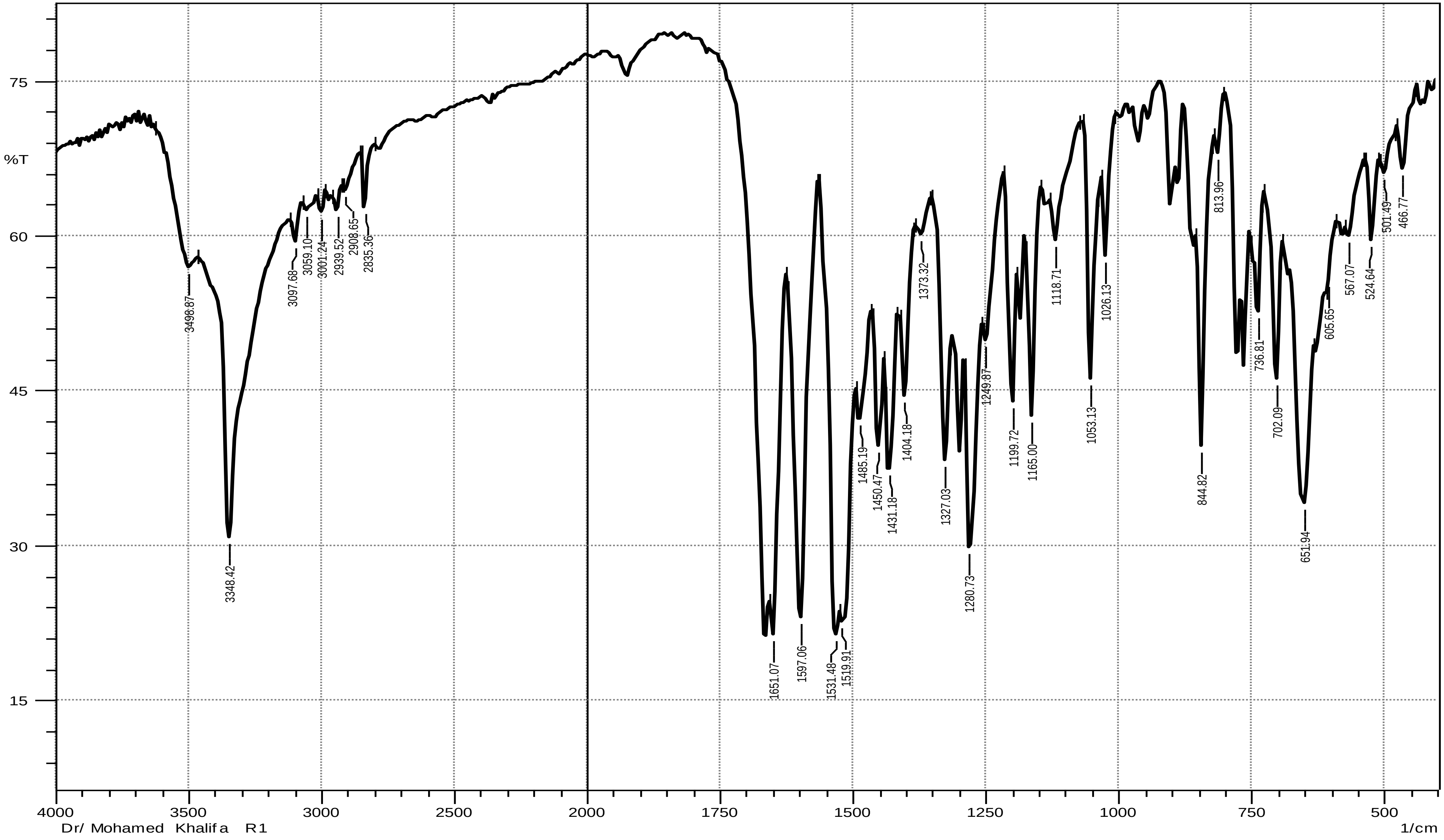

Dr/ Mohamed Khalifa 7a

جامعة القاهرة
كلية الصيدلة

MAU
Microanalytical Unit-FOPCU
وحدة التحاليل الدقيقة
معمل الأشعة تحت الحمراء

IR Lab

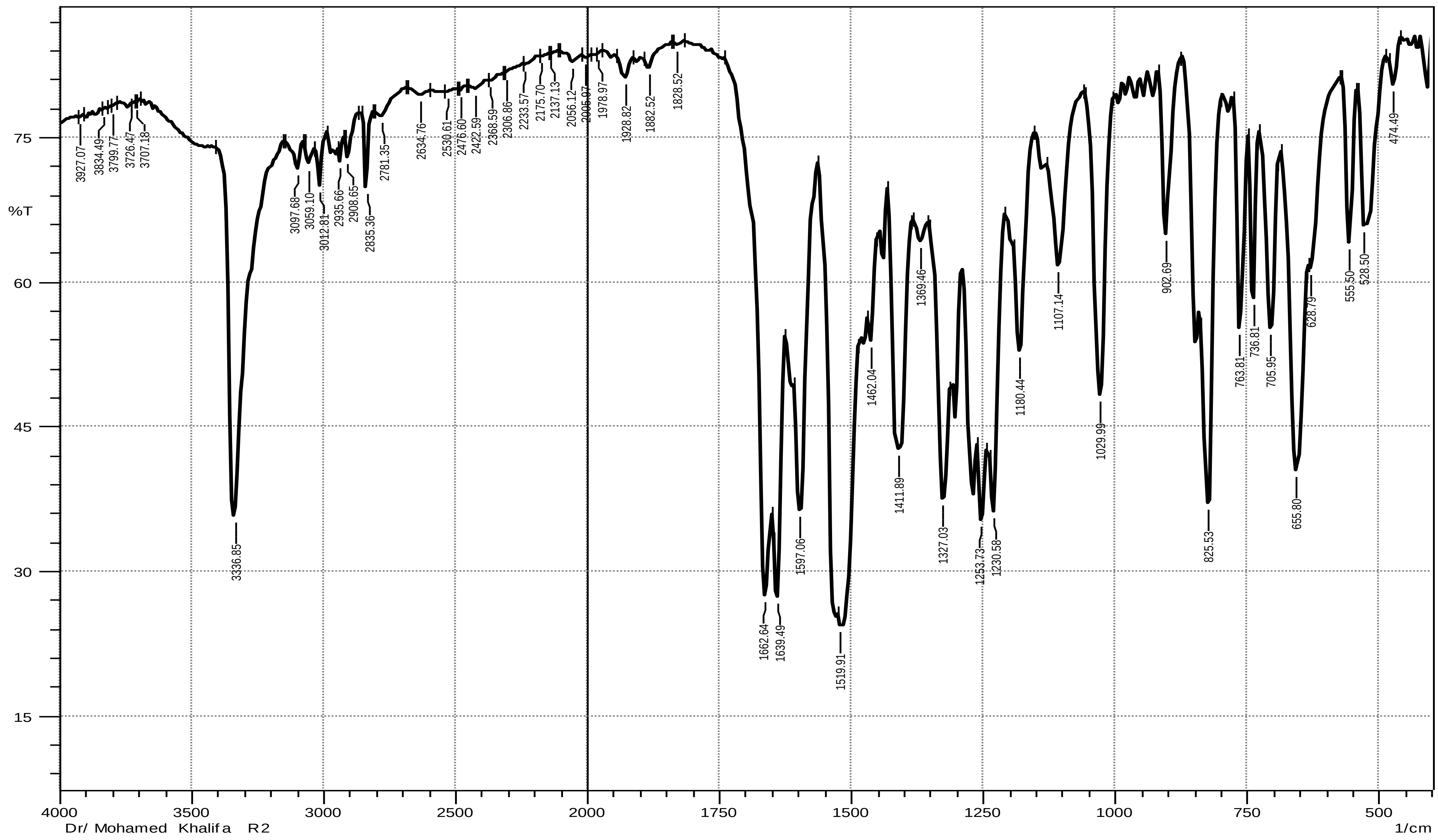

Dr/ Mohamed Khalifa 7b

جامعة القاهرة
كلية الصيدلة

MAU
Microanalytical Unit-FOPCU
وحدة التحاليل الدقيقة
معمل الأشعة تحت الحمراء

IR Lab

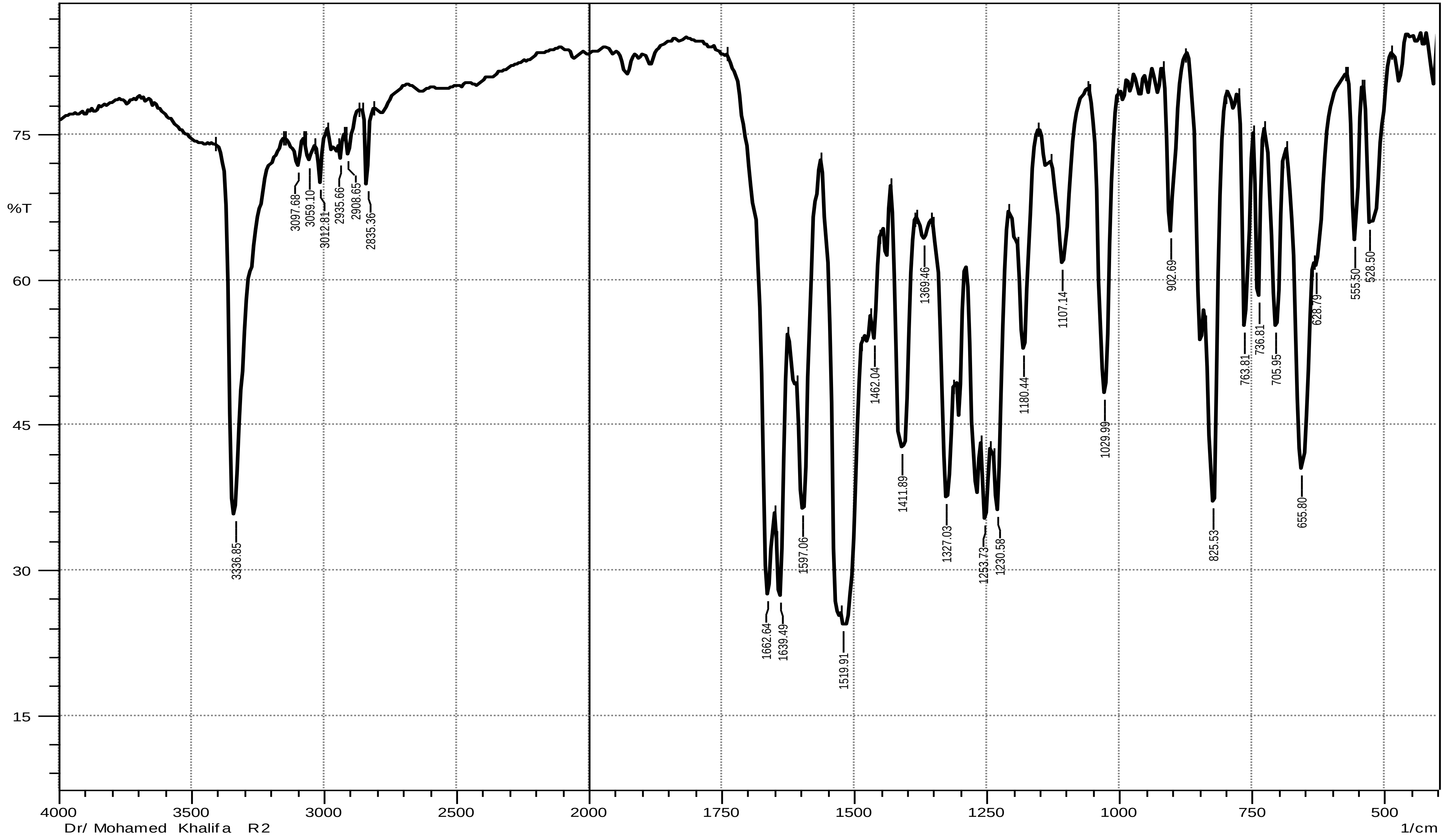

Dr/ Mohamed Khalifa 7b

Microanalytical Unit-FOPCU
وحدة التحاليل الدقيقة
معمل الأشعة تحت الحمراء

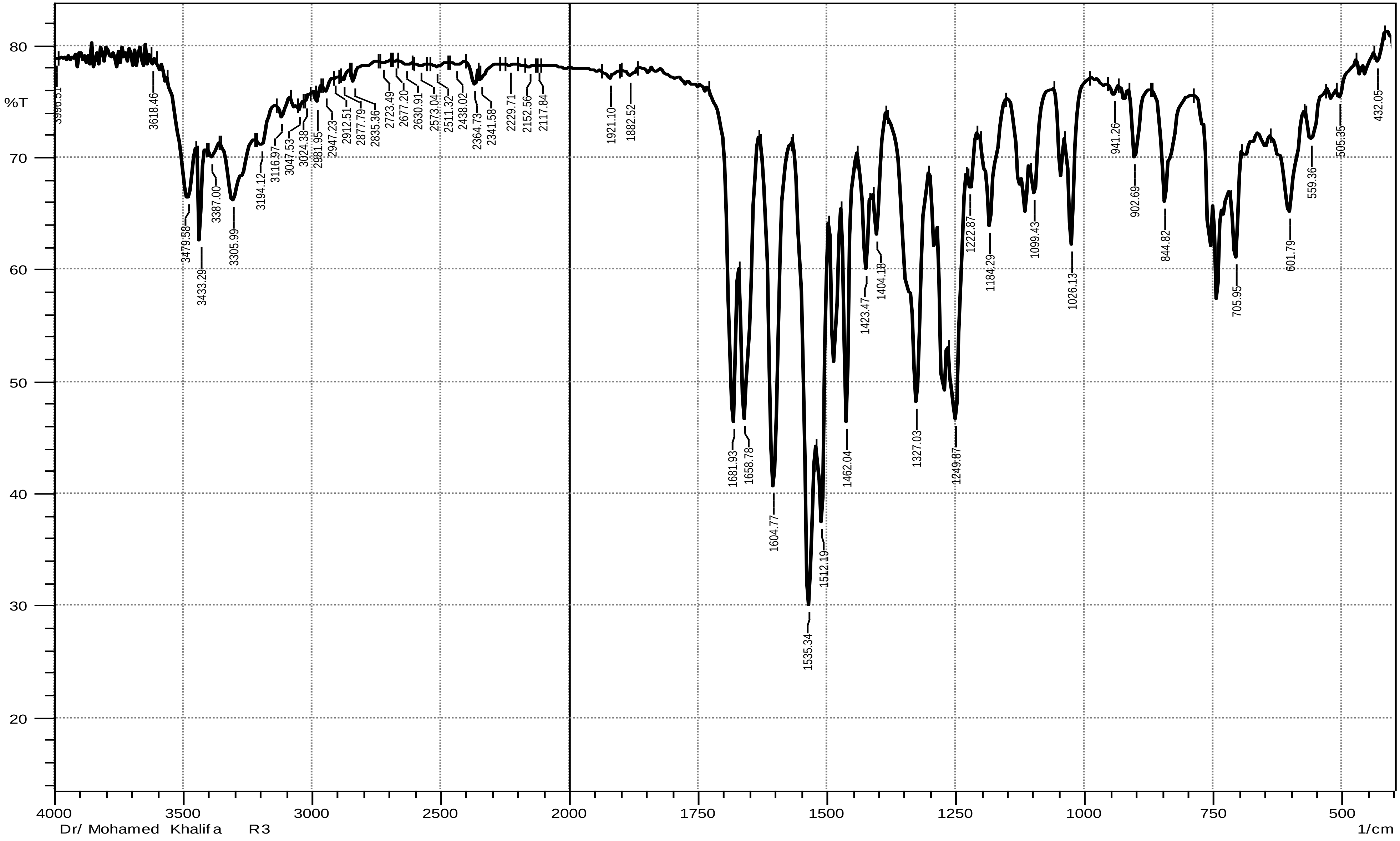

Dr/ Mohamed Khalifa 7c

جامعة القاهرة

كلية الصيدلة

MAU

Microanalytical Unit-FOPCU

وحدة التحاليل الدقيقة

معمل الأشعة تحت الحمراء

IR Lab

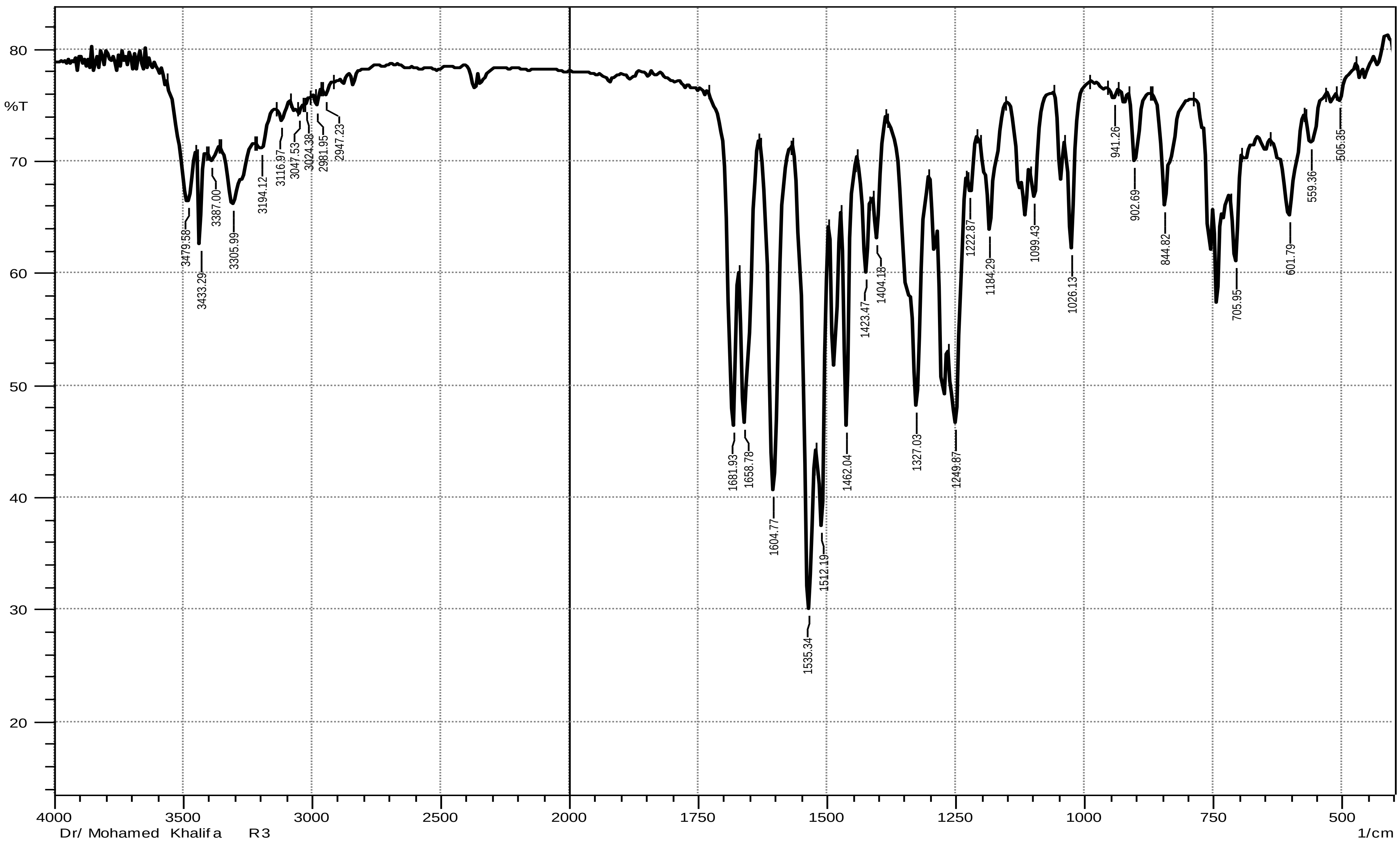

Dr/ Mohamed Khalifa 7c

جامعة القاهرة

كلية الصيدلة

MAU

Microanalytical Unit-FOPCU

وحدة التحاليل الدقيقة

معمل الأشعة تحت الحمراء

IR Lab

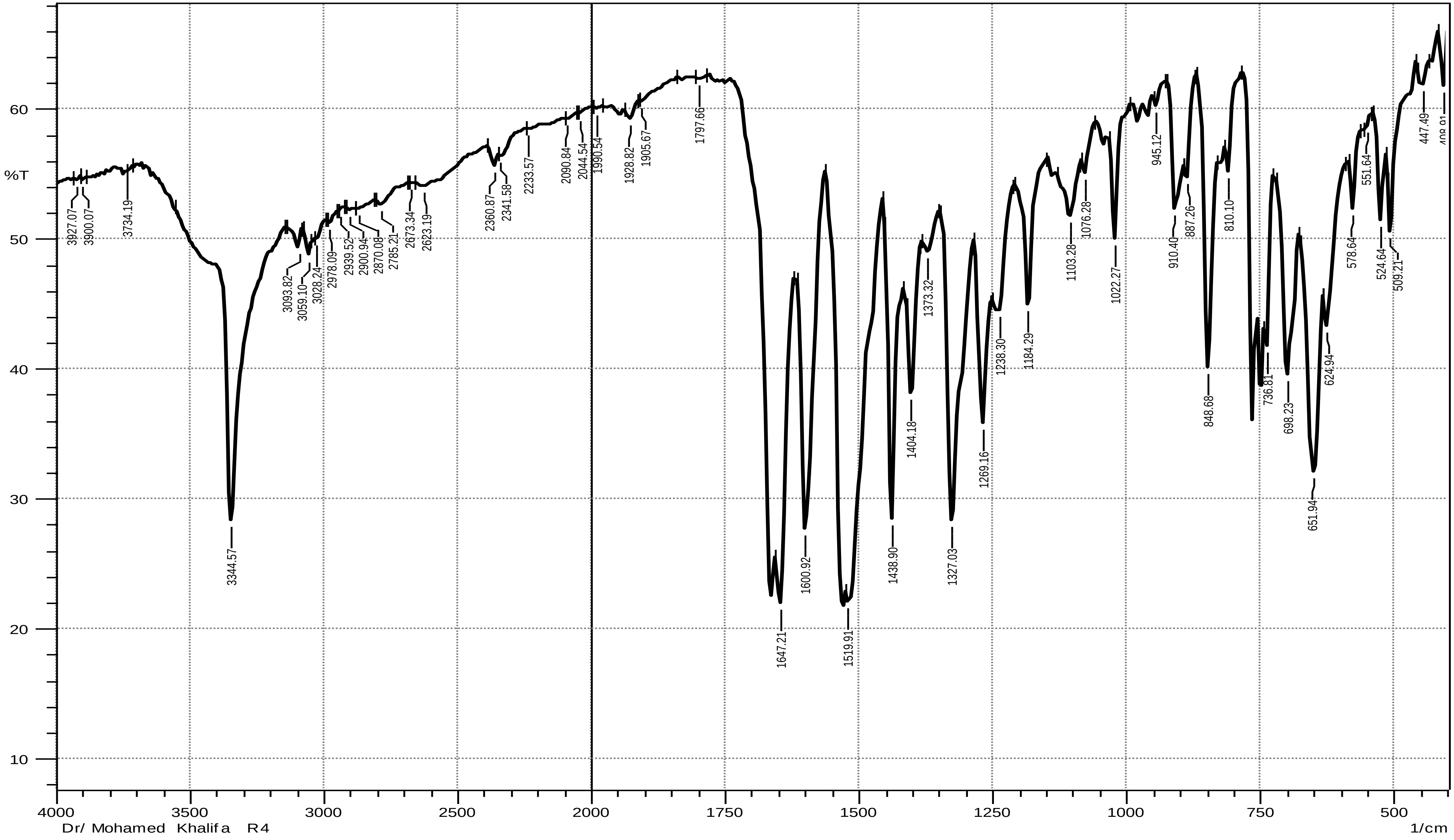

Dr/ Mohamed Khalifa 7d

جامعة القاهرة

كلية الصيدلة

MAU

Microanalytical Unit-FOPCU

وحدة التحاليل الدقيقة

معمل الأشعة تحت الحمراء

IR Lab

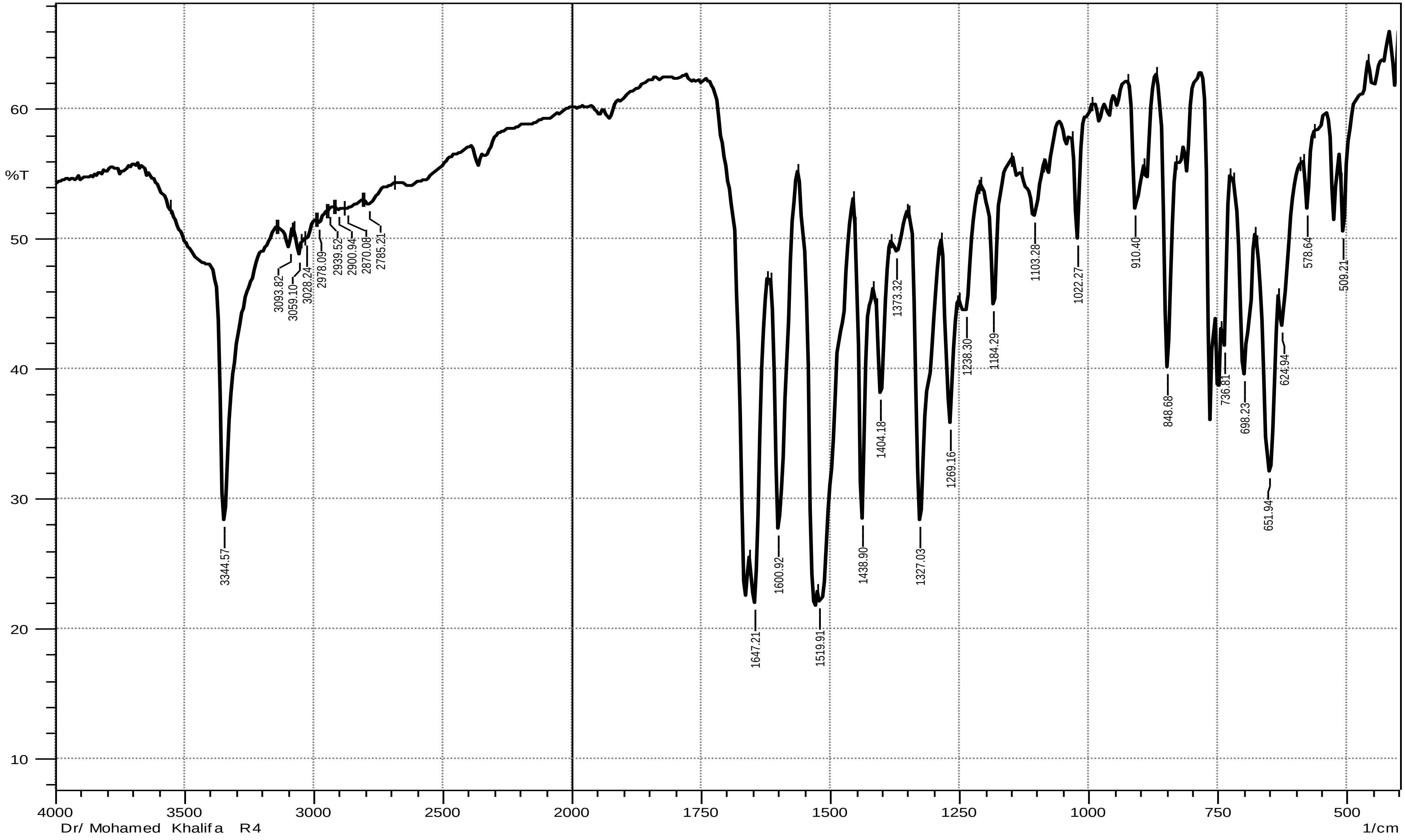

Dr/ Mohamed Khalifa 7d

جامعة القاهرة
كلية الصيدلة
MAU
Microanalytical Unit-FOPCU
وحدة التحاليل الدقيقة
معمل الأشعة تحت الحمراء
IR Lab

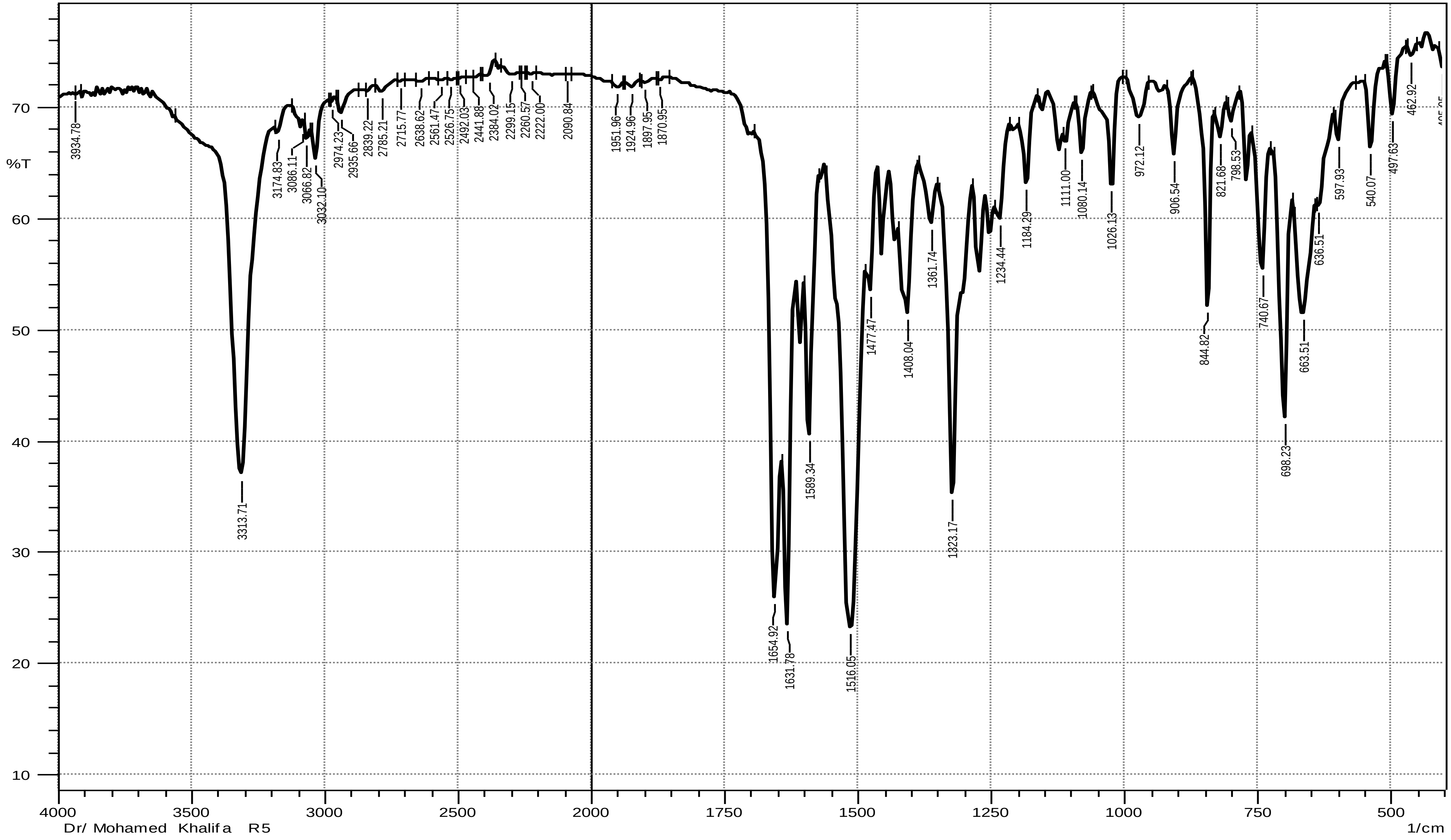

Dr/ Mohamed Khalifa 6

جامعة القاهرة
كلية الصيدلة

MAU
Microanalytical Unit-FOPCU
وحدة التحاليل الدقيقة
معمل الأشعة تحت الحمراء

IR Lab

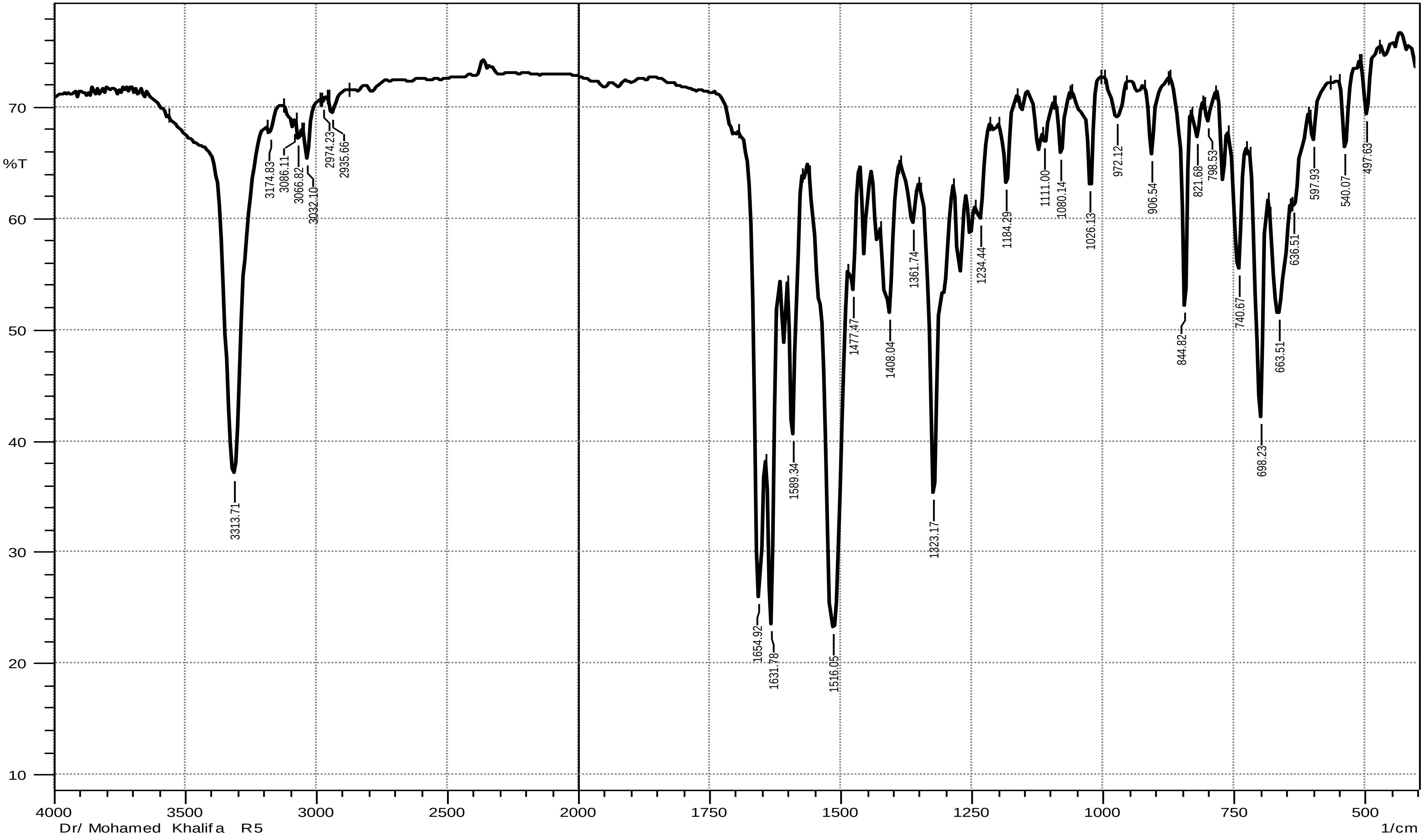

Dr/ Mohamed Khalifa 6

جامعة القاهرة

كلية الصيدلة

MAU

Microanalytical Unit-FOPCU

وحدة التحاليل الدقيقة

معمل الأشعة تحت الحمراء

IR Lab

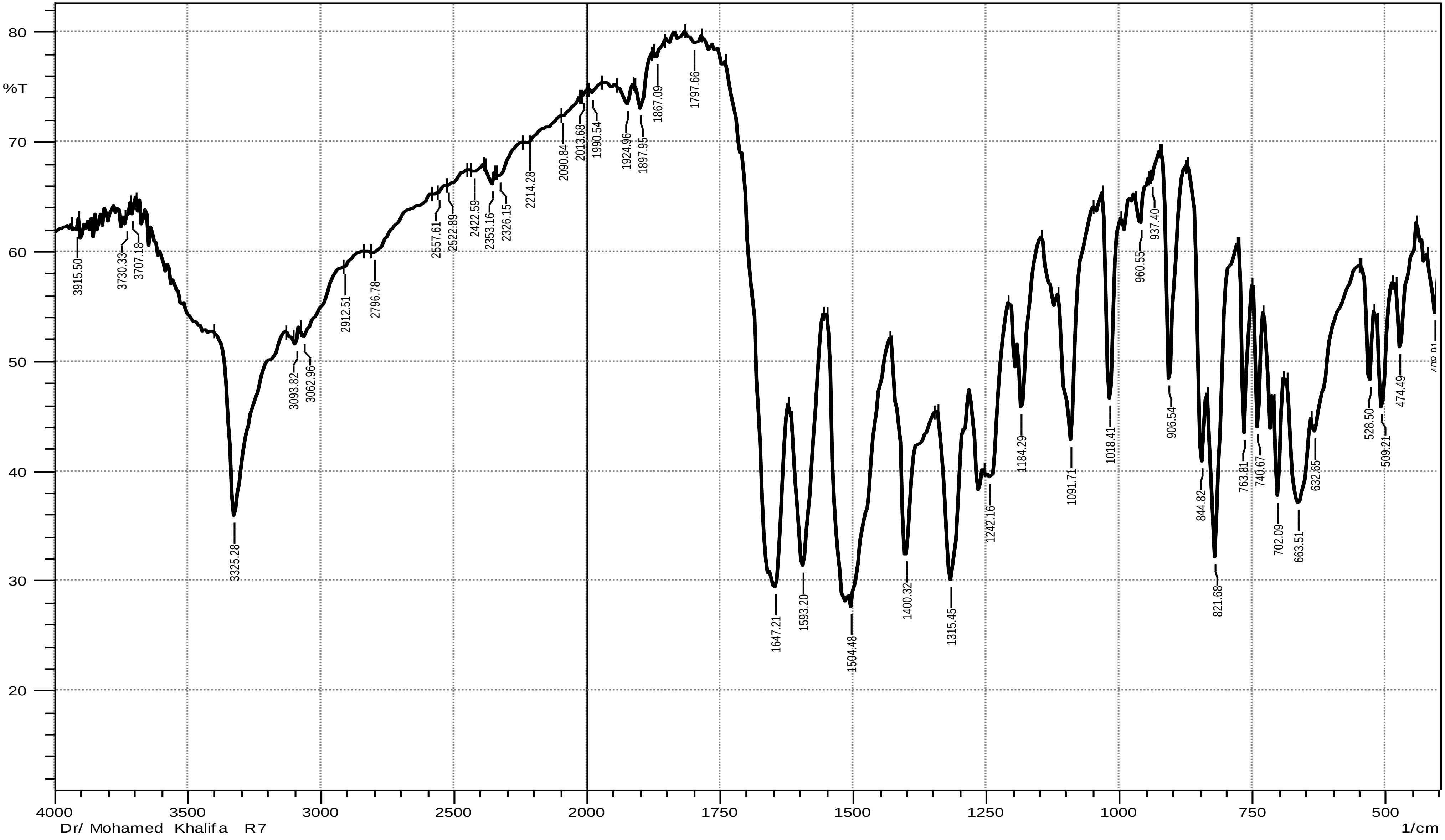

Dr/ Mohamed Khalifa 7e

جامعة القاهرة
كلية الصيدلة

Microanalytical Unit-FOPCU
وحدة التحاليل الدقيقة
معمل الأشعة تحت الحمراء

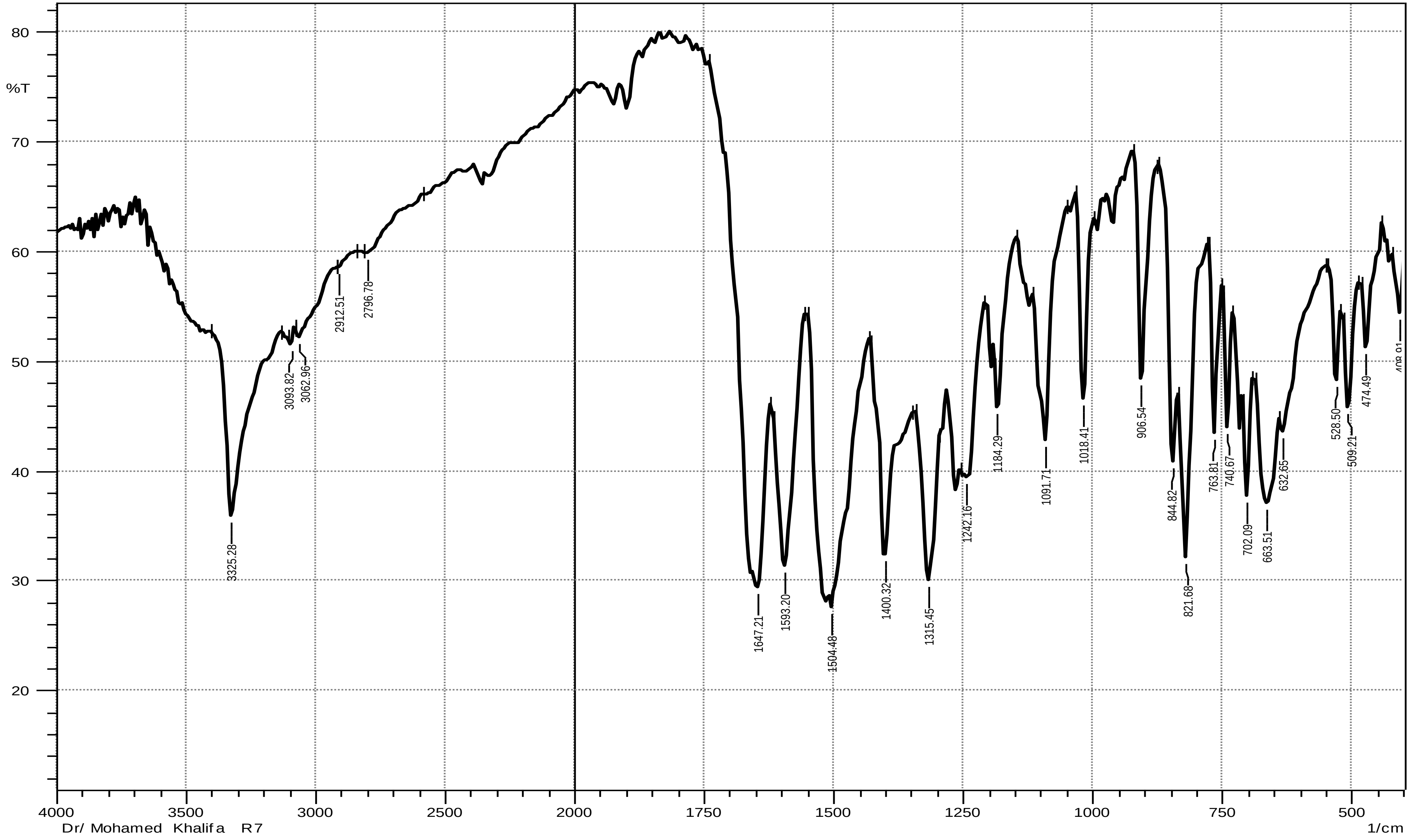

Dr/ Mohamed Khalifa 7e

جامعة القاهرة
كلية الصيدلة
MAU
Microanalytical Unit-FOPCU
وحدة التحاليل الدقيقة
معمل الأشعة تحت الحمراء
IR Lab

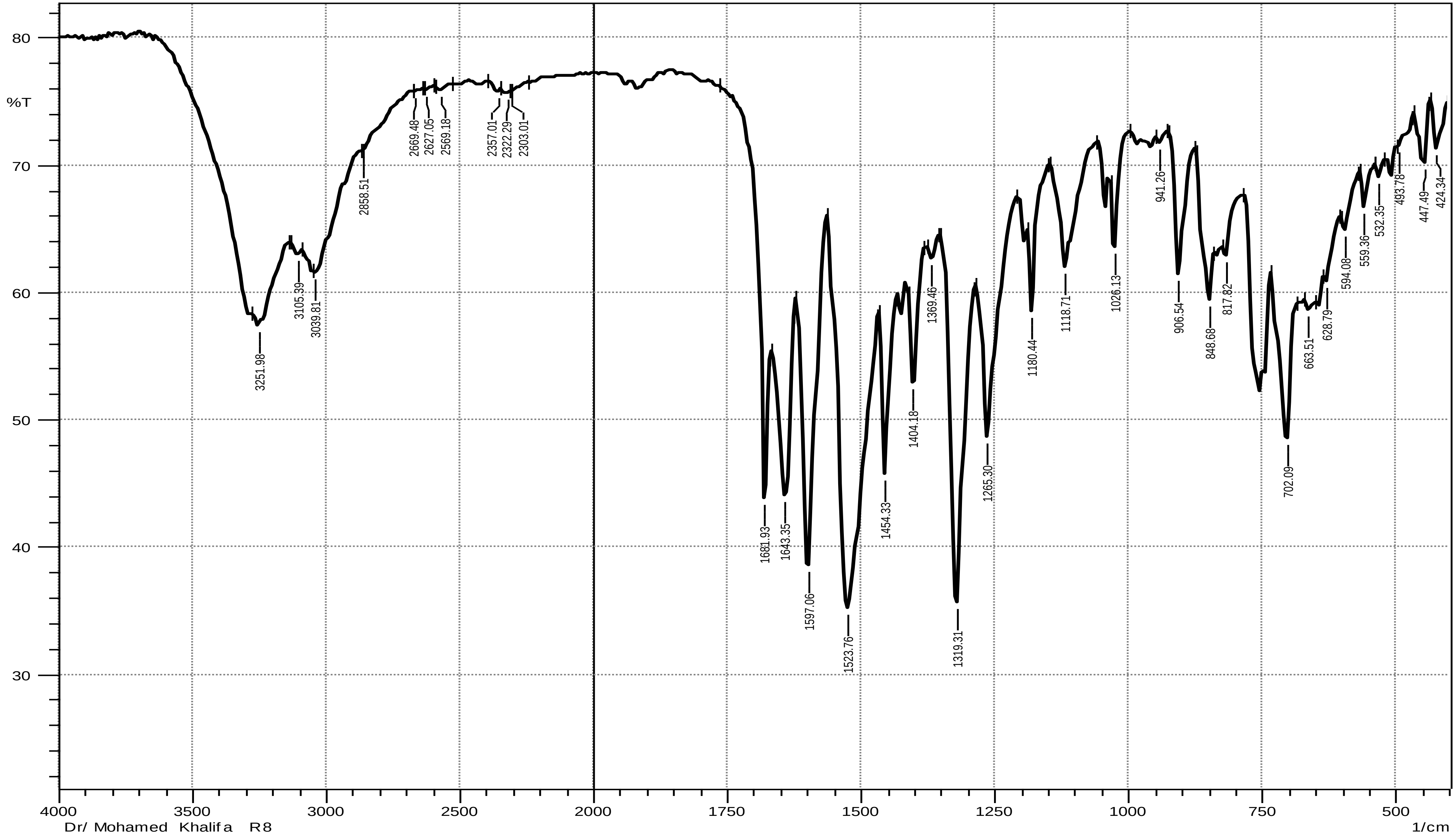

Dr/ Mohamed Khalifa 7f

جامعة القاهرة

كلية الصيدلة

MAU

Microanalytical Unit-FOPCU

وحدة التحاليل الدقيقة

معمل الأشعة تحت الحمراء

IR Lab

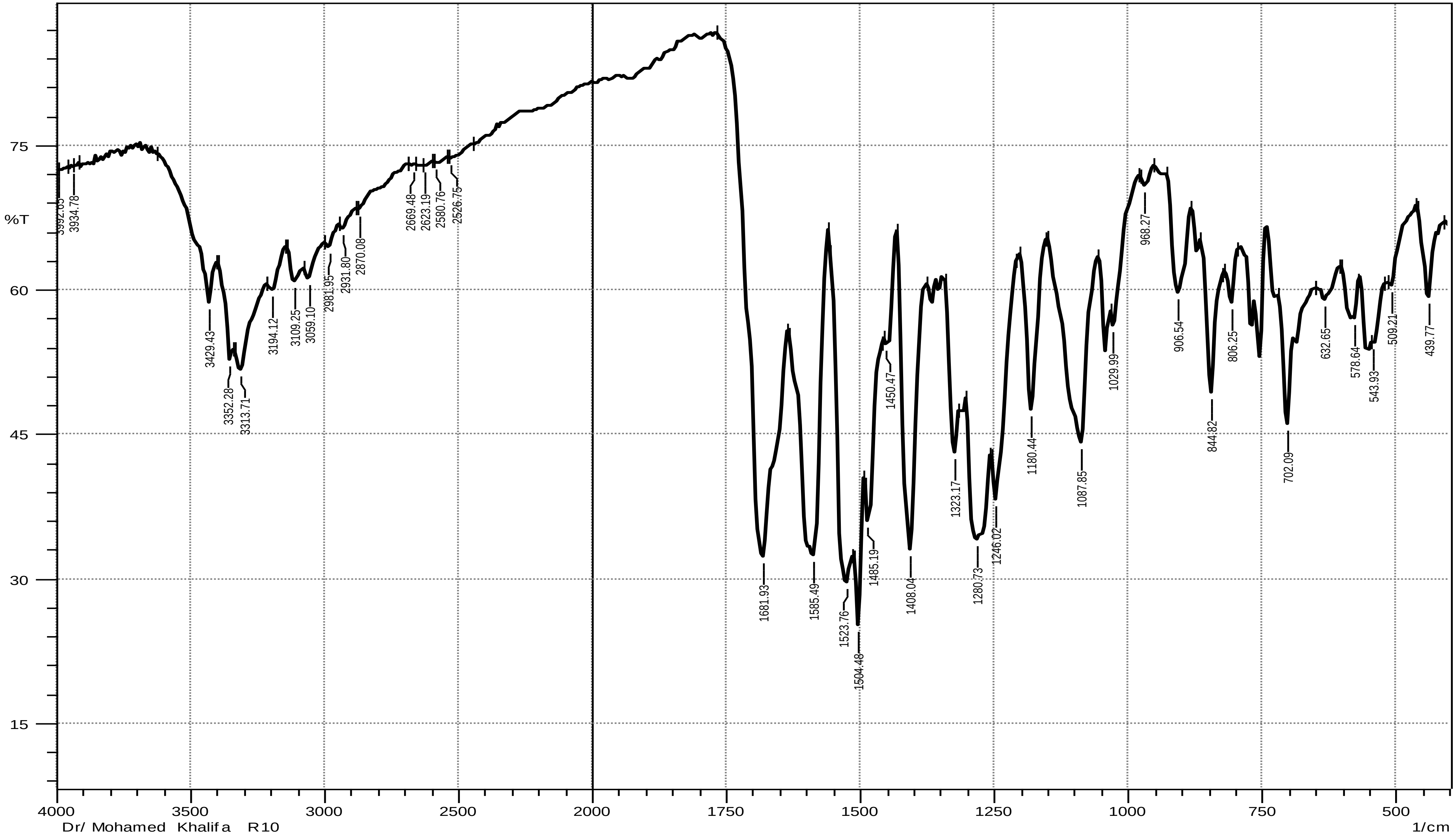

Dr/ Mohamed Khalifa 7g

جامعة القاهرة
كلية الصيدلة

Microanalytical Unit-FOPCU
وحدة التحاليل الدقيقة
معمل الأشعة تحت الحمراء

IR Lab

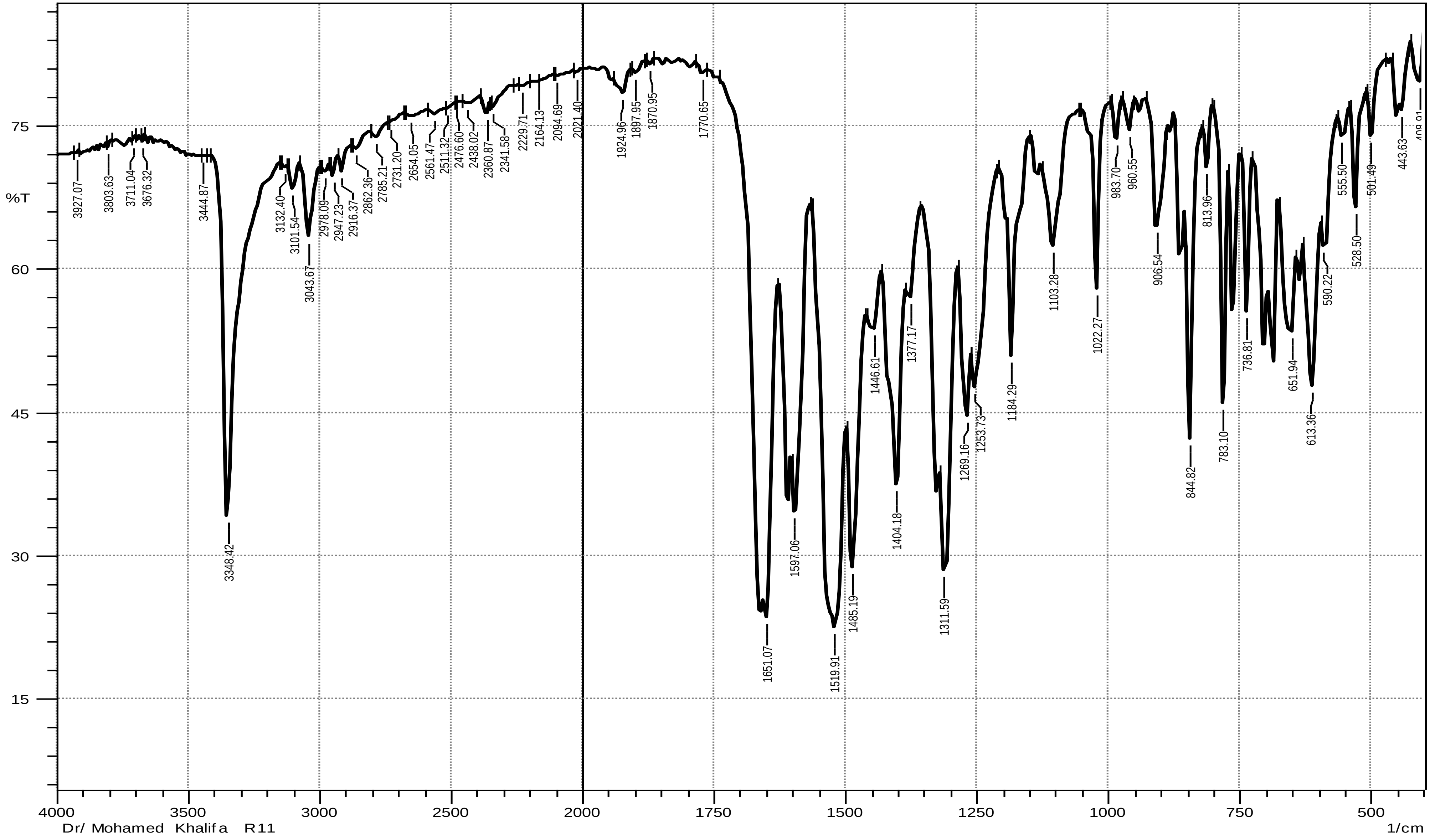

Dr/ Mohamed Khalifa 7h

جامعة القاهرة
كلية الصيدلة

MAU
Microanalytical Unit-FOPCU
وحدة التحاليل الدقيقة
معمل الأشعة تحت الحمراء

IR Lab

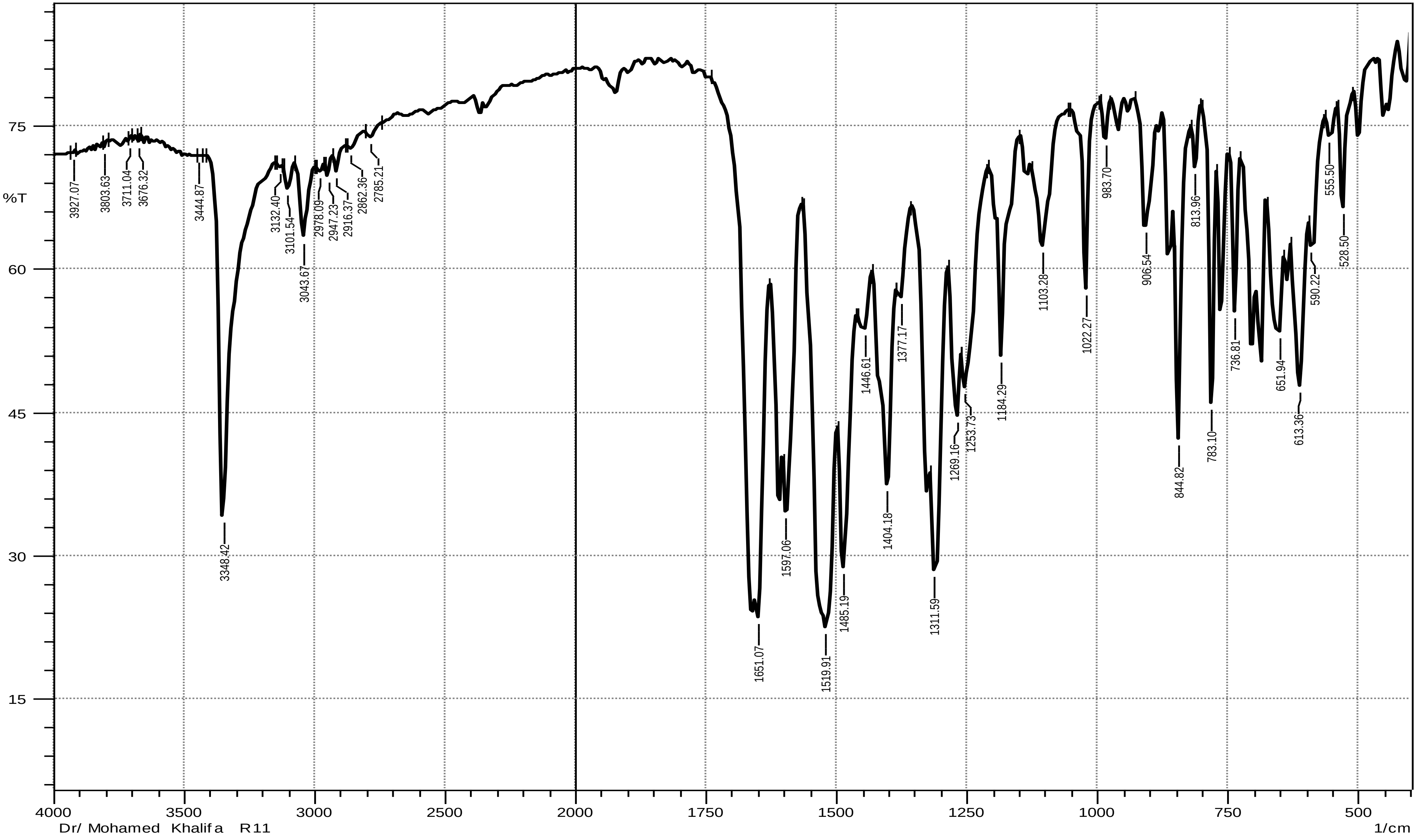

Dr/ Mohamed Khalifa 7h

جامعة القاهرة
كلية الصيدلة

MAU
Microanalytical Unit-FOPCU
وحدة التحاليل الدقيقة
معمل الأشعة تحت الحمراء

IR Lab

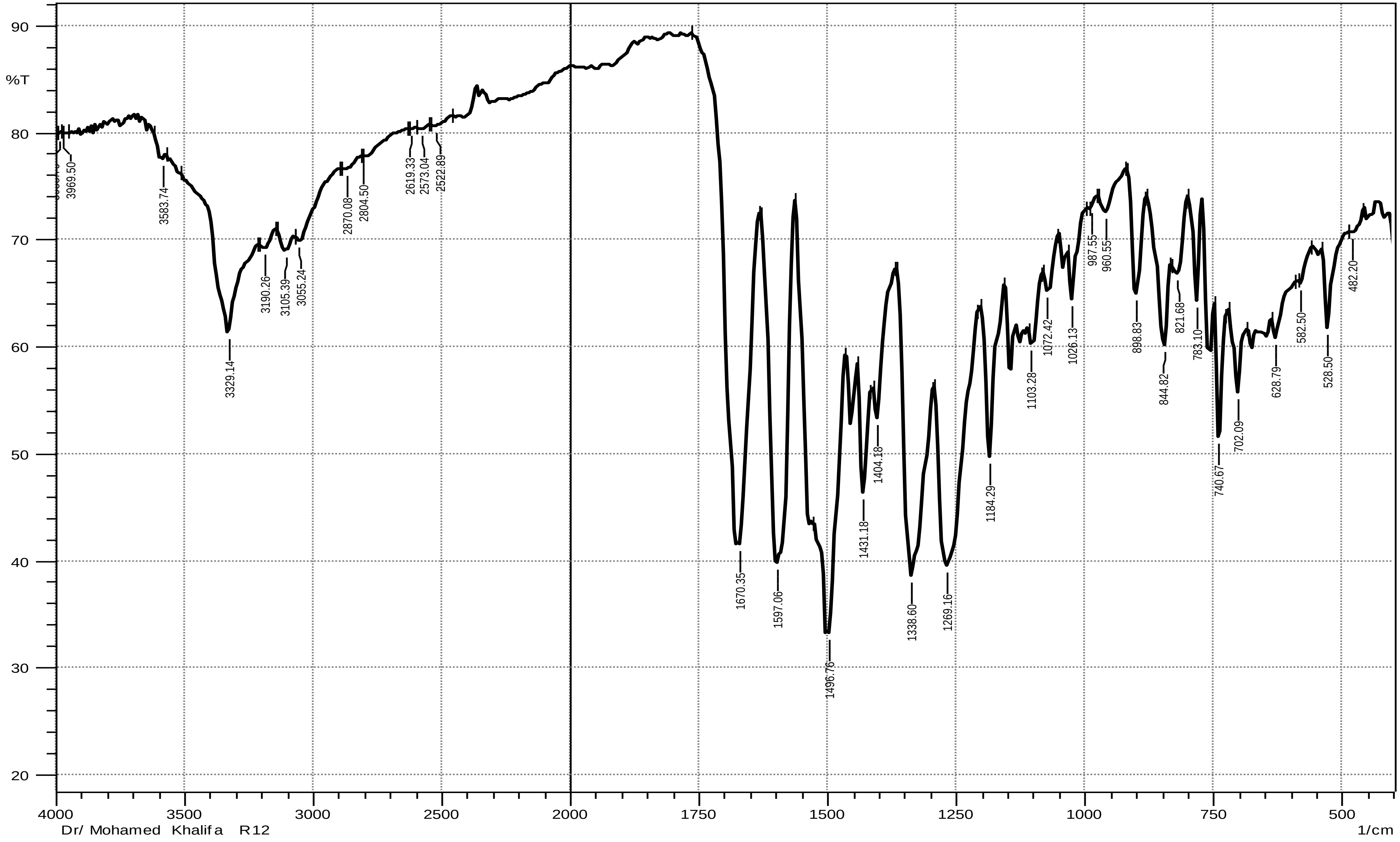

Dr/ Mohamed Khalifa 7i

Microanalytical Unit-FOPCU
وحدة التحاليل الدقيقة
معمل الأشعة تحت الحمراء

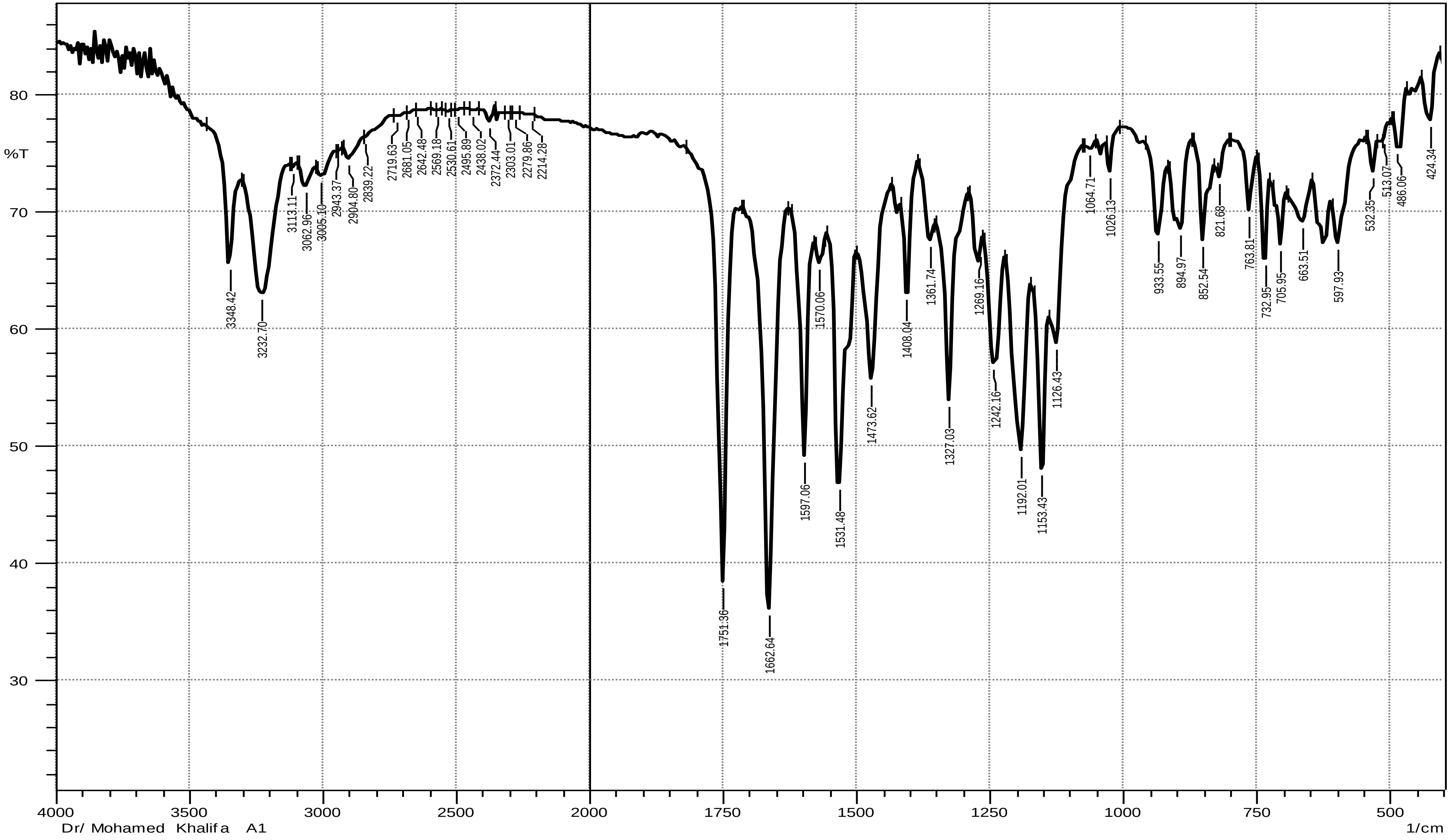

Dr/ Mohamed Khalifa 10

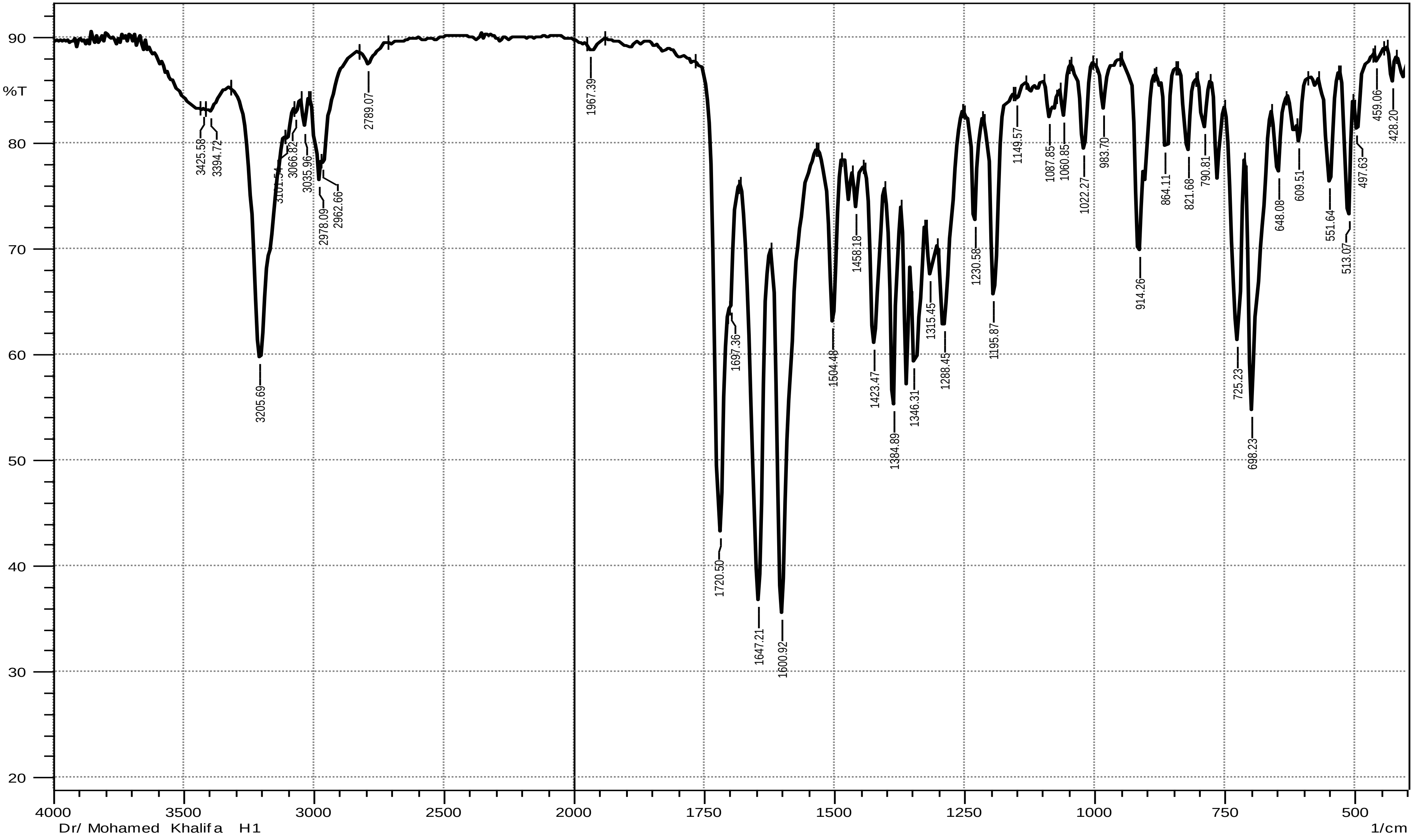

Dr/ Mohamed Khalifa 18c

جامعة القاهرة

كلية الصيدلة

MAU

Microanalytical Unit-FOPCU

وحدة التحاليل الدقيقة

معمل الأشعة تحت الحمراء

IR Lab

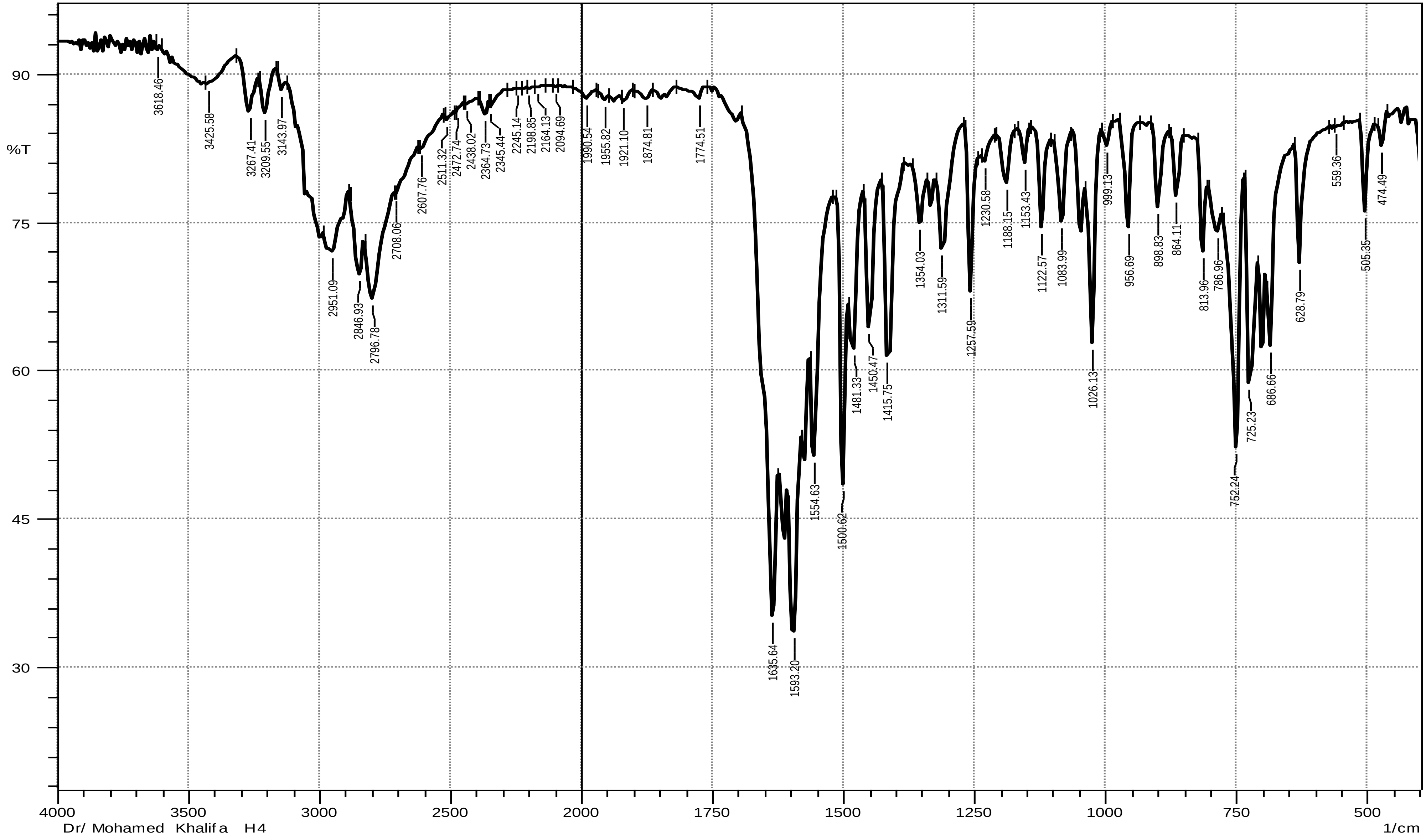

Dr/ Mohamed Khalifa 18b

جامعة القاهرة
كلية الصيدلة

MAU
Microanalytical Unit-FOPCU
وحدة التحاليل الدقيقة
معمل الأشعة تحت الحمراء

IR Lab

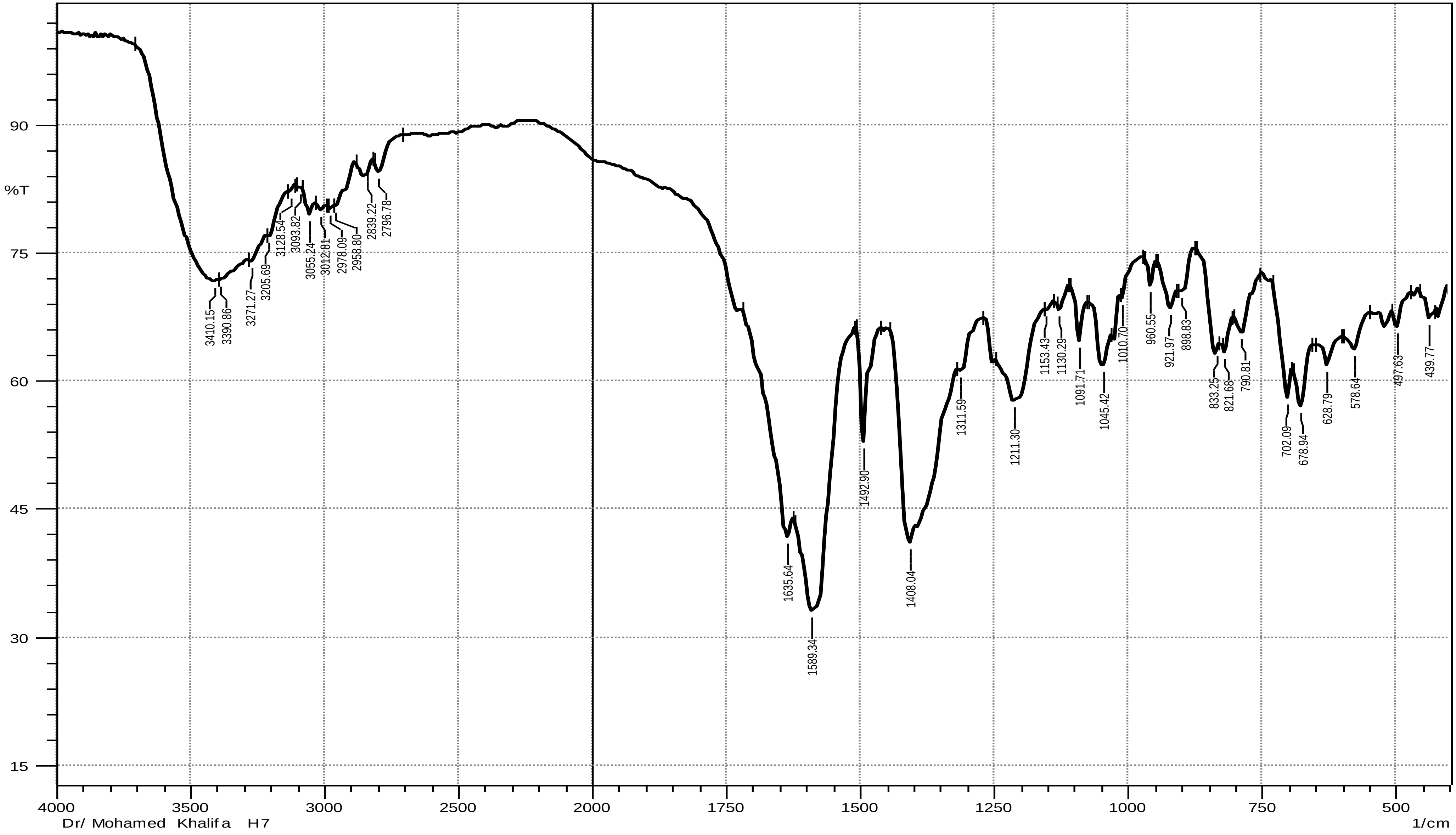

Dr/ Mohamed Khalifa 18a

جامعة القاهرة
كلية الصيدلة

MAU
Microanalytical Unit-FOPCU
وحدة التحاليل الدقيقة
معمل الأشعة تحت الحمراء

IR Lab

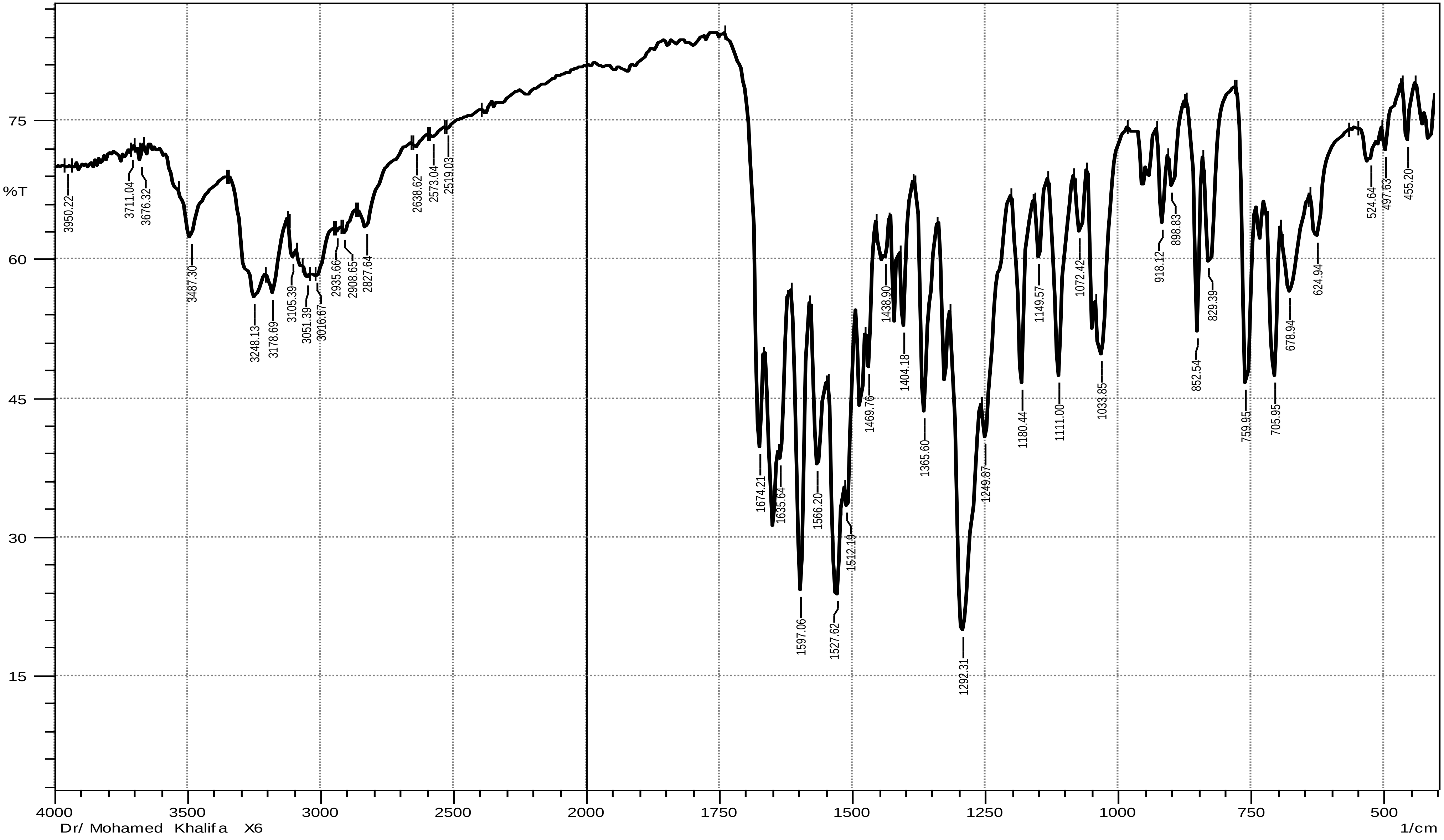

Dr/ Mohamed Khalifa 11f

جامعة القاهرة

كلية الصيدلة

MAU

Microanalytical Unit-FOPCU

وحدة التحاليل الدقيقة

معمل الأشعة تحت الحمراء

IR Lab

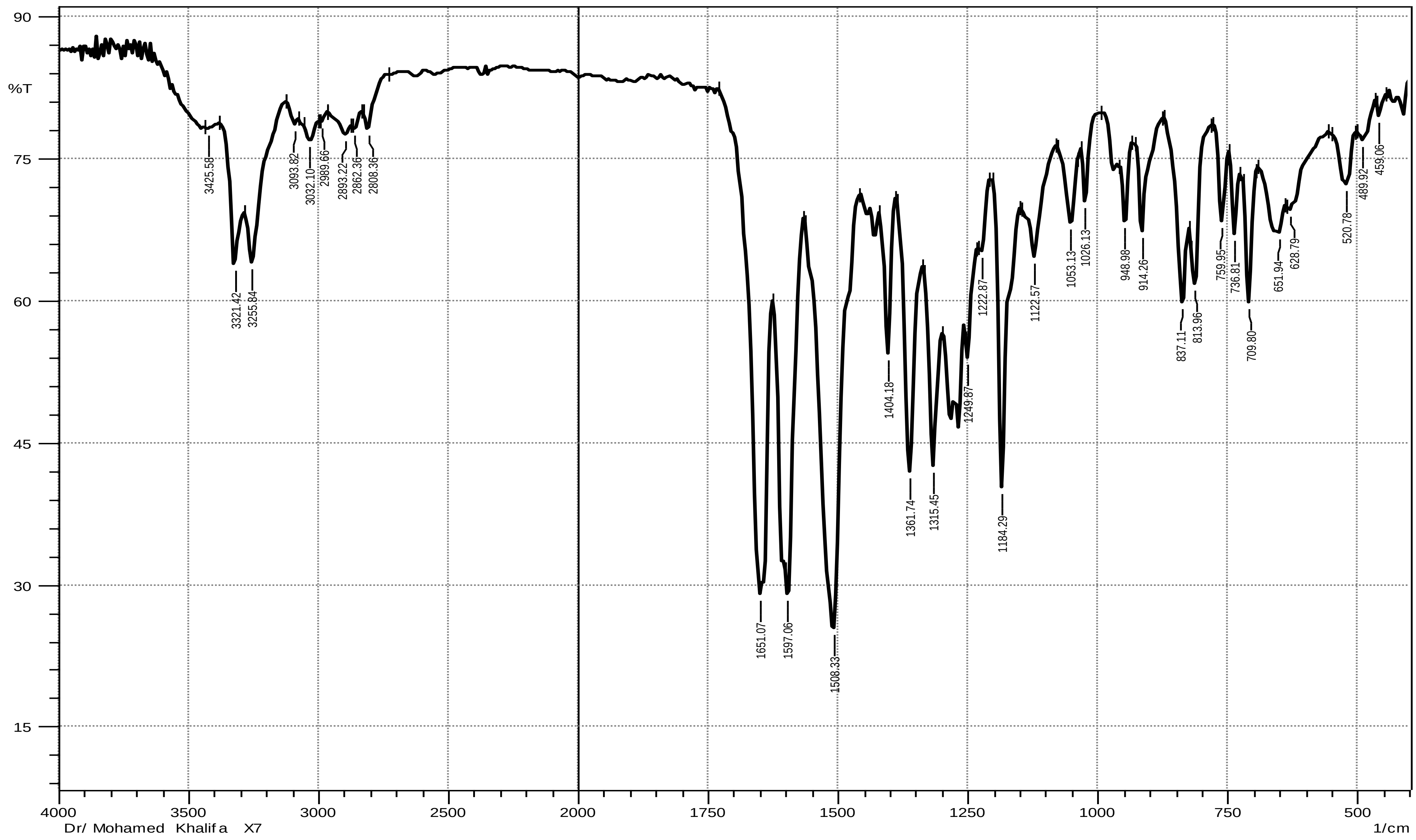

Dr/ Mohamed Khalifa 11g

جامعة القاهرة
كلية الصيدلة

MAU
Microanalytical Unit-FOPCU
وحدة التحاليل الدقيقة
معمل الأشعة تحت الحمراء

IR Lab

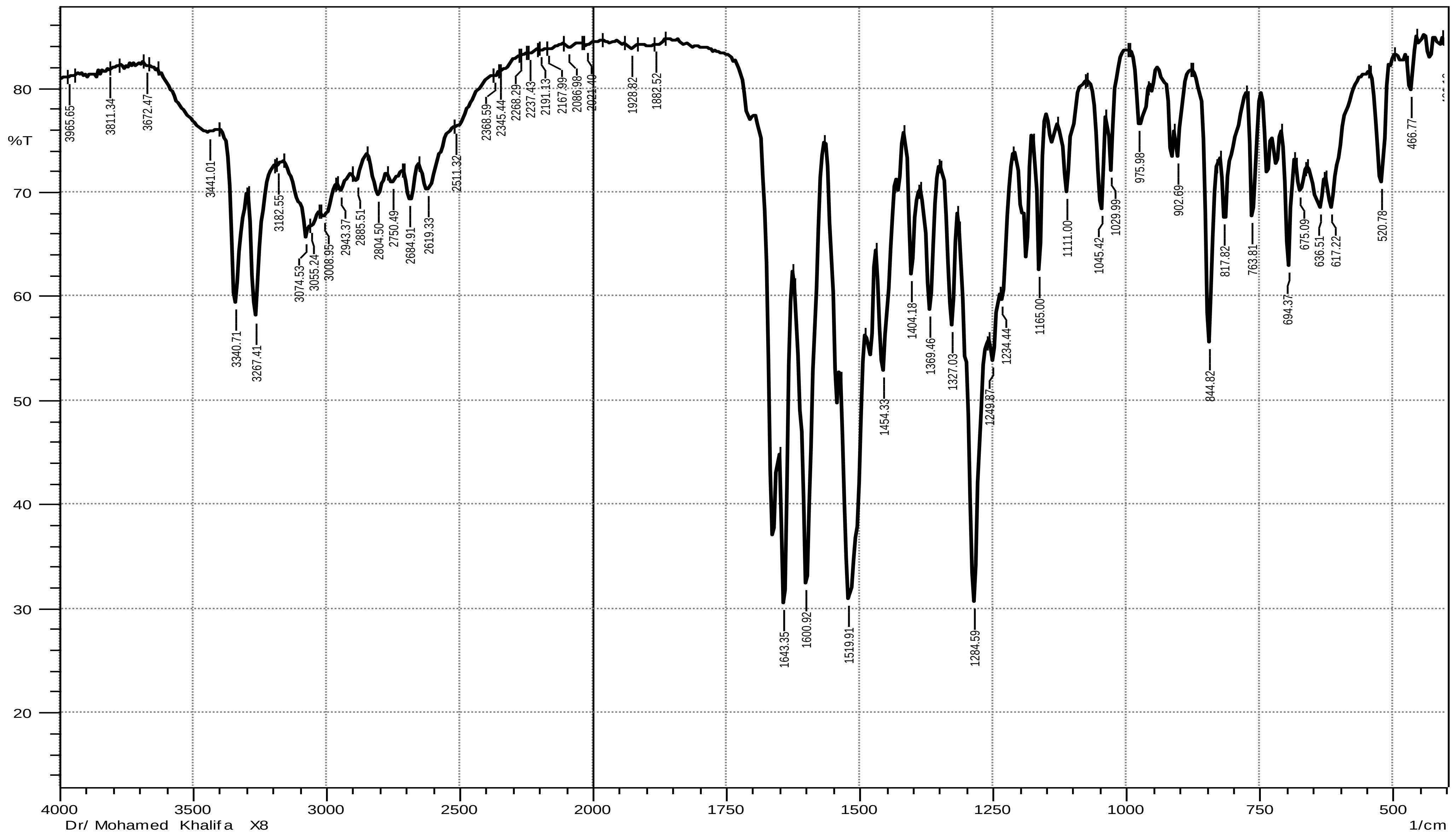

Dr/ Mohamed Khalifa 11h

mohamed khalifa- H1-Hnmr-ow

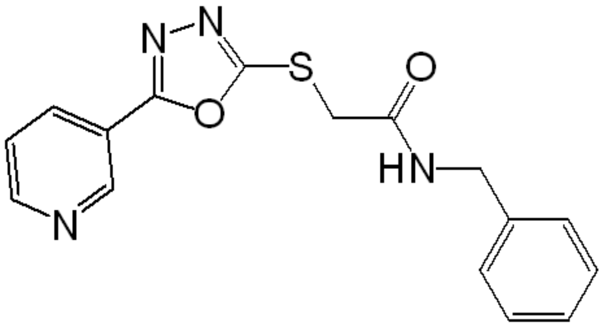

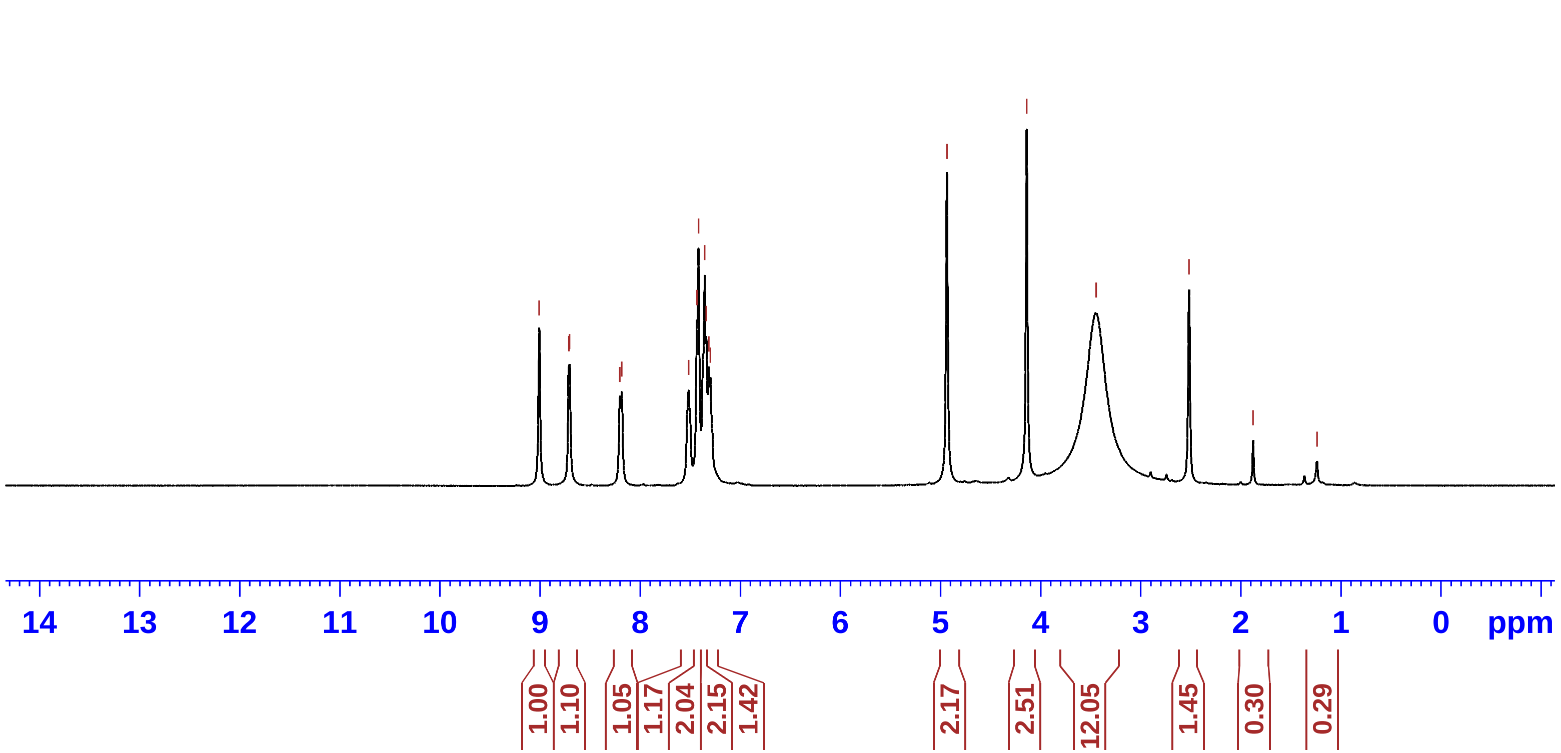


```
Current Data Parameters
NAME      mohamed khalifa- H1-Hnmr-ow
EXPNO                    10
PROCNO                    1

F2 - Acquisition Parameters
Date_              20201116
Time                  12.16 h
INSTRUM               spect
PROBHD   Z108618_0945 (
PULPROG                zg30
TD                    65536
SOLVENT                DMSO
NS                       16
DS                        2
SWH                8012.820 Hz
FIDRES             0.244532 Hz
AQ                4.0894465 sec
RG                     99.3
DW                   62.400 usec
DE                     6.50 usec
TE                    294.7 K
D1               1.00000000 sec
TD0                       1
SFO1            400.2024712 MHz
NUC1                     1H
P1                    13.50 usec
PLW1            13.00000000 W

F2 - Processing parameters
SI                    65536
SF              400.2000000 MHz
WDW                      EM
SSB      0
LB                     0.30 Hz
GB       0
PC                     1.00
```

mohamed khalifa- H1-Hnmr-ow

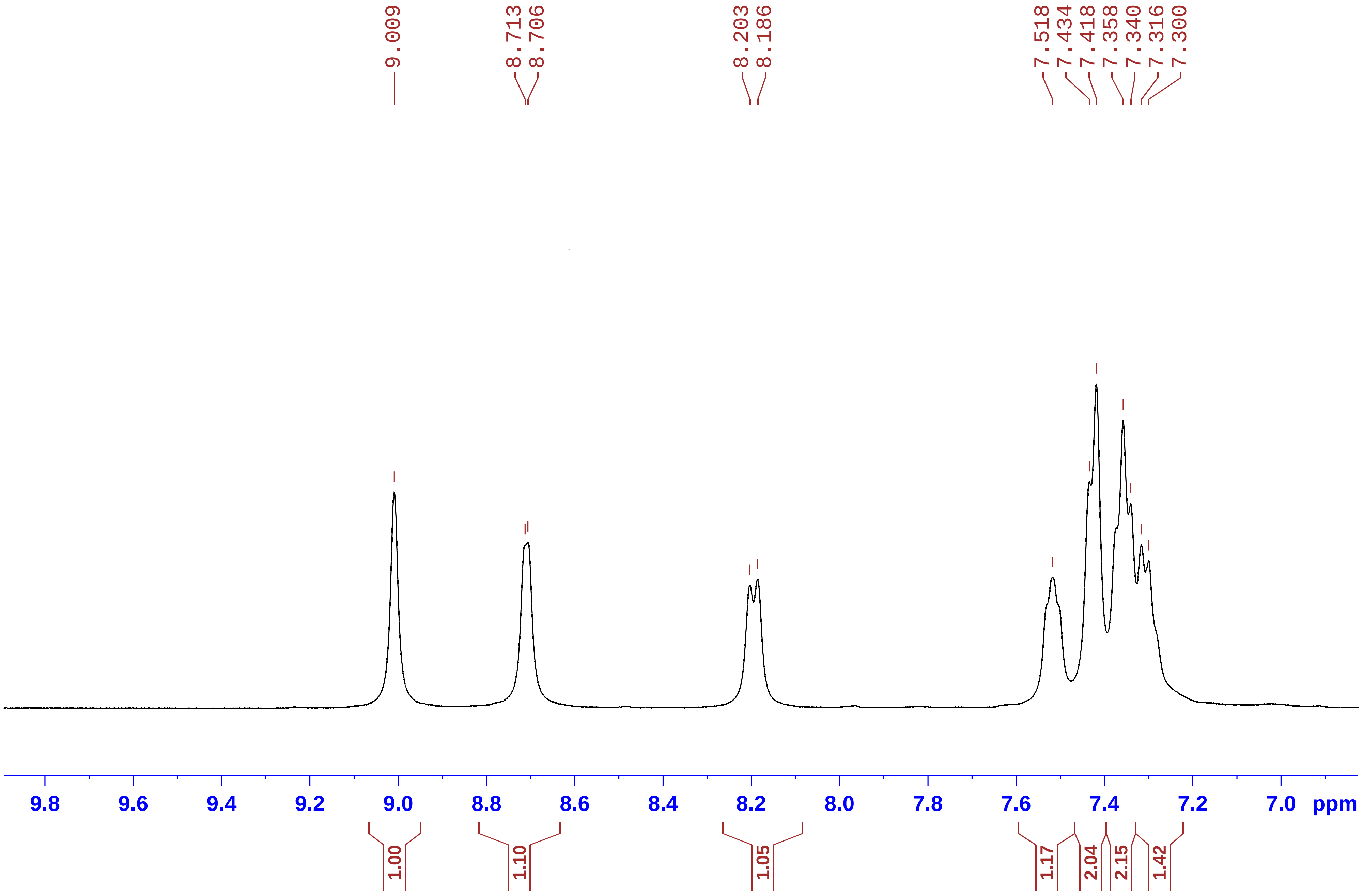

mohamed khalifa- H1-Hnmr-ow

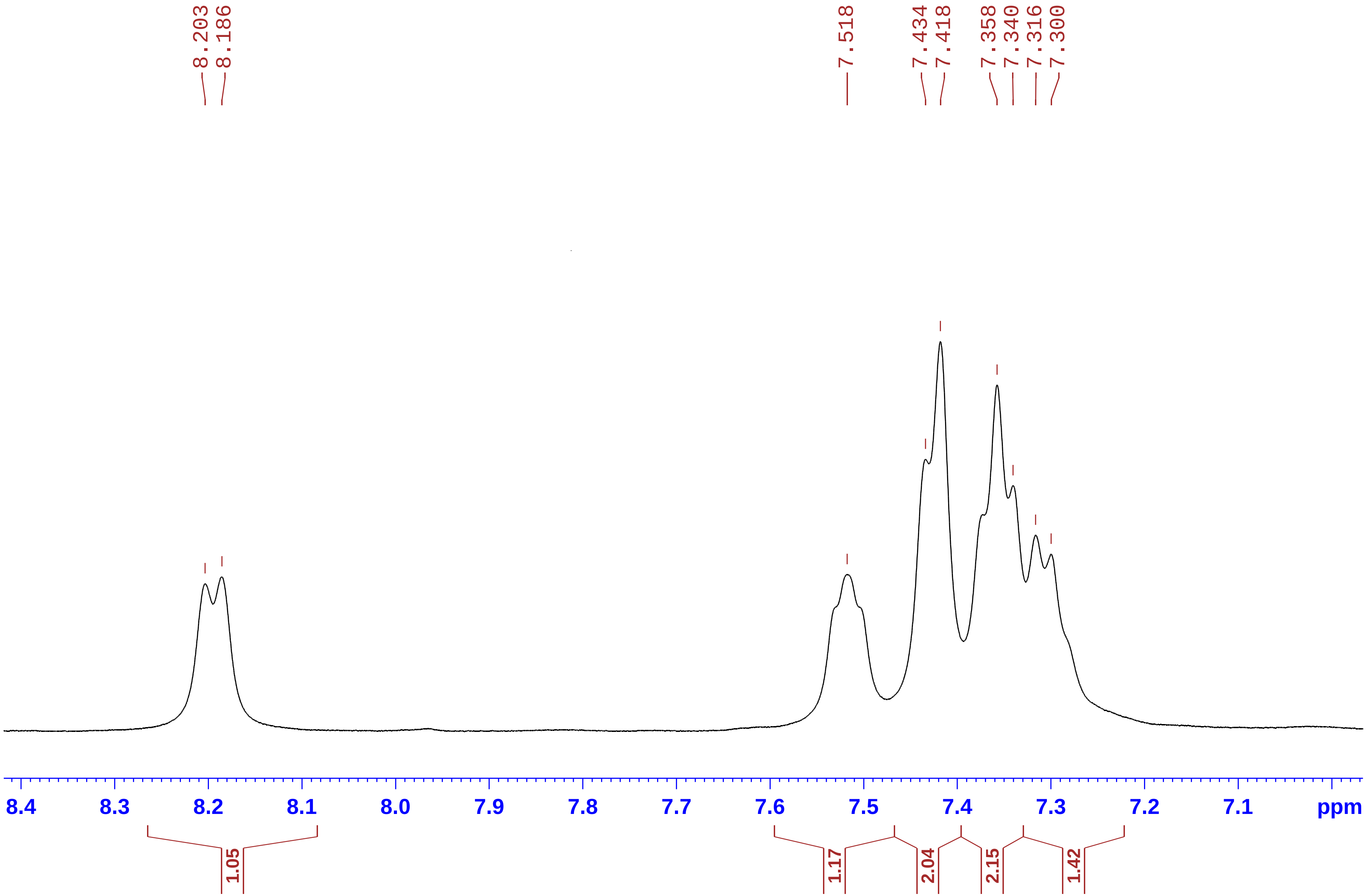

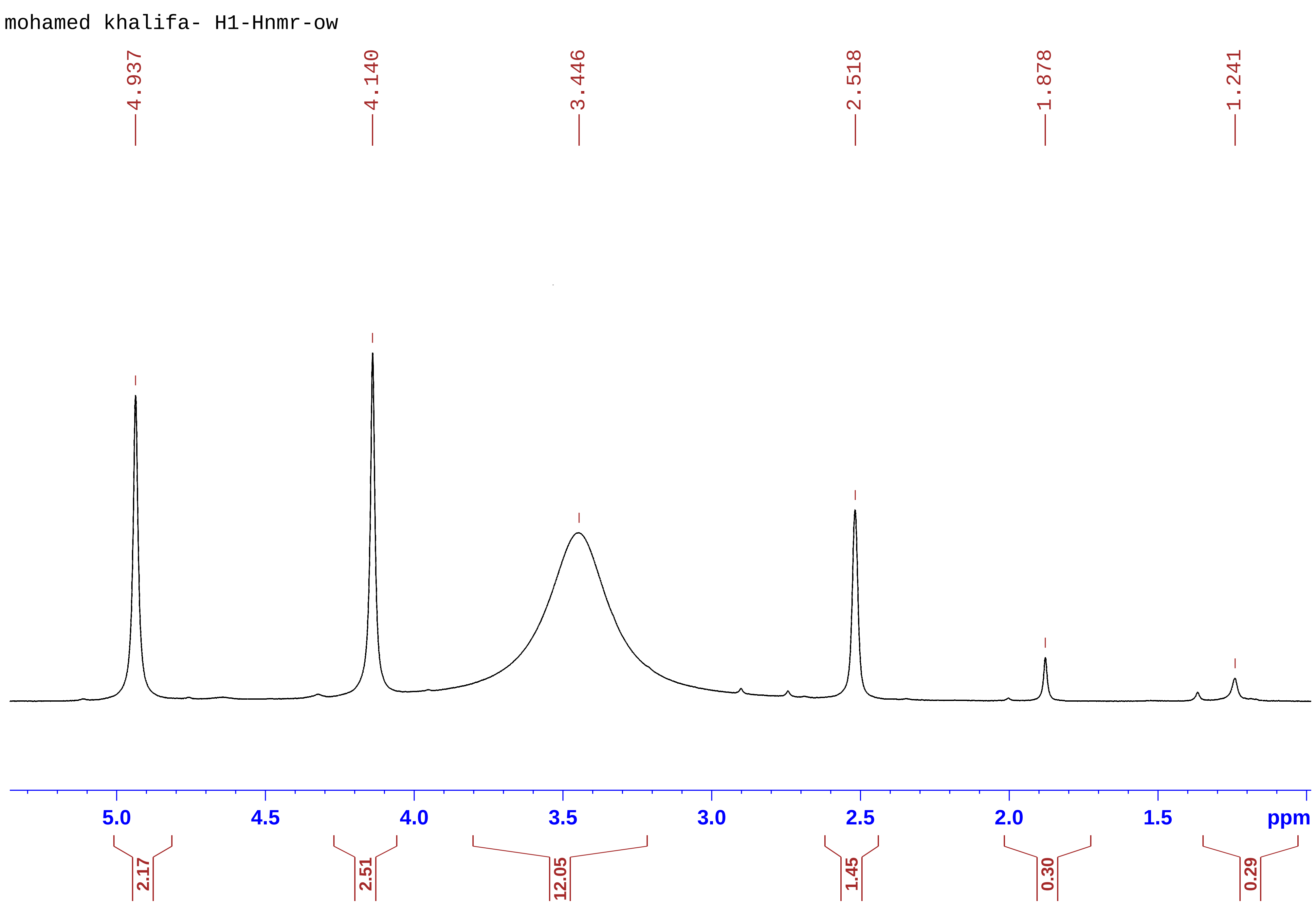
mohamed khalifa- H1-Hnmr-ow
4.937
4.140
3.446
2.518
1.878
1.241
5.0
4.5
4.0
3.5
3.0
2.5
2.0
1.5
ppm
2.17
2.51
12.05
1.45
0.30
0.29

mohamed khalifa- R16-Hnmr

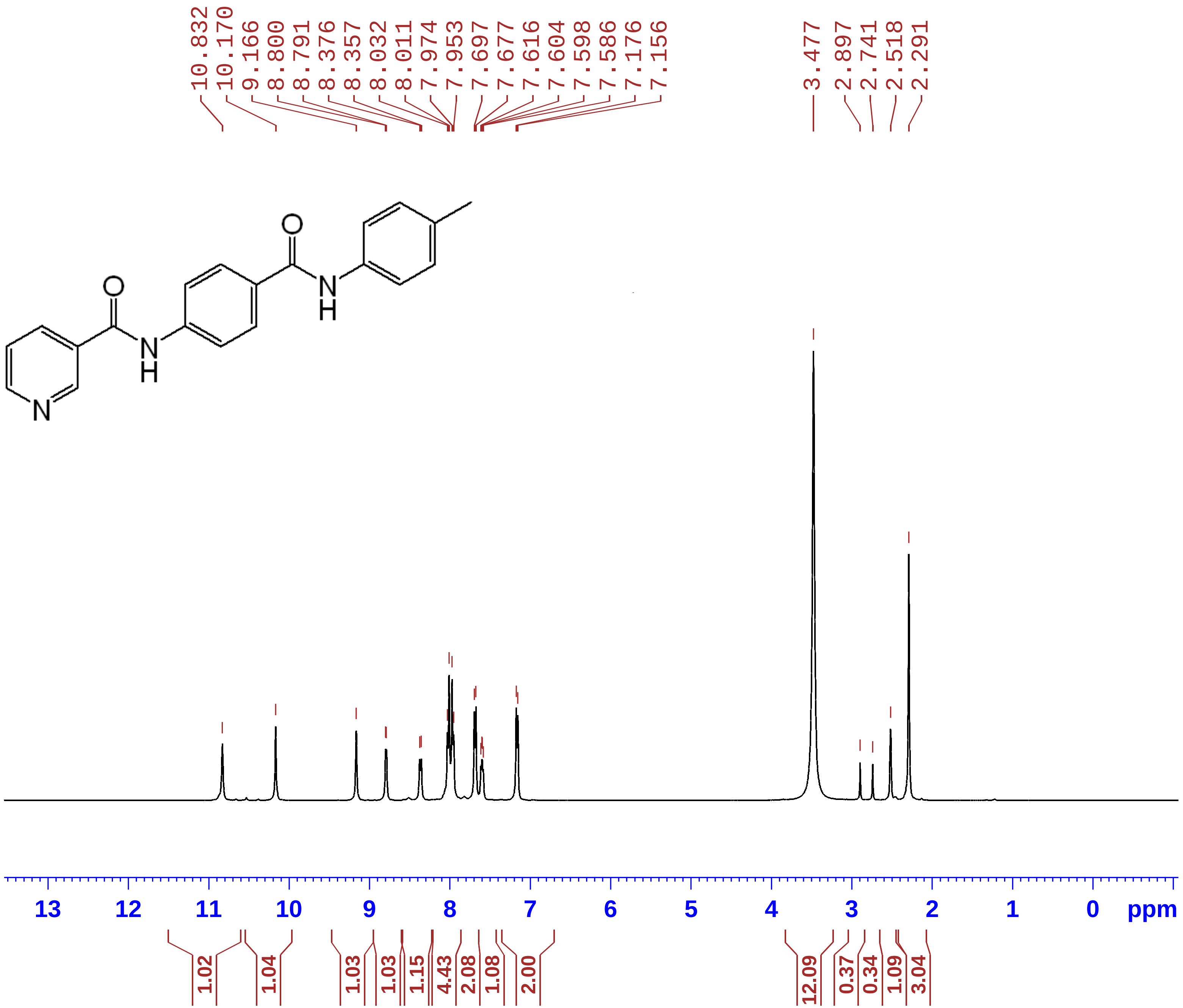

Faculty of Pharmacy

```
Current Data Parameters
NAME      mohamed khalifa- R16-Hnmr-ow
EXPNO                   10
PROCNO                   1

F2 - Acquisition Parameters
Date_              20201116
Time                  12.07 h
INSTRUM               spect
PROBHD    Z108618_0945 (
PULPROG                zg30
TD                    65536
SOLVENT                DMSO
NS                       16
DS                        2
SWH                8012.820 Hz
FIDRES             0.244532 Hz
AQ                4.0894465 sec
RG                    78.59
DW                   62.400 usec
DE                     6.50 usec
TE                    294.7 K
D1               1.00000000 sec
TD0                       1
SFO1            400.2024712 MHz
NUC1                     1H
P1                    13.50 usec
PLW1            13.00000000 W

F2 - Processing parameters
SI                    65536
SF              400.2000000 MHz
WDW                      EM
SSB       0
LB                     0.30 Hz
GB        0
PC                     1.00
```

mohamed khalifa- R16-Hnmr

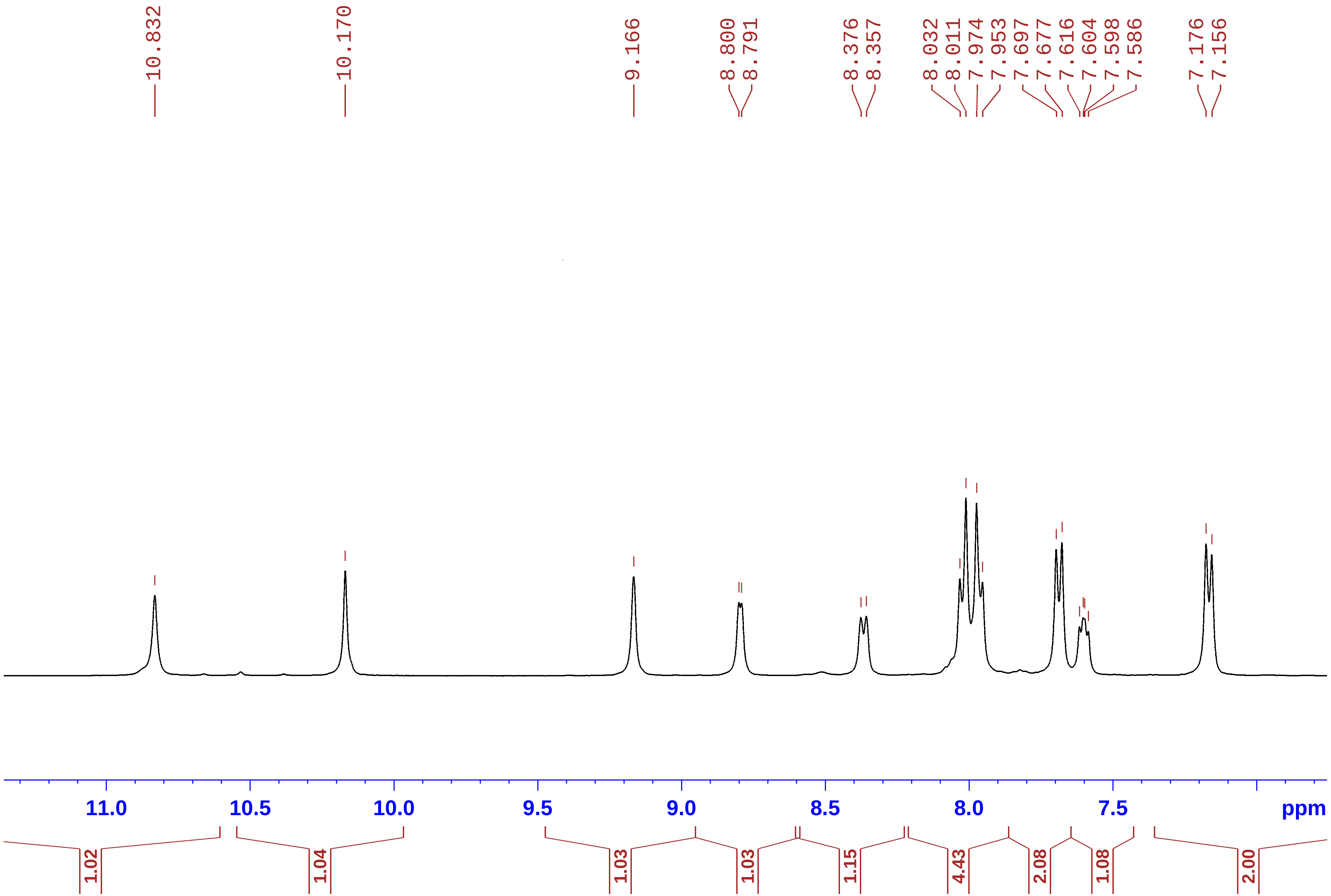

mohamed khalifa- R16-Hnmr

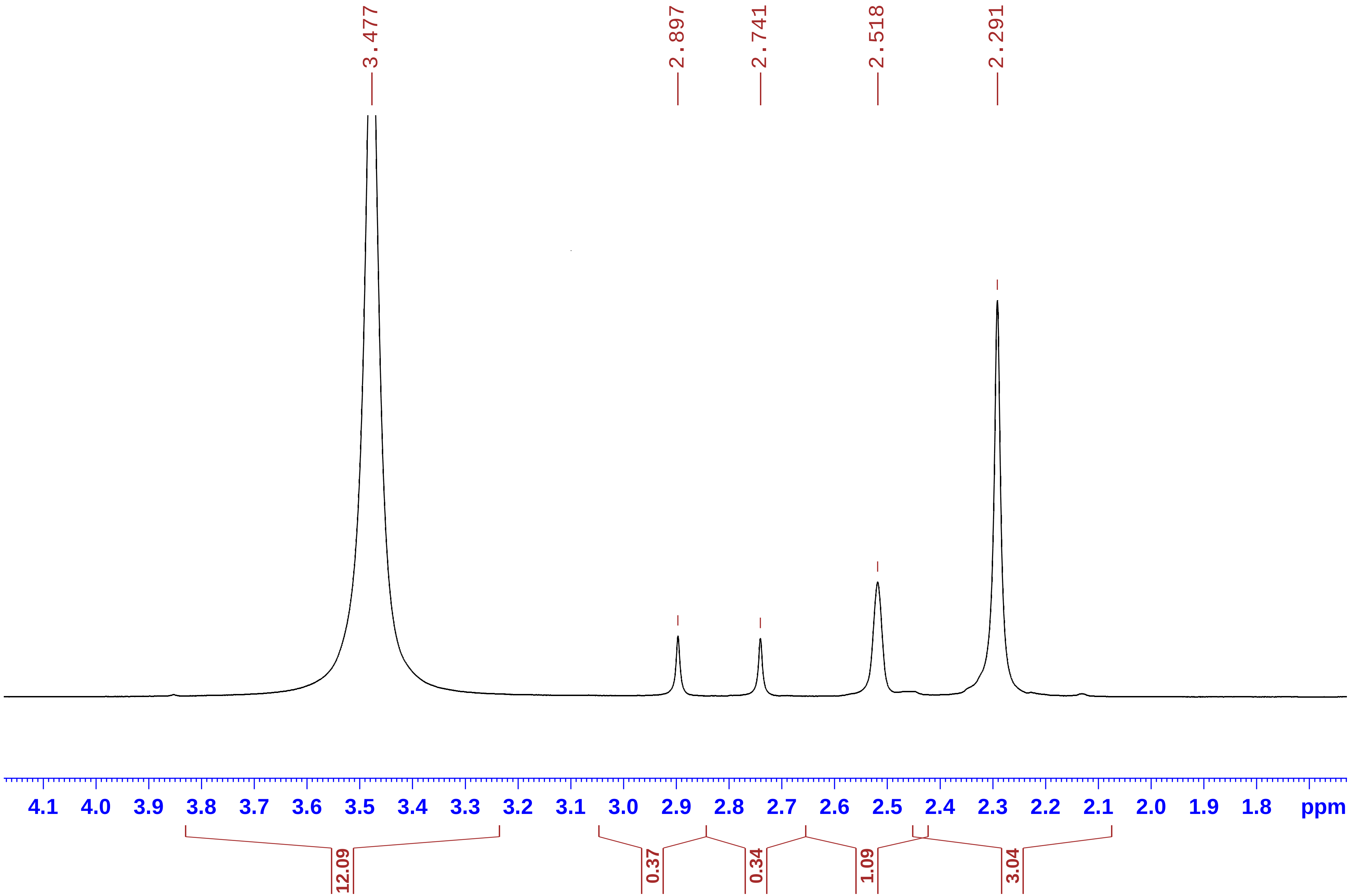

mohamed khalifa- R16-Hnmr

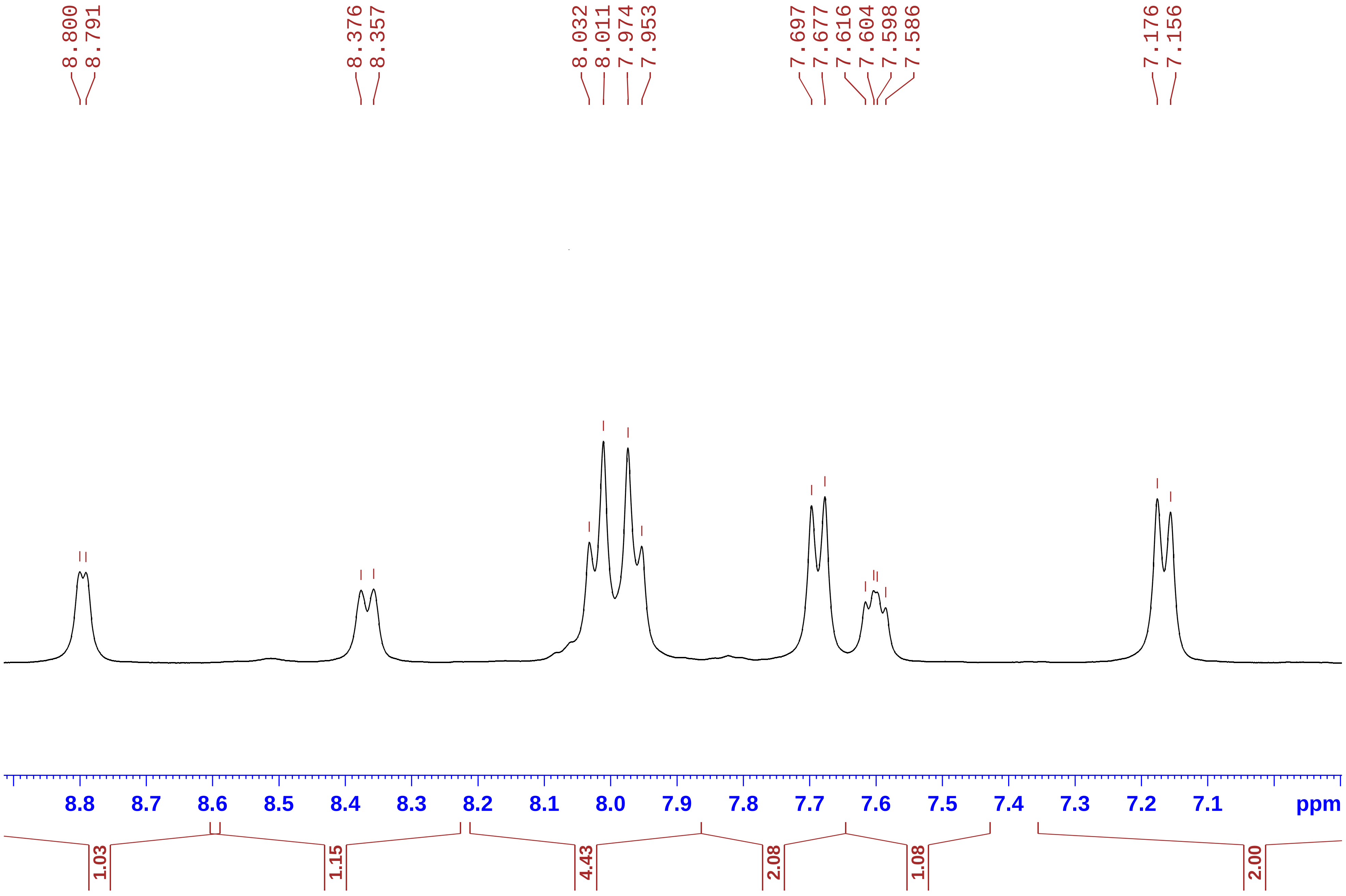

mohamed khalifa- X4-Hnmr-ow

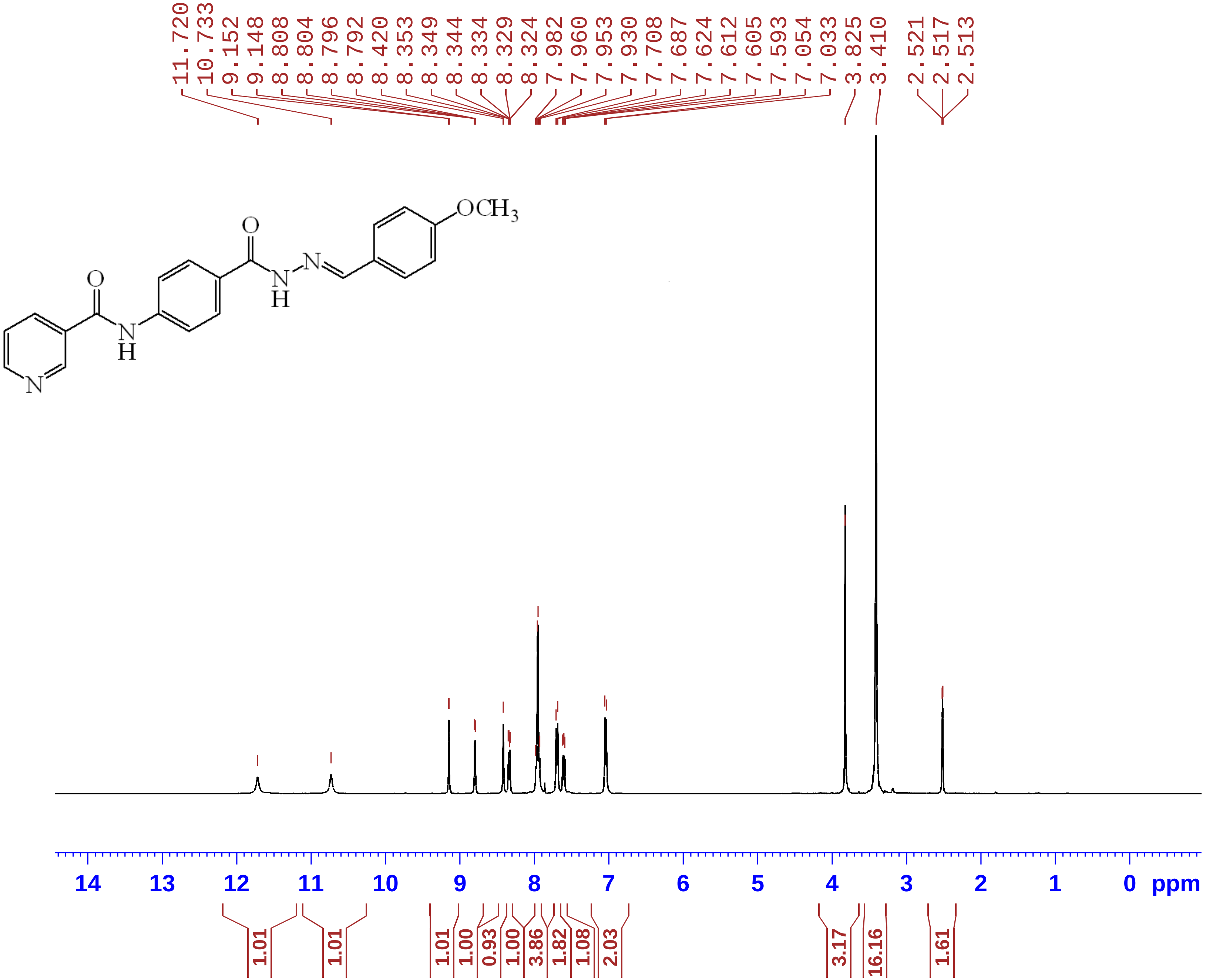

Faculty of Pharmacy

```
Current Data Parameters
NAME       mohamed khalifa- X4-Hnmr-ow
EXPNO                    10
PROCNO                    1

F2 - Acquisition Parameters
Date_              20201116
Time                  12.11 h
INSTRUM               spect
PROBHD        Z108618_0945 (
PULPROG                zg30
TD                    65536
SOLVENT                DMSO
NS                       16
DS                        2
SWH                8012.820 Hz
FIDRES             0.244532 Hz
AQ                4.0894465 sec
RG                     99.3
DW                   62.400 usec
DE                     6.50 usec
TE                    294.6 K
D1               1.00000000 sec
TD0                       1
SFO1            400.2024712 MHz
NUC1                     1H
P1                    13.50 usec
PLW1            13.00000000 W

F2 - Processing parameters
SI                    65536
SF              400.2000000 MHz
WDW                      EM
SSB      0
LB                     0.30 Hz
GB       0
PC                     1.00
```

mohamed khalifa- X4-Hnmr-ow

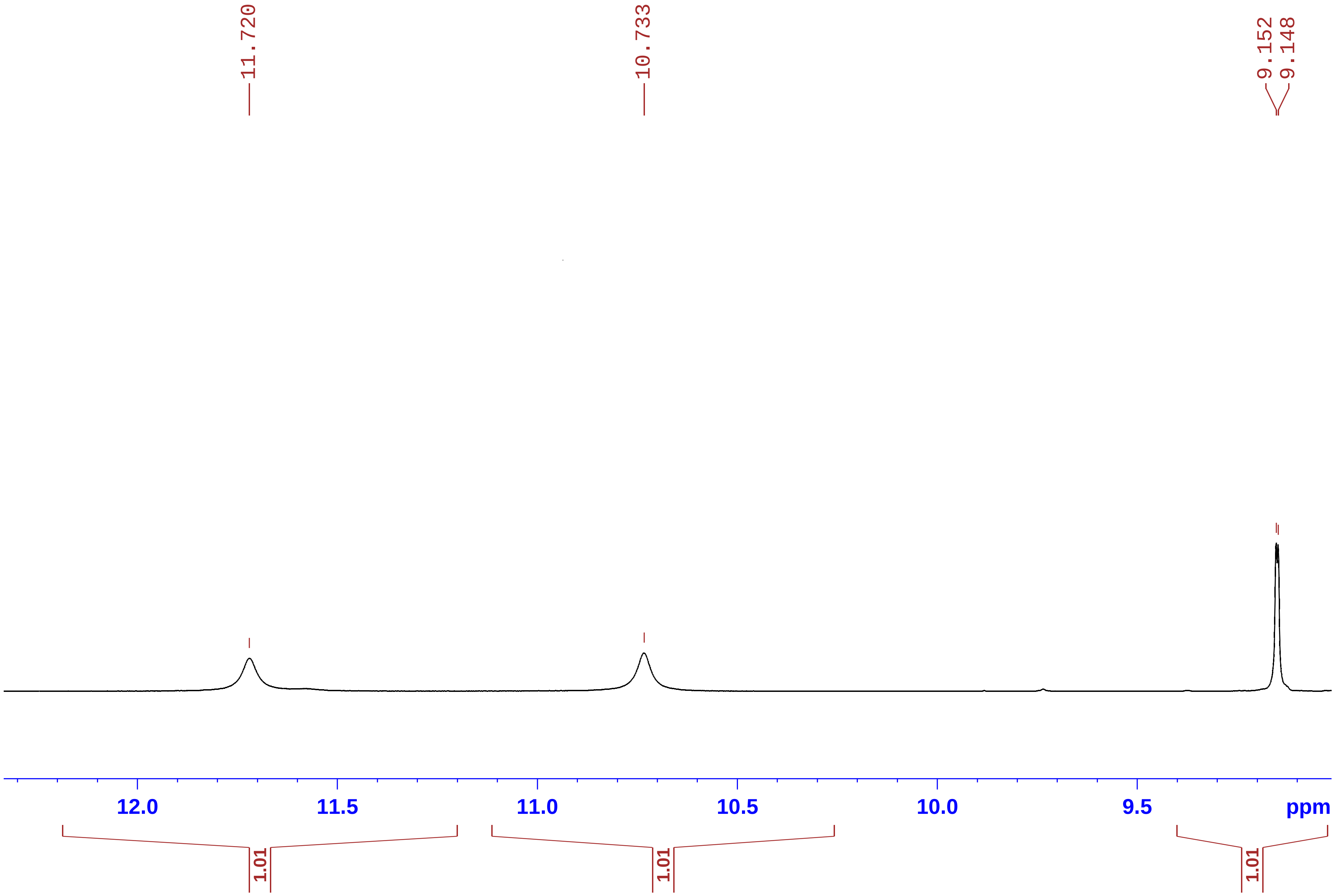

mohamed khalifa- X4-Hnmr-ow

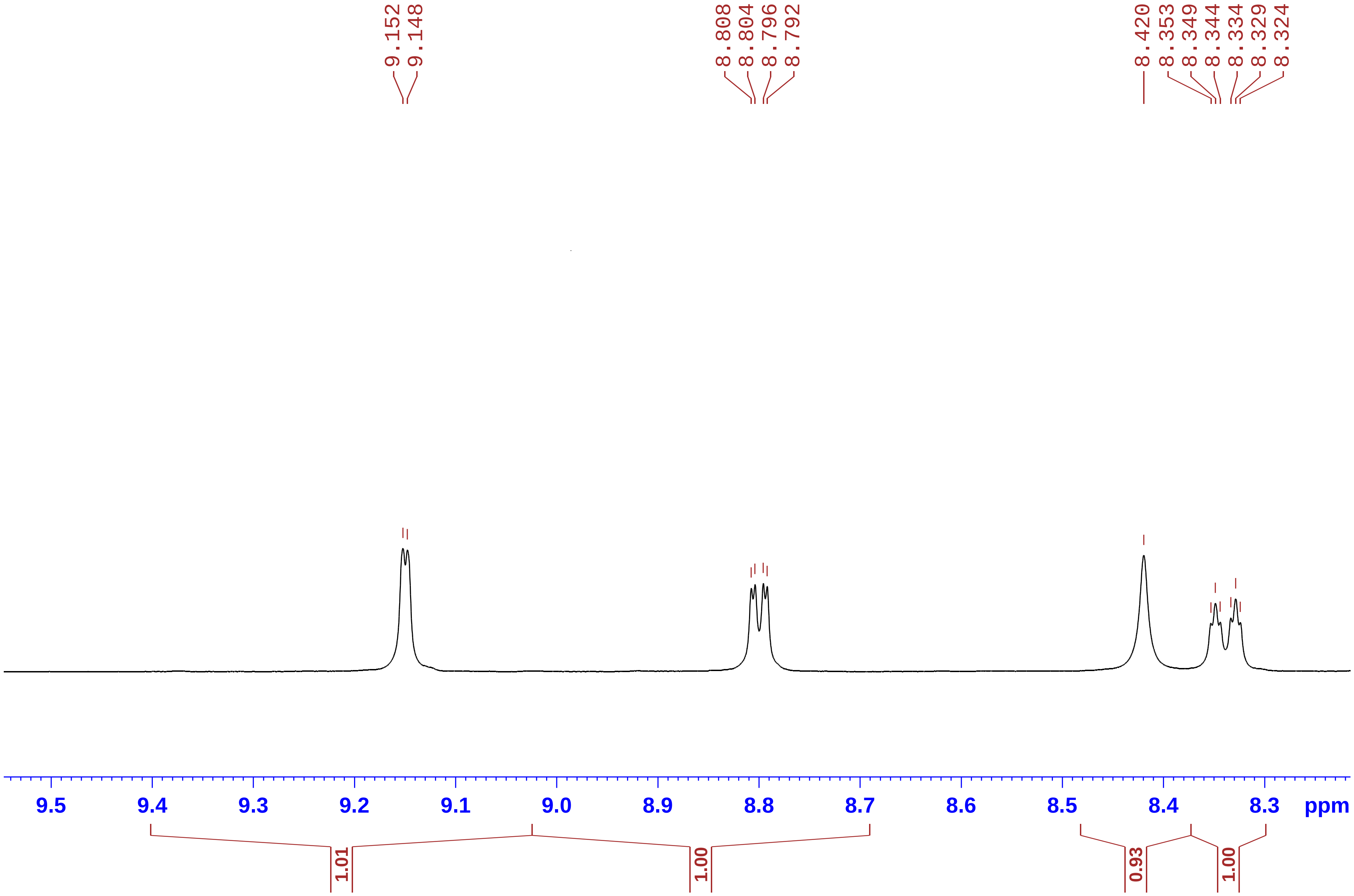

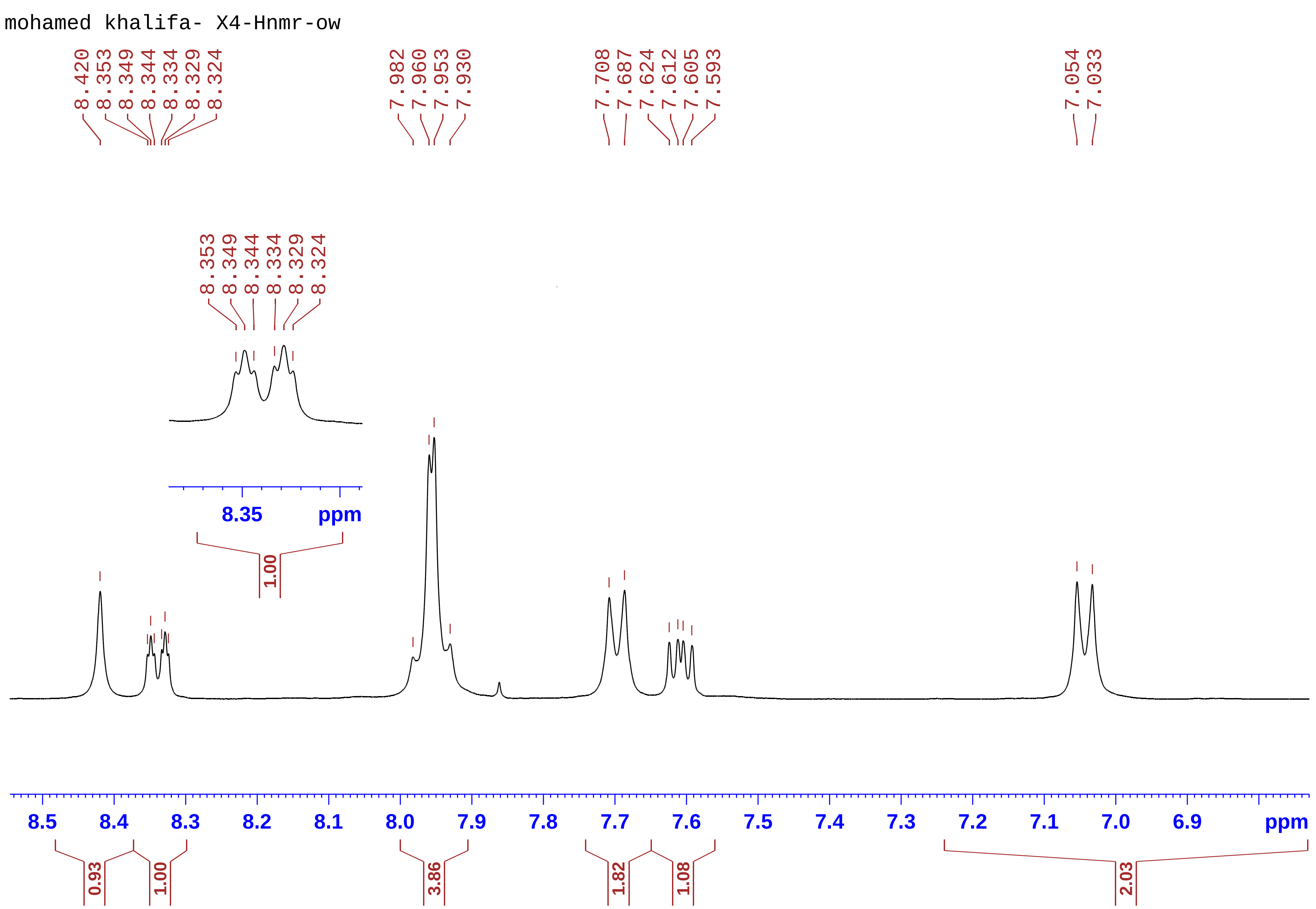
mohamed khalifa- X4-Hnmr-ow
8.420
8.353
8.349
8.344
8.334
8.329
8.324
7.982
7.960
7.953
7.930
7.708
7.687
7.624
7.612
7.605
7.593
7.054
7.033
8.353
8.349
8.344
8.334
8.329
8.324
8.35
ppm
1.00
8.5
8.4
8.3
8.2
8.1
8.0
7.9
7.8
7.7
7.6
7.5
7.4
7.3
7.2
7.1
7.0
6.9
ppm
0.93
1.00
3.86
1.82
1.08
2.03

mohamed khalifa- X4-Hnmr-ow

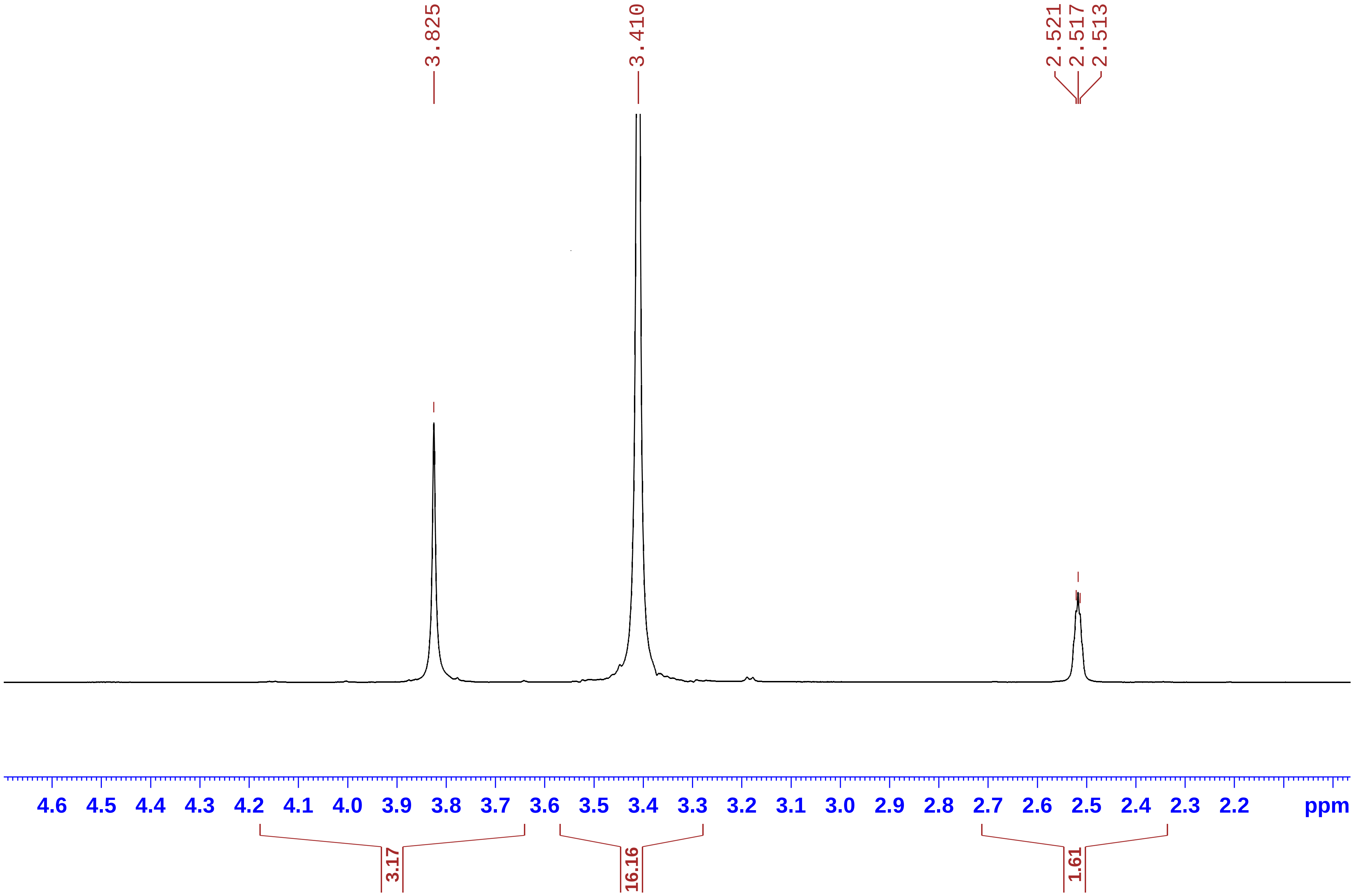

Mohamed khalifa-A1-Hnmr-ES

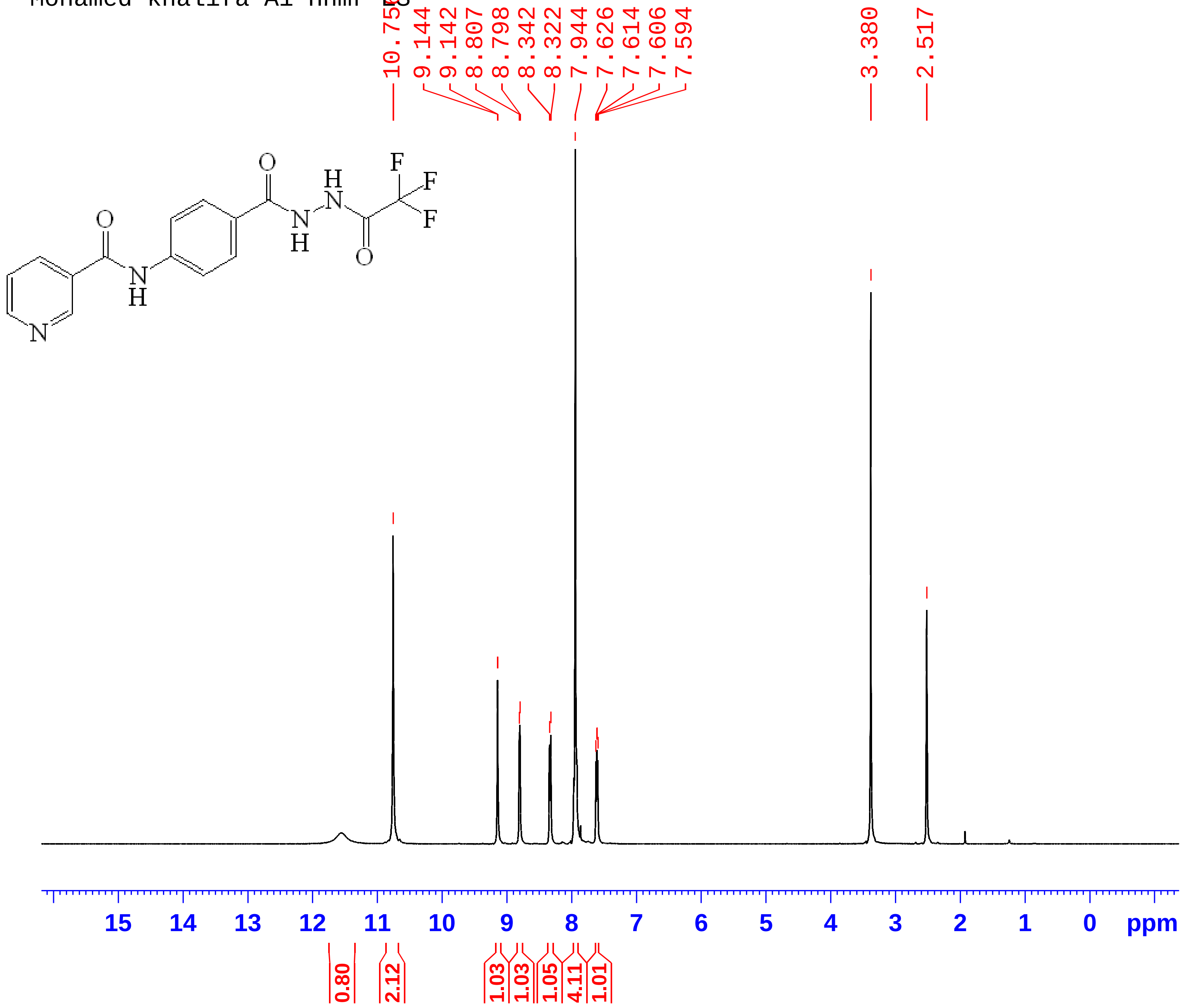

Current Data Parameters
NAME Mohamed khalifa-A1-Hnmr-ES
EXPNO 10
PROCNO 1

F2 - Acquisition Parameters
Date_ 20201126
Time 14.25 h
INSTRUM spect
PROBHD Z108618_0945 (
PULPROG zg30
TD 65536
SOLVENT DMSO
NS 16
DS 2
SWH 8012.820 Hz
FIDRES 0.244532 Hz
AQ 4.0894465 sec
RG 176.72
DW 62.400 usec
DE 6.50 usec
TE 293.2 K
D1 1.00000000 sec
TD0 1
SFO1 400.2024712 MHz
NUC1 1H
P1 13.50 usec
PLW1 13.00000000 W

F2 - Processing parameters
SI 65536
SF 400.2000000 MHz
WDW EM
SSB 0
LB 0.30 Hz
GB 0
PC 1.00

Mohamed khalifa-A1-Hnmr-ES

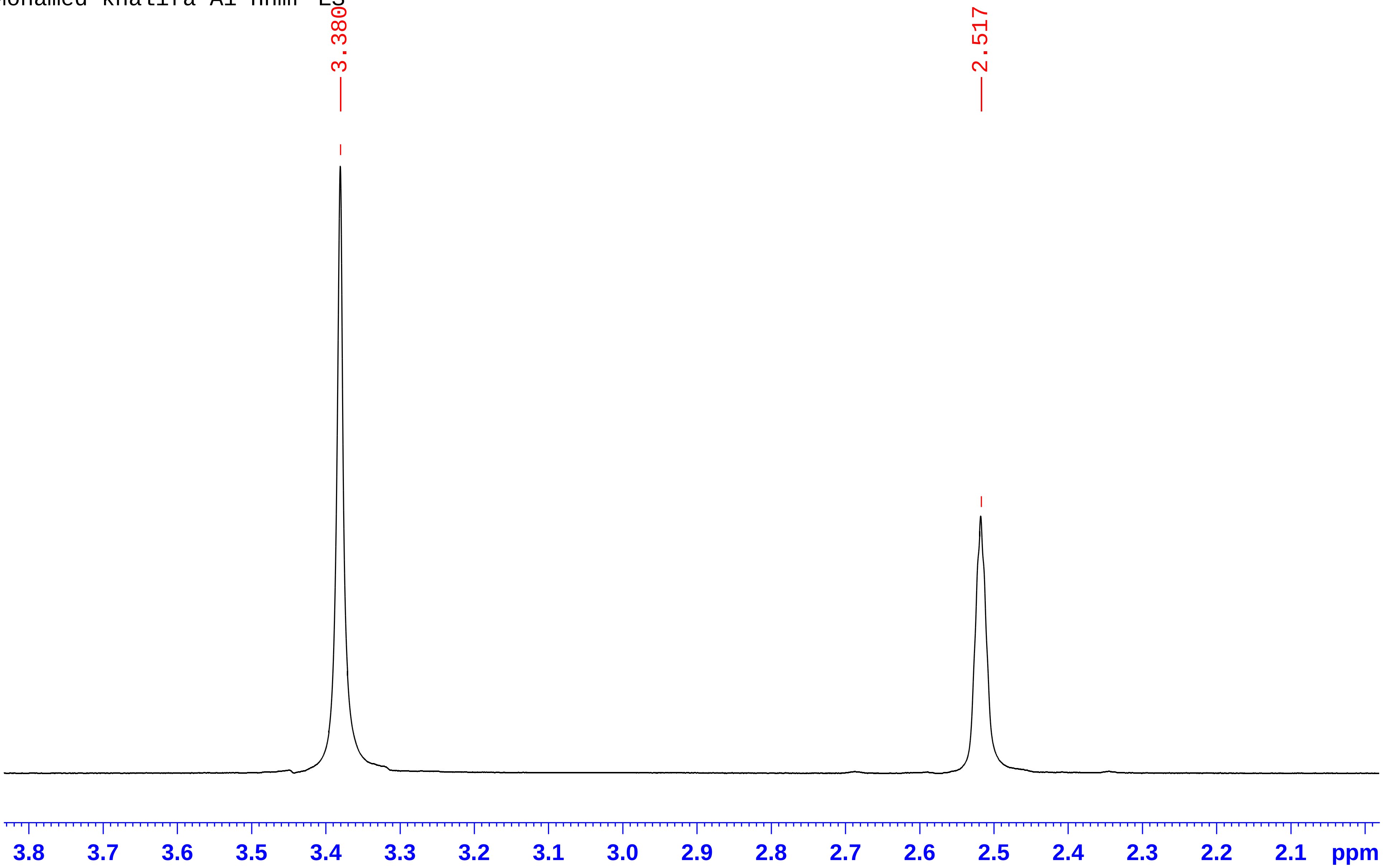

Mohamed khalifa-A1-Hnmr-ES

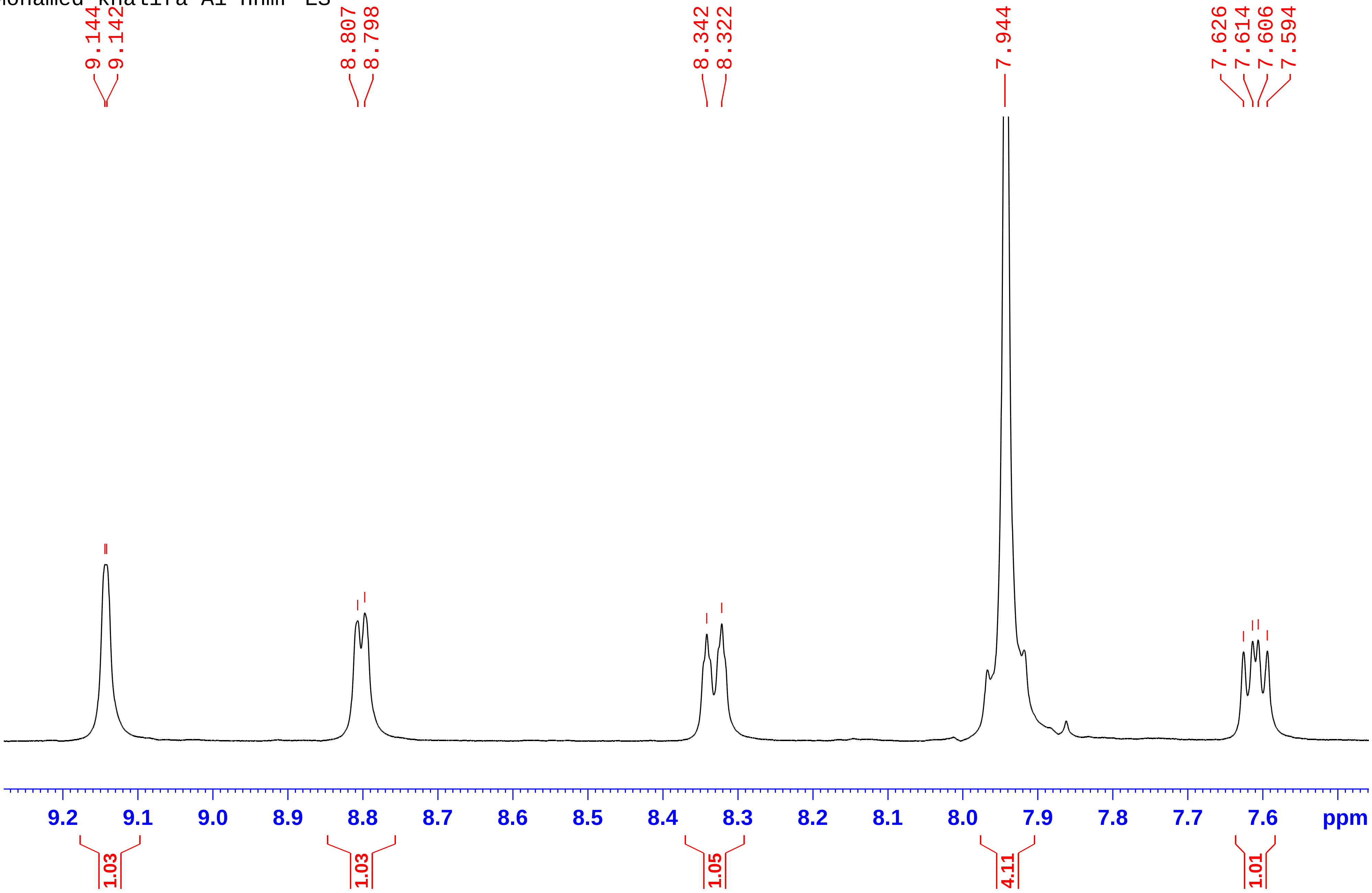

Mohamed khalifa-A1-Hnmr-ES

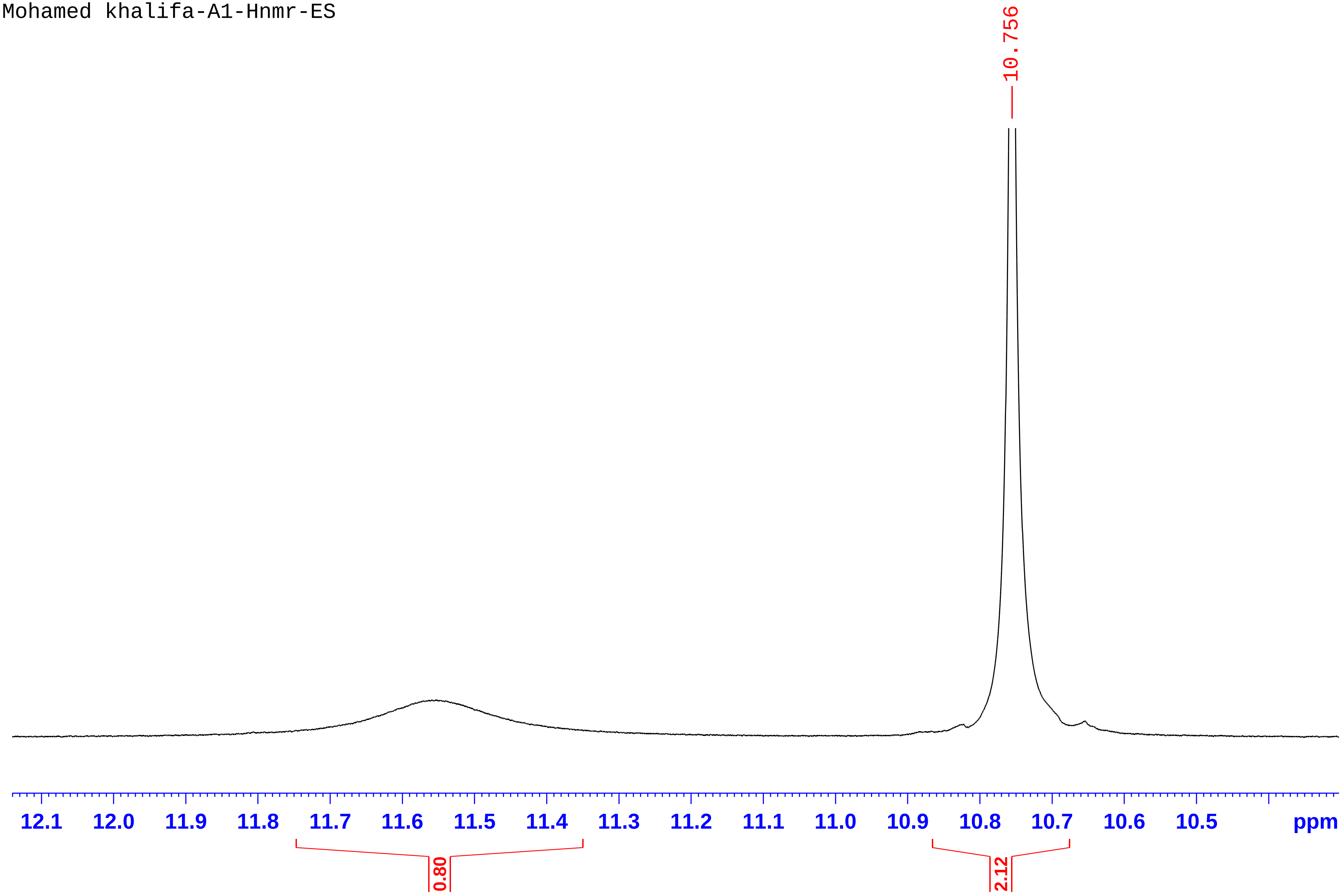

Mohamed khalifa-H4-Hnmr-ES

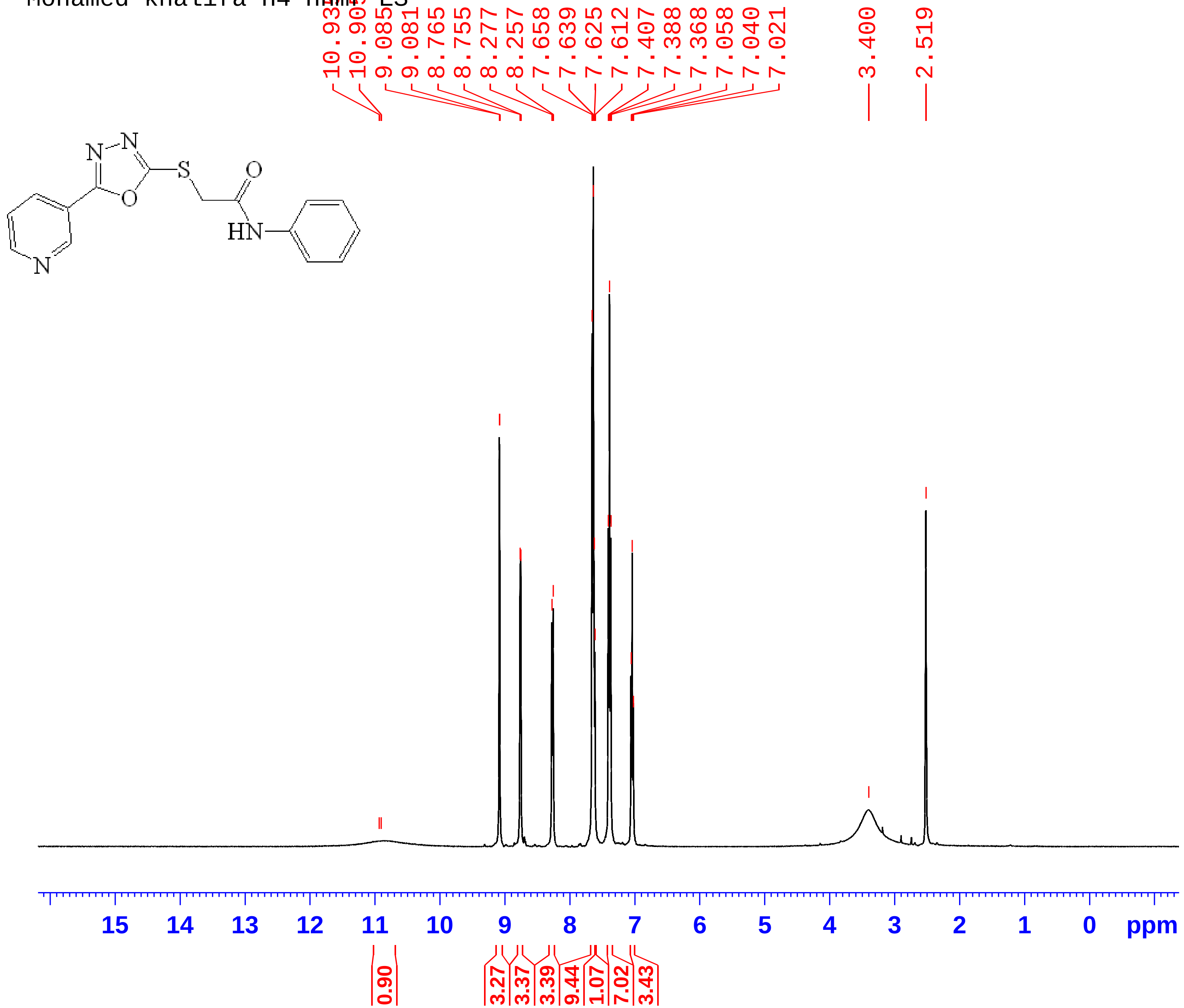

Current Data Parameters

NAME	Mohamed khalifa-H4-Hnmr-ES
EXPNO	10
PROCNO	1

F2 - Acquisition Parameters

Date_	20201126
Time	14.30 h
INSTRUM	spect
PROBHD	Z108618_0945 (
PULPROG	zg30
TD	65536
SOLVENT	DMSO
NS	16
DS	2
SWH	8012.820 Hz
FIDRES	0.244532 Hz
AQ	4.0894465 sec
RG	135.42
DW	62.400 usec
DE	6.50 usec
TE	293.2 K
D1	1.00000000 sec
TD0	1
SFO1	400.2024712 MHz
NUC1	1H
P1	13.50 usec
PLW1	13.00000000 W

F2 - Processing parameters

SI	65536
SF	400.2000000 MHz
WDW	EM
SSB	0
LB	0.30 Hz
GB	0
PC	1.00

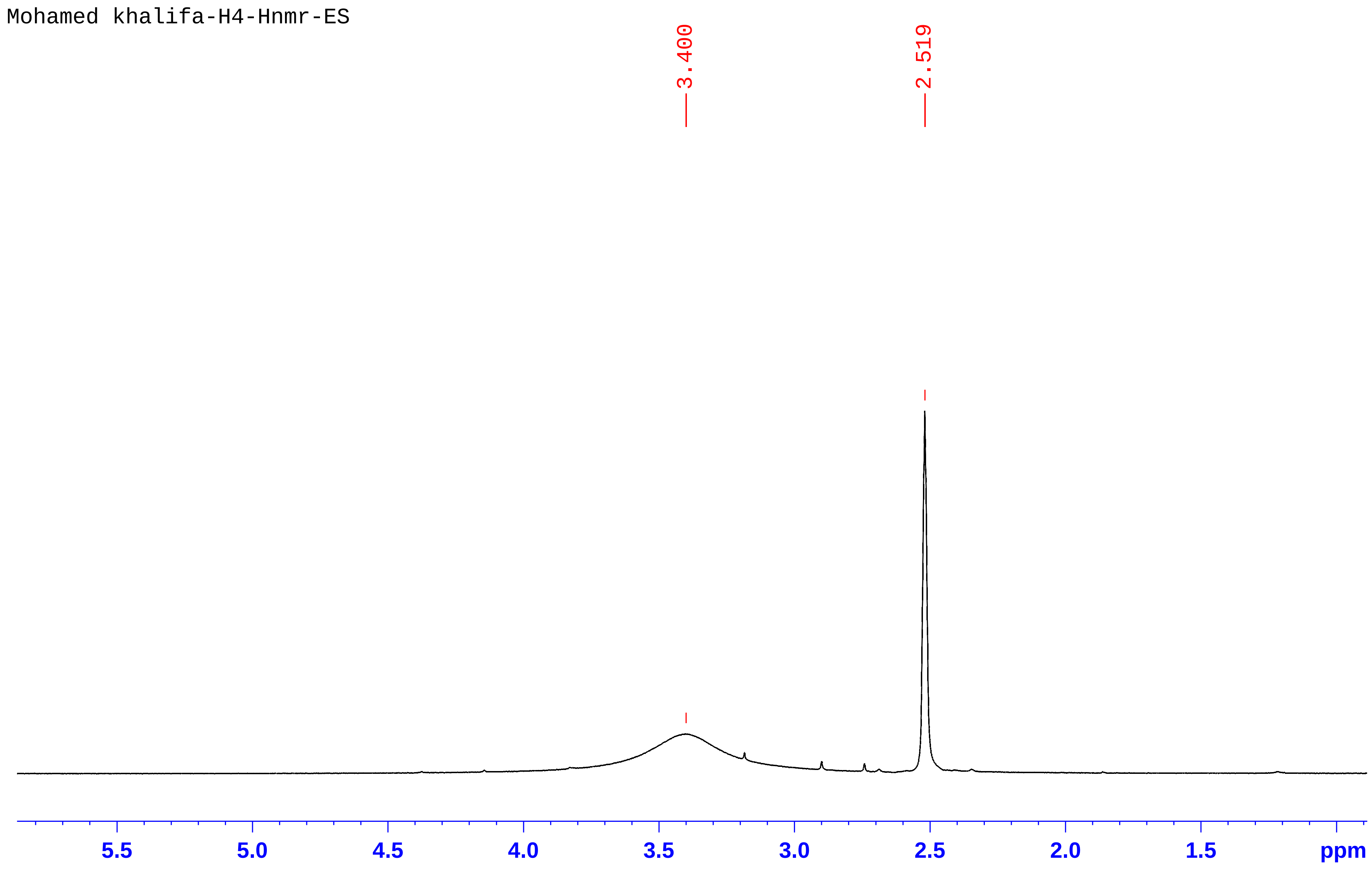
Mohamed khalifa-H4-Hnmr-ES
3.400
2.519
5.5
5.0
4.5
4.0
3.5
3.0
2.5
2.0
1.5
ppm

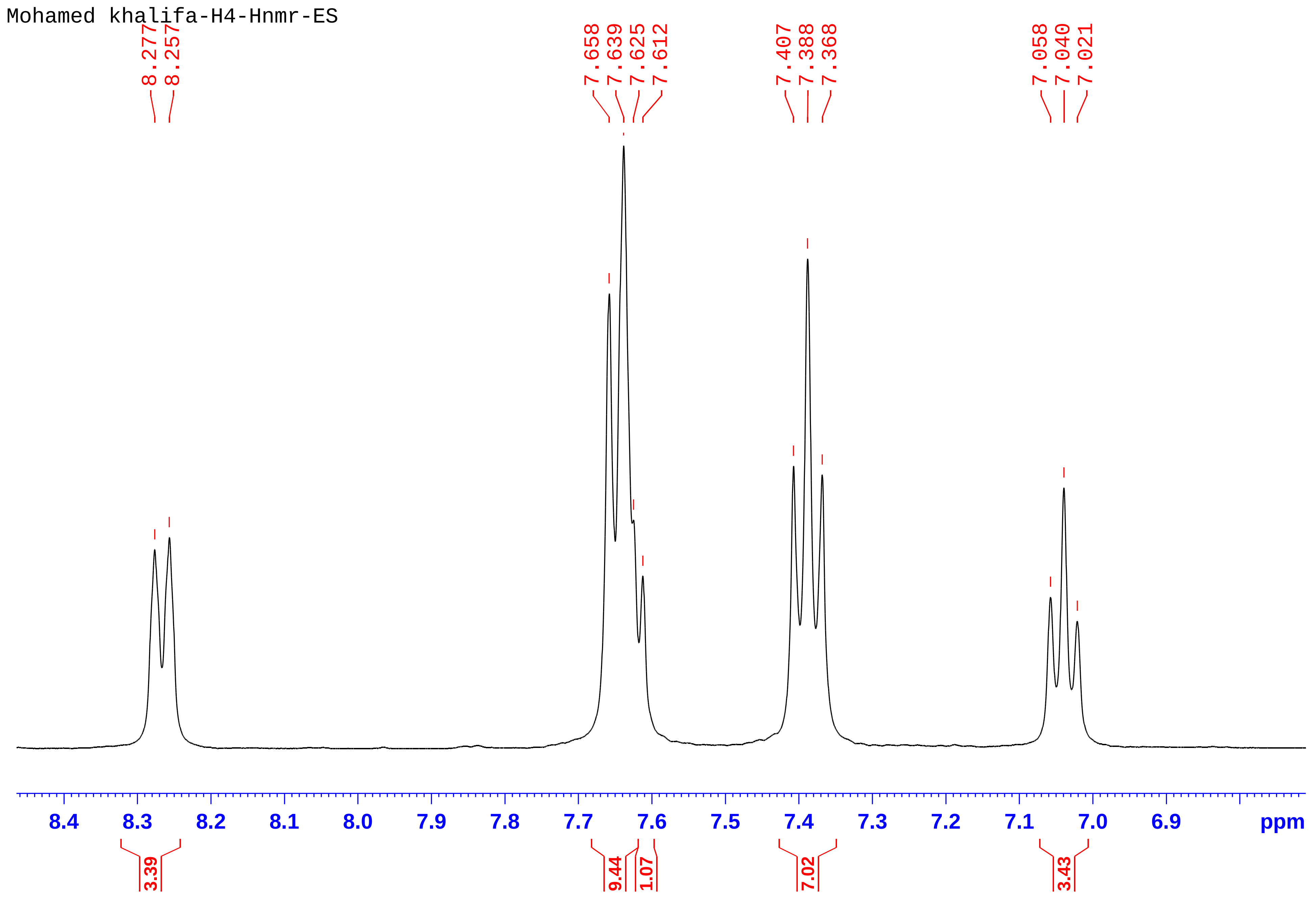
Mohamed khalifa-H4-Hnmr-ES
8.277
8.257
7.658
7.639
7.625
7.612
7.407
7.388
7.368
7.058
7.040
7.021
8.4
8.3
8.2
8.1
8.0
7.9
7.8
7.7
7.6
7.5
7.4
7.3
7.2
7.1
7.0
6.9
ppm
3.39
9.44
1.07
7.02
3.43

Mohamed khalifa-H4-Hnmr-ES

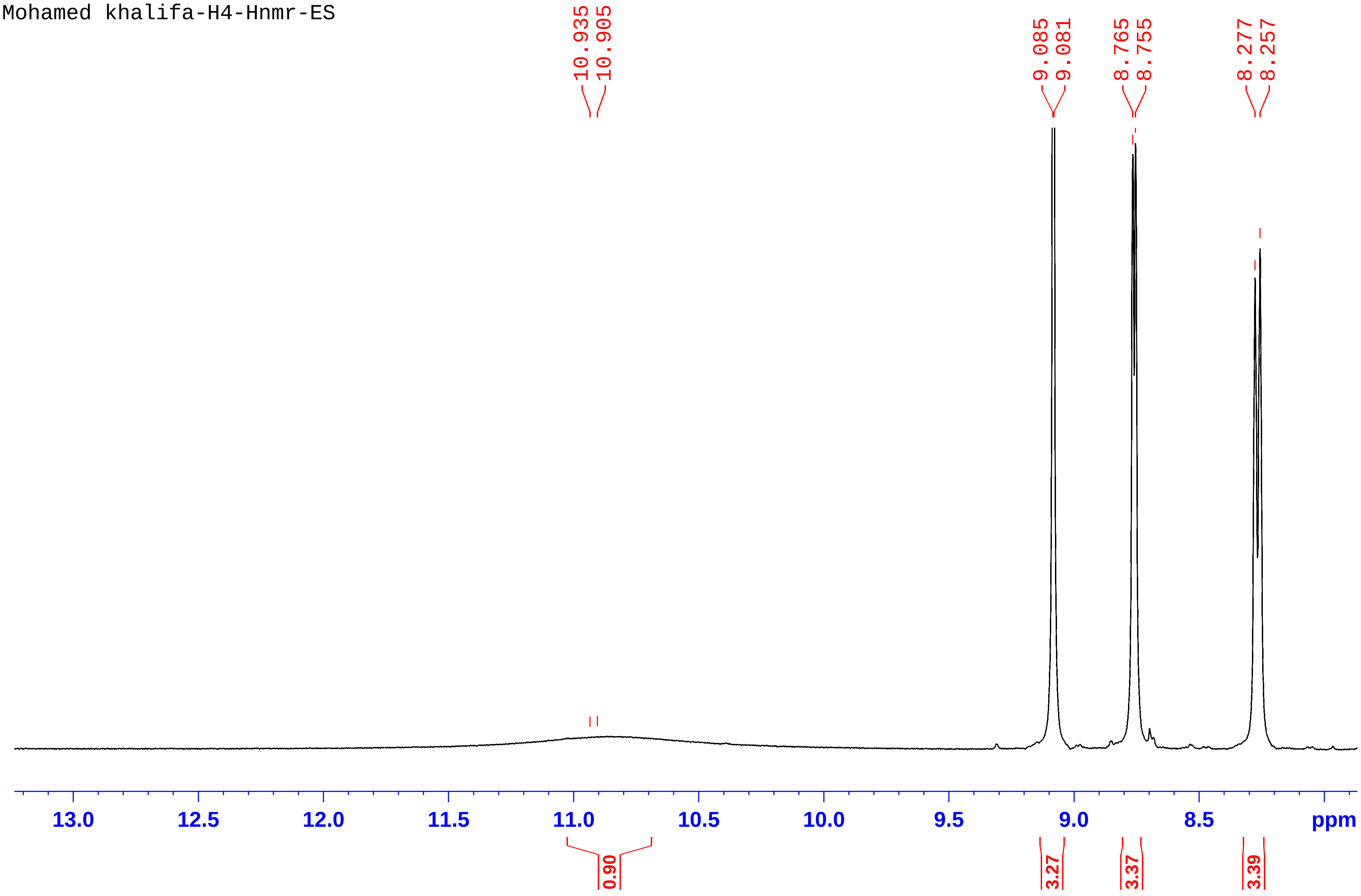

Mohamed khalifa-H4-Hnmr-ES

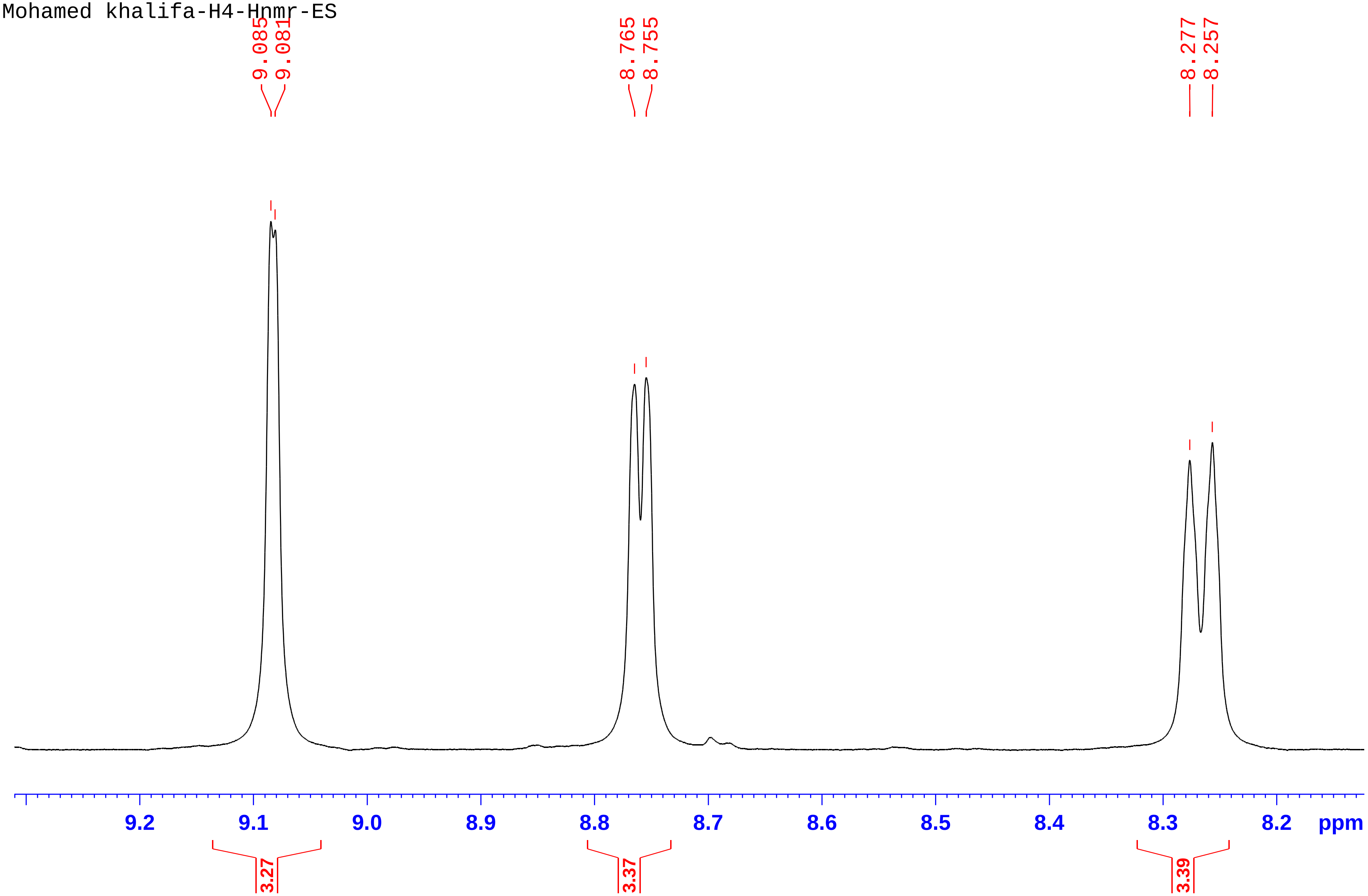

Mohamed khalifa-H7-Hnmr-ES

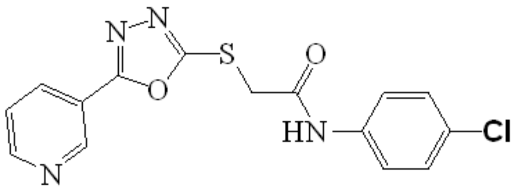

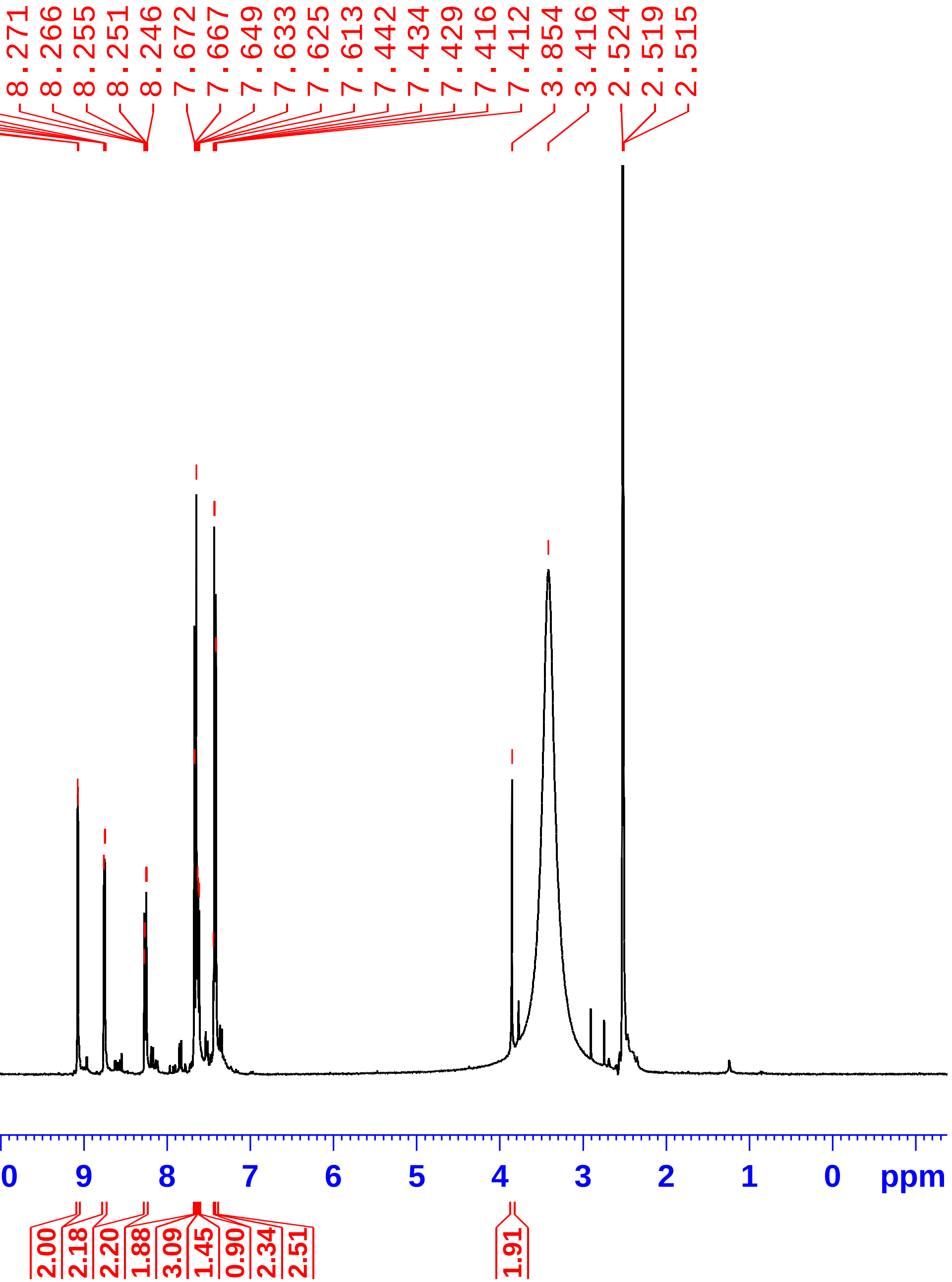

Faculty of Pharmacy

Current Data Parameters
NAME Mohamed khalifa-H7-Hnmr-ES
EXPNO 10
PROCNO 1

F2 - Acquisition Parameters
Date_ 20201126
Time 14.34 h
INSTRUM spect
PROBHD Z108618_0945 (
PULPROG zg30
TD 65536
SOLVENT DMSO
NS 16
DS 2
SWH 8012.820 Hz
FIDRES 0.244532 Hz
AQ 4.0894465 sec
RG 176.72
DW 62.400 usec
DE 6.50 usec
TE 293.2 K
D1 1.00000000 sec
TD0 1
SFO1 400.2024712 MHz
NUC1 1H
P1 13.50 usec
PLW1 13.00000000 W

F2 - Processing parameters
SI 65536
SF 400.2000000 MHz
WDW EM
SSB 0
LB 0.30 Hz
GB 0
PC 1.00

Mohamed khalifa-H7-Hnmr-ES

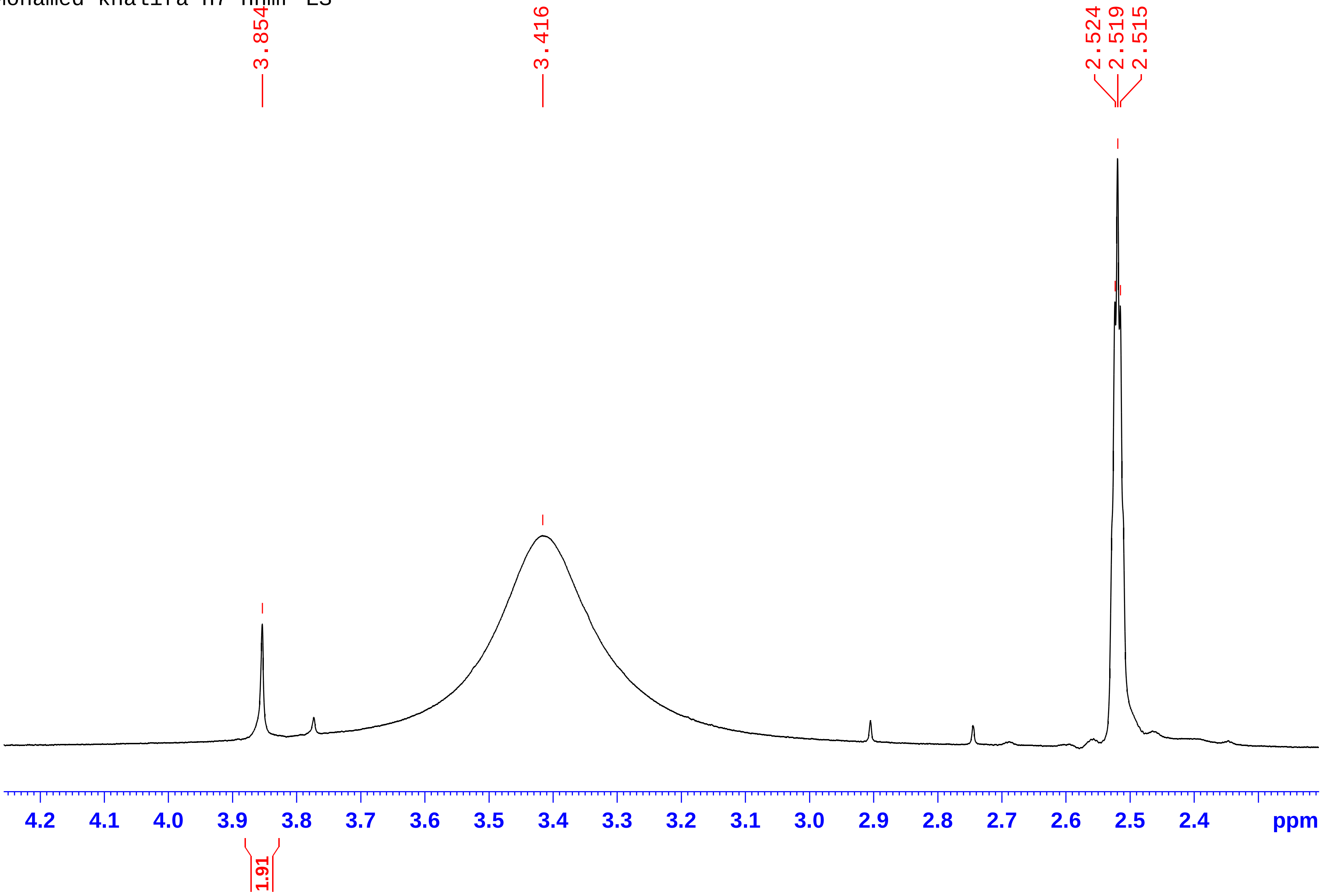

Mohamed khalifa-H7-Hnmr-ES

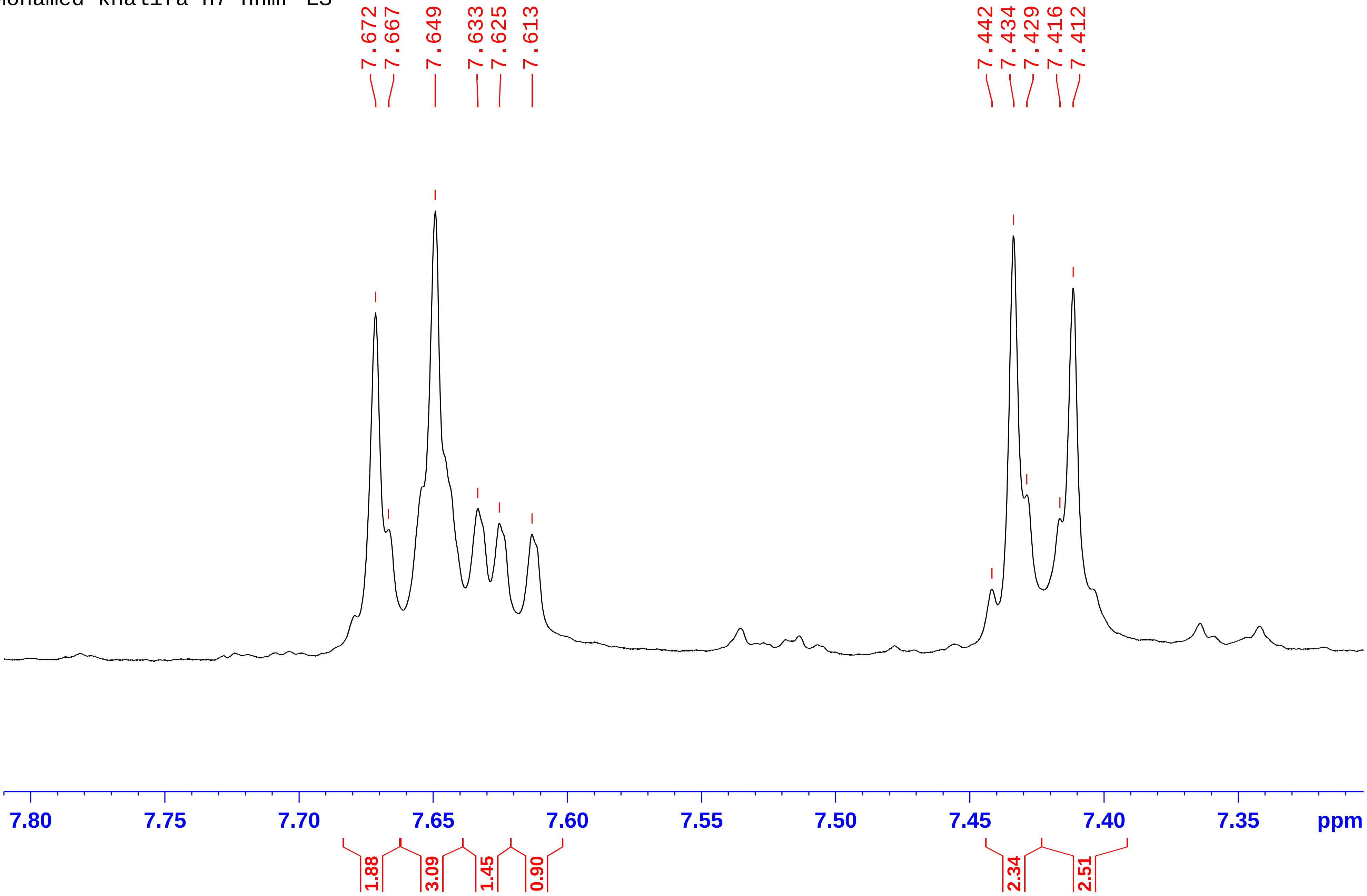

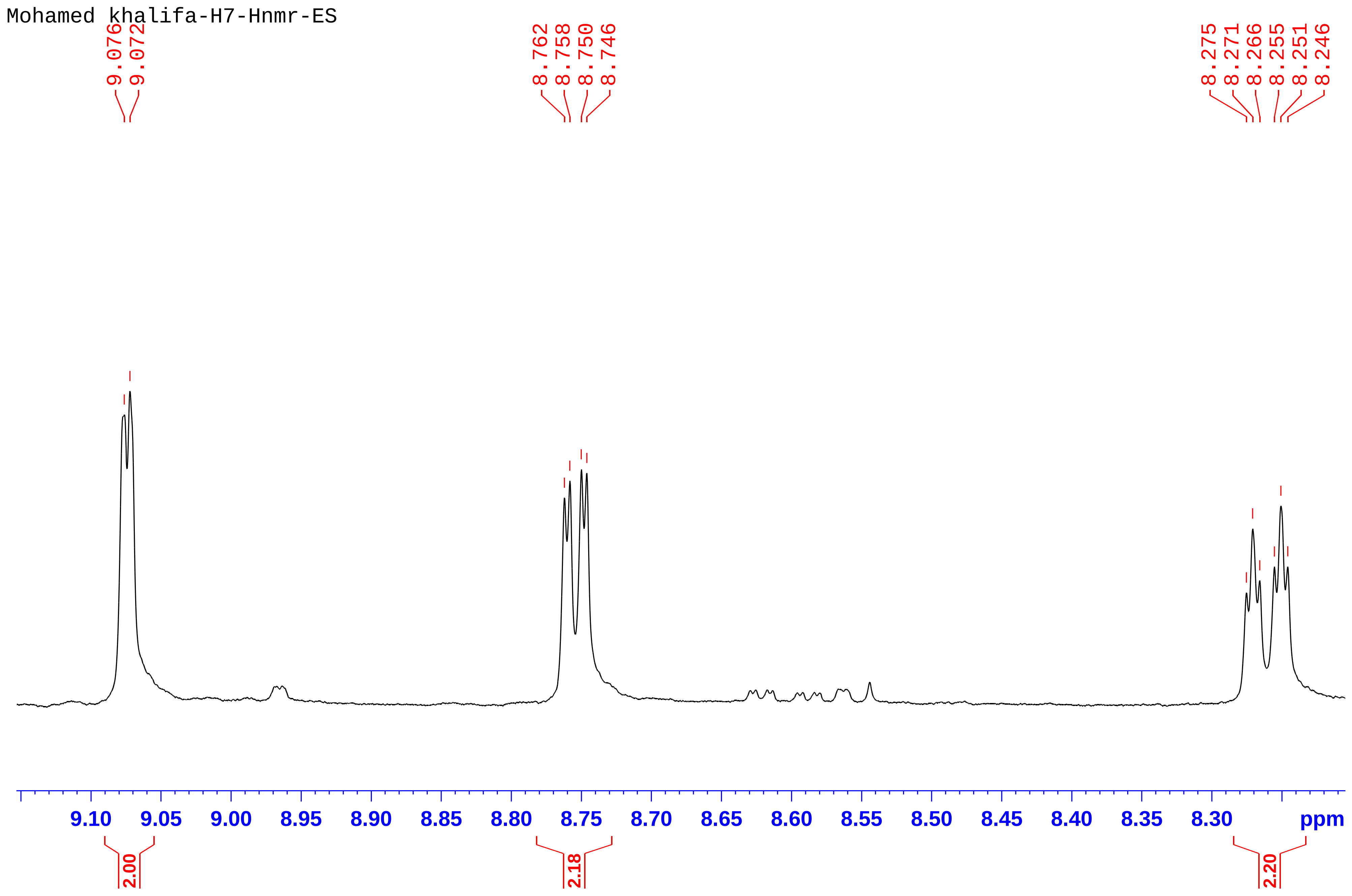

Mohamed khalifa-H7-Hnmr-ES
9.076
9.072
8.762
8.758
8.750
8.746
8.275
8.271
8.266
8.255
8.251
8.246
9.10
9.05
9.00
8.95
8.90
8.85
8.80
8.75
8.70
8.65
8.60
8.55
8.50
8.45
8.40
8.35
8.30
ppm
2.00
2.18
2.20

Mohamed khalifa-R1-Hnmr-ES

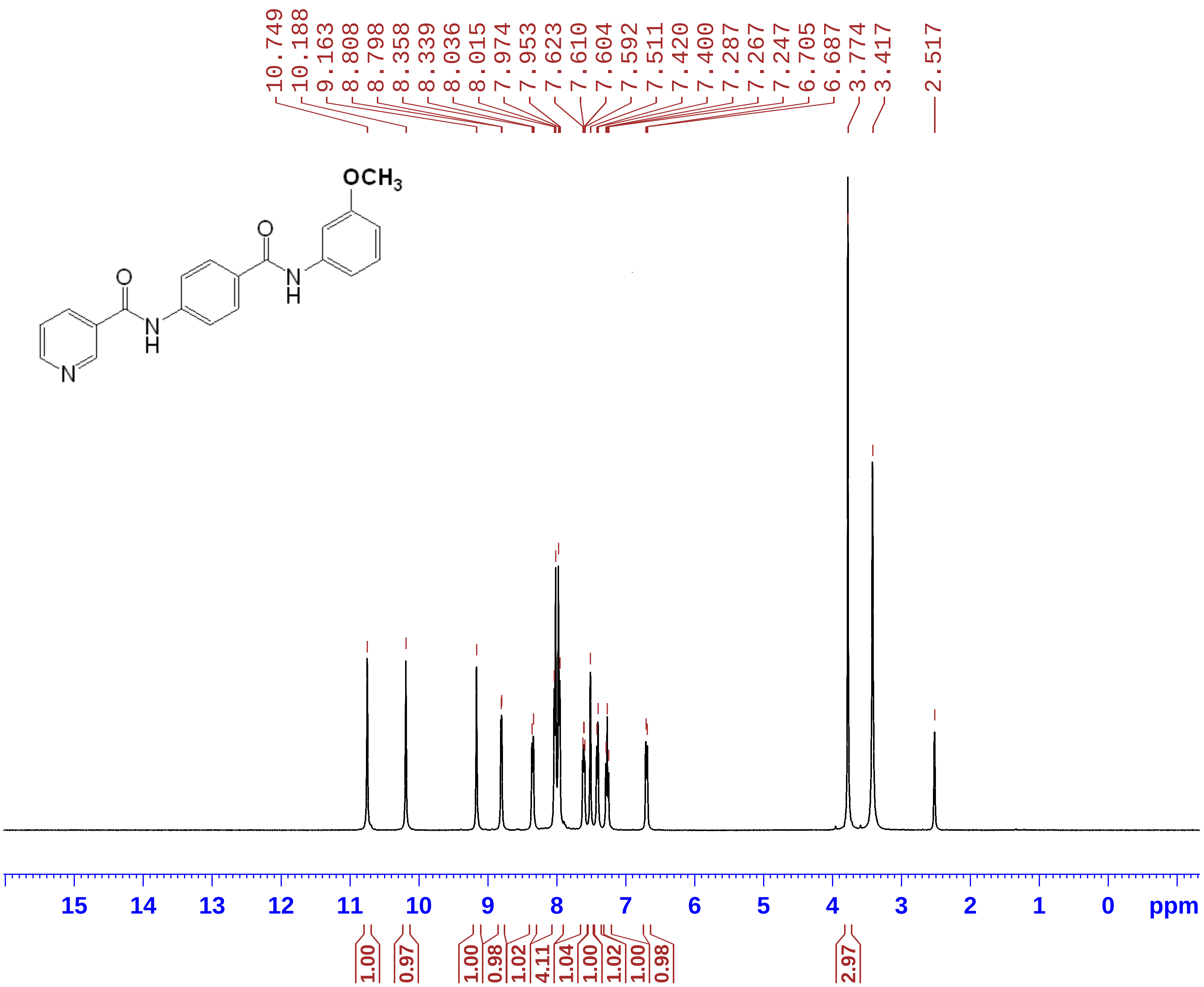

Current Data Parameters
NAME Mohamed khalifa-R1-
EXPNO 50
PROCNO 1

F2 - Acquisition Parameters
Date_ 20201126
Time 12.47 h
INSTRUM spect
PROBHD Z108618_0945 (
PULPROG zg30
TD 65536
SOLVENT DMSO
NS 16
DS 2
SWH 8012.820 Hz
FIDRES 0.244532 Hz
AQ 4.0894465 sec
RG 78.59
DW 62.400 usec
DE 6.50 usec
TE 293.1 K
D1 1.00000000 sec
TD0 1
SFO1 400.2024712 MHz
NUC1 1H
P1 13.50 usec
PLW1 13.00000000 W

F2 - Processing parameters
SI 65536
SF 400.2000000 MHz
WDW EM
SSB 0
LB 0.30 Hz
GB 0
PC 1.00

Mohamed khalifa-R1-Hnmr-ES

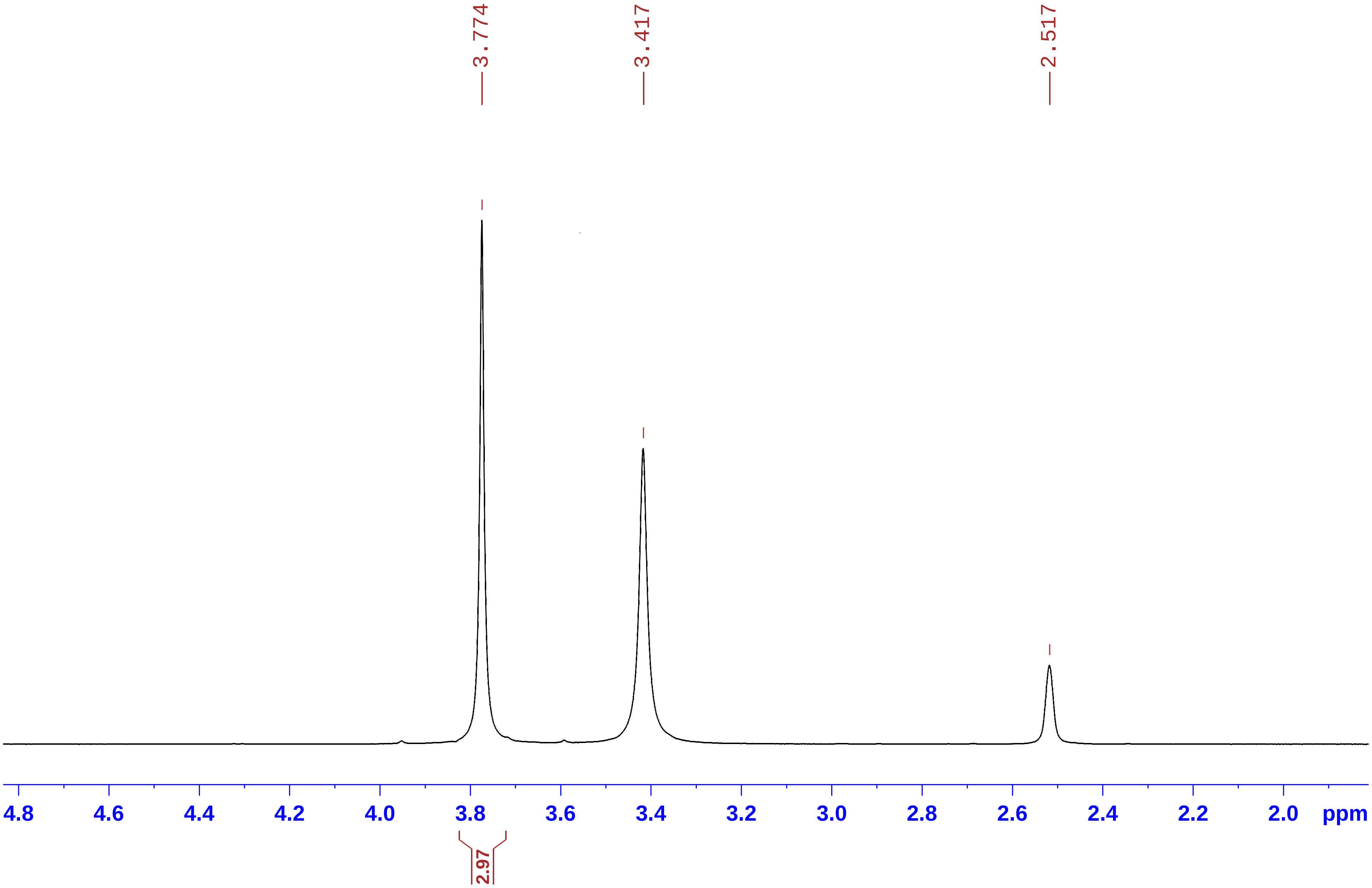

Mohamed khalifa-R1-Hnmr-ES

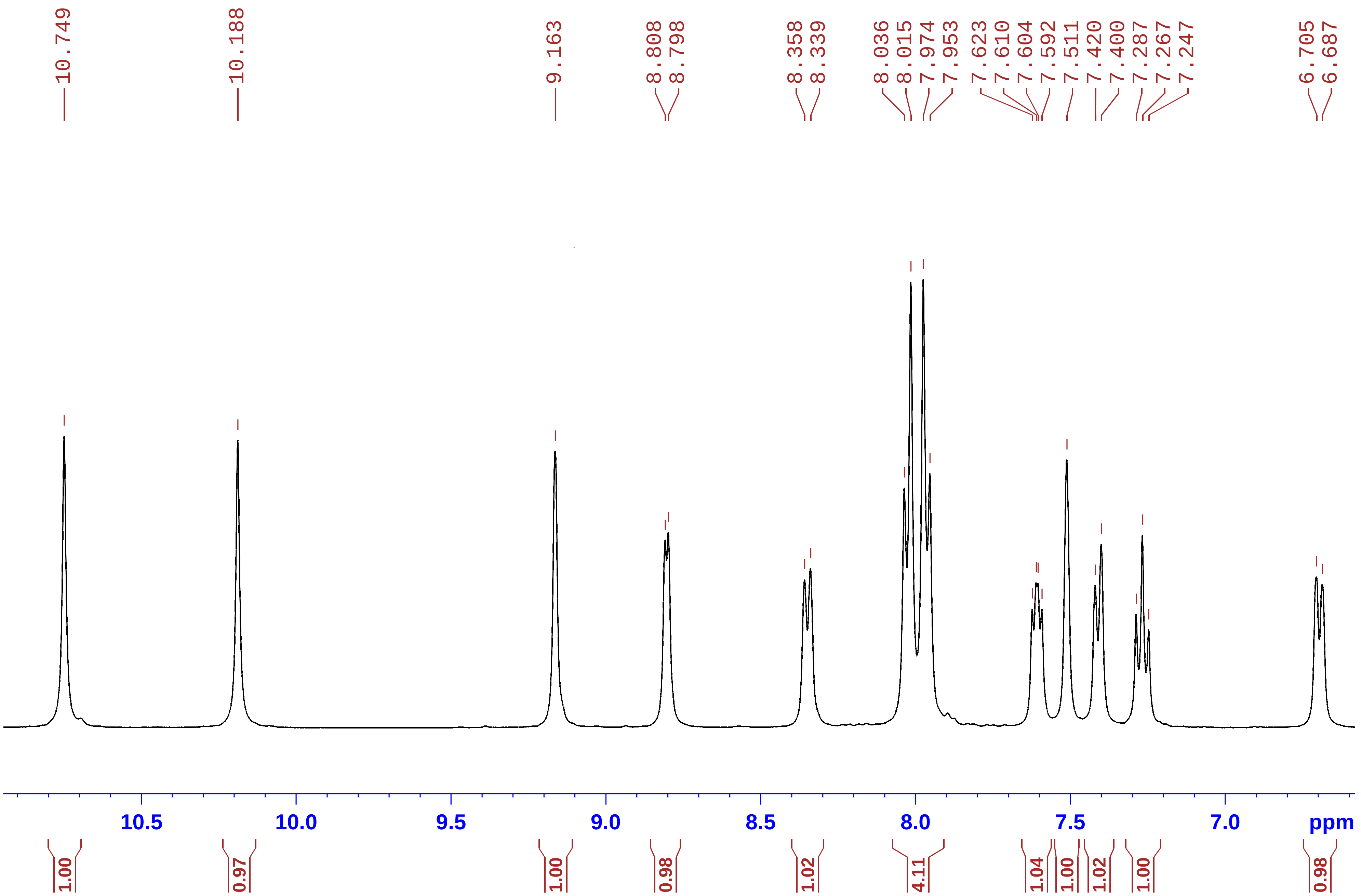

Mohamed khalifa-R2-Hnmr-ES

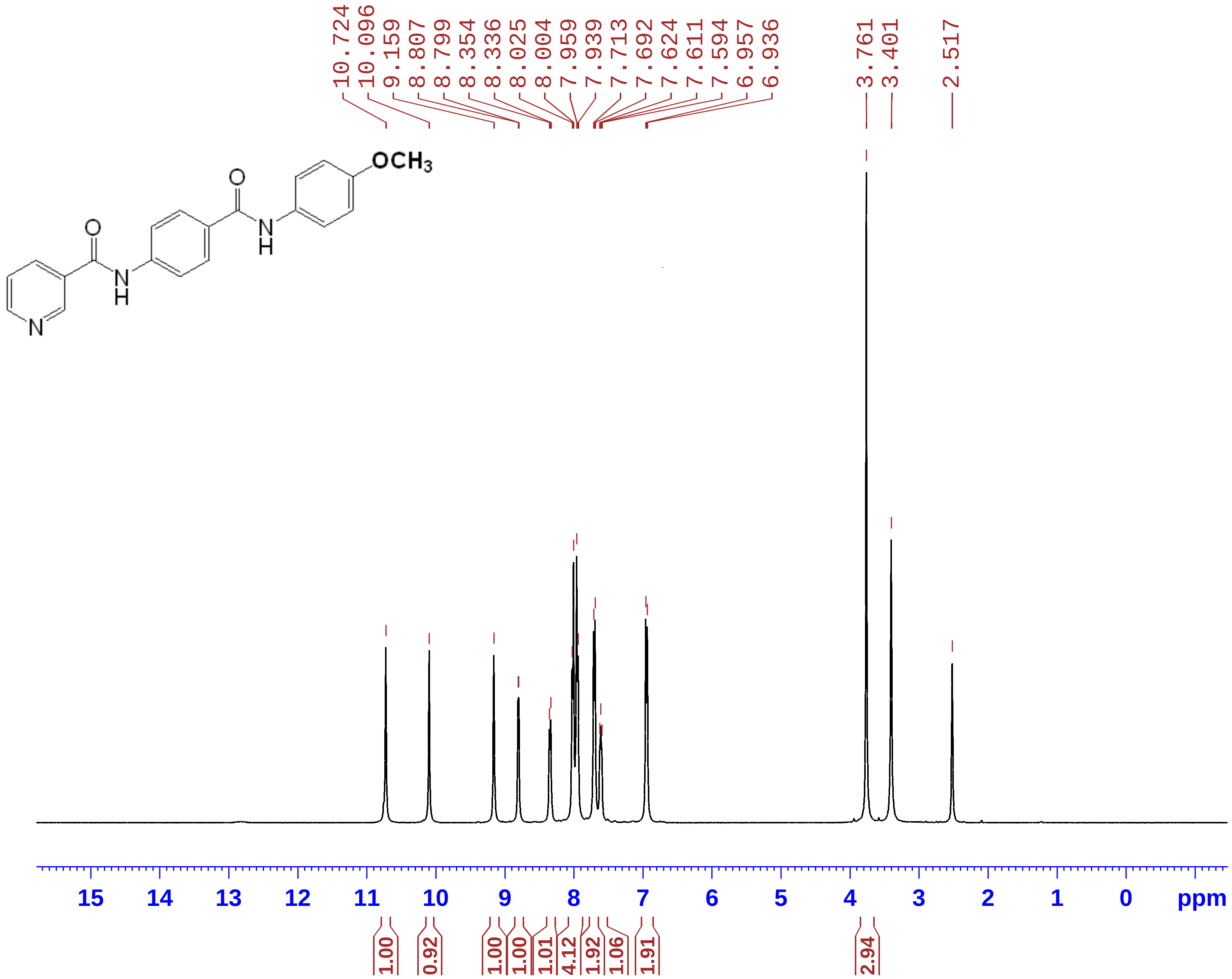

BRUKER

Faculty of Pharmacy

```
Current Data Parameters
NAME        Mohamed khalifa-R2-
EXPNO                        20
PROCNO                        1

F2 - Acquisition Parameters
Date_                  20201126
Time                      12.51 h
INSTRUM                   spect
PROBHD       Z108618_0945 (
PULPROG                    zg30
TD                        65536
SOLVENT                    DMSO
NS                           16
DS                            2
SWH                    8012.820 Hz
FIDRES                 0.244532 Hz
AQ                    4.0894465 sec
RG                       112.56
DW                       62.400 usec
DE                         6.50 usec
TE                        293.1 K
D1                   1.00000000 sec
TD0                           1
SFO1                400.2024712 MHz
NUC1                         1H
P1                        13.50 usec
PLW1                13.00000000 W

F2 - Processing parameters
SI                        65536
SF                  400.2000000 MHz
WDW                          EM
SSB          0
LB                         0.30 Hz
GB           0
PC                         1.00
```

Mohamed khalifa-R2-Hnmr-ES

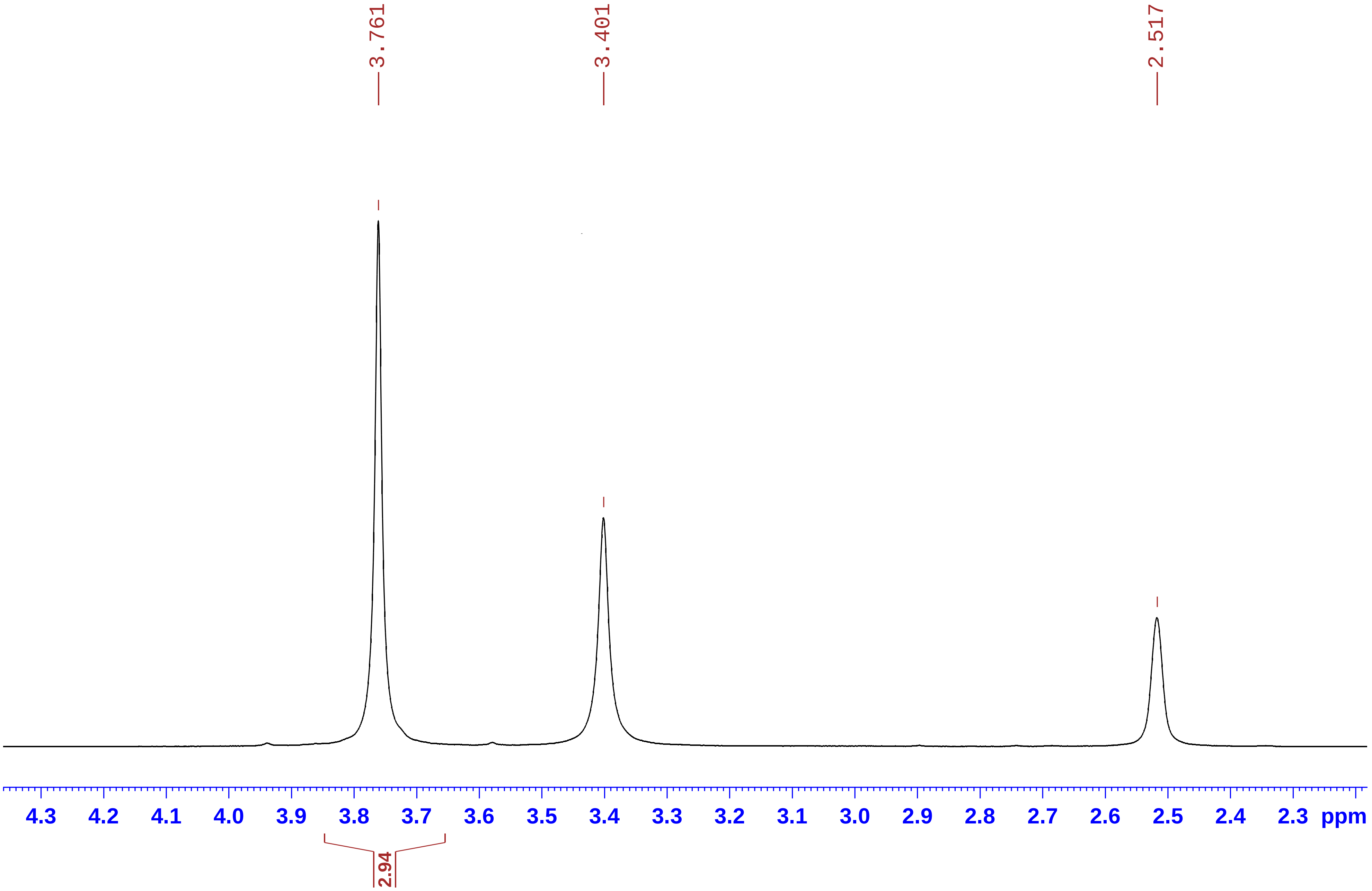

Mohamed khalifa-R2-Hnmr-ES

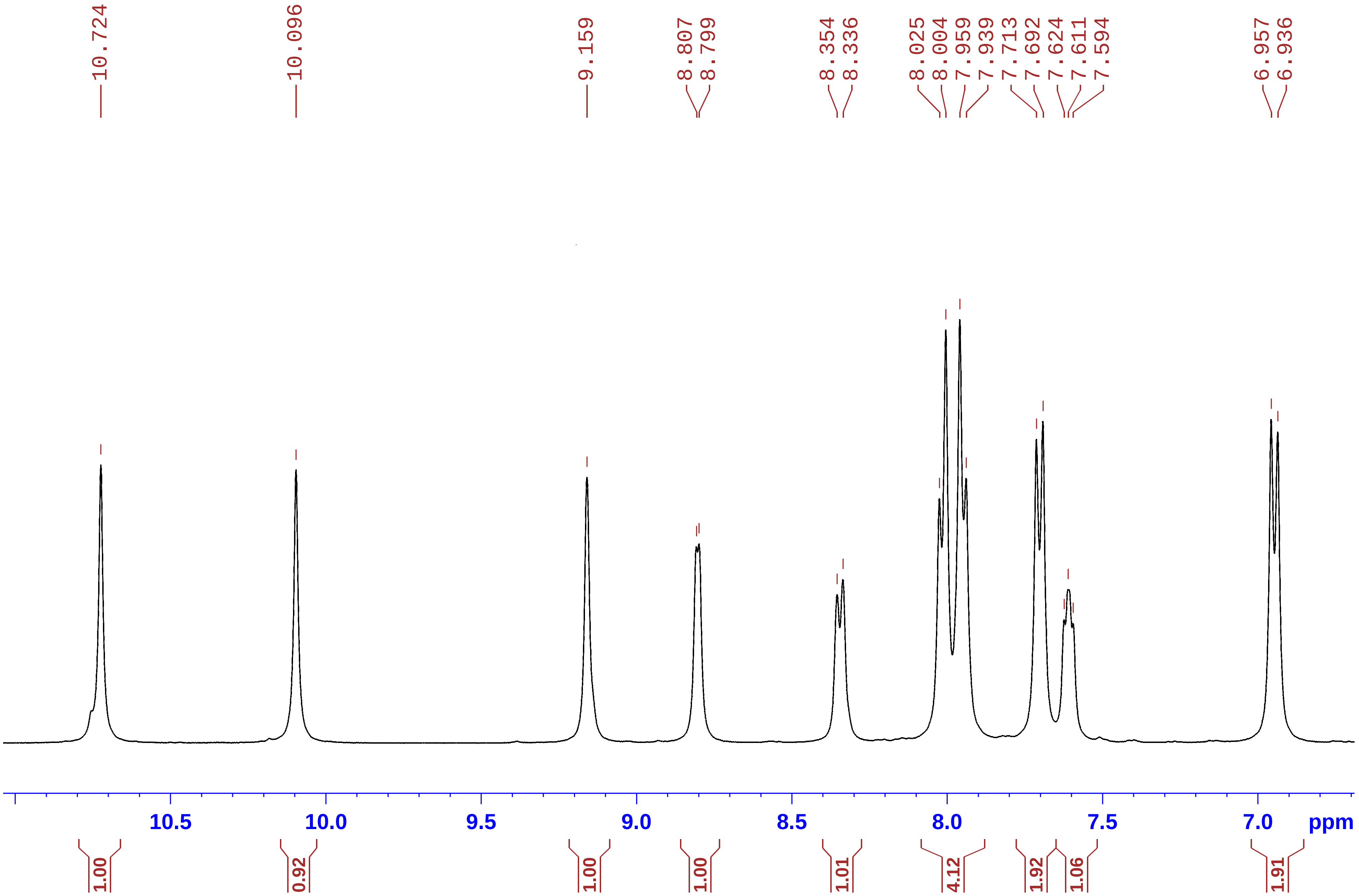

Mohamed khalifa-R3-Hnmr-ES

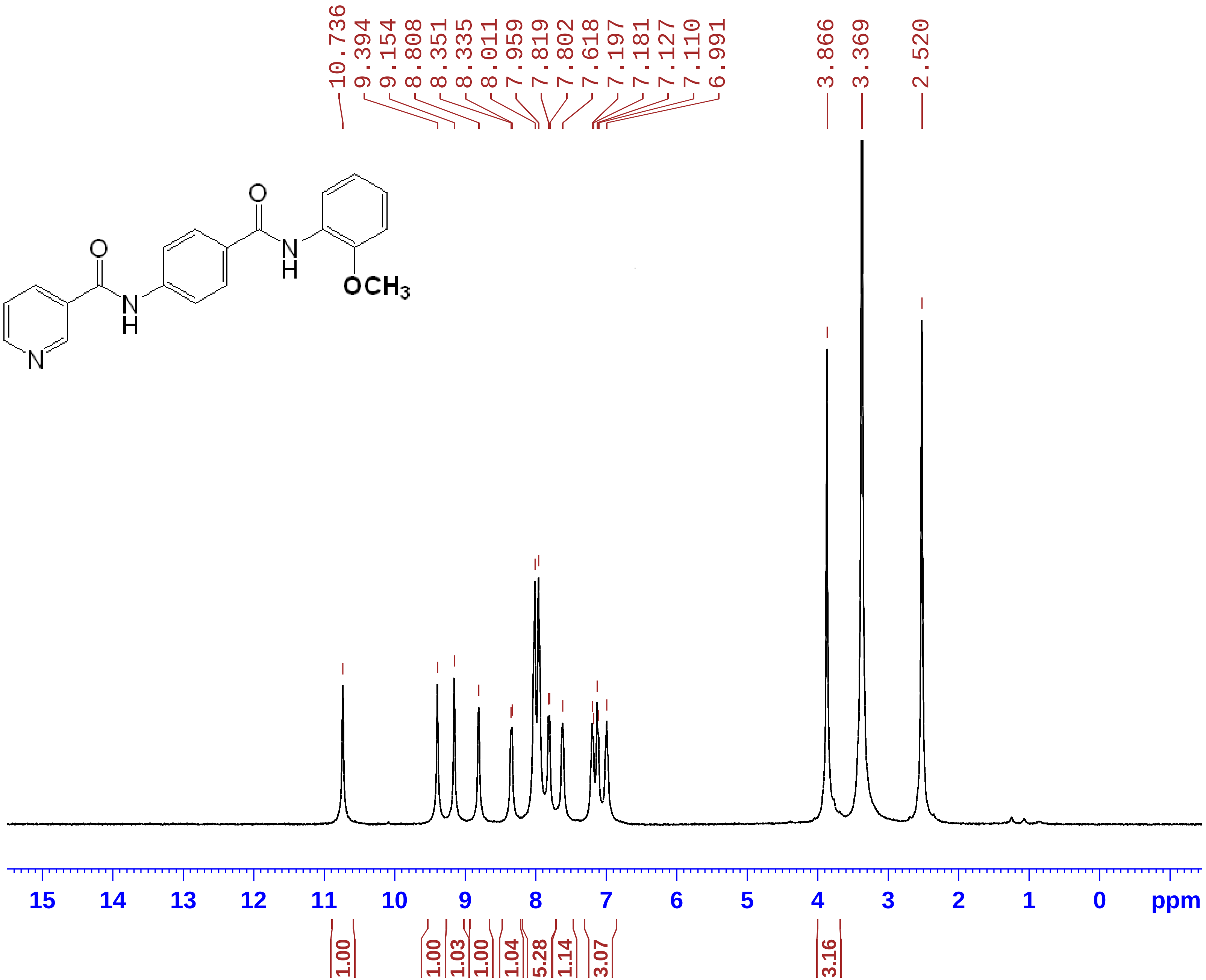


```
Current Data Parameters
NAME      Mohamed khalifa-R3-
EXPNO                    20
PROCNO                    1

F2 - Acquisition Parameters
Date_              20201126
Time                  12.57 h
INSTRUM               spect
PROBHD   Z108618_0945 (
PULPROG                zg30
TD                    65536
SOLVENT                DMSO
NS                       16
DS                        2
SWH                8012.820 Hz
FIDRES             0.244532 Hz
AQ                4.0894465 sec
RG                   197.77
DW                   62.400 usec
DE                     6.50 usec
TE                    293.3 K
D1               1.00000000 sec
TD0                       1
SFO1            400.2024712 MHz
NUC1                     1H
P1                    13.50 usec
PLW1            13.00000000 W

F2 - Processing parameters
SI                    65536
SF              400.2000000 MHz
WDW                      EM
SSB      0
LB                     0.30 Hz
GB       0
PC                     1.00
```

Mohamed khalifa-R3-Hnmr-ES

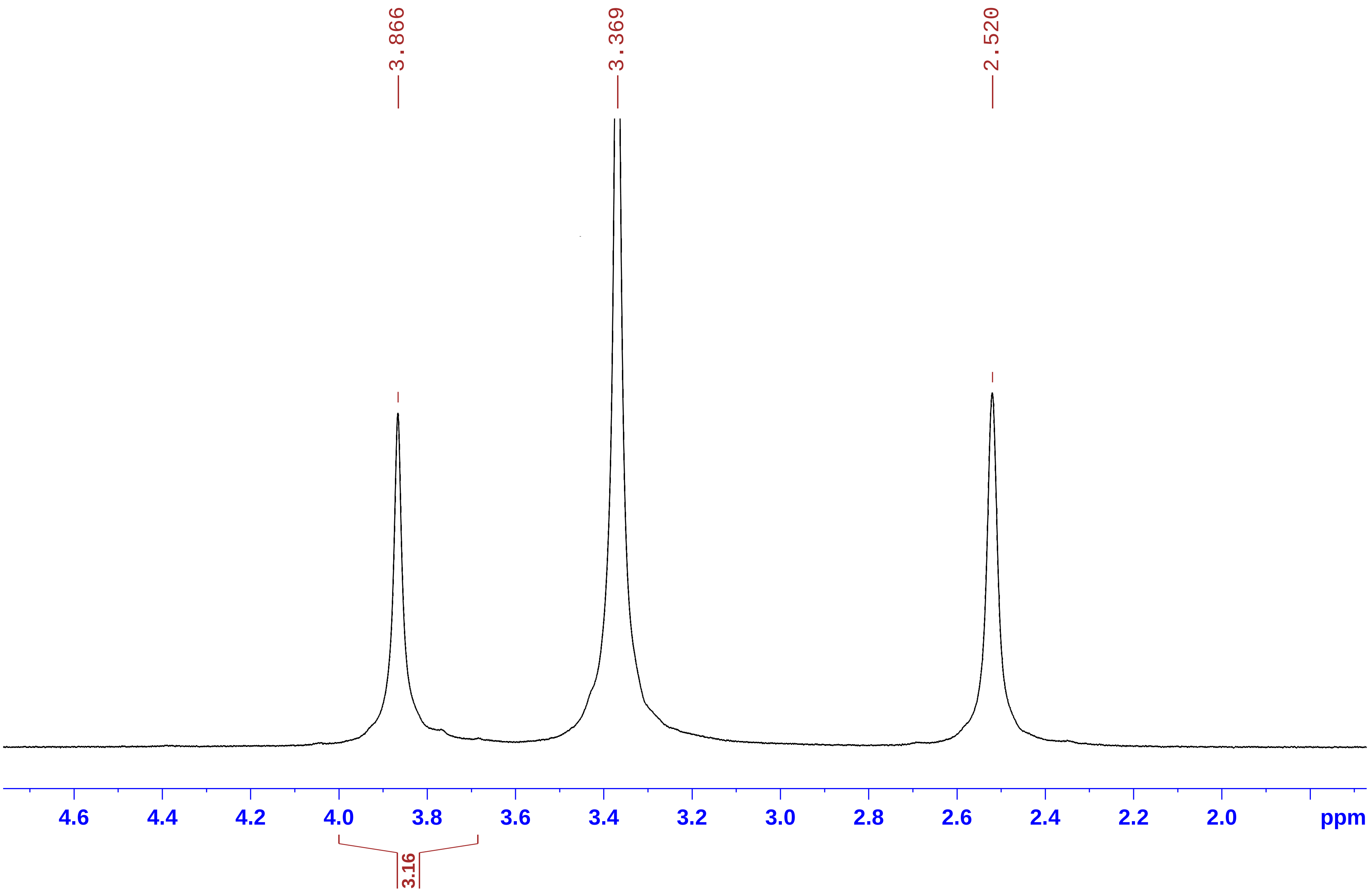

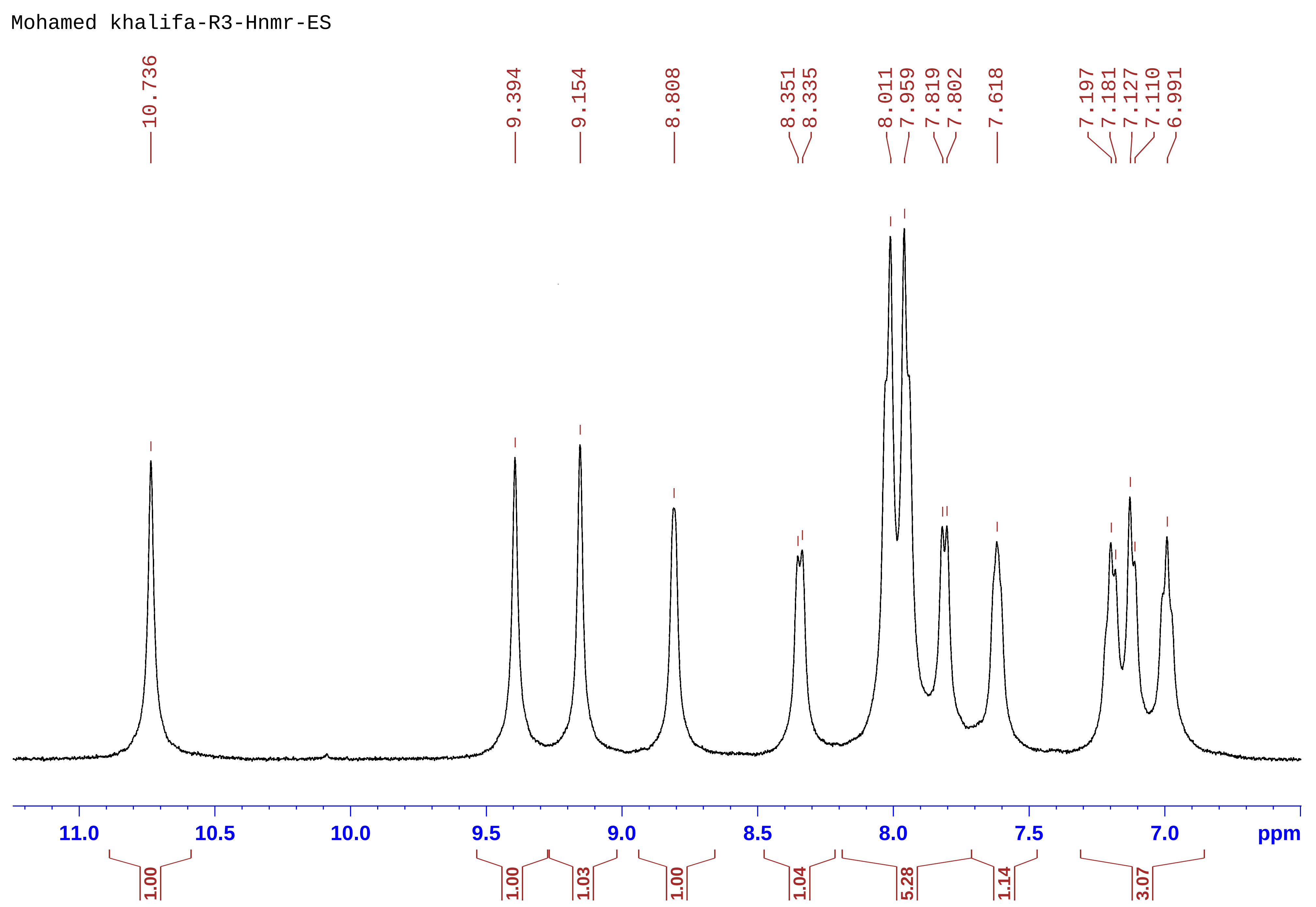

Mohamed khalifa-R3-Hnmr-ES
10.736
9.394
9.154
8.808
8.351
8.335
8.011
7.959
7.819
7.802
7.618
7.197
7.181
7.127
7.110
6.991
11.0
10.5
10.0
9.5
9.0
8.5
8.0
7.5
7.0
ppm
1.00
1.00
1.03
1.00
1.04
5.28
1.14
3.07

Mohamed khalifa-R4-Hnmr-ES

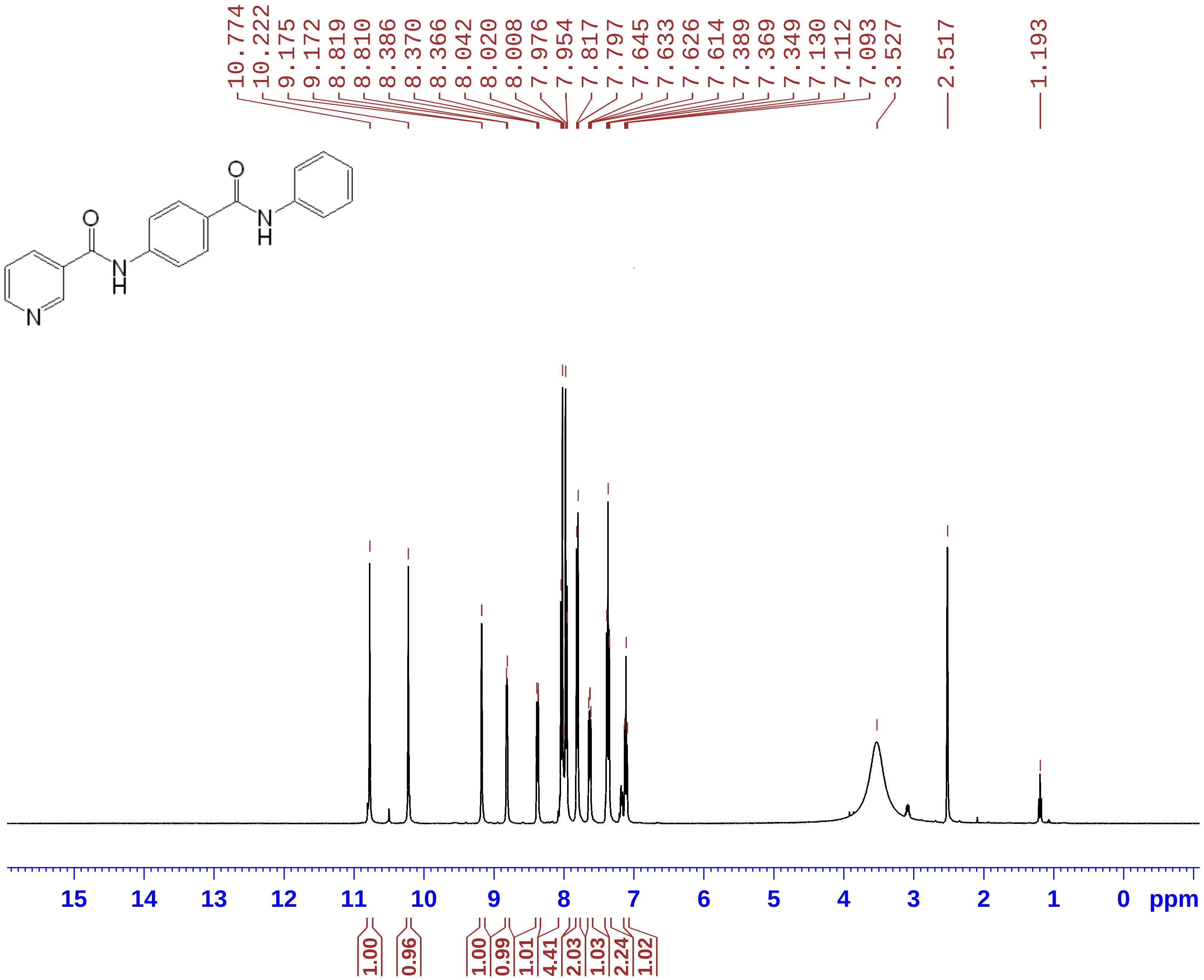


```
Current Data Parameters
NAME       Mohamed khalifa-R4-
EXPNO                     10
PROCNO                     1

F2 - Acquisition Parameters
Date_               20201126
Time                   13.02 h
INSTRUM                spect
PROBHD    Z108618_0945 (
PULPROG                 zg30
TD                     65536
SOLVENT                 DMSO
NS                        16
DS                         2
SWH                 8012.820 Hz
FIDRES              0.244532 Hz
AQ                 4.0894465 sec
RG                    112.56
DW                    62.400 usec
DE                      6.50 usec
TE                     293.1 K
D1                1.00000000 sec
TD0                        1
SFO1             400.2024712 MHz
NUC1                      1H
P1                     13.50 usec
PLW1             13.00000000 W

F2 - Processing parameters
SI                     65536
SF               400.2000000 MHz
WDW                       EM
SSB      0
LB                      0.30 Hz
GB       0
PC                      1.00
```

Mohamed khalifa-R4-Hnmr-ES

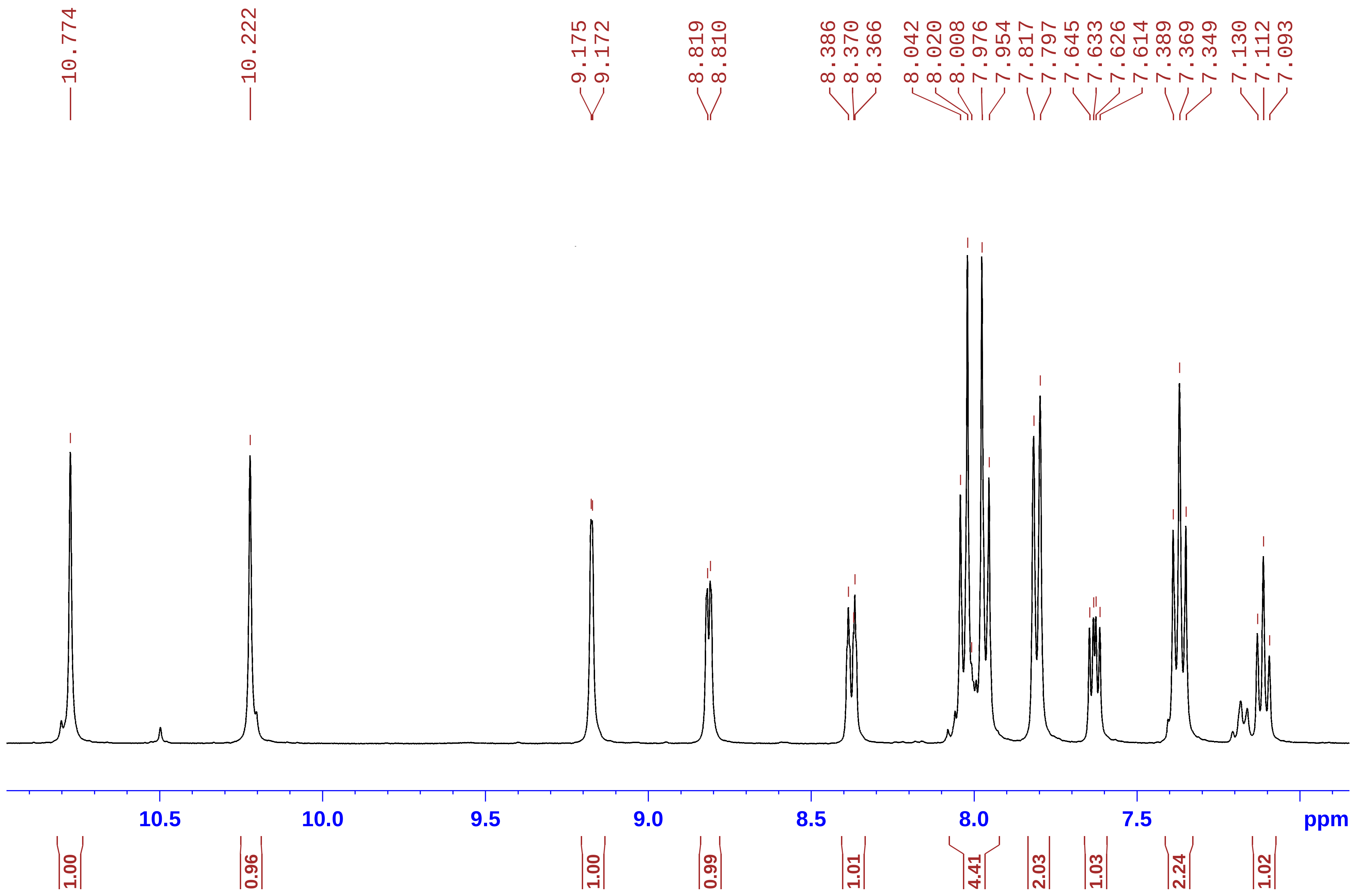

Mohamed khalifa-R5-Hnmr-ES

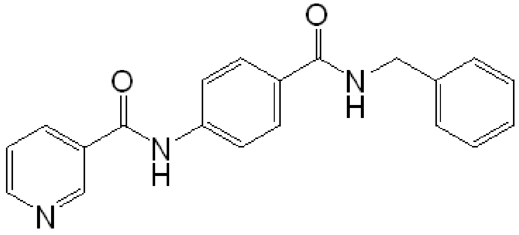

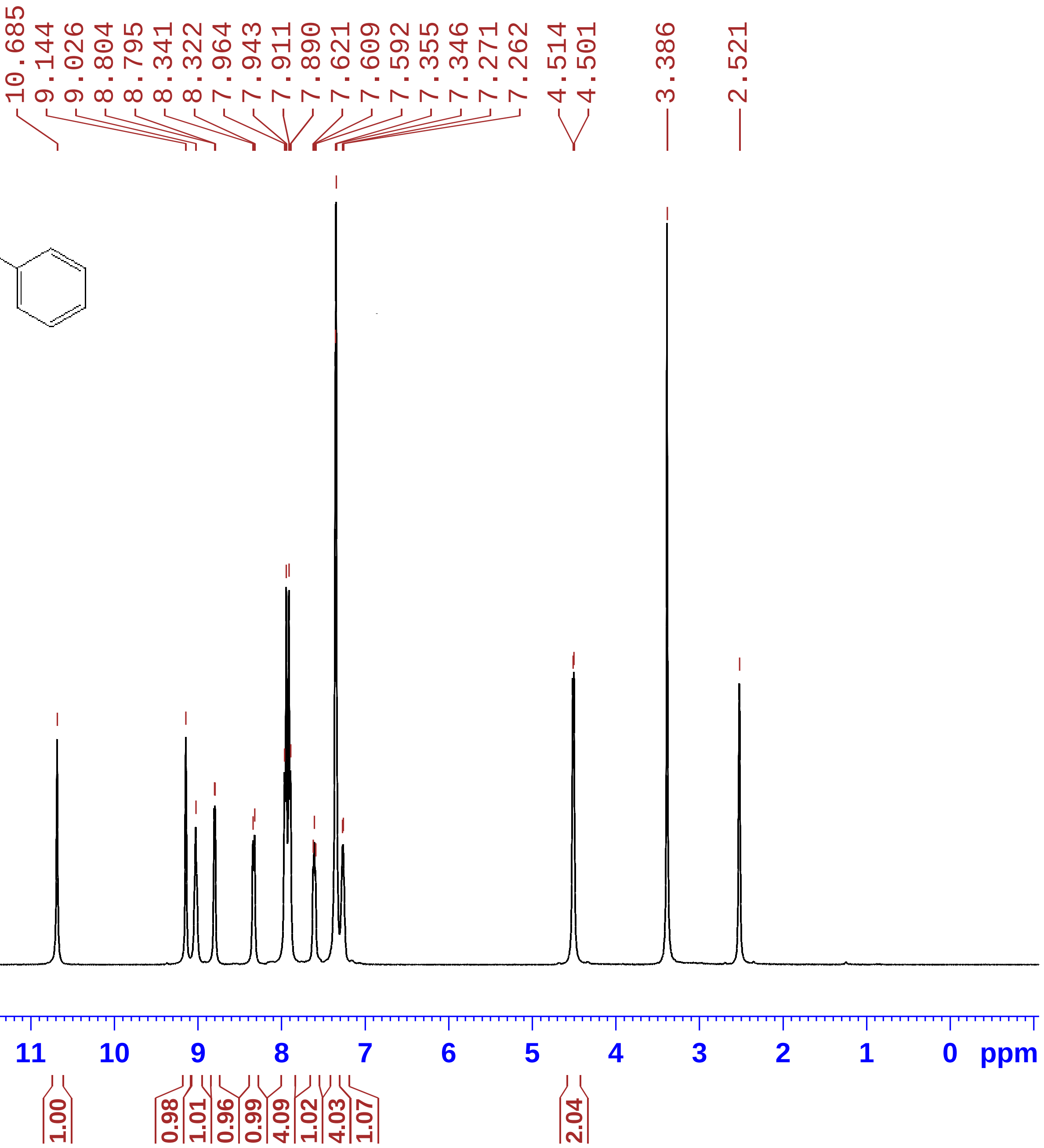

Current Data Parameters
NAME Mohamed khalifa-R5-
EXPNO 10
PROCNO 1

F2 - Acquisition Parameters
Date_ 20201126
Time 13.07 h
INSTRUM spect
PROBHD Z108618_0945 (
PULPROG zg30
TD 65536
SOLVENT DMSO
NS 16
DS 2
SWH 8012.820 Hz
FIDRES 0.244532 Hz
AQ 4.0894465 sec
RG 135.42
DW 62.400 usec
DE 6.50 usec
TE 293.2 K
D1 1.00000000 sec
TD0 1
SFO1 400.2024712 MHz
NUC1 1H
P1 13.50 usec
PLW1 13.00000000 W

F2 - Processing parameters
SI 65536
SF 400.2000000 MHz
WDW EM
SSB 0
LB 0.30 Hz
GB 0
PC 1.00

Mohamed khalifa-R5-Hnmr-ES

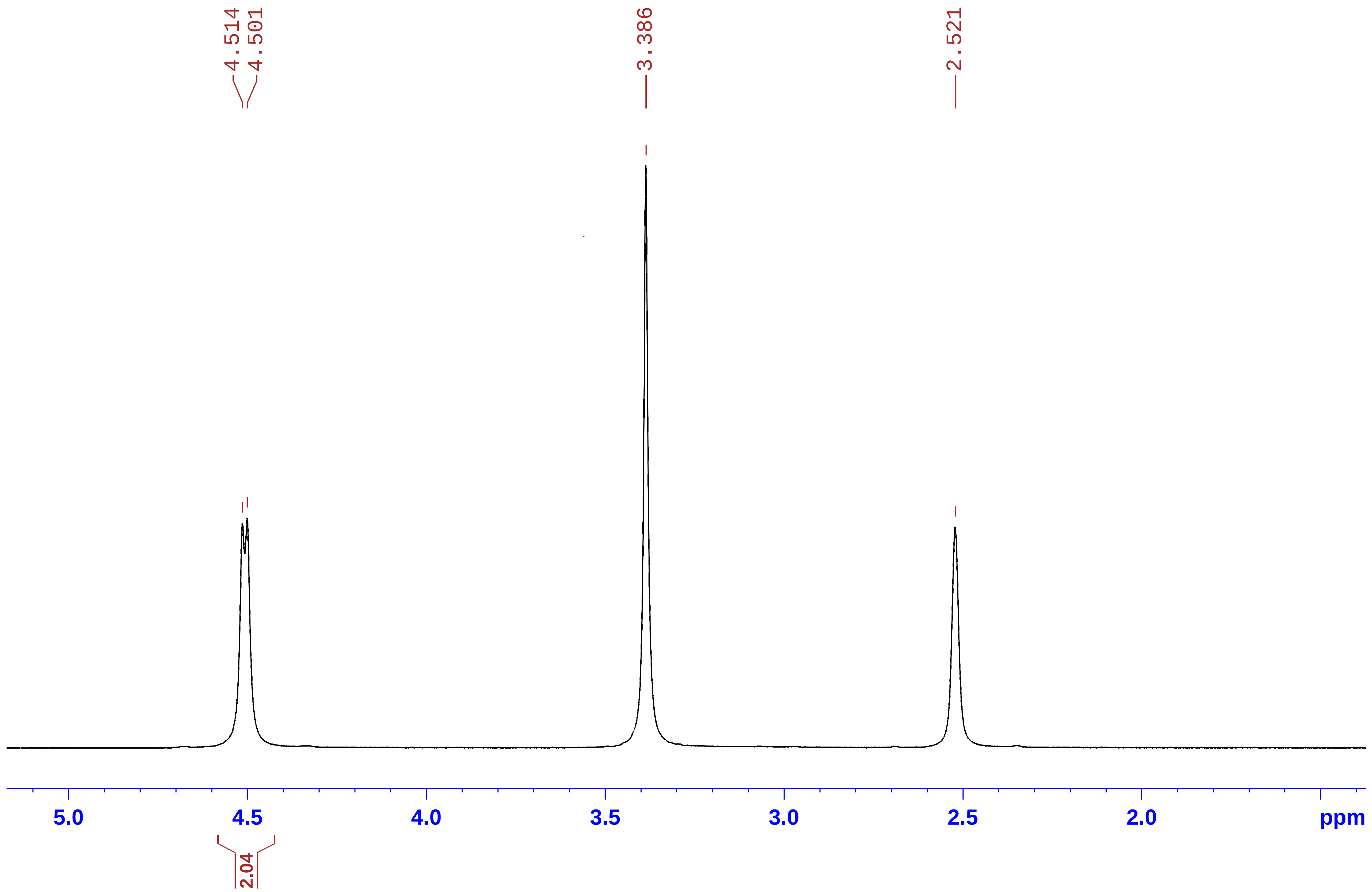

Mohamed khalifa-R5-Hnmr-ES

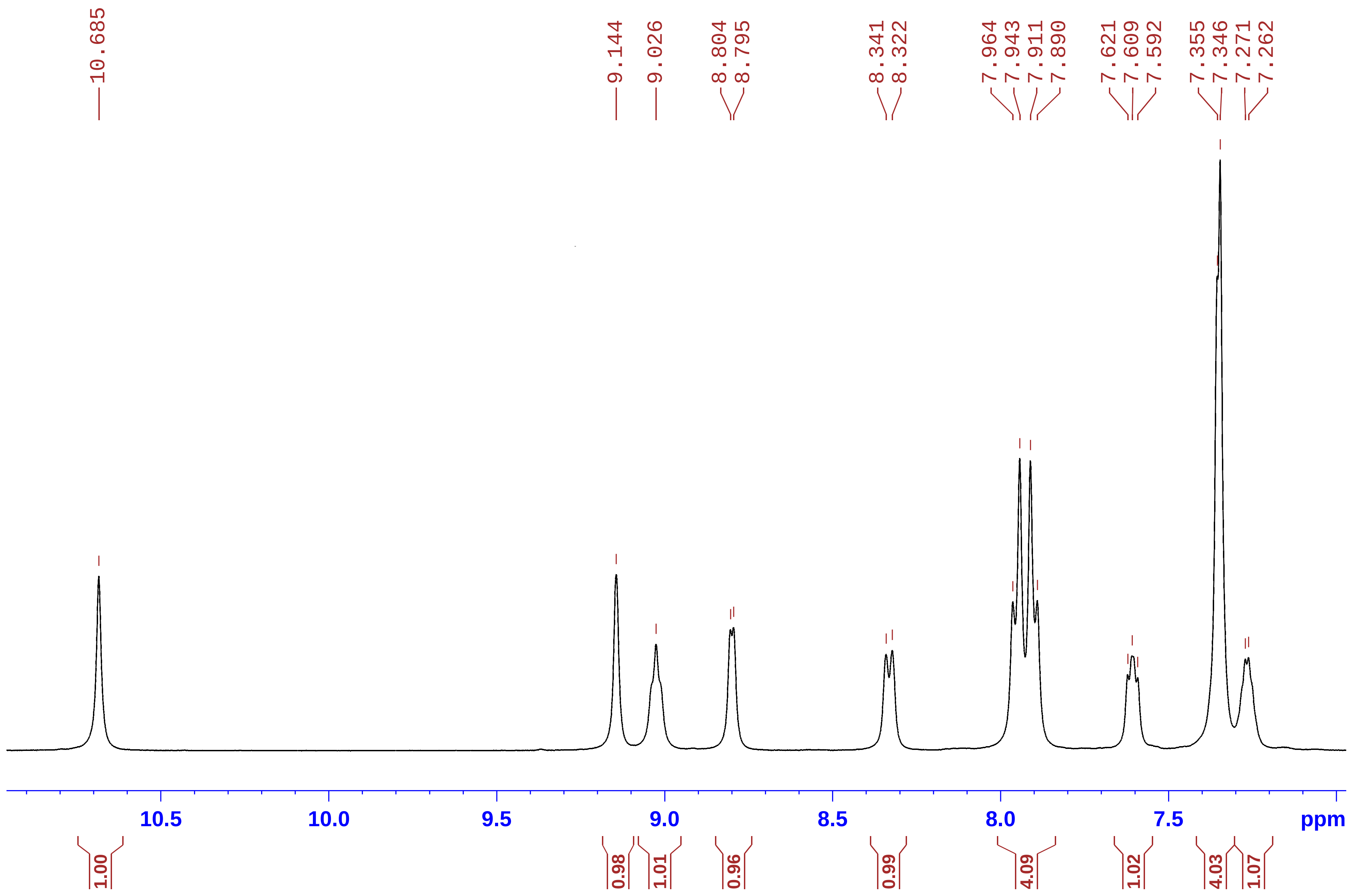

Mohamed khalifa-R7-Hnmr-ES

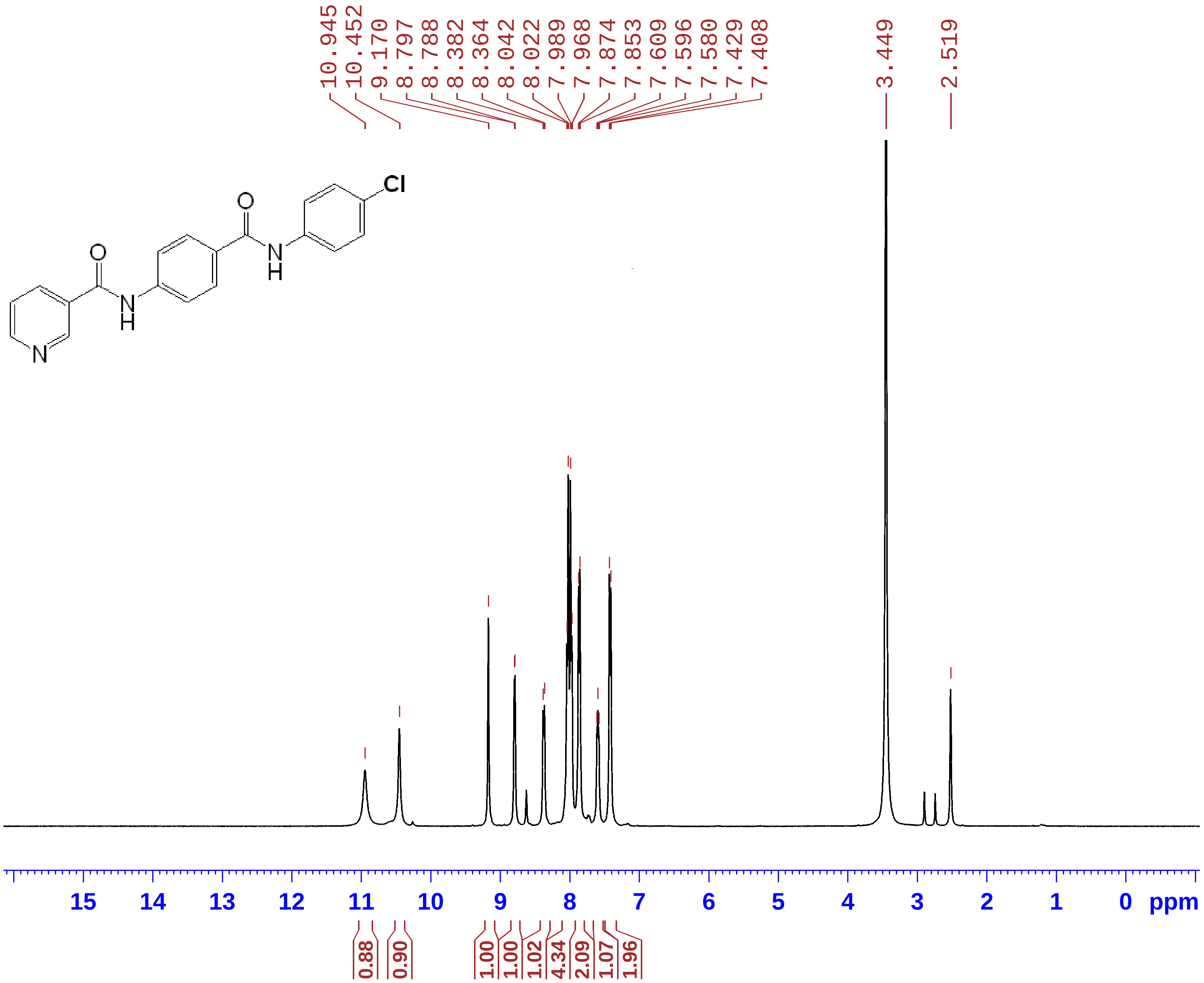

Current Data Parameters
NAME Mohamed khalifa-R7-
EXPNO 10
PROCNO 1

F2 - Acquisition Parameters
Date_ 20201126
Time 13.12 h
INSTRUM spect
PROBHD Z108618_0945 (
PULPROG zg30
TD 65536
SOLVENT DMSO
NS 16
DS 2
SWH 8012.820 Hz
FIDRES 0.244532 Hz
AQ 4.0894465 sec
RG 88.92
DW 62.400 usec
DE 6.50 usec
TE 293.1 K
D1 1.00000000 sec
TD0 1
SFO1 400.2024712 MHz
NUC1 1H
P1 13.50 usec
PLW1 13.00000000 W

F2 - Processing parameters
SI 65536
SF 400.2000000 MHz
WDW EM
SSB 0
LB 0.30 Hz
GB 0
PC 1.00

Mohamed khalifa-R7-Hnmr-ES

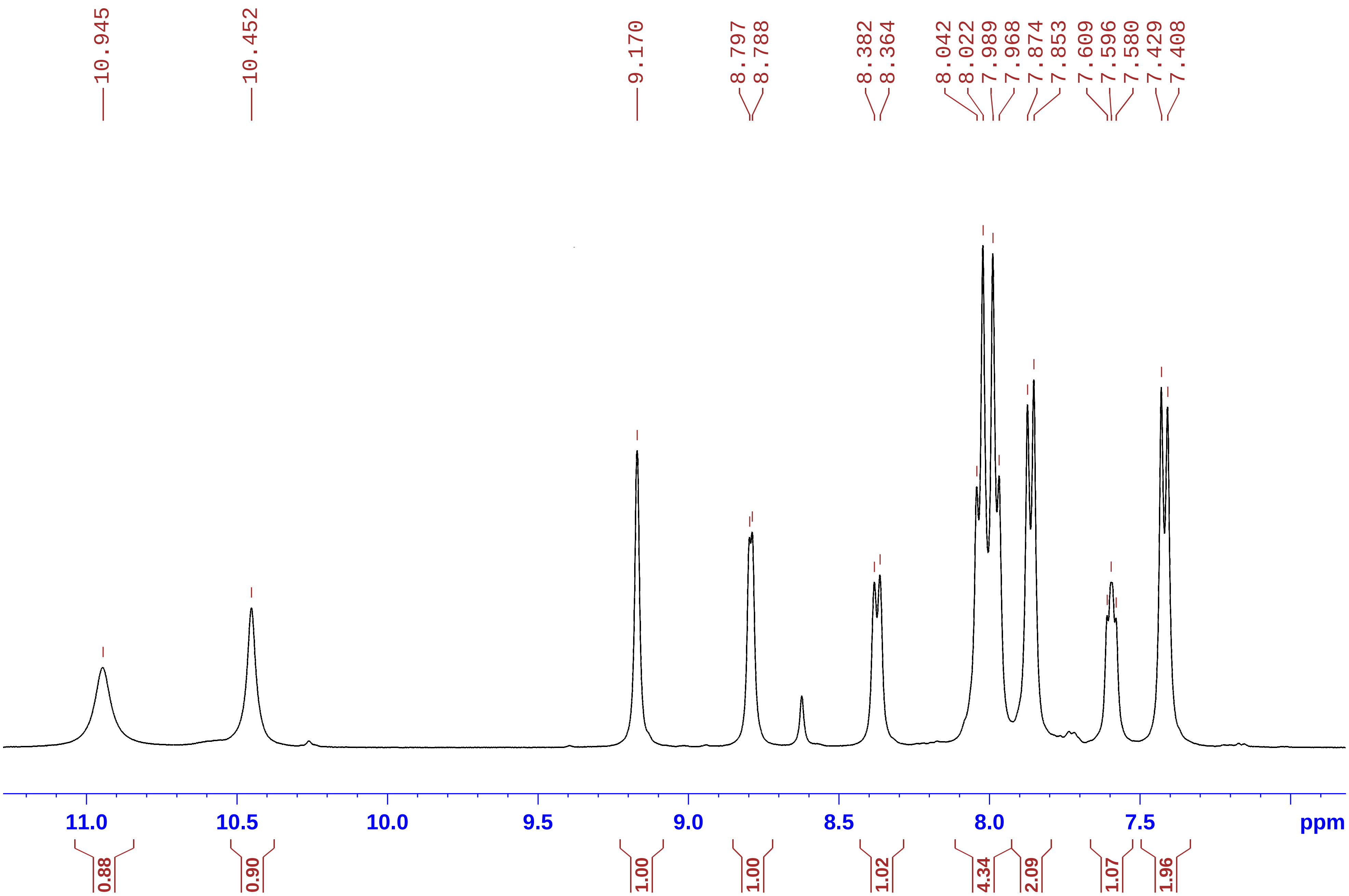

Mohamed khalifa-R8-Hnmr-ES

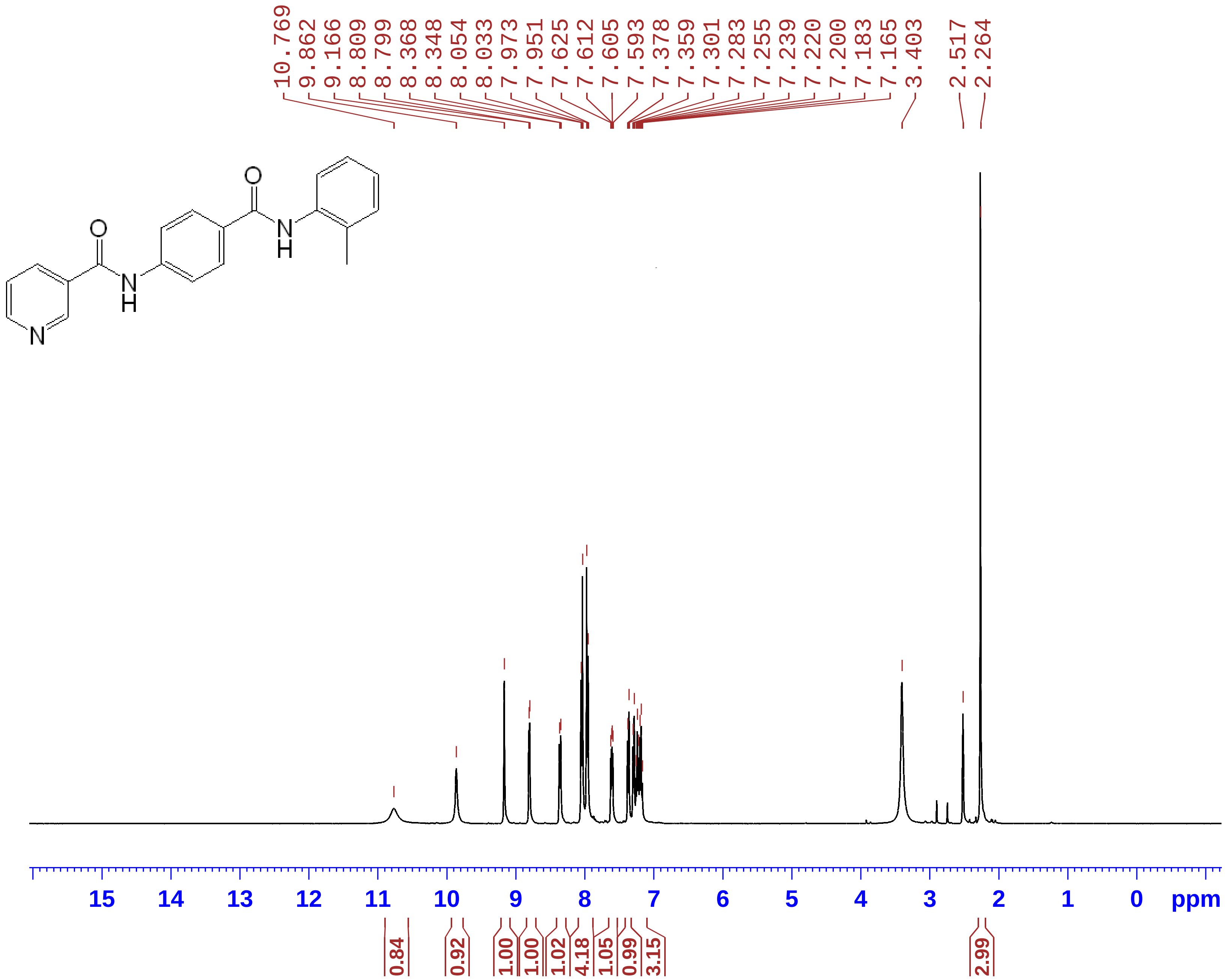

Current Data Parameters
NAME Mohamed khalifa-R8-
EXPNO 10
PROCNO 1

F2 - Acquisition Parameters
Date_ 20201126
Time 13.17 h
INSTRUM spect
PROBHD Z108618_0945 (
PULPROG zg30
TD 65536
SOLVENT DMSO
NS 16
DS 2
SWH 8012.820 Hz
FIDRES 0.244532 Hz
AQ 4.0894465 sec
RG 99.3
DW 62.400 usec
DE 6.50 usec
TE 293.1 K
D1 1.00000000 sec
TD0 1
SFO1 400.2024712 MHz
NUC1 1H
P1 13.50 usec
PLW1 13.00000000 W

F2 - Processing parameters
SI 65536
SF 400.2000000 MHz
WDW EM
SSB 0
LB 0.30 Hz
GB 0
PC 1.00

Mohamed khalifa-R8-Hnmr-ES

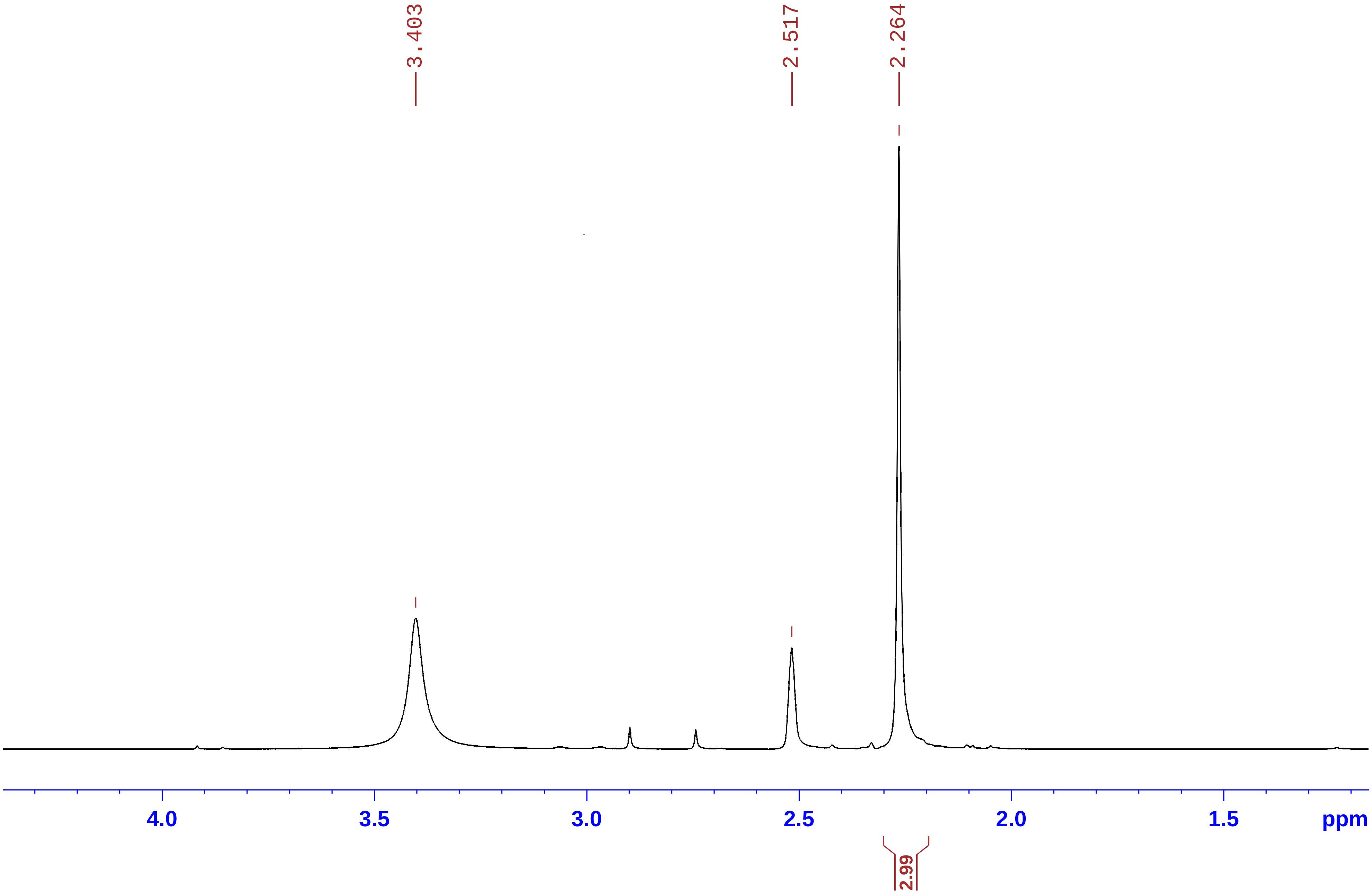

Mohamed khalifa-R8-Hnmr-ES

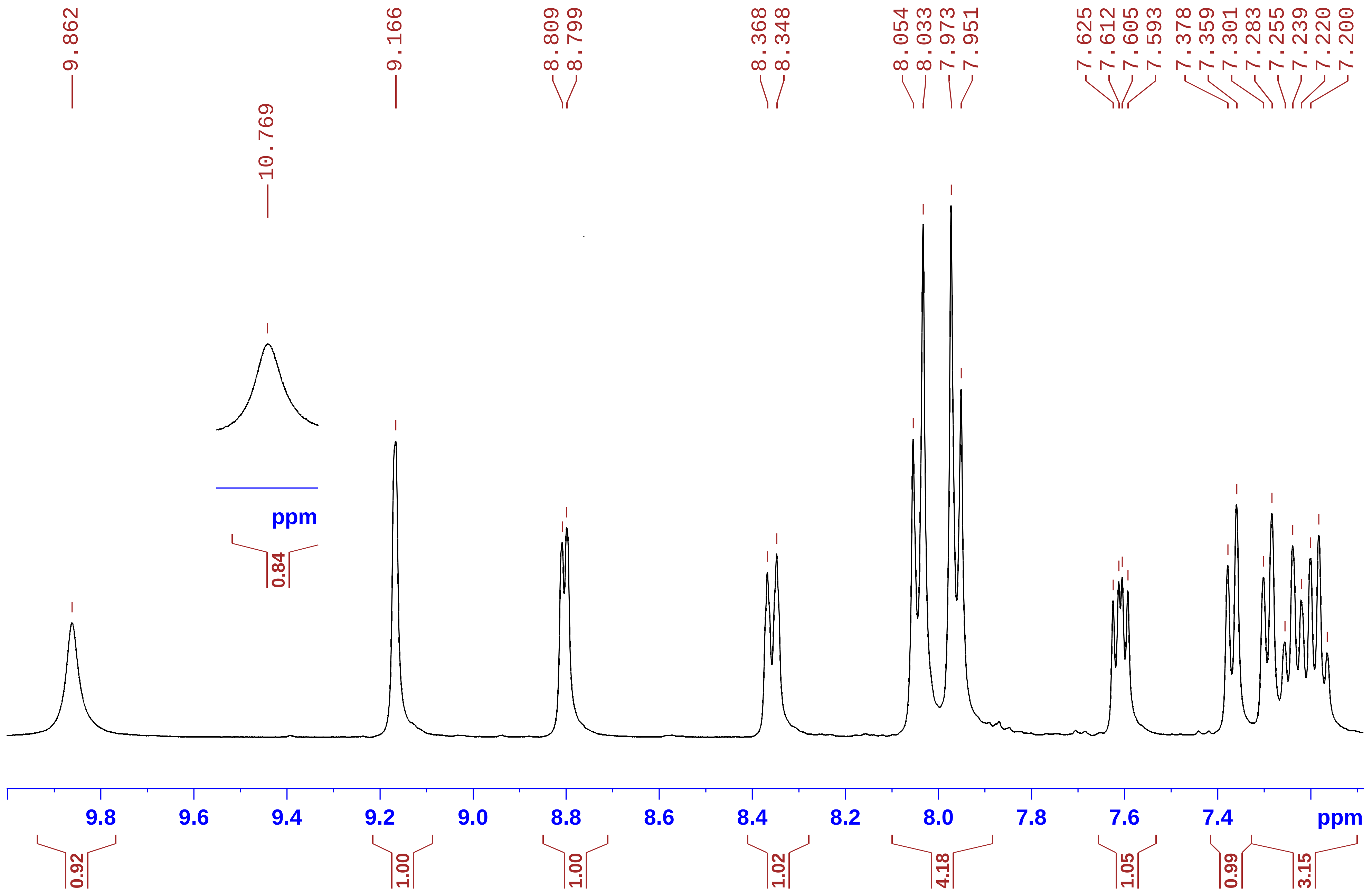

Mohamed khalifa-R10-Hnmr-ES

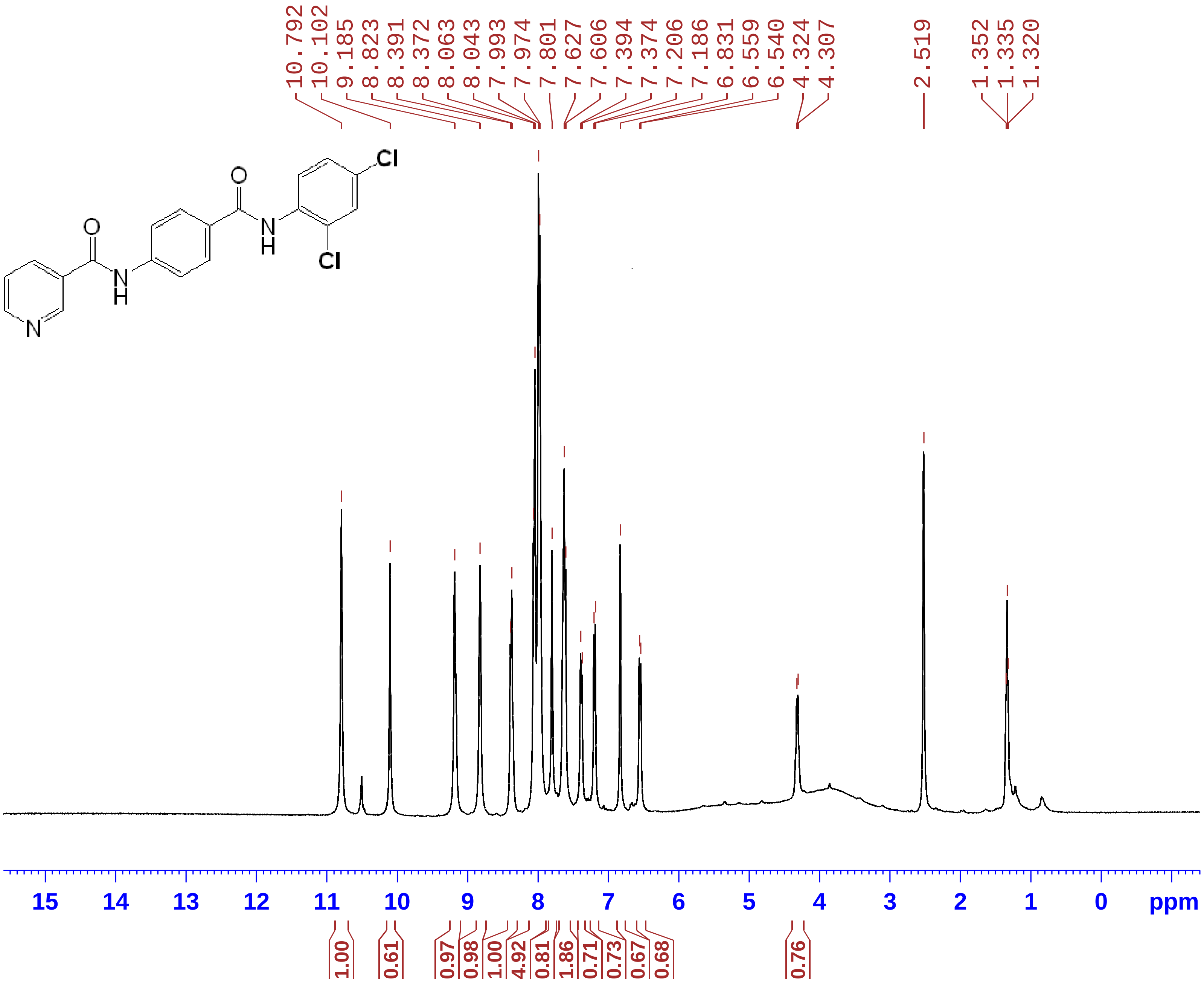

Current Data Parameters
NAME Mohamed khalifa-R10
EXPNO 10
PROCNO 1

F2 - Acquisition Parameters
Date_ 20201126
Time 13.21 h
INSTRUM spect
PROBHD Z108618_0945 (
PULPROG zg30
TD 65536
SOLVENT DMSO
NS 16
DS 2
SWH 8012.820 Hz
FIDRES 0.244532 Hz
AQ 4.0894465 sec
RG 99.3
DW 62.400 usec
DE 6.50 usec
TE 293.2 K
D1 1.00000000 sec
TD0 1
SFO1 400.2024712 MHz
NUC1 1H
P1 13.50 usec
PLW1 13.00000000 W

F2 - Processing parameters
SI 65536
SF 400.2000000 MHz
WDW EM
SSB 0
LB 0.30 Hz
GB 0
PC 1.00

Mohamed khalifa-R10-Hnmr-ES

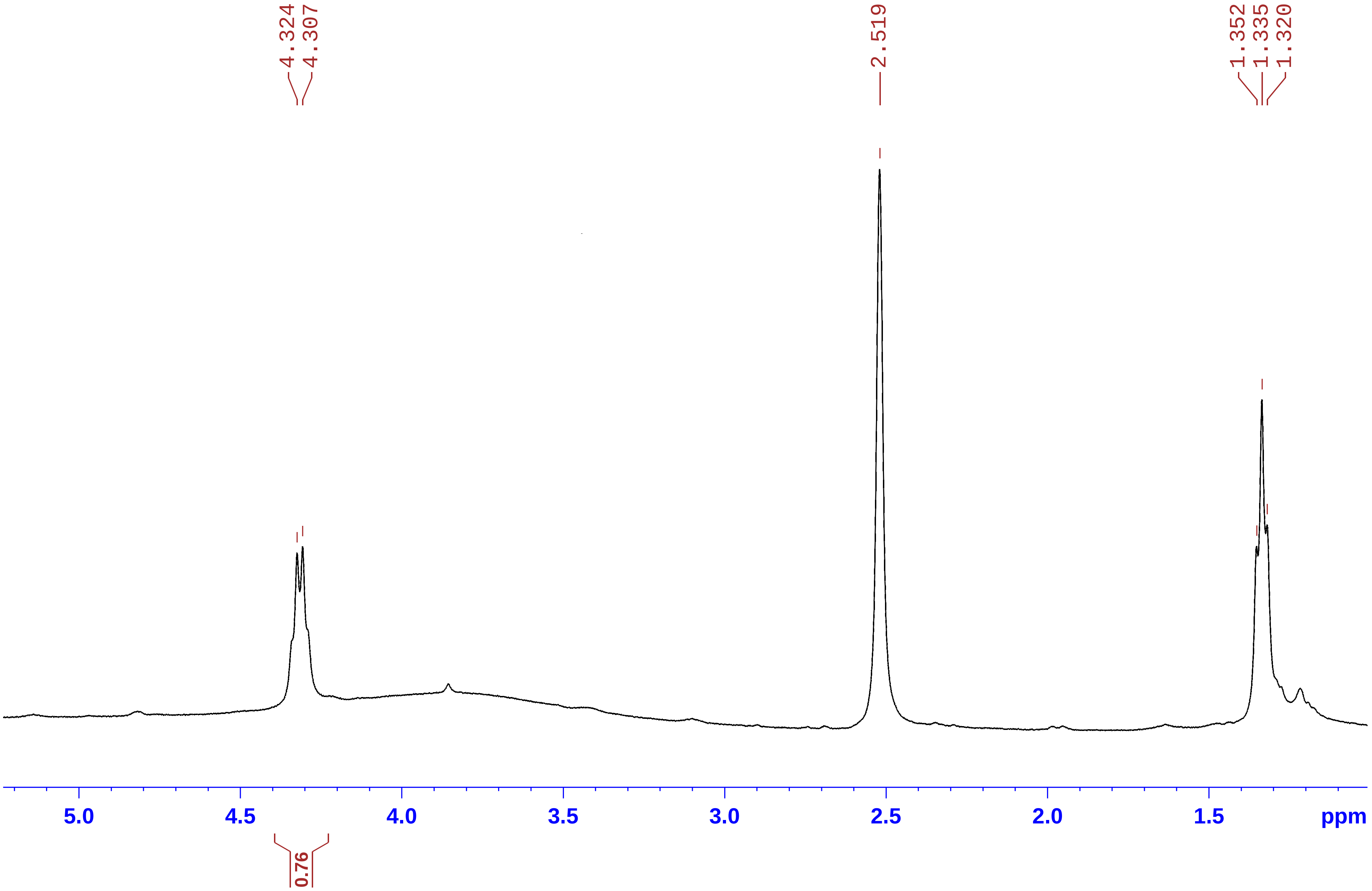

Mohamed khalifa-R10-Hnmr-ES

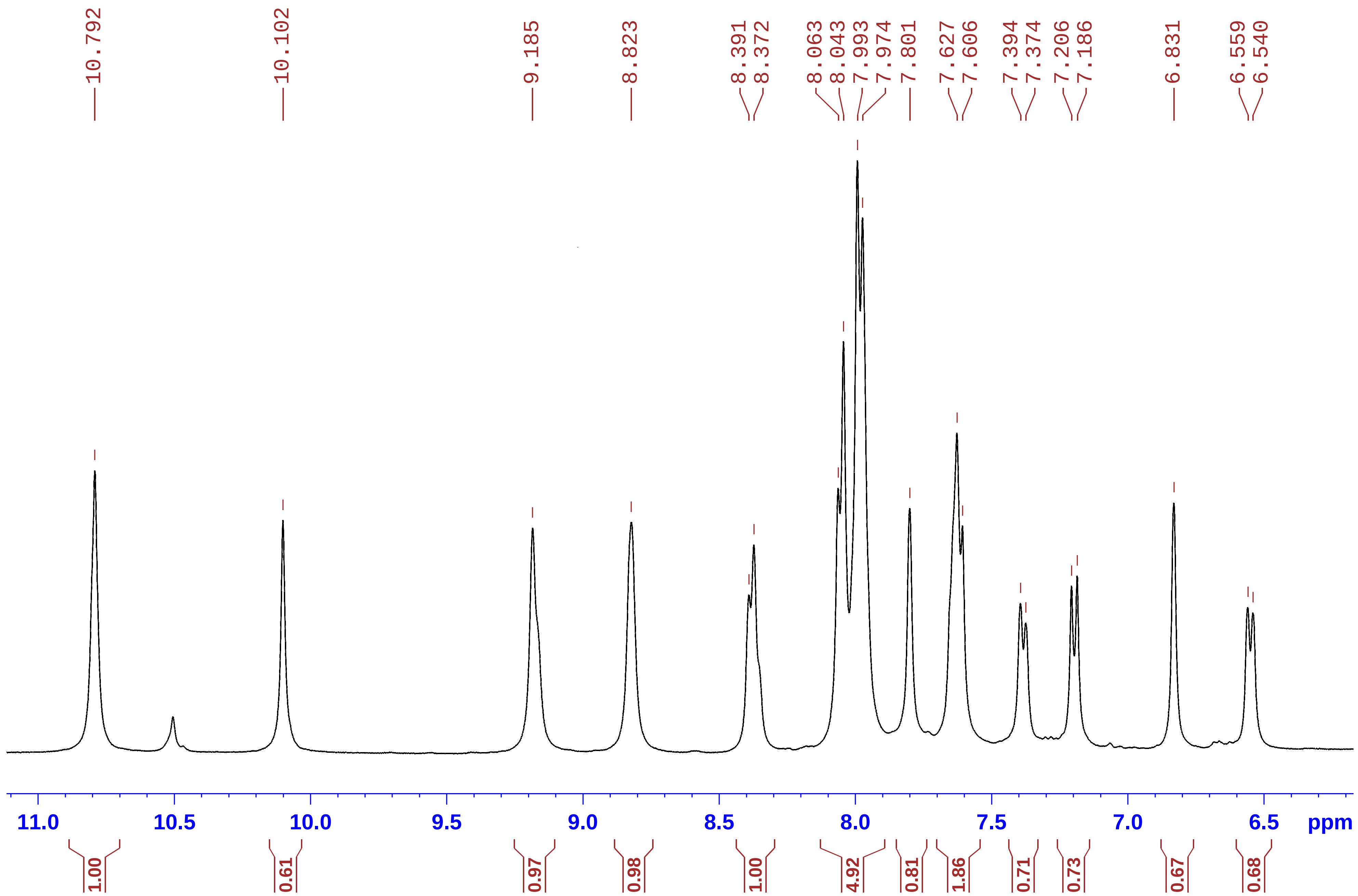

Mohamed khalifa-R11-Hnmr-ES

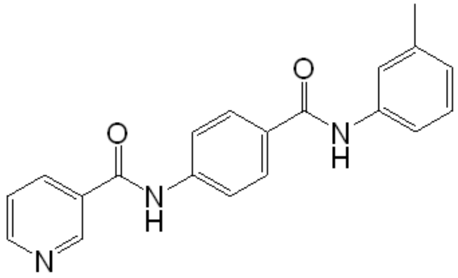

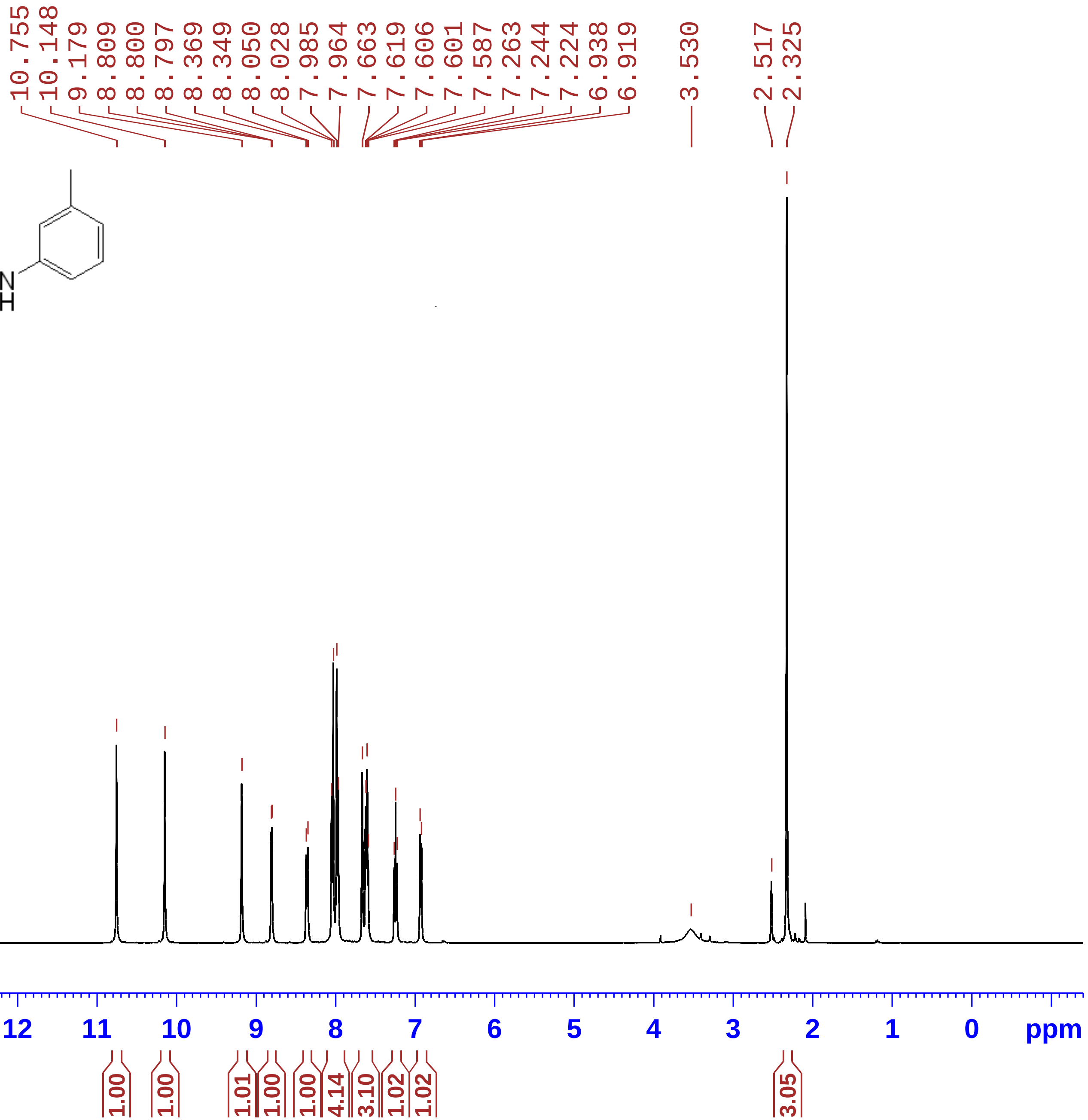

Current Data Parameters
NAME Mohamed khalifa-R11
EXPNO 10
PROCNO 1

F2 - Acquisition Parameters
Date_ 20201126
Time 13.26 h
INSTRUM spect
PROBHD Z108618_0945 (
PULPROG zg30
TD 65536
SOLVENT DMSO
NS 16
DS 2
SWH 8012.820 Hz
FIDRES 0.244532 Hz
AQ 4.0894465 sec
RG 54.17
DW 62.400 usec
DE 6.50 usec
TE 293.1 K
D1 1.00000000 sec
TD0 1
SFO1 400.2024712 MHz
NUC1 1H
P1 13.50 usec
PLW1 13.00000000 W

F2 - Processing parameters
SI 65536
SF 400.2000000 MHz
WDW EM
SSB 0
LB 0.30 Hz
GB 0
PC 1.00

Mohamed khalifa-R11-Hnmr-ES

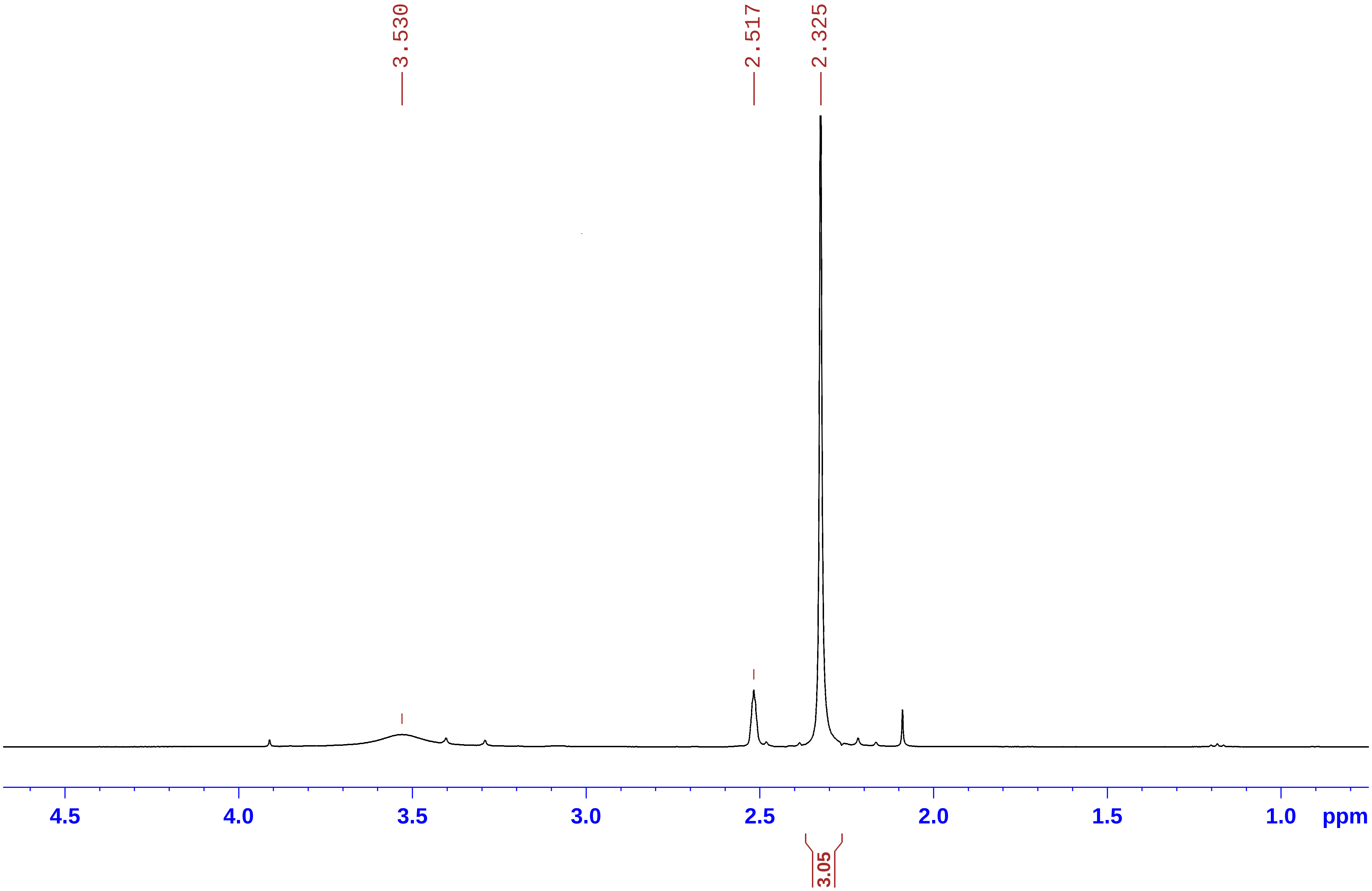

Mohamed khalifa-R11-Hnmr-ES

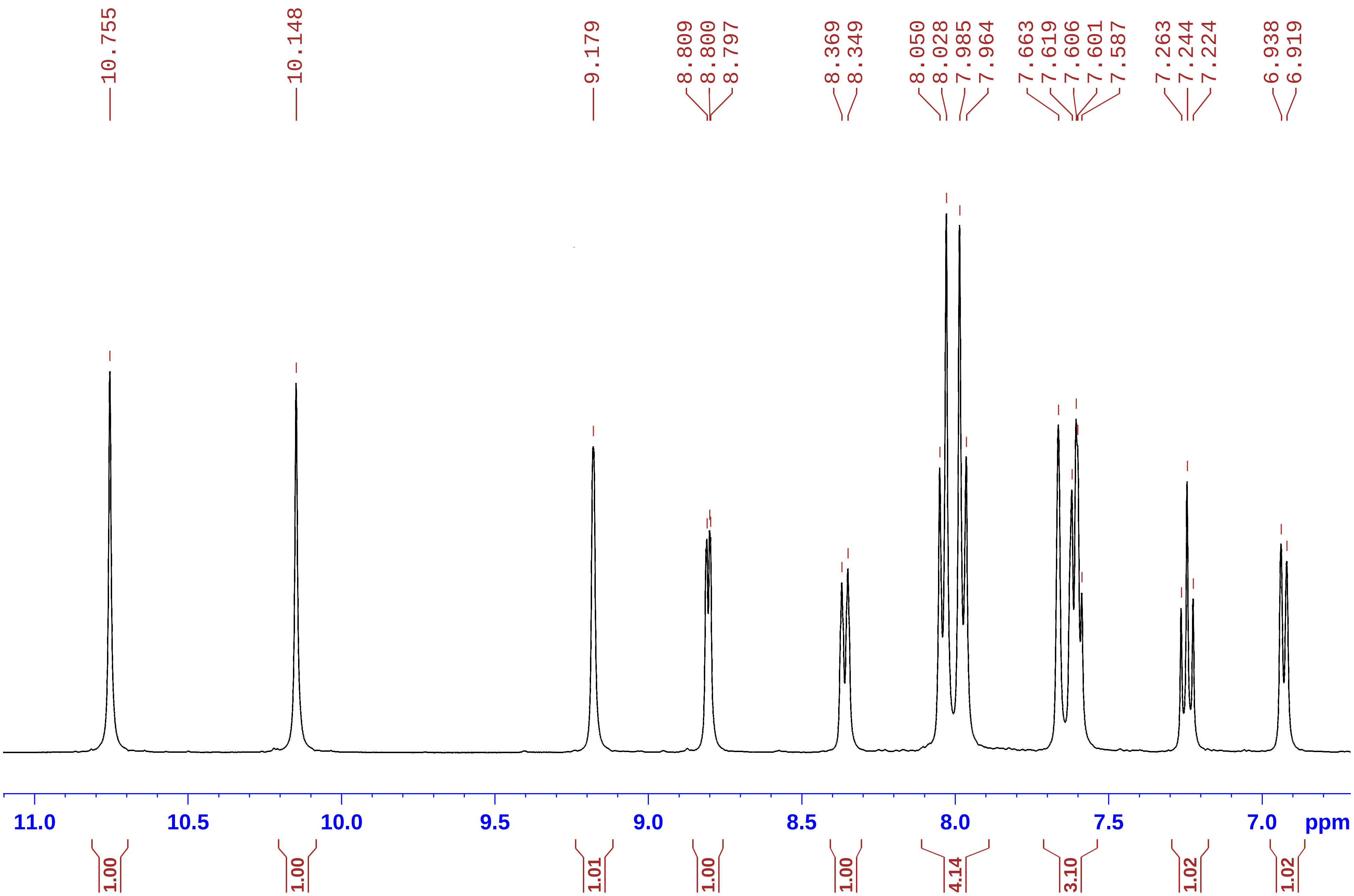

Mohamed khalifa-R12-Hnmr-ES

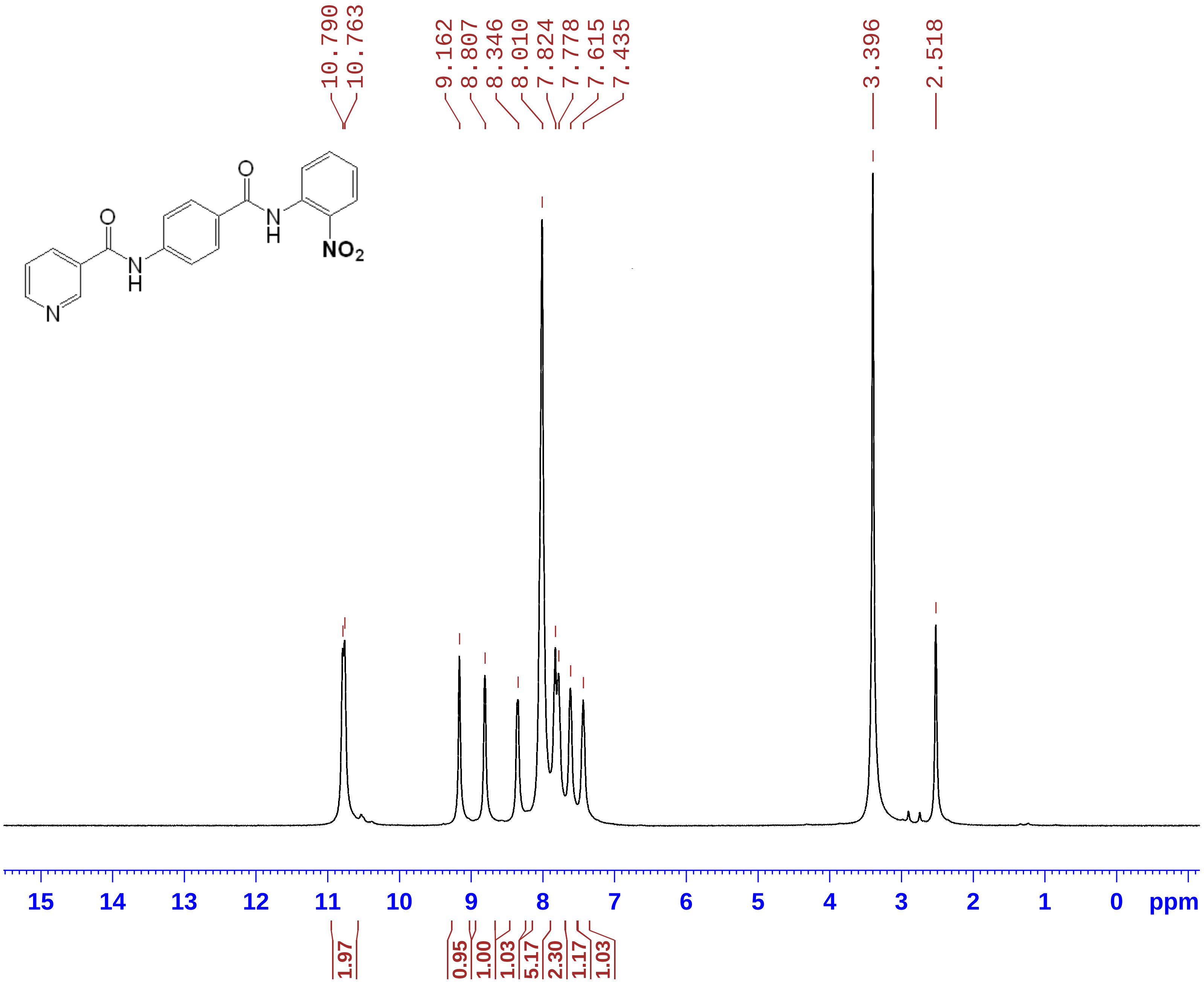

Faculty of Pharmacy

Current Data Parameters	
NAME	Mohamed khalifa-R12
EXPNO	10
PROCNO	1

F2 - Acquisition Parameters	
Date_	20201126
Time	13.31 h
INSTRUM	spect
PROBHD	Z108618_0945 (
PULPROG	zg30
TD	65536
SOLVENT	DMSO
NS	16
DS	2
SWH	8012.820 Hz
FIDRES	0.244532 Hz
AQ	4.0894465 sec
RG	120.93
DW	62.400 usec
DE	6.50 usec
TE	293.1 K
D1	1.00000000 sec
TD0	1
SFO1	400.2024712 MHz
NUC1	1H
P1	13.50 usec
PLW1	13.00000000 W

F2 - Processing parameters	
SI	65536
SF	400.2000000 MHz
WDW	EM
SSB	0
LB	0.30 Hz
GB	0
PC	1.00

Mohamed khalifa-R12-Hnmr-ES

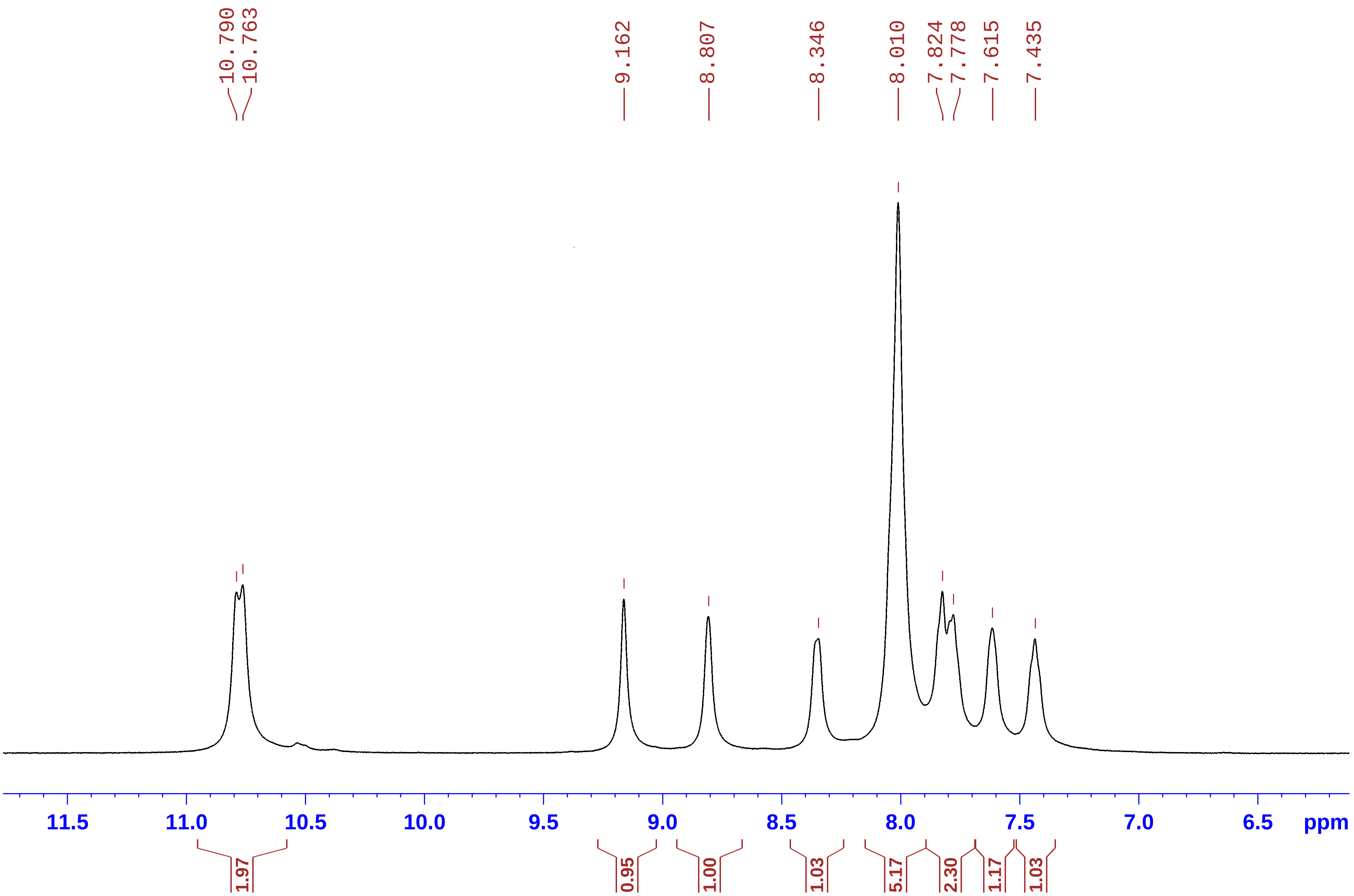

Mohamed khalifa-R14-Hnmr-ES

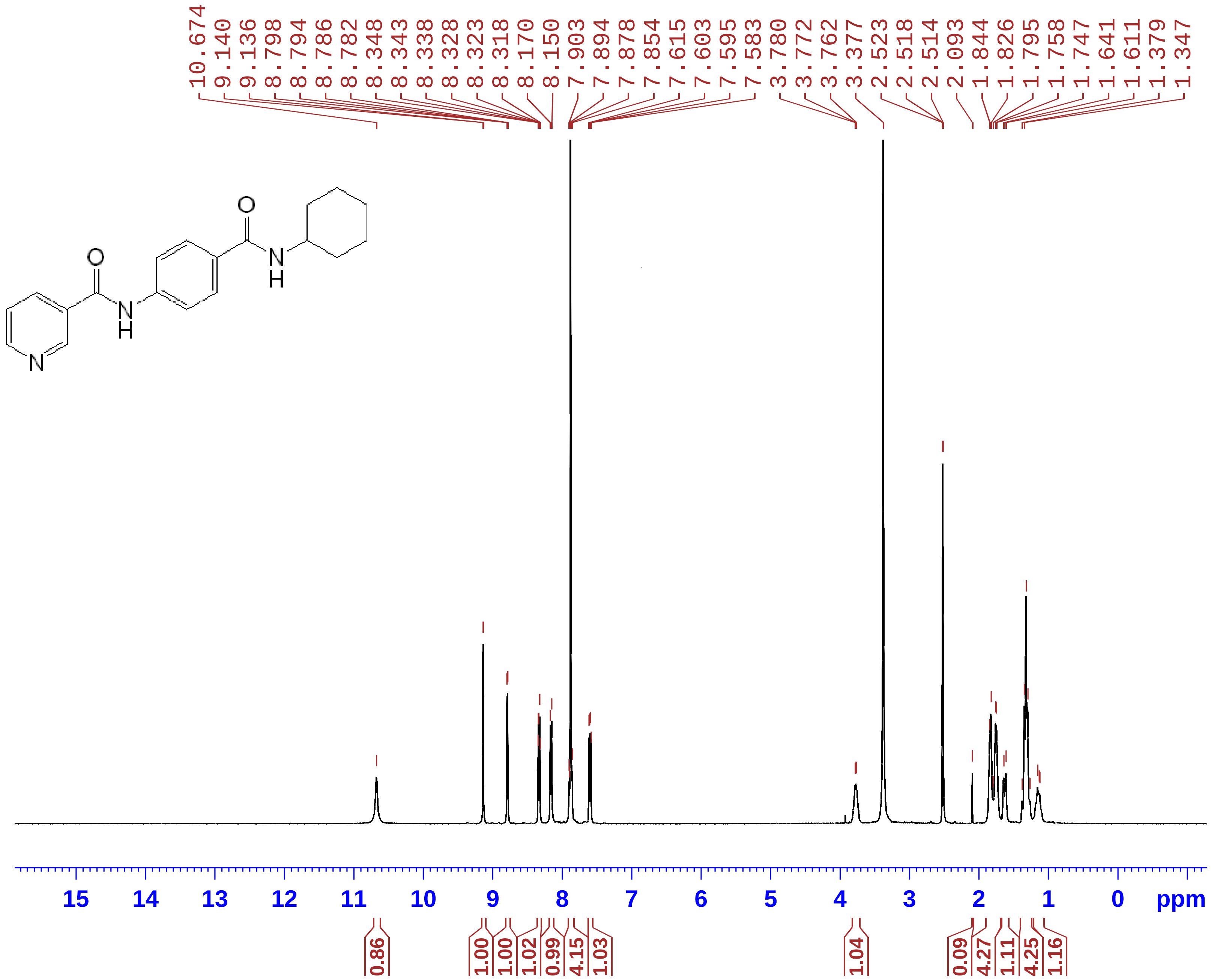


```
Current Data Parameters
NAME     Mohamed khalifa-R14
EXPNO                     10
PROCNO                     1

F2 - Acquisition Parameters
Date_              20201126
Time                  13.36 h
INSTRUM               spect
PROBHD    Z108618_0945 (
PULPROG                zg30
TD                    65536
SOLVENT                DMSO
NS                       16
DS                        2
SWH                8012.820 Hz
FIDRES             0.244532 Hz
AQ                4.0894465 sec
RG                   135.42
DW                   62.400 usec
DE                     6.50 usec
TE                    293.2 K
D1               1.00000000 sec
TD0                       1
SFO1            400.2024712 MHz
NUC1                     1H
P1                    13.50 usec
PLW1            13.00000000 W

F2 - Processing parameters
SI                    65536
SF              400.2000000 MHz
WDW                      EM
SSB      0
LB                     0.30 Hz
GB       0
PC                     1.00
```

Faculty of Pharmacy

Mohamed khalifa-R14-Hnmr-ES

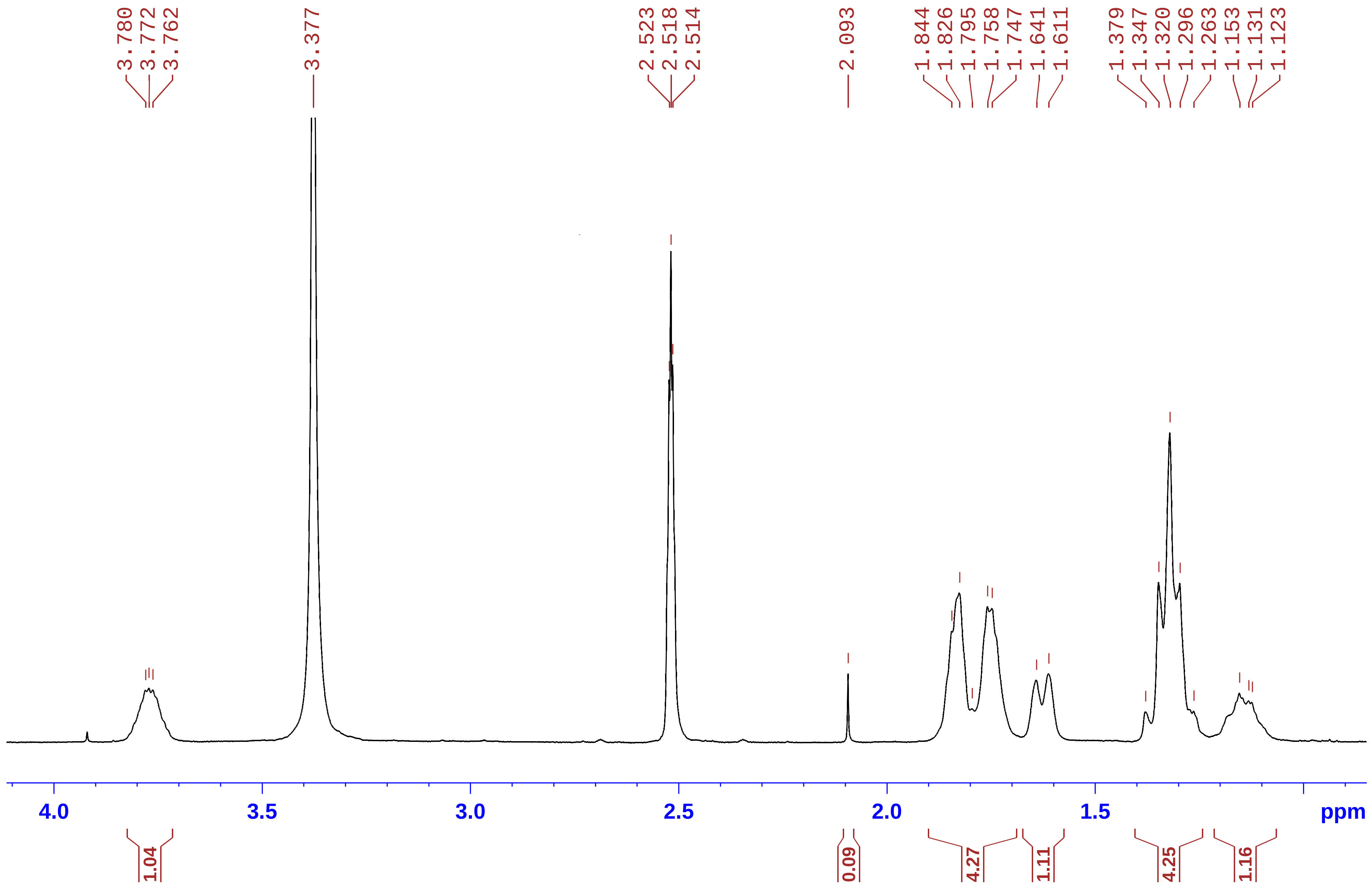

Mohamed khalifa-R14-Hnmr-ES

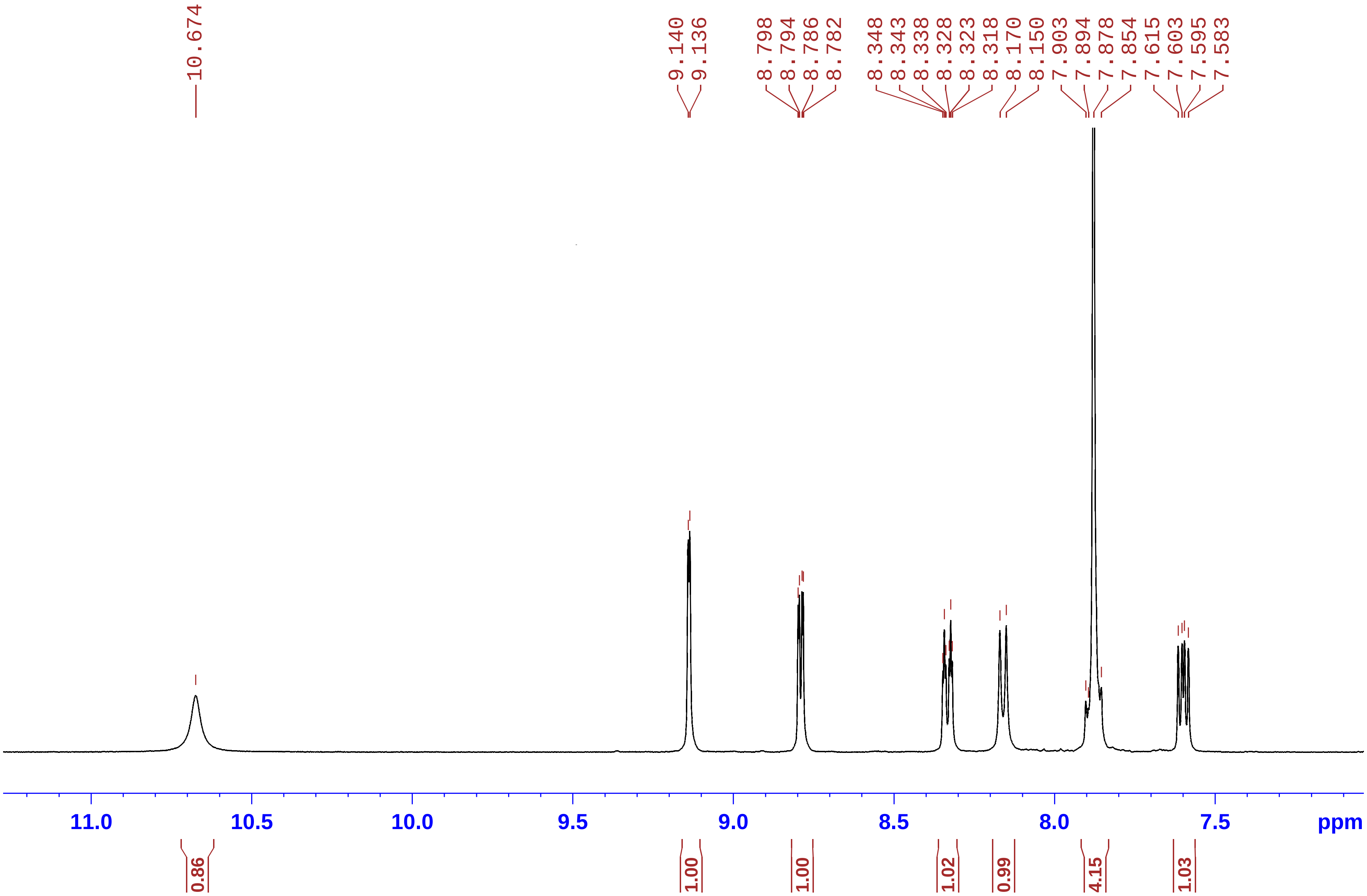

Mohamed khalifa-R20-Hnmr-ES

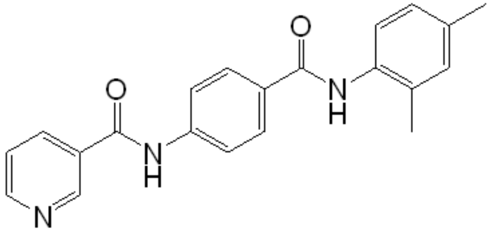

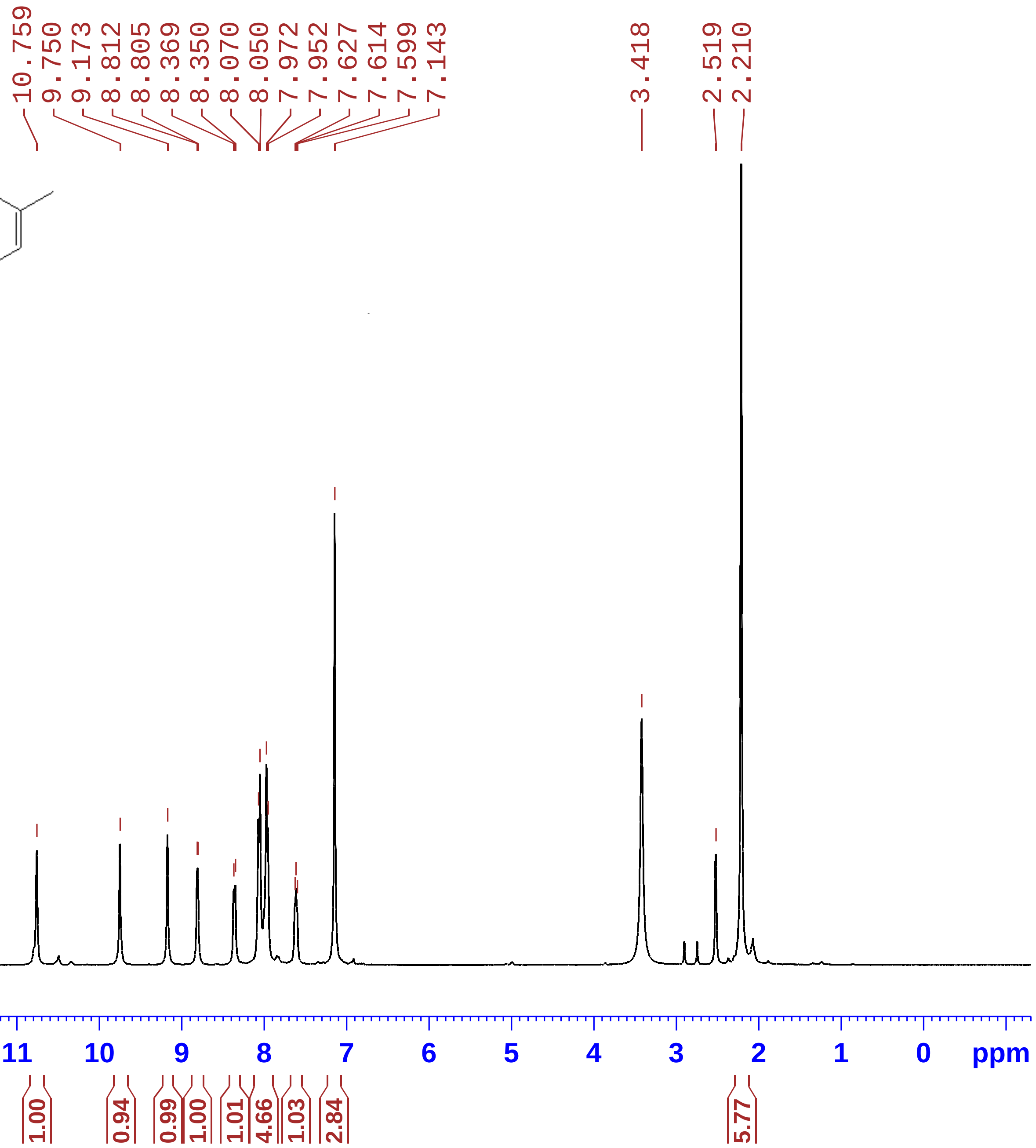

Faculty of Pharmacy

Current Data Parameters
NAME Mohamed khalifa-R20
EXPNO 10
PROCNO 1

F2 - Acquisition Parameters
Date_ 20201126
Time 13.41 h
INSTRUM spect
PROBHD Z108618_0945 (
PULPROG zg30
TD 65536
SOLVENT DMSO
NS 16
DS 2
SWH 8012.820 Hz
FIDRES 0.244532 Hz
AQ 4.0894465 sec
RG 78.59
DW 62.400 usec
DE 6.50 usec
TE 293.1 K
D1 1.00000000 sec
TD0 1
SFO1 400.2024712 MHz
NUC1 1H
P1 13.50 usec
PLW1 13.00000000 W

F2 - Processing parameters
SI 65536
SF 400.2000000 MHz
WDW EM
SSB 0
LB 0.30 Hz
GB 0
PC 1.00

Mohamed khalifa-R20-Hnmr-ES

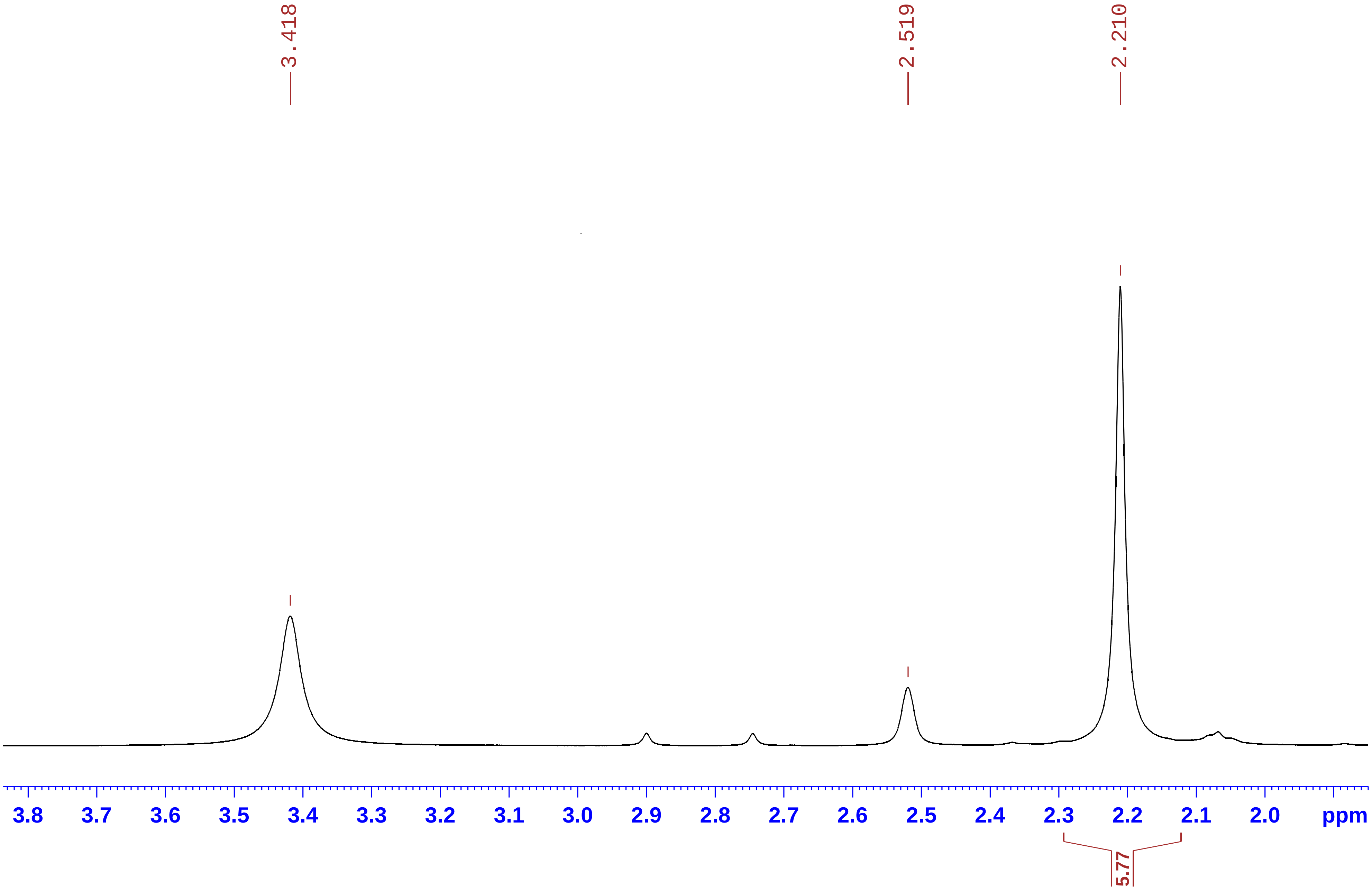

Mohamed khalifa-R20-Hnmr-ES

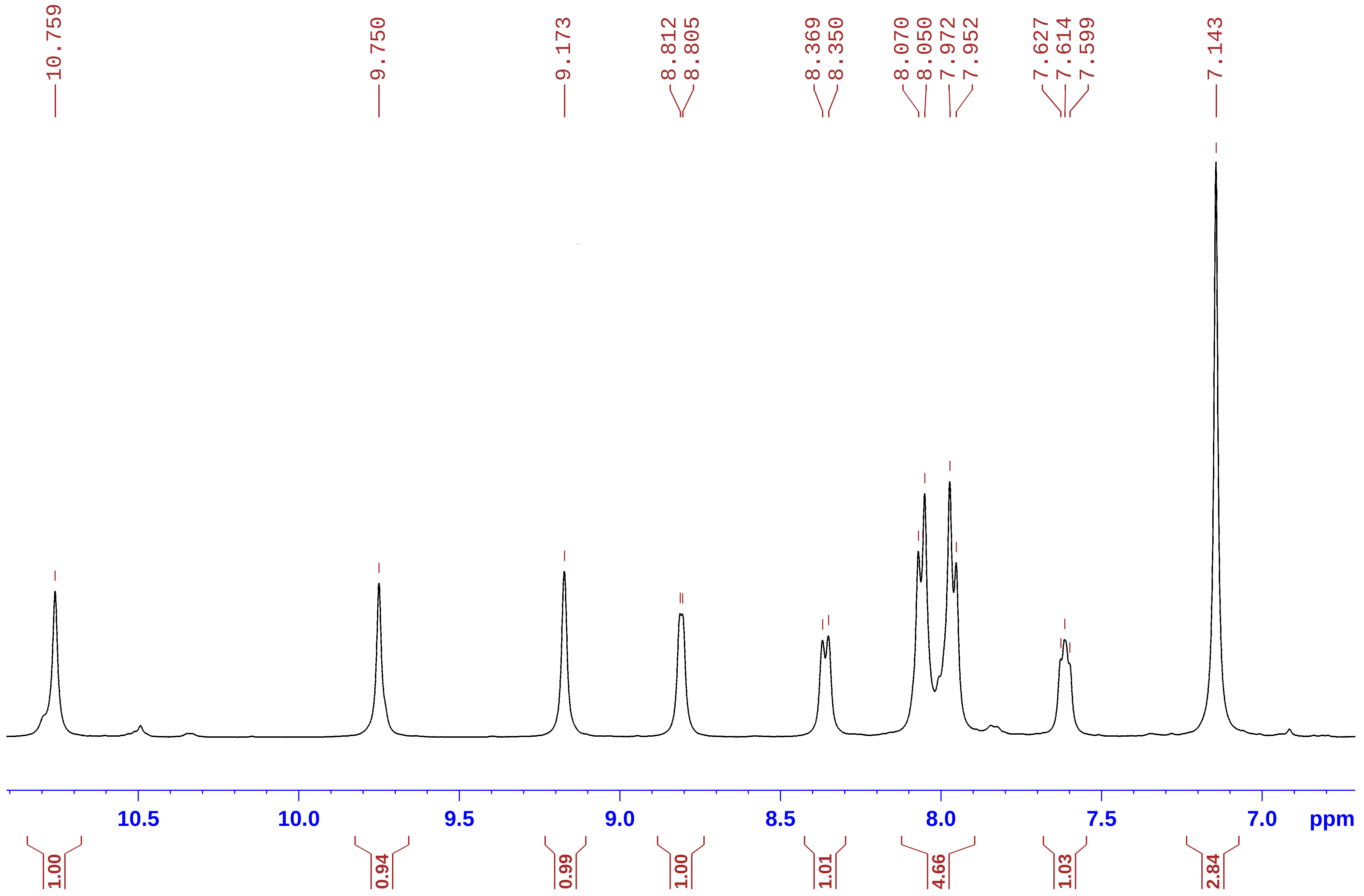

Mohamed khalifa-R21-Hnmr-ES

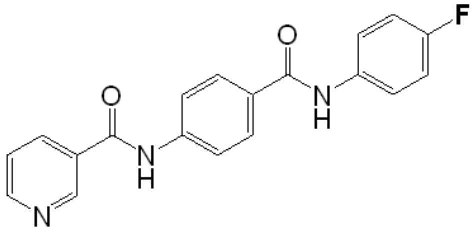

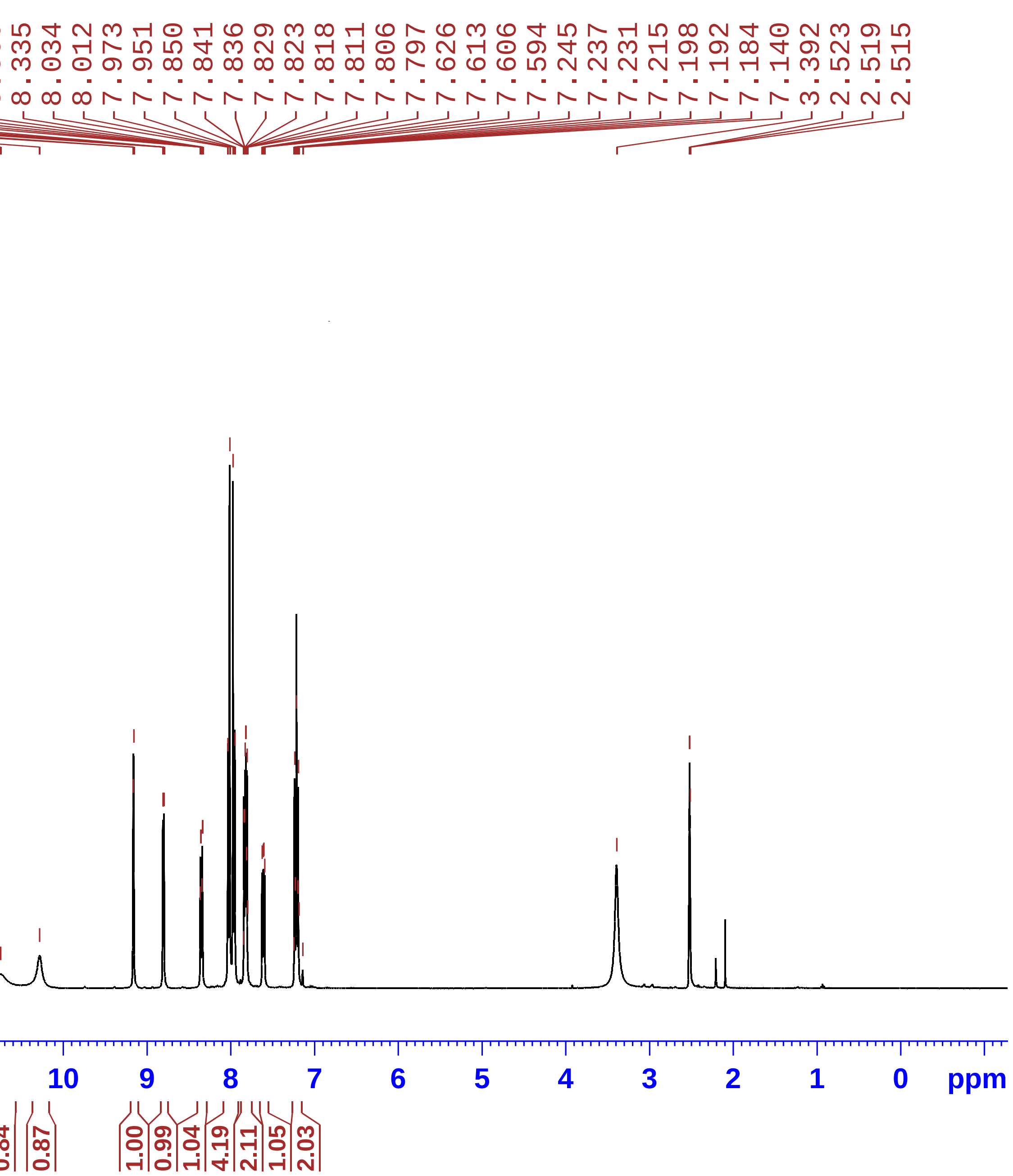

Current Data Parameters
NAME Mohamed khalifa-R21
EXPNO 10
PROCNO 1

F2 - Acquisition Parameters
Date_ 20201126
Time 13.46 h
INSTRUM spect
PROBHD Z108618_0945 (
PULPROG zg30
TD 65536
SOLVENT DMSO
NS 16
DS 2
SWH 8012.820 Hz
FIDRES 0.244532 Hz
AQ 4.0894465 sec
RG 120.93
DW 62.400 usec
DE 6.50 usec
TE 293.2 K
D1 1.00000000 sec
TD0 1
SFO1 400.2024712 MHz
NUC1 1H
P1 13.50 usec
PLW1 13.00000000 W

F2 - Processing parameters
SI 65536
SF 400.2000000 MHz
WDW EM
SSB 0
LB 0.30 Hz
GB 0
PC 1.00

Mohamed khalifa-R21-Hnmr-ES

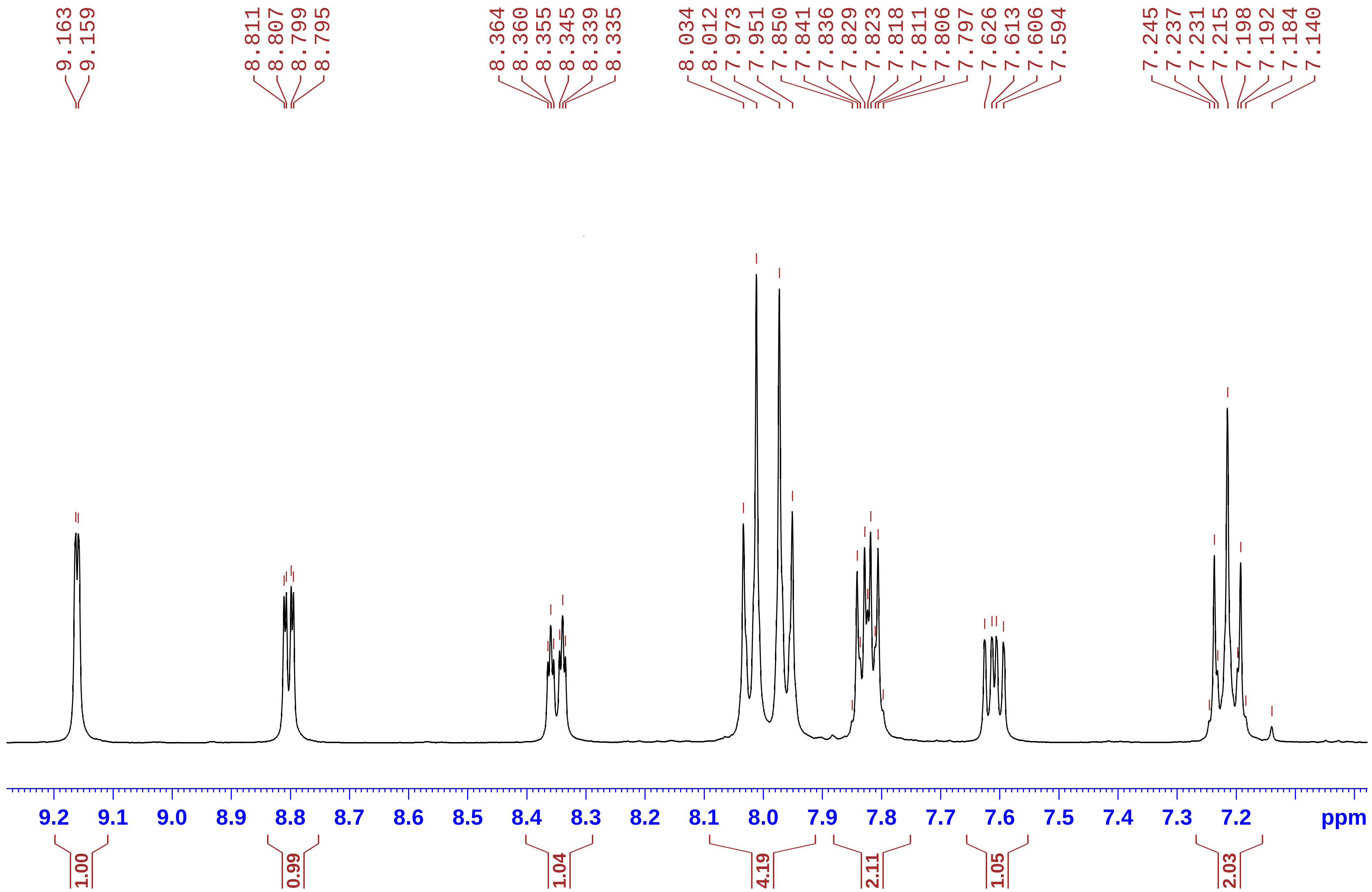

Mohamed khalifa-R21-Hnmr-ES

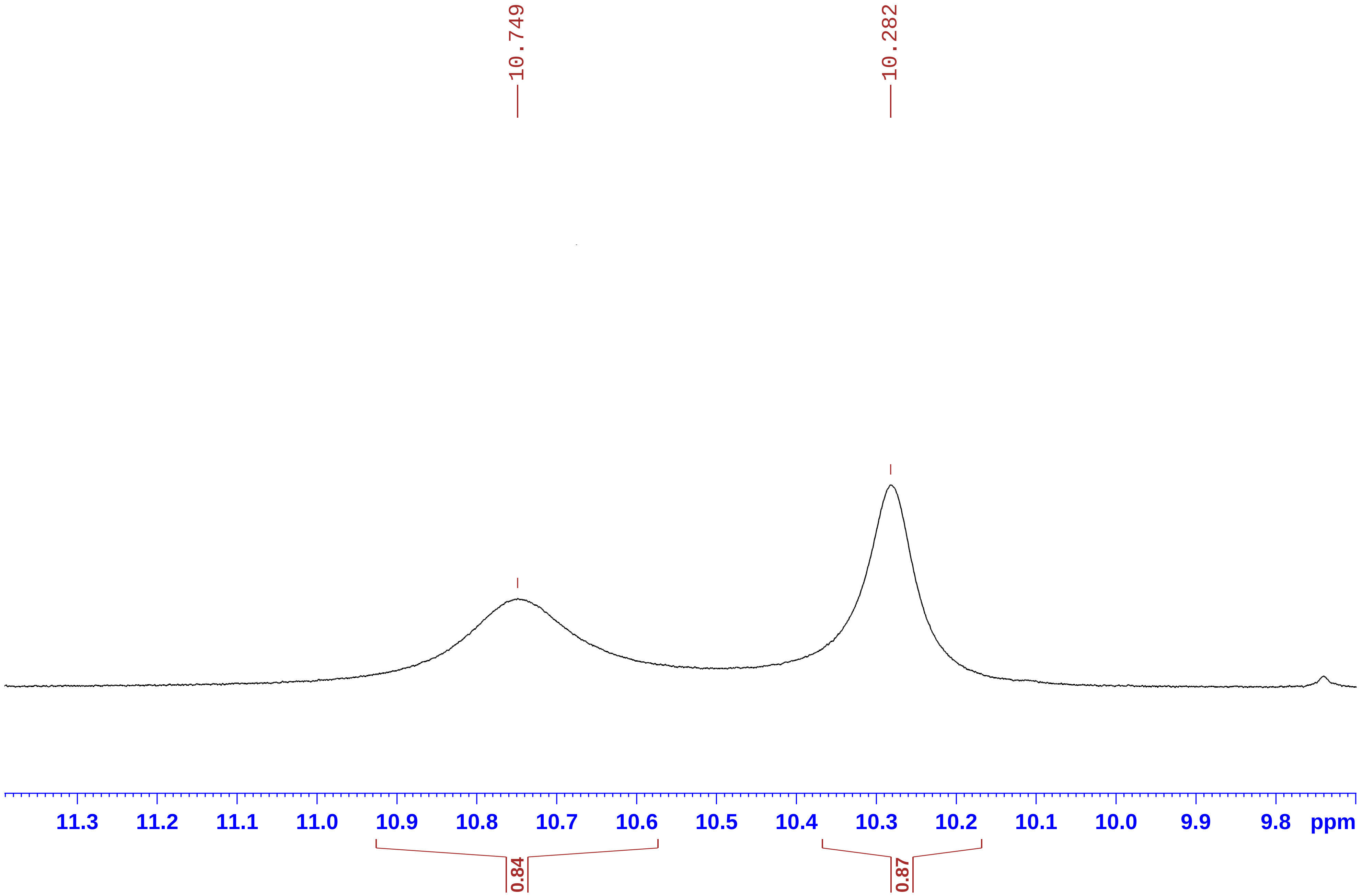

Mohamed khalifa-X1-Hnmr-ES

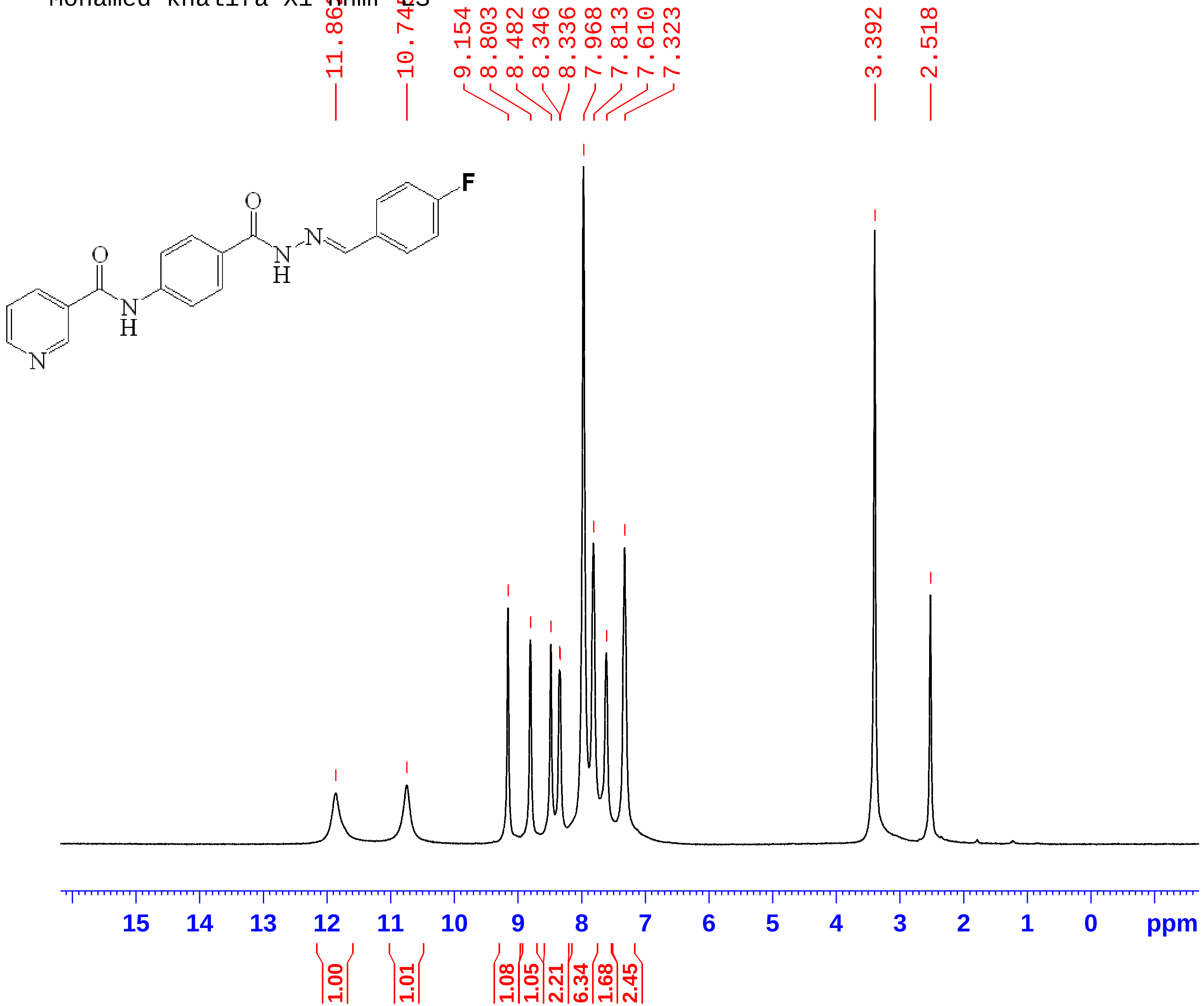

Faculty of Pharmacy

Current Data Parameters

NAME	Mohamed khalifa-X1-Hnmr-ES
EXPNO	10
PROCNO	1

F2 - Acquisition Parameters

Date_	20201126
Time	13.51 h
INSTRUM	spect
PROBHD	Z108618_0945 (
PULPROG	zg30
TD	65536
SOLVENT	DMSO
NS	16
DS	2
SWH	8012.820 Hz
FIDRES	0.244532 Hz
AQ	4.0894465 sec
RG	158.72
DW	62.400 usec
DE	6.50 usec
TE	293.3 K
D1	1.00000000 sec
TD0	1
SFO1	400.2024712 MHz
NUC1	1H
P1	13.50 usec
PLW1	13.00000000 W

F2 - Processing parameters

SI	65536
SF	400.2000000 MHz
WDW	EM
SSB	0
LB	0.30 Hz
GB	0
PC	1.00

Mohamed khalifa-X1-Hnmr-ES

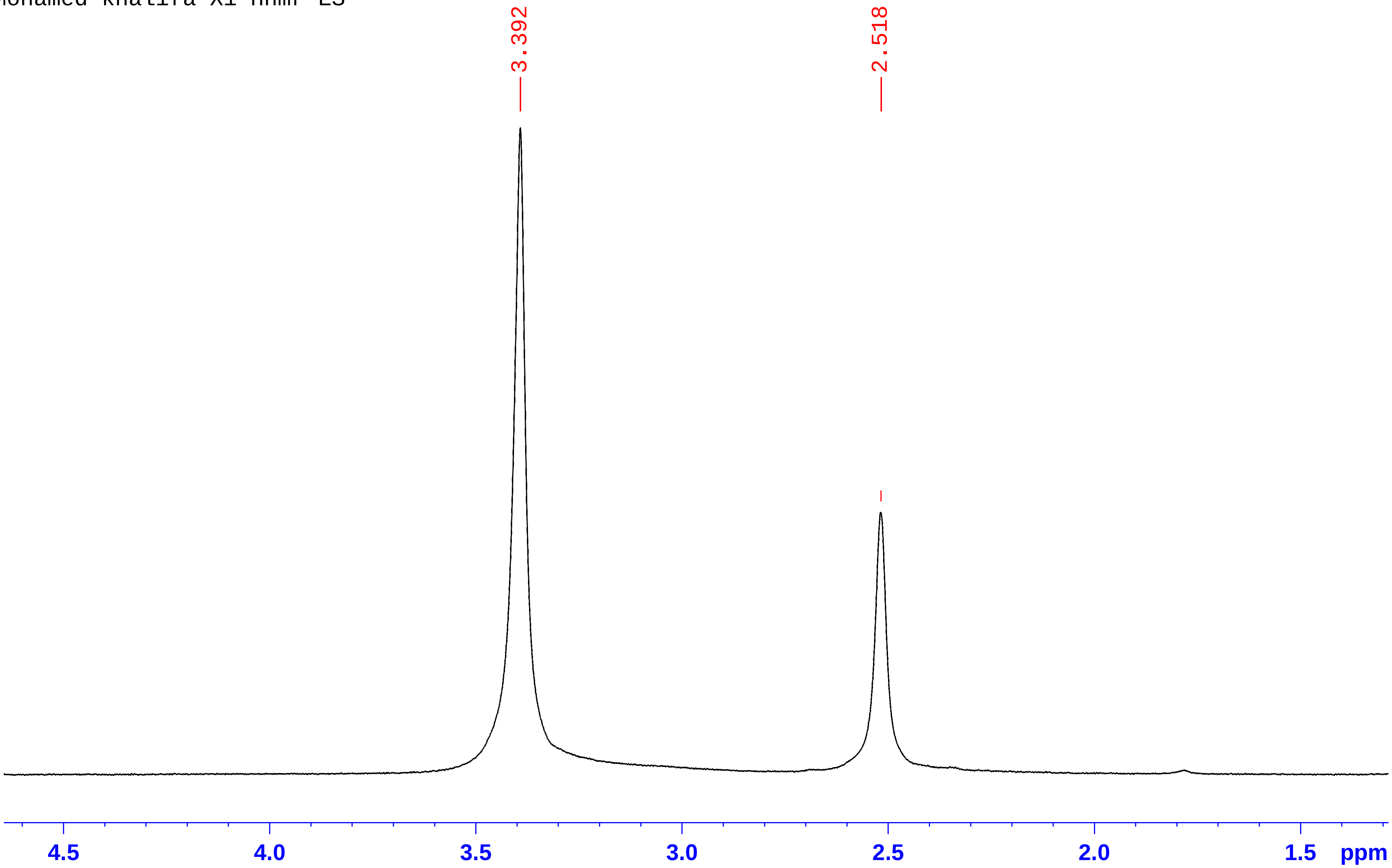

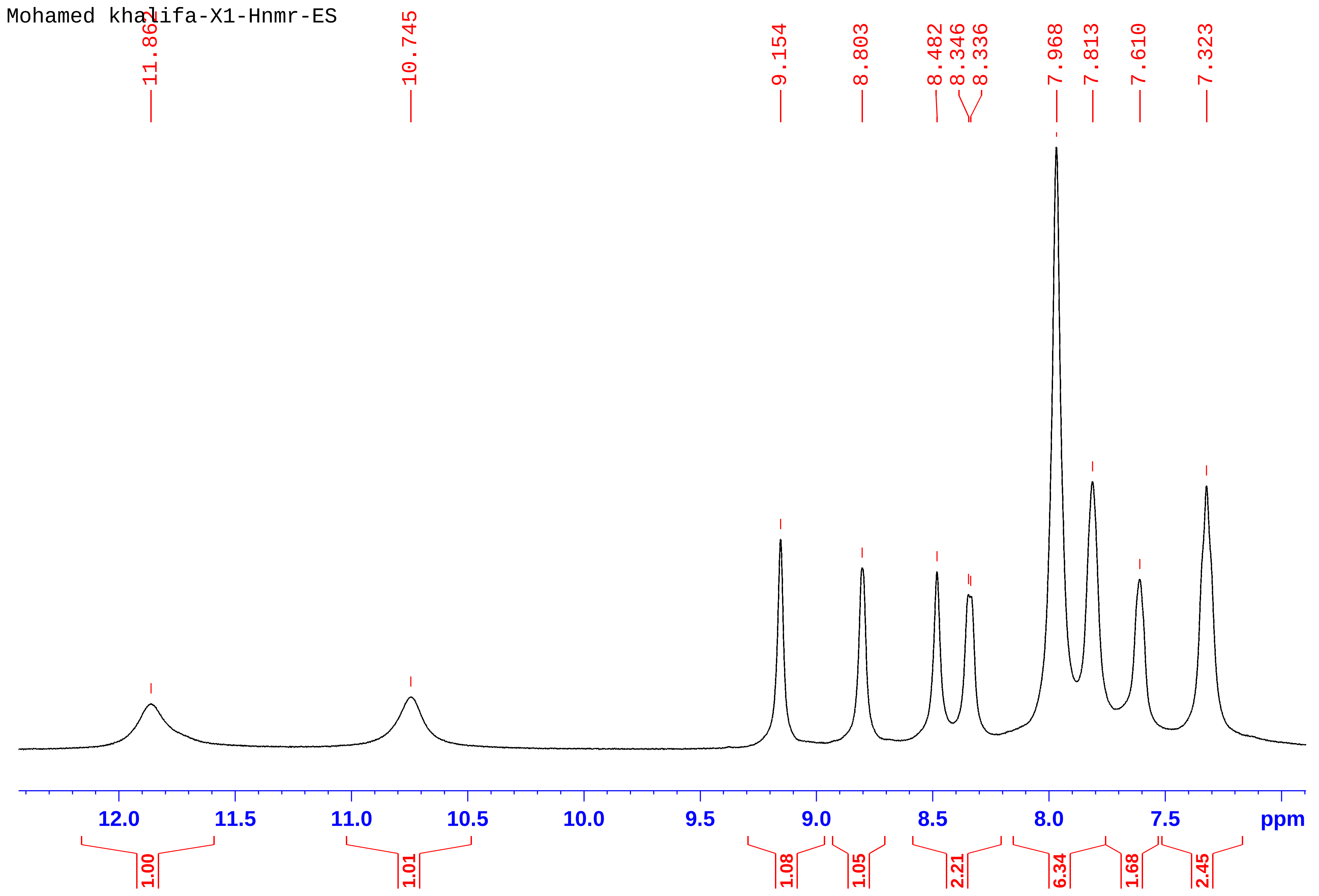
Mohamed khalifa-X1-Hnmr-ES
11.862
10.745
9.154
8.803
8.482
8.346
8.336
7.968
7.813
7.610
7.323
12.0
11.5
11.0
10.5
10.0
9.5
9.0
8.5
8.0
7.5
ppm
1.00
1.01
1.08
1.05
2.21
6.34
1.68
2.45

Mohamed khalifa-X2-Hnmr-ES

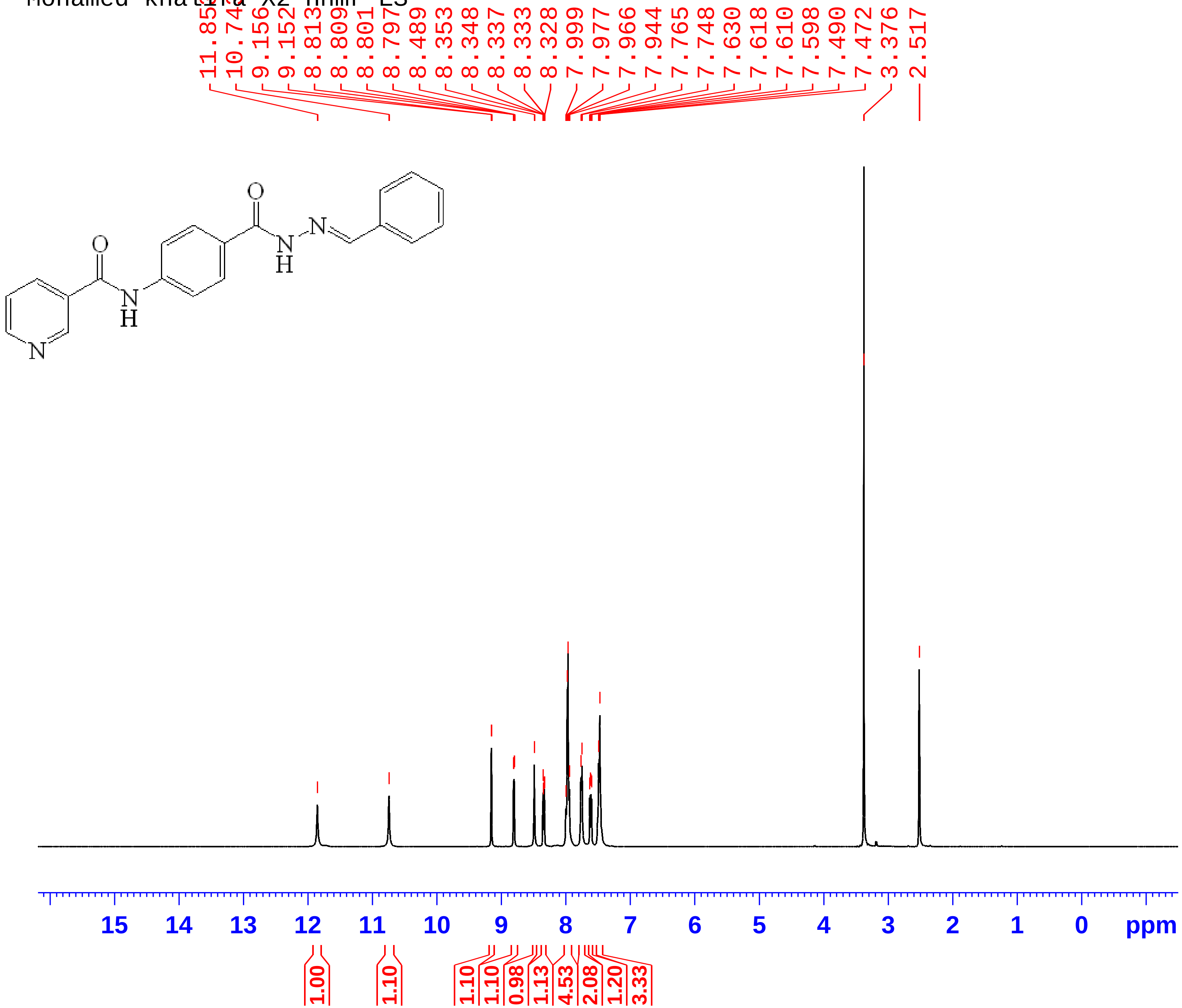

Current Data Parameters

NAME	Mohamed khalifa-X2-Hnmr-ES
EXPNO	10
PROCNO	1

F2 - Acquisition Parameters

Date_	20201126
Time	13.57 h
INSTRUM	spect
PROBHD	Z108618_0945 (
PULPROG	zg30
TD	65536
SOLVENT	DMSO
NS	16
DS	2
SWH	8012.820 Hz
FIDRES	0.244532 Hz
AQ	4.0894465 sec
RG	176.72
DW	62.400 usec
DE	6.50 usec
TE	293.3 K
D1	1.00000000 sec
TD0	1
SFO1	400.2024712 MHz
NUC1	1H
P1	13.50 usec
PLW1	13.00000000 W

F2 - Processing parameters

SI	65536
SF	400.2000000 MHz
WDW	EM
SSB	0
LB	0.30 Hz
GB	0
PC	1.00

Mohamed khalifa-X2-Hnmr-ES

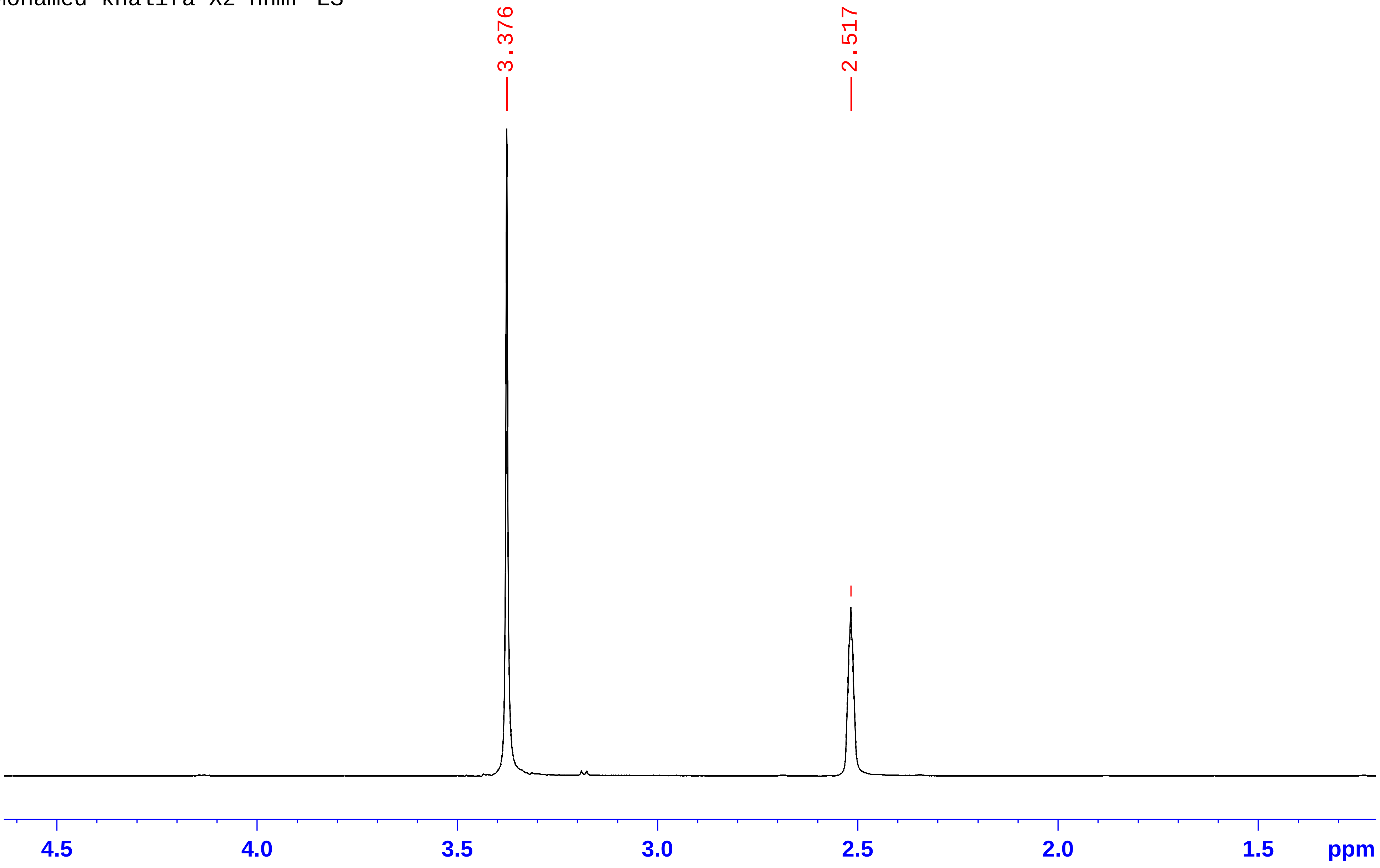

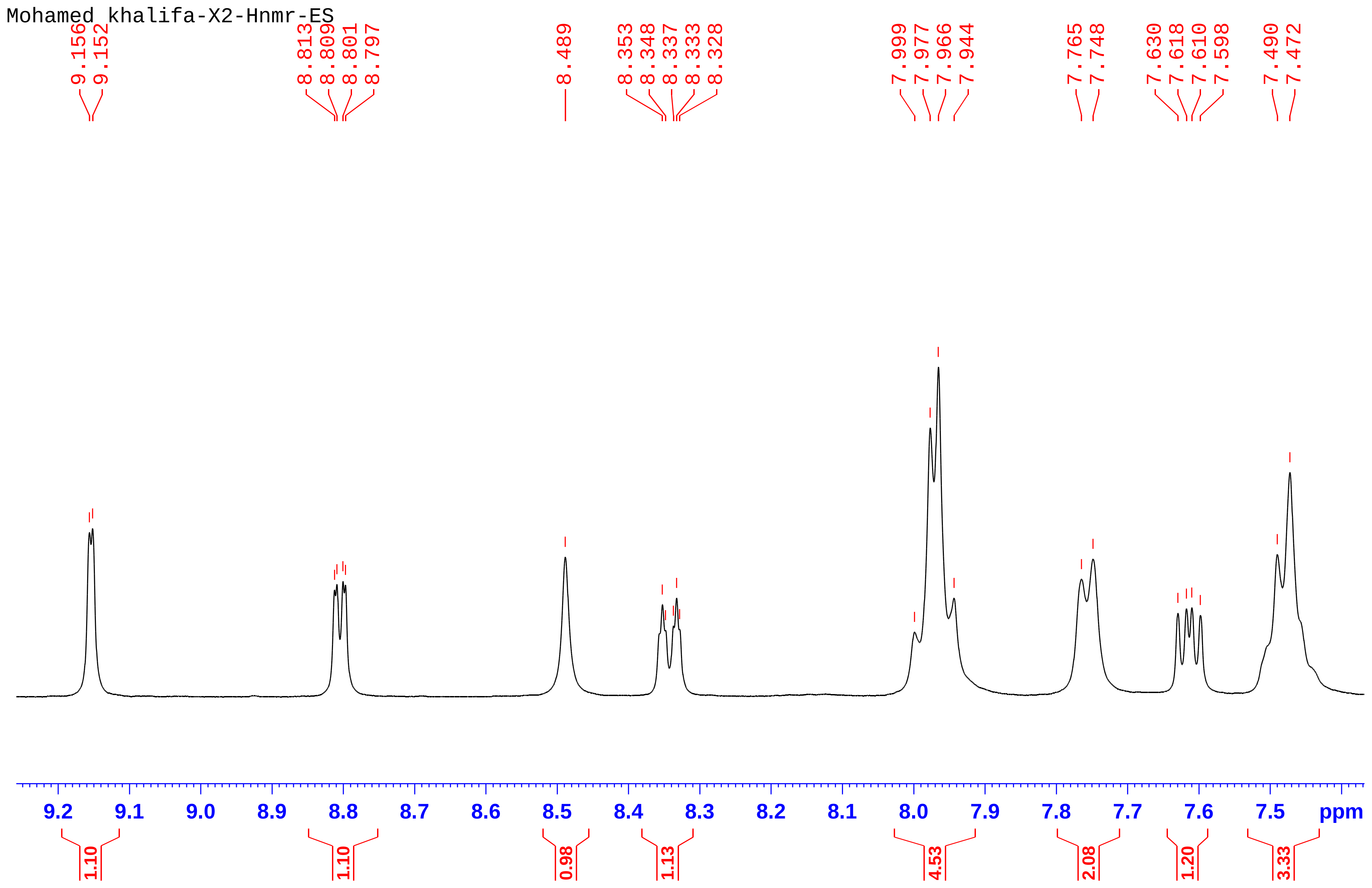
Mohamed khalifa-X2-Hnmr-ES
9.156
9.152
8.813
8.809
8.801
8.797
8.489
8.353
8.348
8.337
8.333
8.328
7.999
7.977
7.966
7.944
7.765
7.748
7.630
7.618
7.610
7.598
7.490
7.472
9.2
9.1
9.0
8.9
8.8
8.7
8.6
8.5
8.4
8.3
8.2
8.1
8.0
7.9
7.8
7.7
7.6
7.5
ppm
1.10
1.10
0.98
1.13
4.53
2.08
1.20
3.33

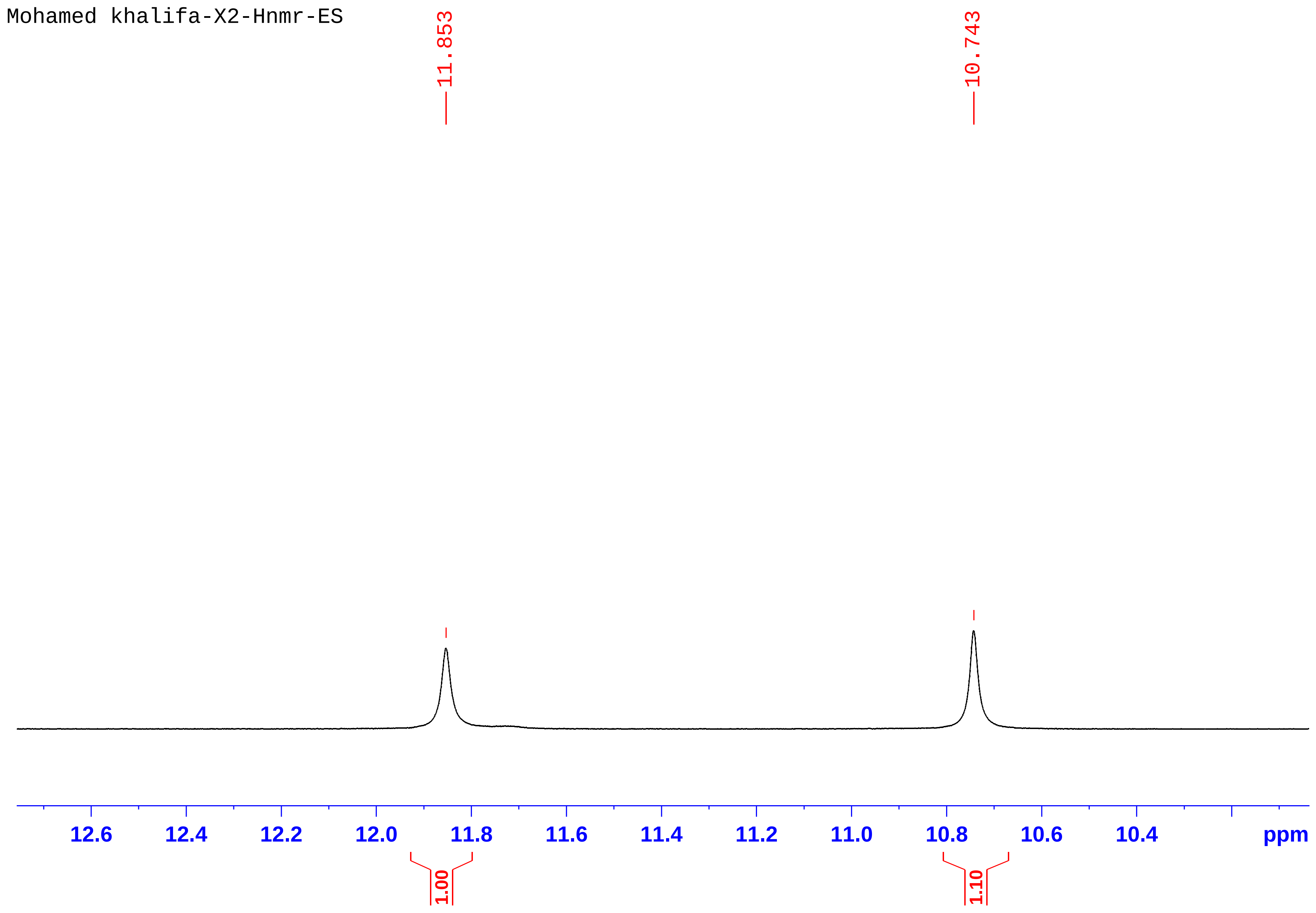

Mohamed khalifa-X2-Hnmr-ES
11.853
10.743
12.6
12.4
12.2
12.0
11.8
11.6
11.4
11.2
11.0
10.8
10.6
10.4
ppm
1.00
1.10

Mohamed khalifa-X2-Hnmr-ES

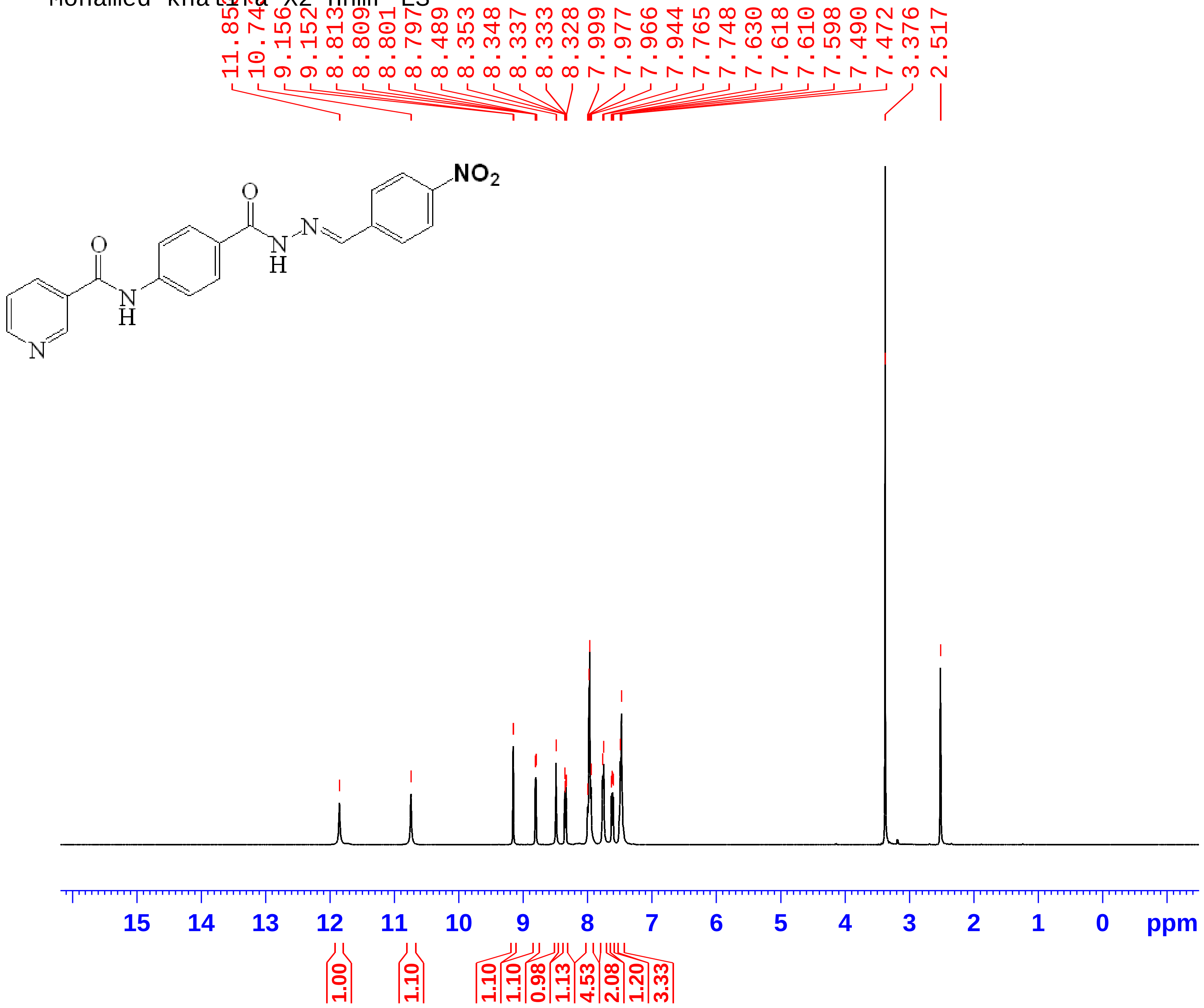

Faculty of Pharmacy

Current Data Parameters

NAME	Mohamed khalifa-X2-Hnmr-ES
EXPNO	10
PROCNO	1

F2 - Acquisition Parameters

Date_	20201126
Time	13.57 h
INSTRUM	spect
PROBHD	Z108618_0945 (
PULPROG	zg30
TD	65536
SOLVENT	DMSO
NS	16
DS	2
SWH	8012.820 Hz
FIDRES	0.244532 Hz
AQ	4.0894465 sec
RG	176.72
DW	62.400 usec
DE	6.50 usec
TE	293.3 K
D1	1.00000000 sec
TD0	1
SFO1	400.2024712 MHz
NUC1	1H
P1	13.50 usec
PLW1	13.00000000 W

F2 - Processing parameters

SI	65536
SF	400.2000000 MHz
WDW	EM
SSB	0
LB	0.30 Hz
GB	0
PC	1.00

Mohamed khalifa-X2-Hnmr-ES

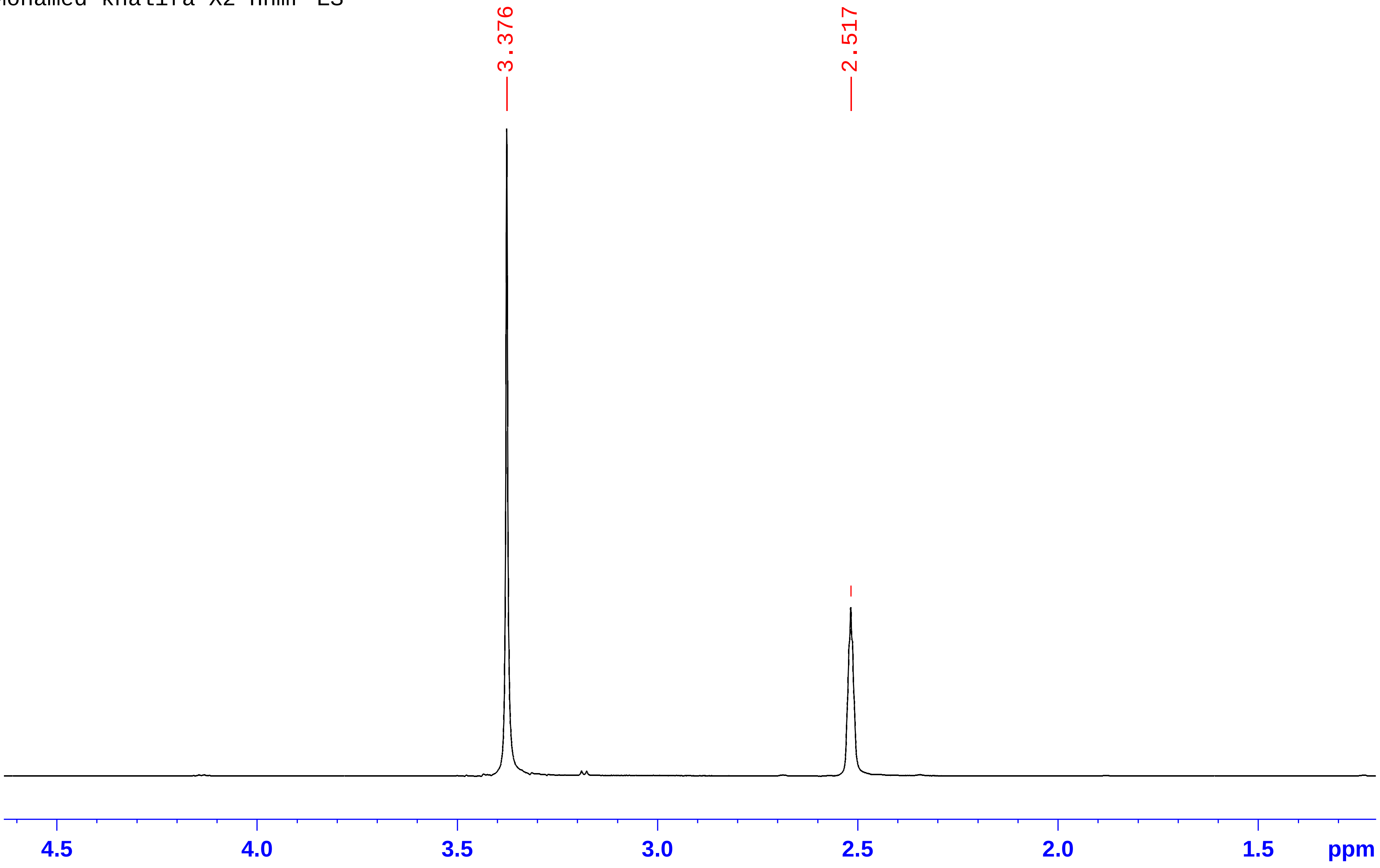

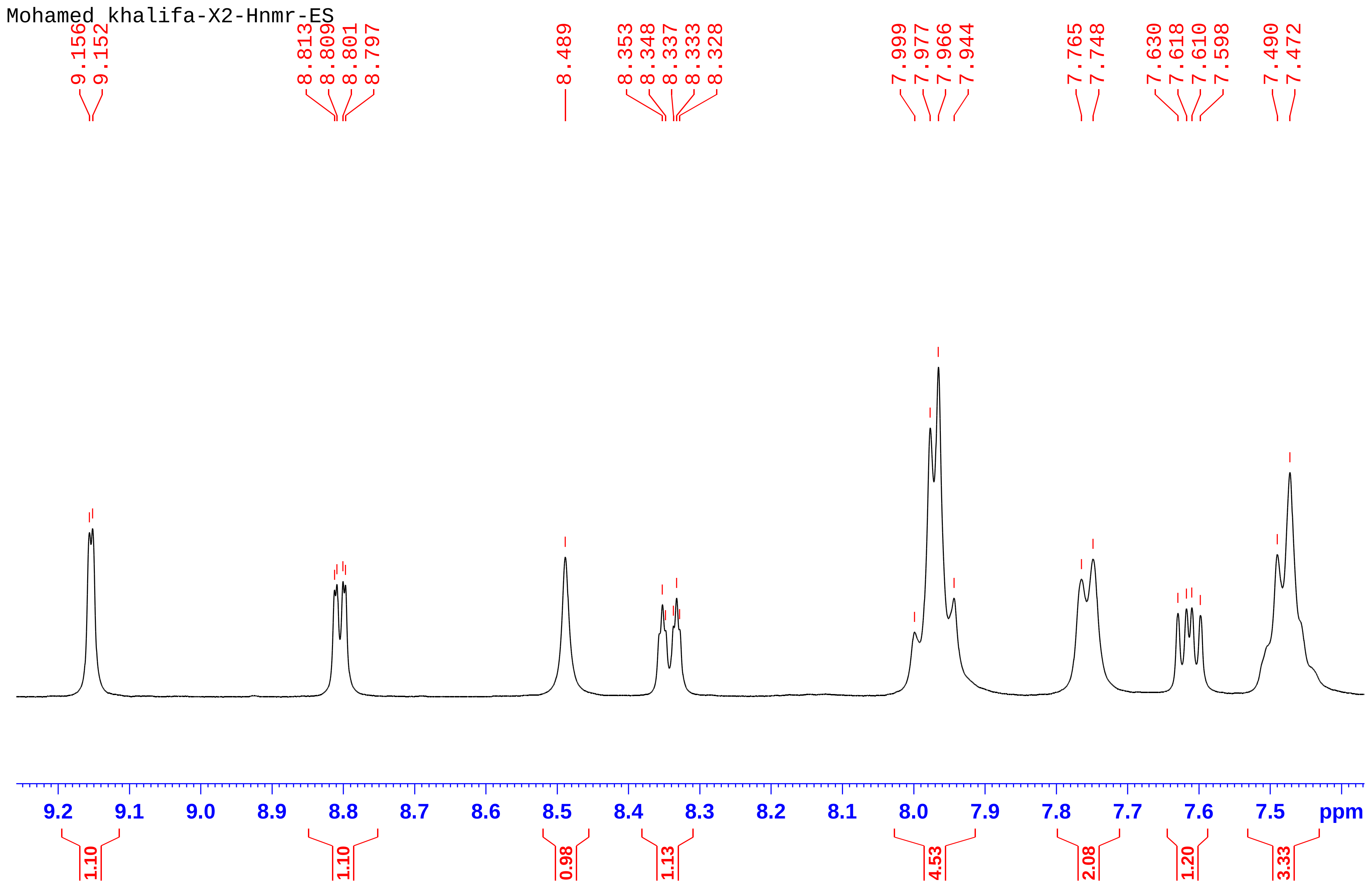

Mohamed khalifa-X2-Hnmr-ES
9.156
9.152
8.813
8.809
8.801
8.797
8.489
8.353
8.348
8.337
8.333
8.328
7.999
7.977
7.966
7.944
7.765
7.748
7.630
7.618
7.610
7.598
7.490
7.472
9.2
9.1
9.0
8.9
8.8
8.7
8.6
8.5
8.4
8.3
8.2
8.1
8.0
7.9
7.8
7.7
7.6
7.5
ppm
1.10
1.10
0.98
1.13
4.53
2.08
1.20
3.33

Mohamed khalifa-X2-Hnmr-ES

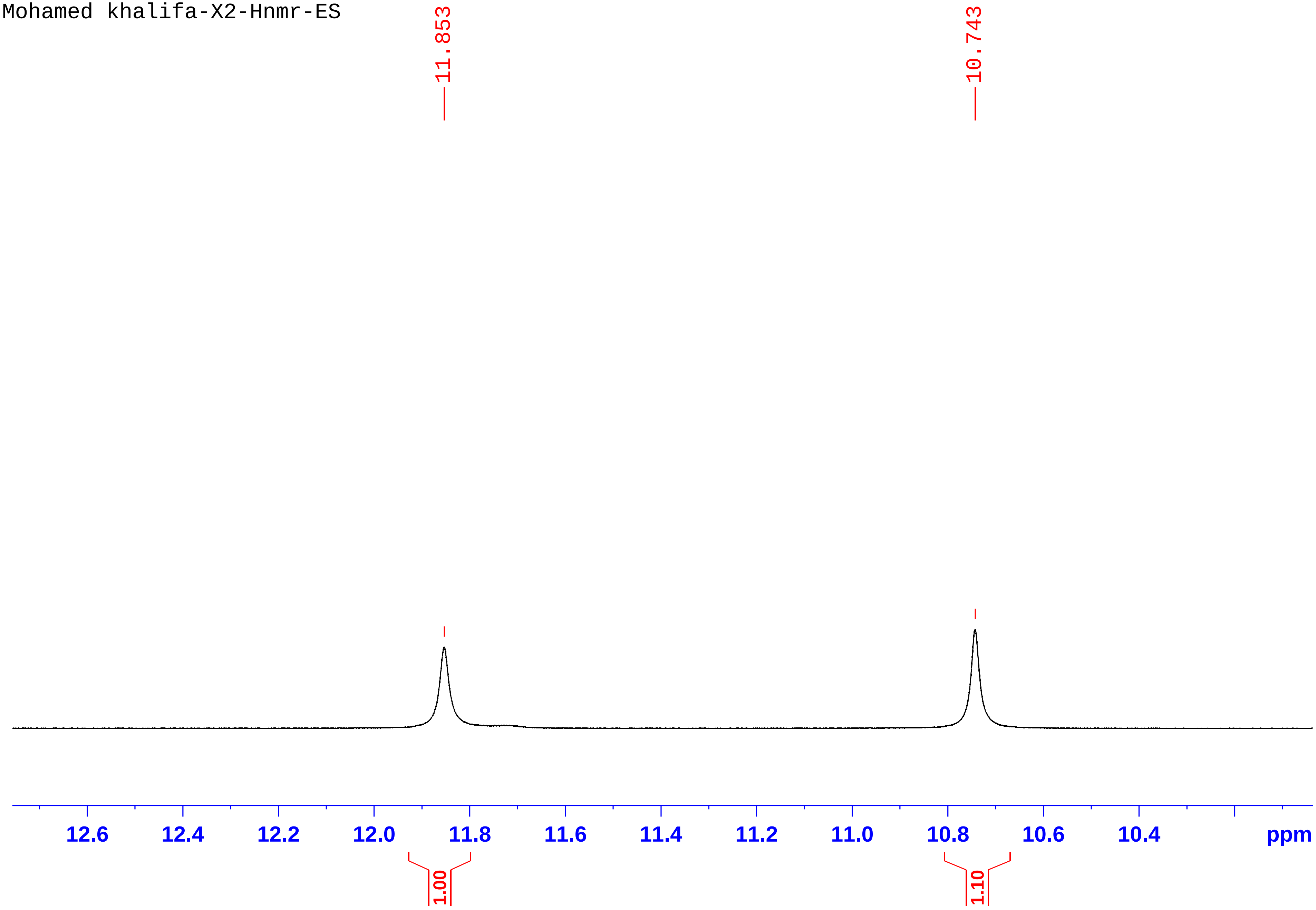

Mohamed khalifa-X3-Hnmr-ES

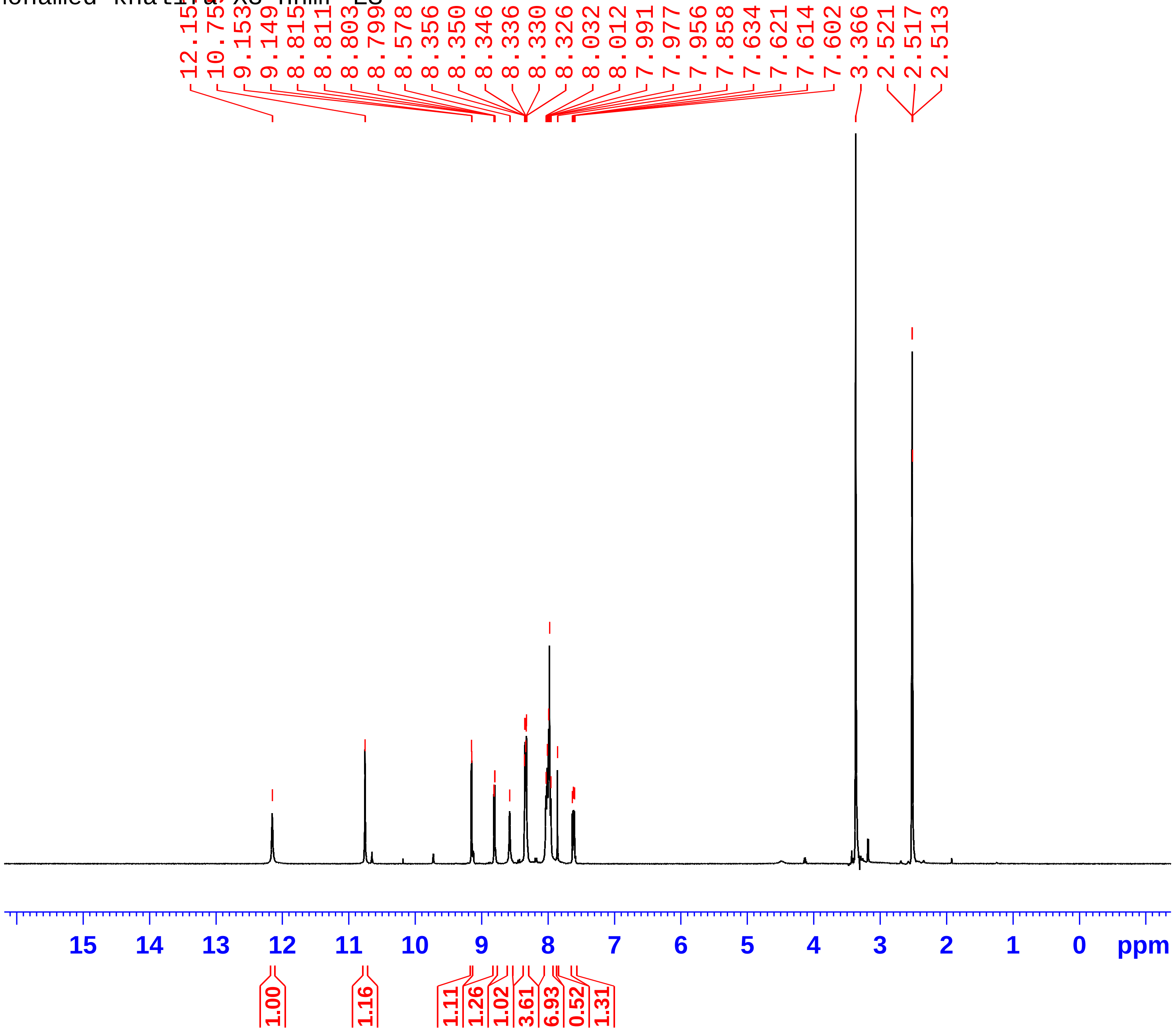

Current Data Parameters
NAME Mohamed khalifa-X3-Hnmr-ES
EXPNO 10
PROCNO 1

F2 - Acquisition Parameters
Date_ 20201126
Time 14.02 h
INSTRUM spect
PROBHD Z108618_0945 (
PULPROG zg30
TD 65536
SOLVENT DMSO
NS 16
DS 2
SWH 8012.820 Hz
FIDRES 0.244532 Hz
AQ 4.0894465 sec
RG 197.77
DW 62.400 usec
DE 6.50 usec
TE 293.3 K
D1 1.00000000 sec
TD0 1
SFO1 400.2024712 MHz
NUC1 1H
P1 13.50 usec
PLW1 13.00000000 W

F2 - Processing parameters
SI 65536
SF 400.2000000 MHz
WDW EM
SSB 0
LB 0.30 Hz
GB 0
PC 1.00

Mohamed khalifa-X5-Hnmr-ES

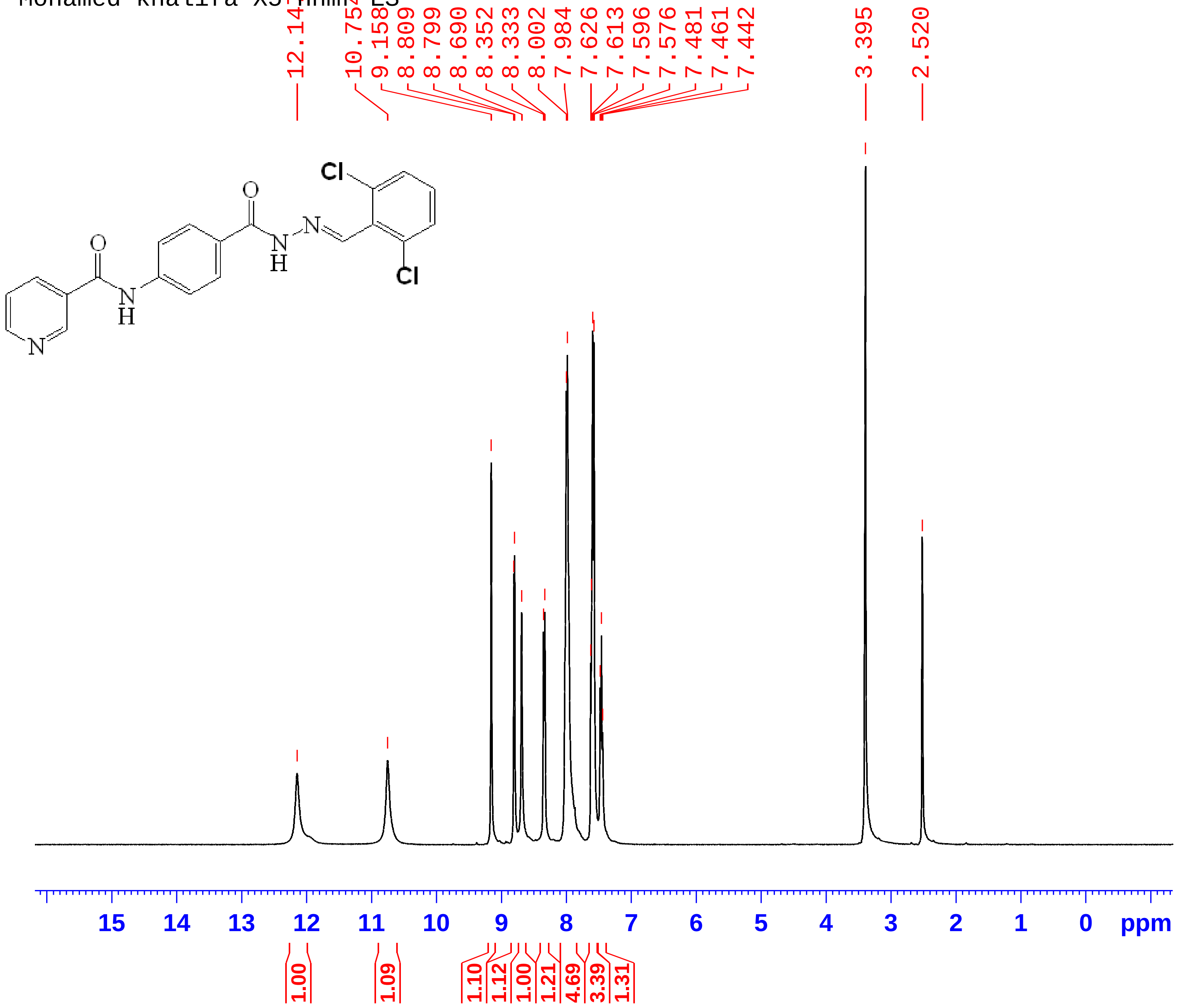

BRUKER

Faculty of Pharmacy

Current Data Parameters

NAME	Mohamed khalifa-X5-Hnmr-ES
EXPNO	10
PROCNO	1

F2 - Acquisition Parameters

Date_	20201126
Time	14.06 h
INSTRUM	spect
PROBHD	Z108618_0945 (
PULPROG	zg30
TD	65536
SOLVENT	DMSO
NS	16
DS	2
SWH	8012.820 Hz
FIDRES	0.244532 Hz
AQ	4.0894465 sec
RG	112.56
DW	62.400 usec
DE	6.50 usec
TE	293.3 K
D1	1.00000000 sec
TD0	1
SFO1	400.2024712 MHz
NUC1	1H
P1	13.50 usec
PLW1	13.00000000 W

F2 - Processing parameters

SI	65536
SF	400.2000000 MHz
WDW	EM
SSB	0
LB	0.30 Hz
GB	0
PC	1.00

Mohamed khalifa-X5-Hnmr-ES

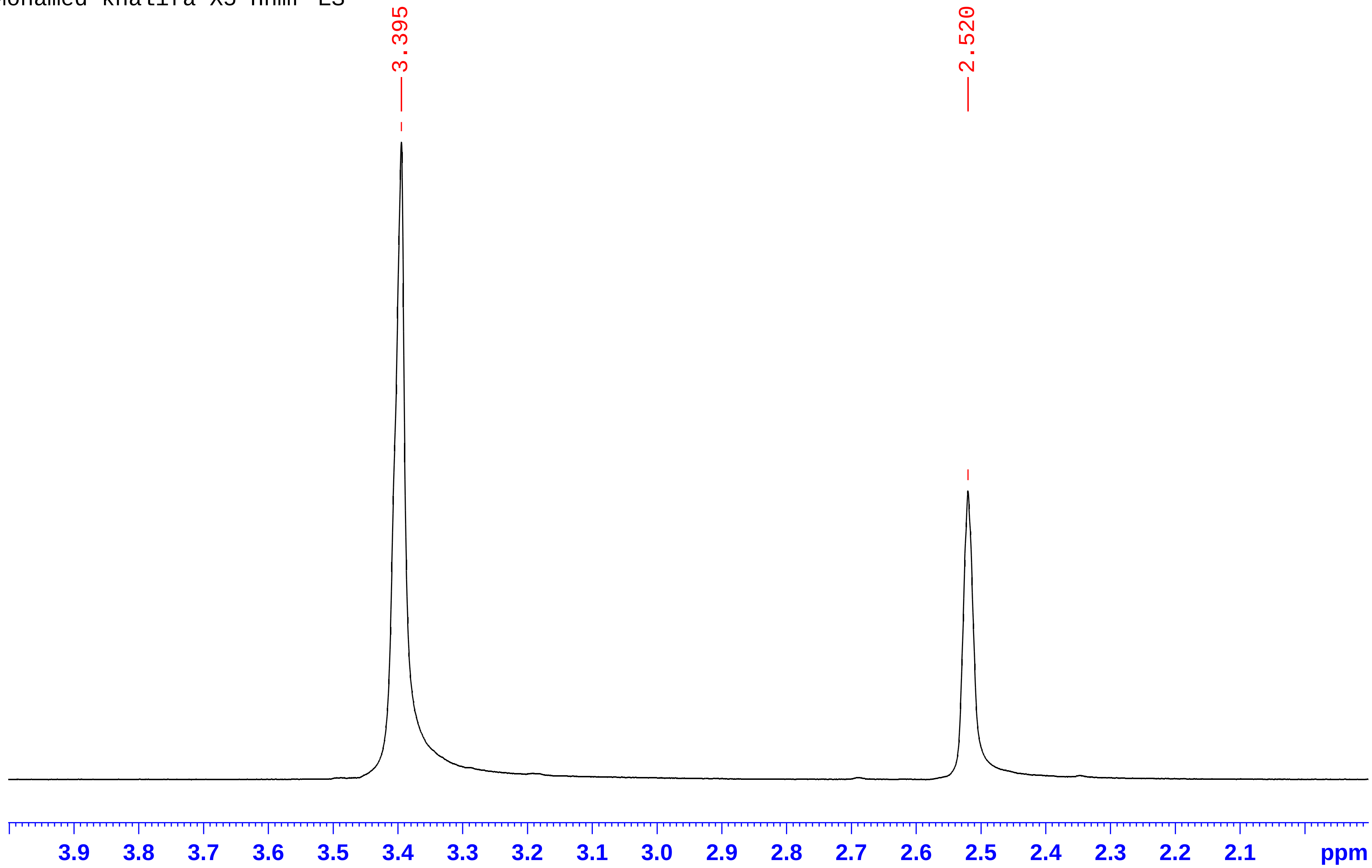

Mohamed khalifa-X5-Hnmr-ES

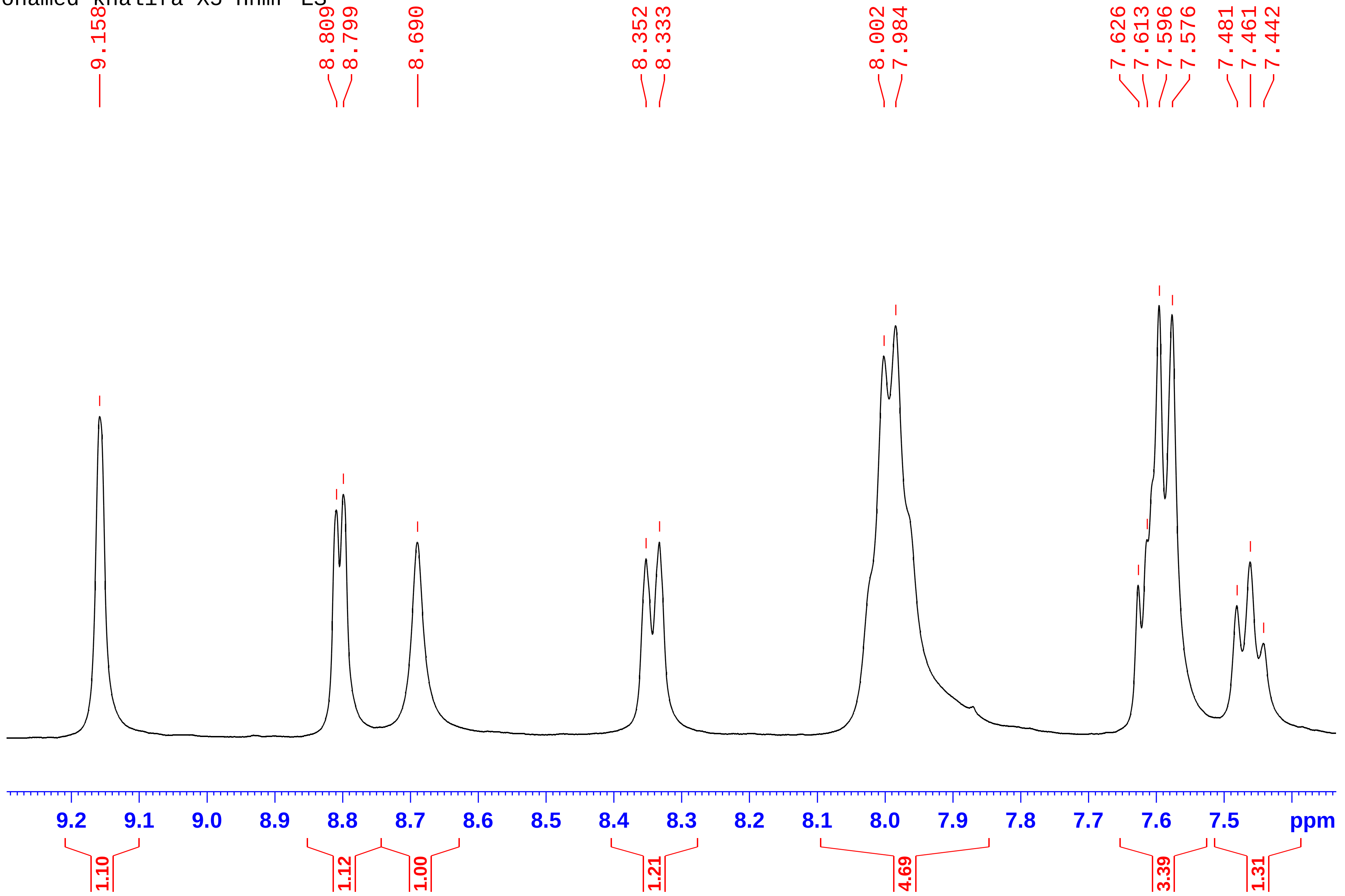

Mohamed khalifa-X5-Hnmr-ES

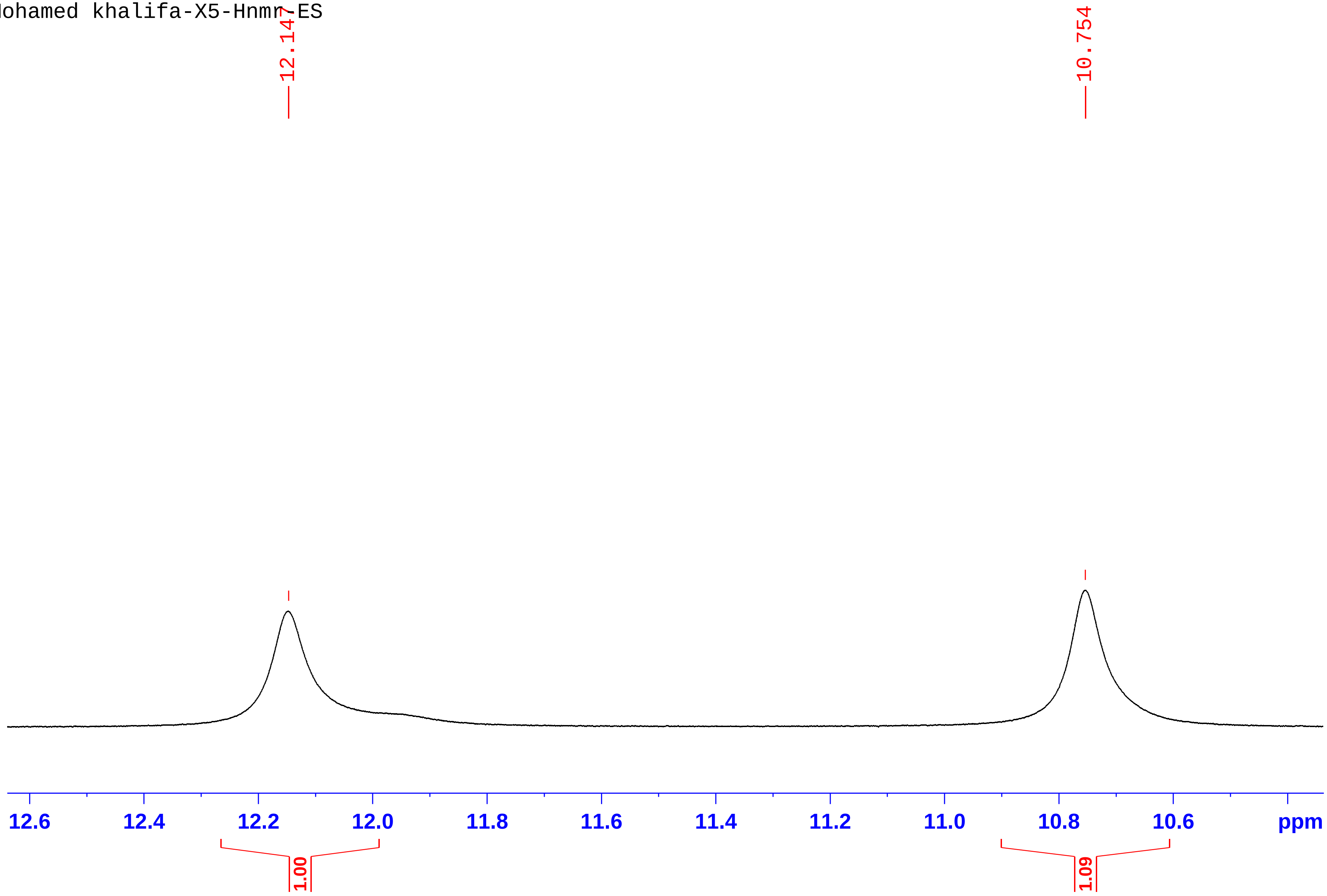

Mohamed khalifa-X6-Hnmr-ES

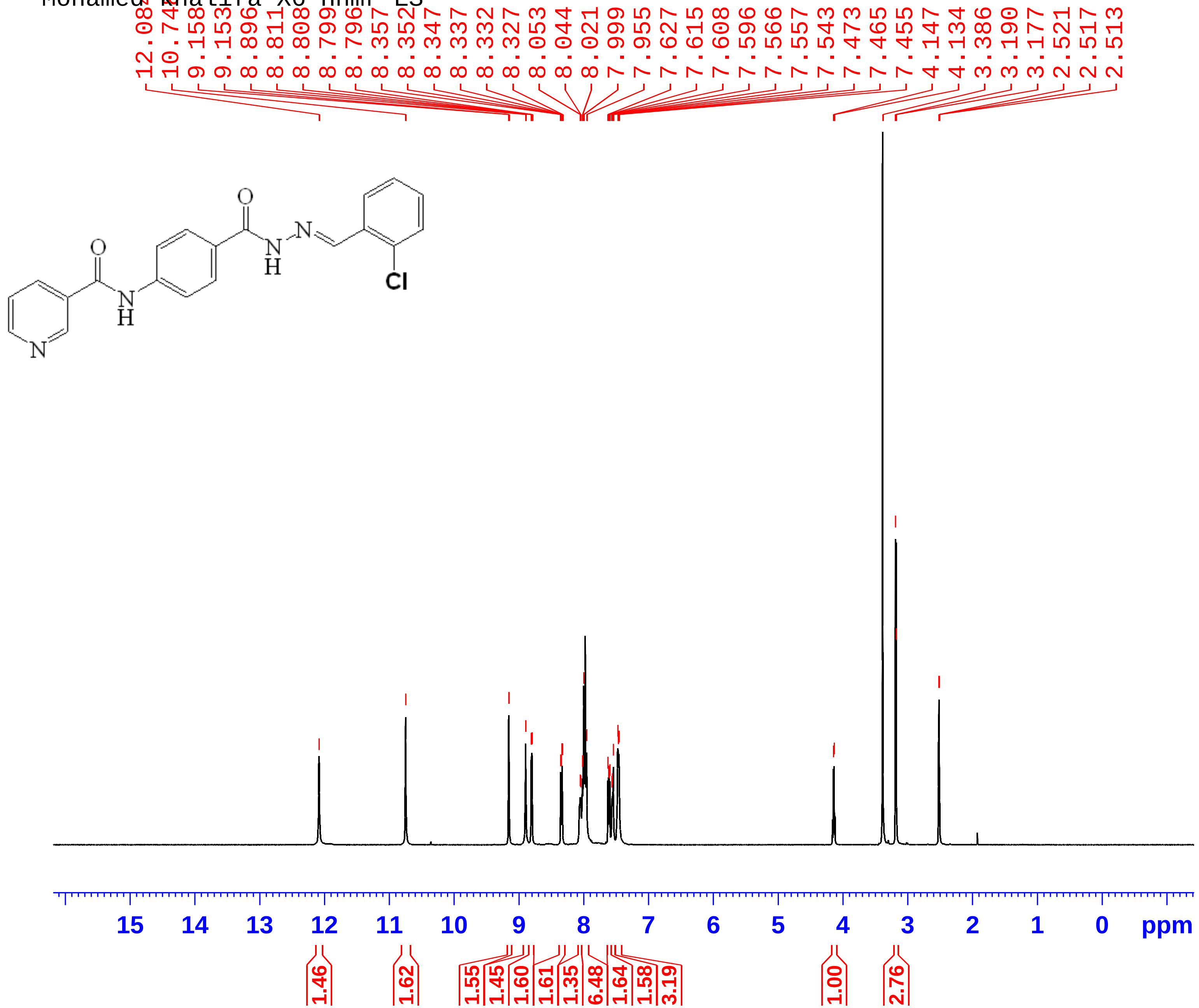

BRUKER

Faculty of Pharmacy

Current Data Parameters
NAME Mohamed khalifa-X6-Hnmr-ES
EXPNO 10
PROCNO 1

F2 - Acquisition Parameters
Date_ 20201126
Time 14.11 h
INSTRUM spect
PROBHD Z108618_0945 (
PULPROG zg30
TD 65536
SOLVENT DMSO
NS 16
DS 2
SWH 8012.820 Hz
FIDRES 0.244532 Hz
AQ 4.0894465 sec
RG 120.93
DW 62.400 usec
DE 6.50 usec
TE 293.2 K
D1 1.00000000 sec
TD0 1
SFO1 400.2024712 MHz
NUC1 1H
P1 13.50 usec
PLW1 13.00000000 W

F2 - Processing parameters
SI 65536
SF 400.2000000 MHz
WDW EM
SSB 0
LB 0.30 Hz
GB 0
PC 1.00

Mohamed khalifa-X6-Hnmr-ES

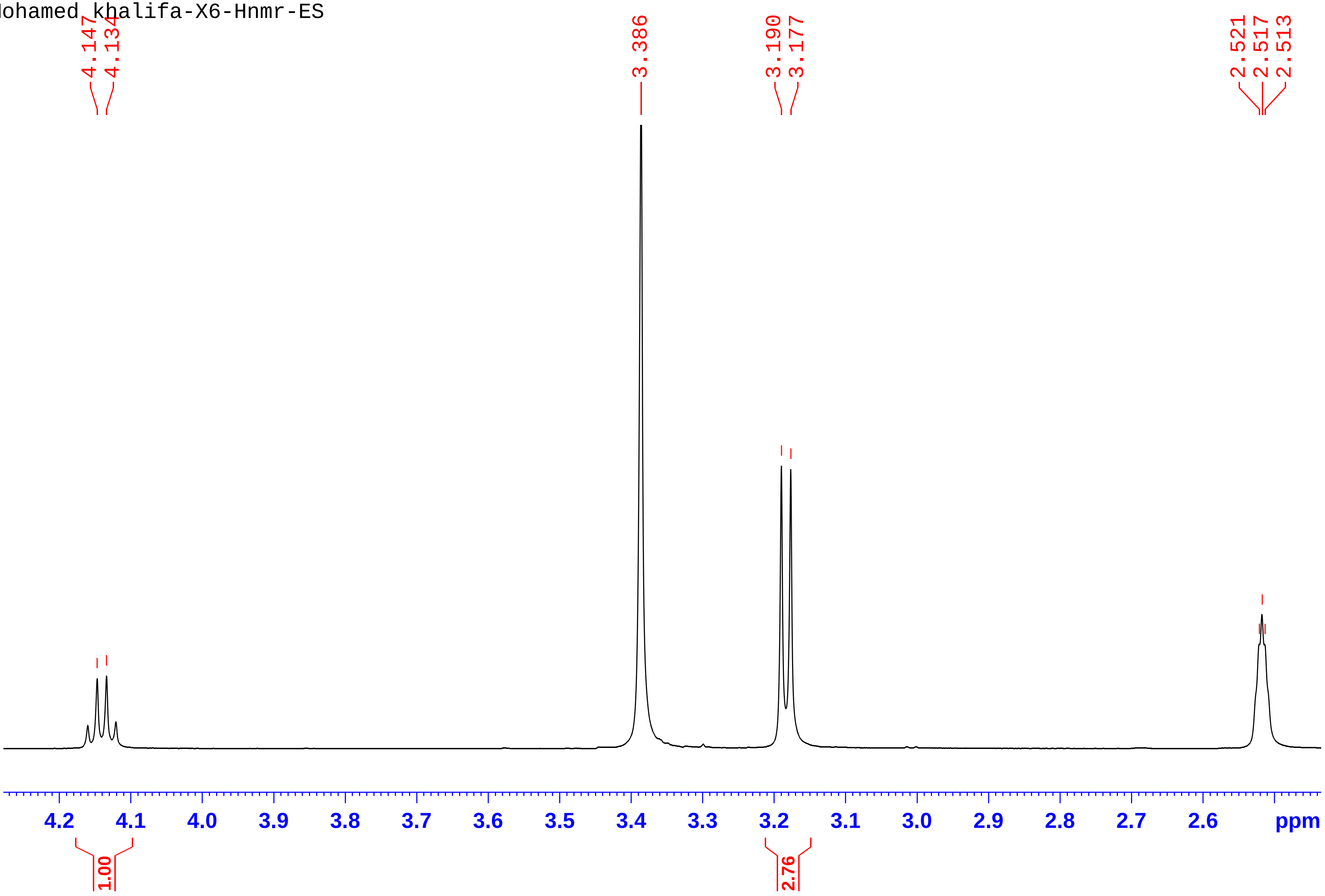

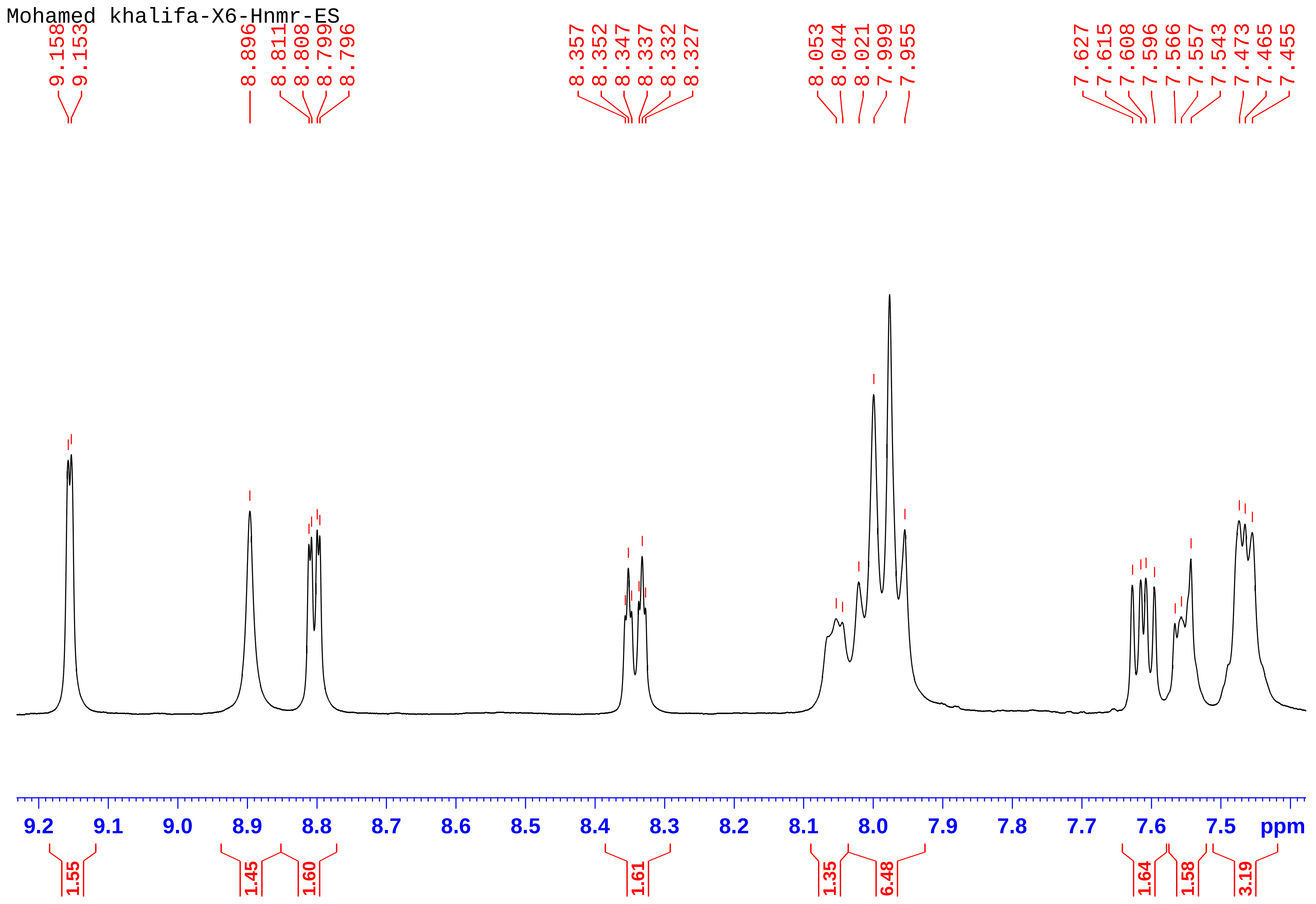

Mohamed khalifa-X6-Hnmr-ES
9.158
9.153
8.896
8.811
8.808
8.799
8.796
8.357
8.352
8.347
8.337
8.332
8.327
8.053
8.044
8.021
7.999
7.955
7.627
7.615
7.608
7.596
7.566
7.557
7.543
7.473
7.465
7.455
9.2
9.1
9.0
8.9
8.8
8.7
8.6
8.5
8.4
8.3
8.2
8.1
8.0
7.9
7.8
7.7
7.6
7.5
ppm
1.55
1.45
1.60
1.61
1.35
6.48
1.64
1.58
3.19

Mohamed khalifa-X6-Hnmr-ES

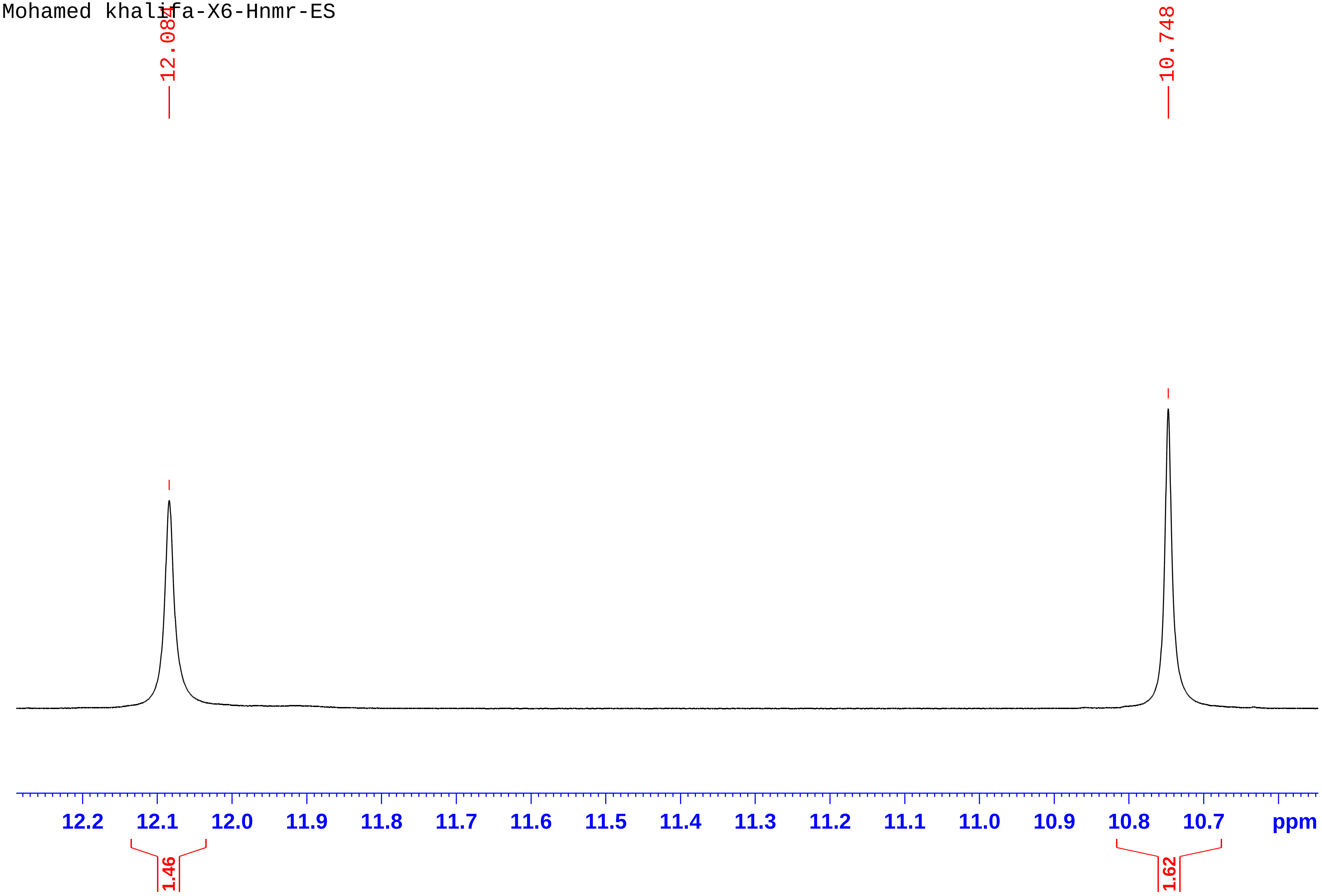

Mohamed khalifa-X7-Hnmr-ES

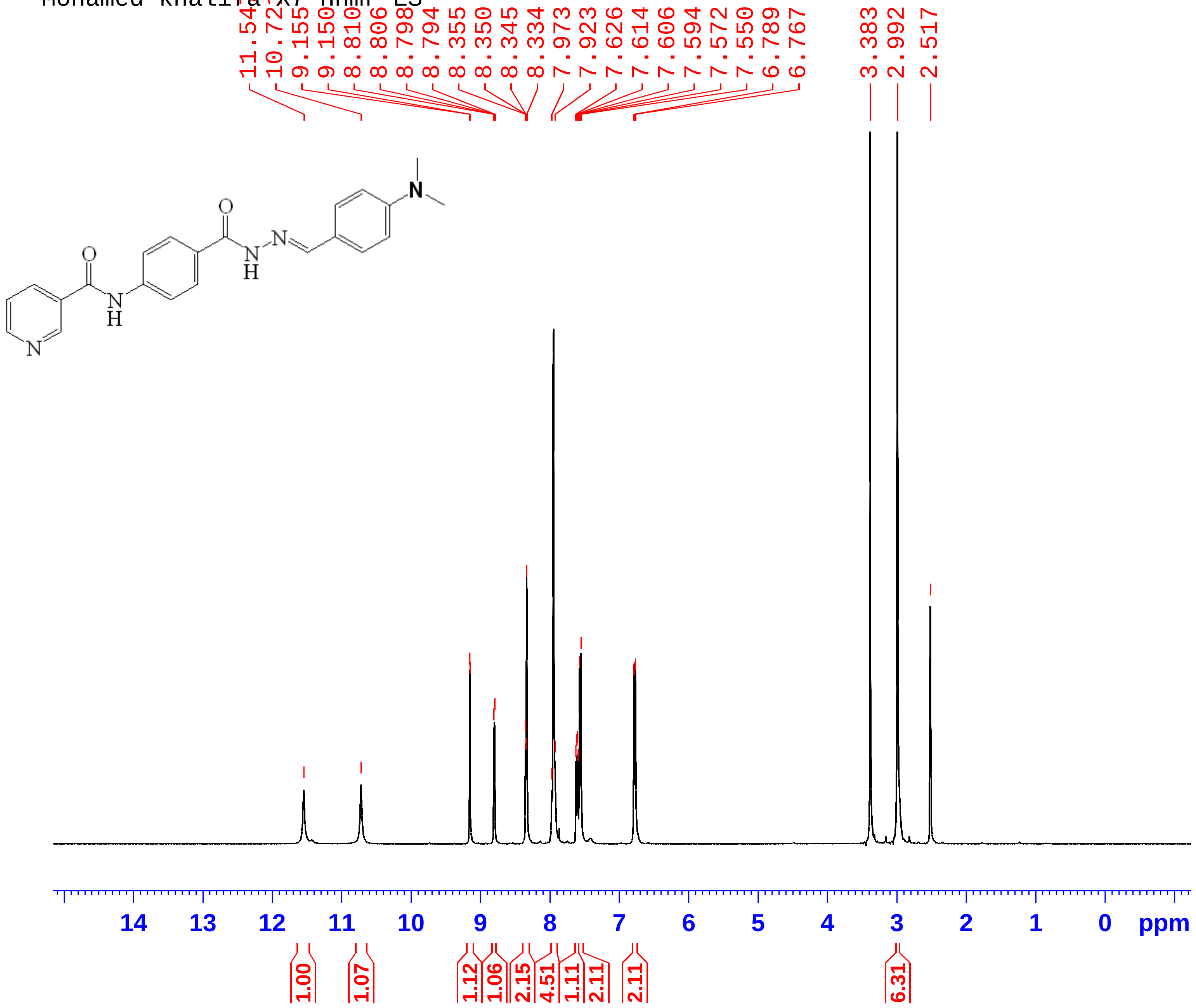

Current Data Parameters	
NAME	Mohamed khalifa-X7-Hnmr-ES
EXPNO	10
PROCNO	1
F2 - Acquisition Parameters	
Date_	20201126
Time	14.16 h
INSTRUM	spect
PROBHD	Z108618_0945 (
PULPROG	zg30
TD	65536
SOLVENT	DMSO
NS	16
DS	2
SWH	8012.820 Hz
FIDRES	0.244532 Hz
AQ	4.0894465 sec
RG	120.93
DW	62.400 usec
DE	6.50 usec
TE	293.2 K
D1	1.00000000 sec
TD0	1
SFO1	400.2024712 MHz
NUC1	1H
P1	13.50 usec
PLW1	13.00000000 W
F2 - Processing parameters	
SI	65536
SF	400.2000000 MHz
WDW	EM
SSB	0
LB	0.30 Hz
GB	0
PC	1.00

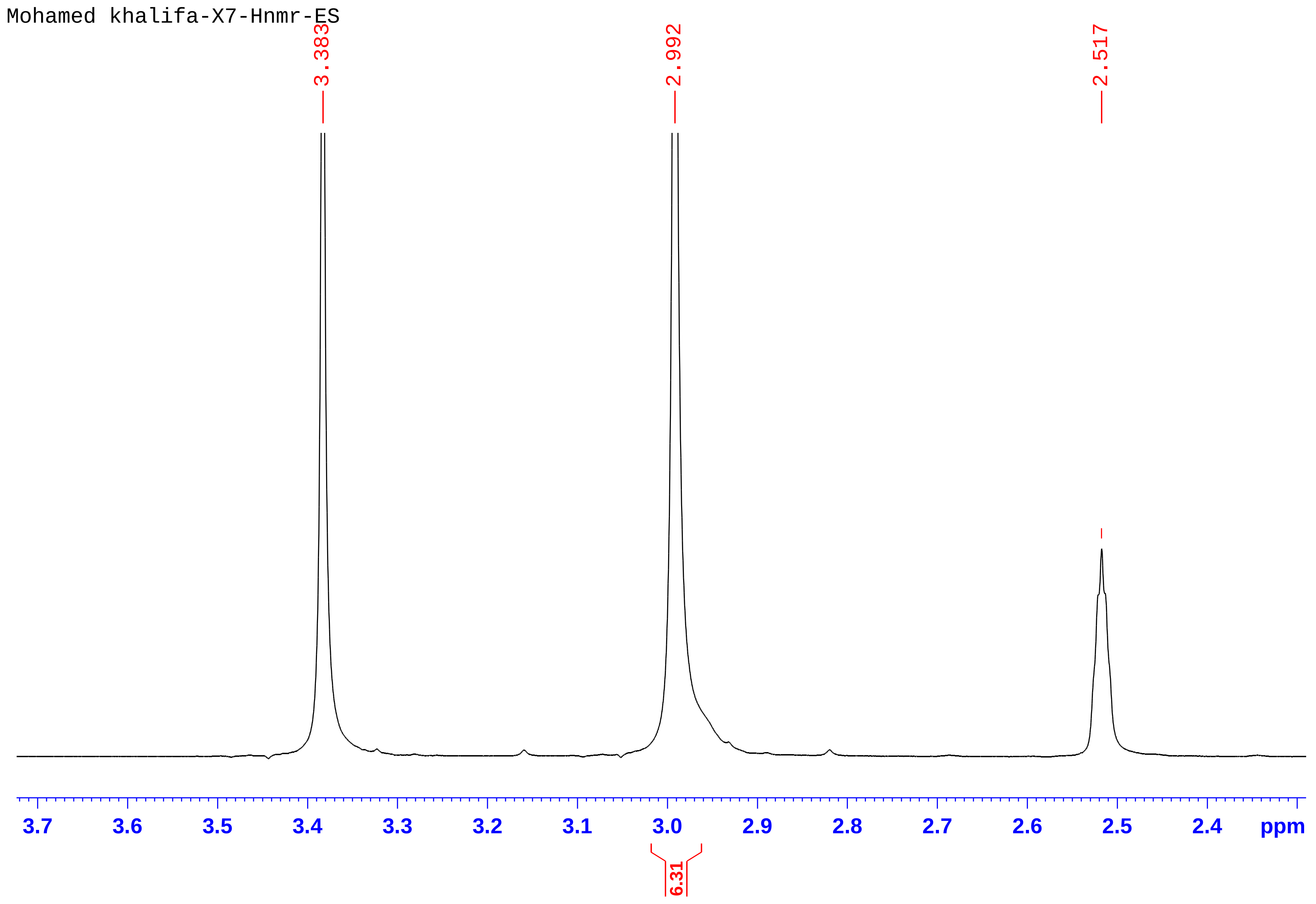

Mohamed khalifa-X7-Hnmr-ES
3.383
2.992
2.517
3.7
3.6
3.5
3.4
3.3
3.2
3.1
3.0
2.9
2.8
2.7
2.6
2.5
2.4
ppm
6.31

Mohamed khalifa-X7-Hnmr-ES

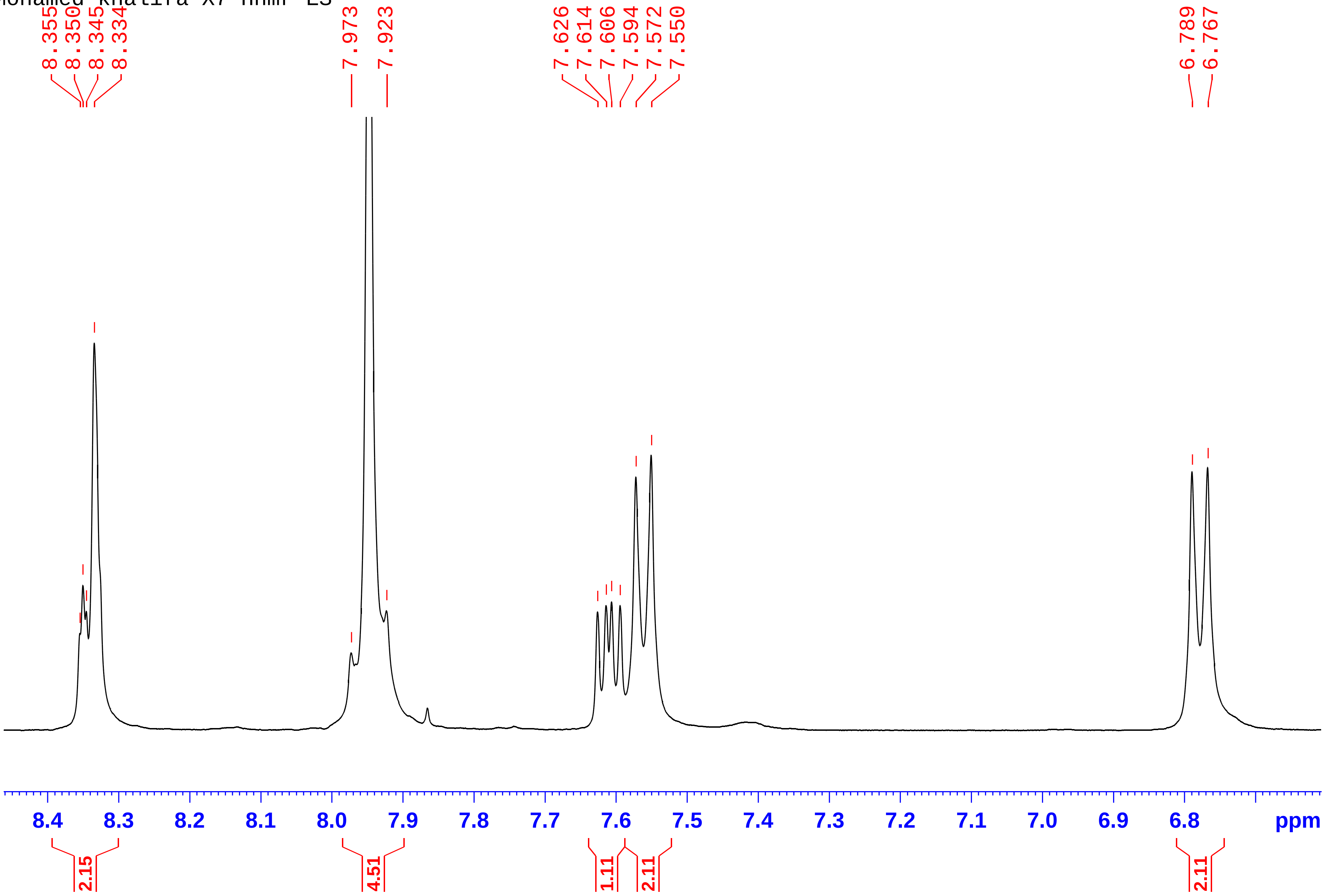

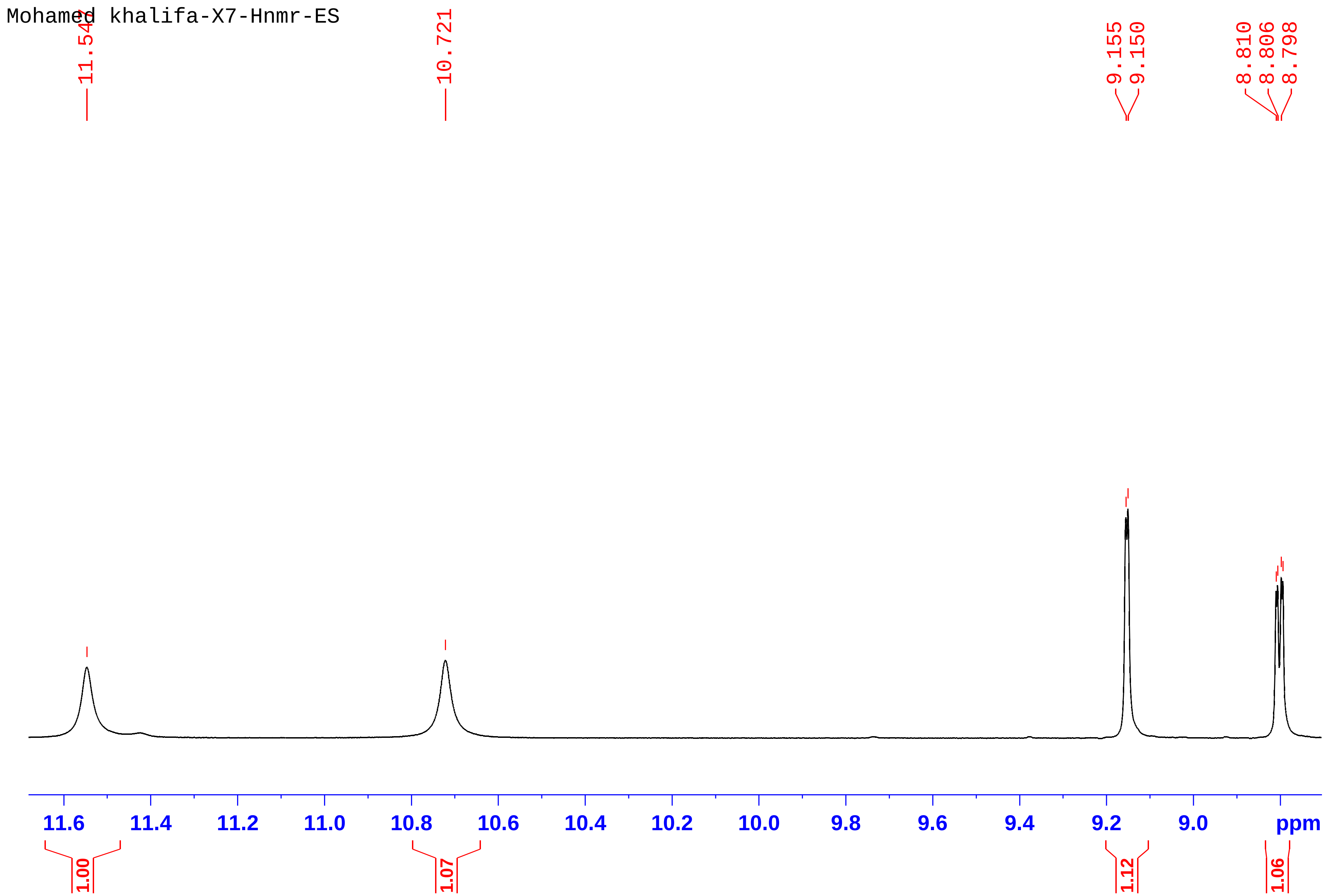
Mohamed khalifa-X7-Hnmr-ES
11.542
10.721
9.155
9.150
8.810
8.806
8.798
11.6
11.4
11.2
11.0
10.8
10.6
10.4
10.2
10.0
9.8
9.6
9.4
9.2
9.0
ppm
1.00
1.07
1.12
1.06

Mohamed khalifa-X8-Hnmr-ES

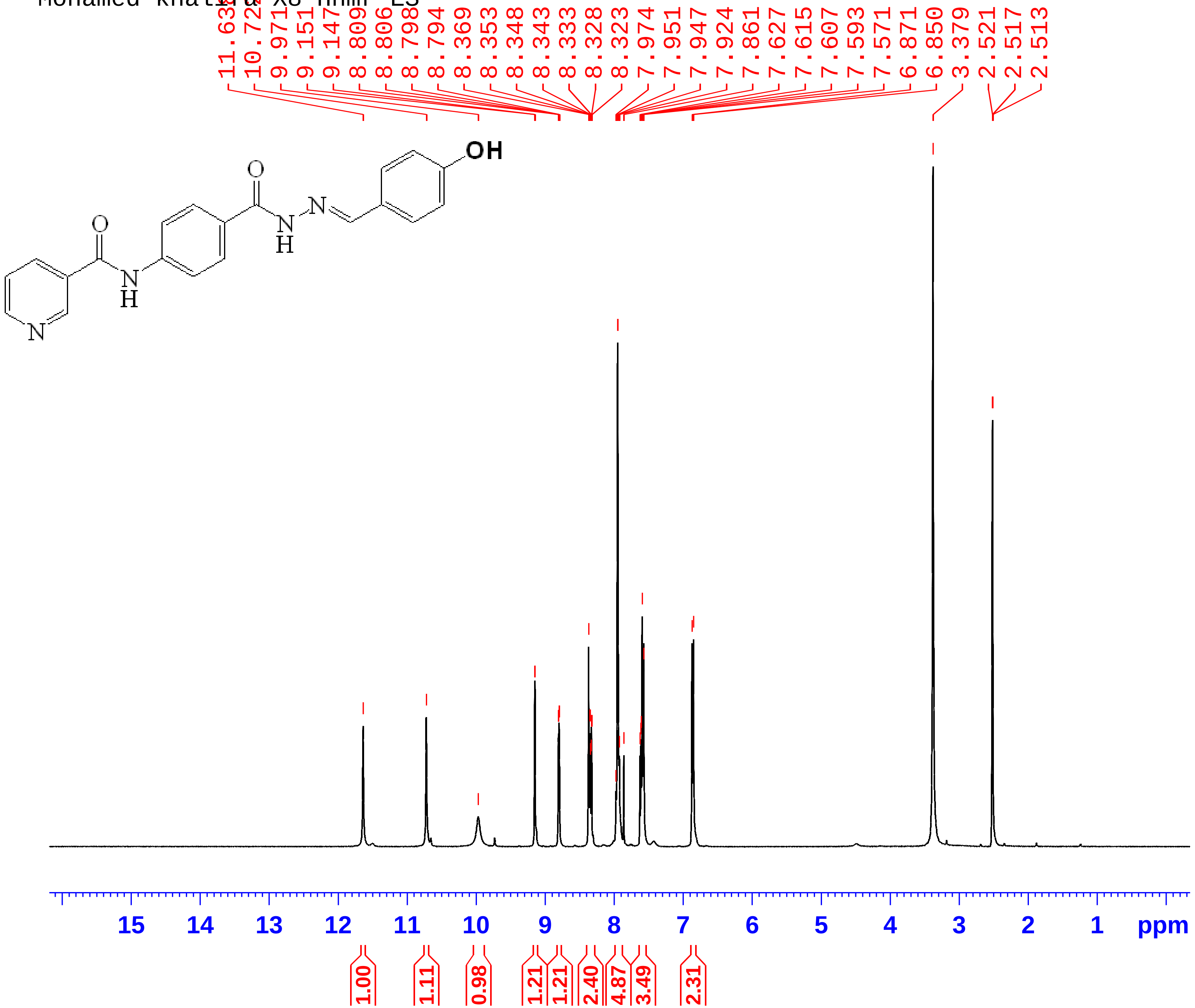

Faculty of Pharmacy

Current Data Parameters

NAME	Mohamed khalifa-X8-Hnmr-ES
EXPNO	10
PROCNO	1

F2 - Acquisition Parameters

Date_	20201126
Time	14.21 h
INSTRUM	spect
PROBHD	Z108618_0945 (
PULPROG	zg30
TD	65536
SOLVENT	DMSO
NS	16
DS	2
SWH	8012.820 Hz
FIDRES	0.244532 Hz
AQ	4.0894465 sec
RG	176.72
DW	62.400 usec
DE	6.50 usec
TE	293.2 K
D1	1.00000000 sec
TD0	1
SFO1	400.2024712 MHz
NUC1	1H
P1	13.50 usec
PLW1	13.00000000 W

F2 - Processing parameters

SI	65536
SF	400.2000000 MHz
WDW	EM
SSB	0
LB	0.30 Hz
GB	0
PC	1.00

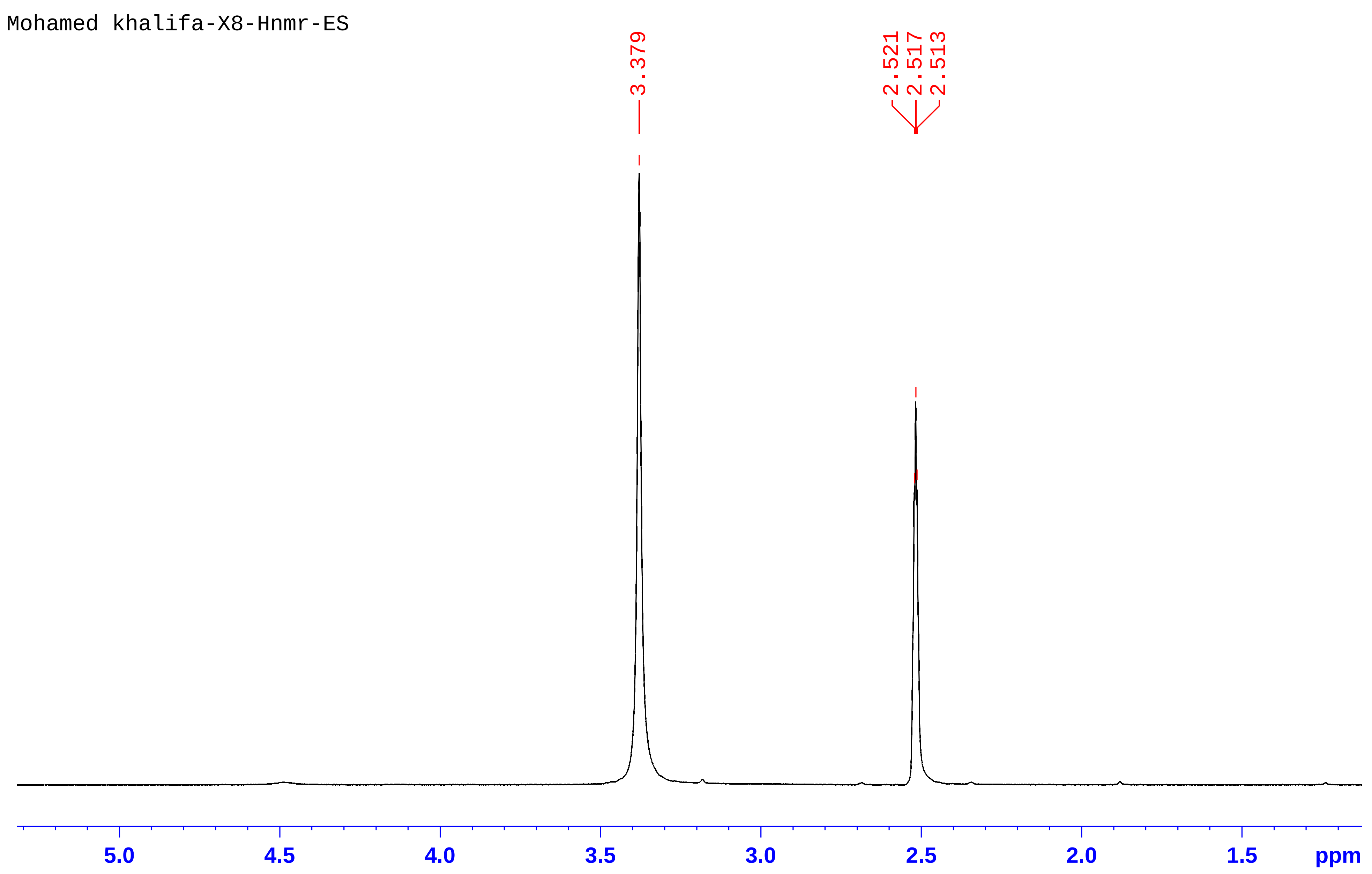
Mohamed khalifa-X8-Hnmr-ES
3.379
2.521
2.517
2.513
5.0
4.5
4.0
3.5
3.0
2.5
2.0
1.5
ppm

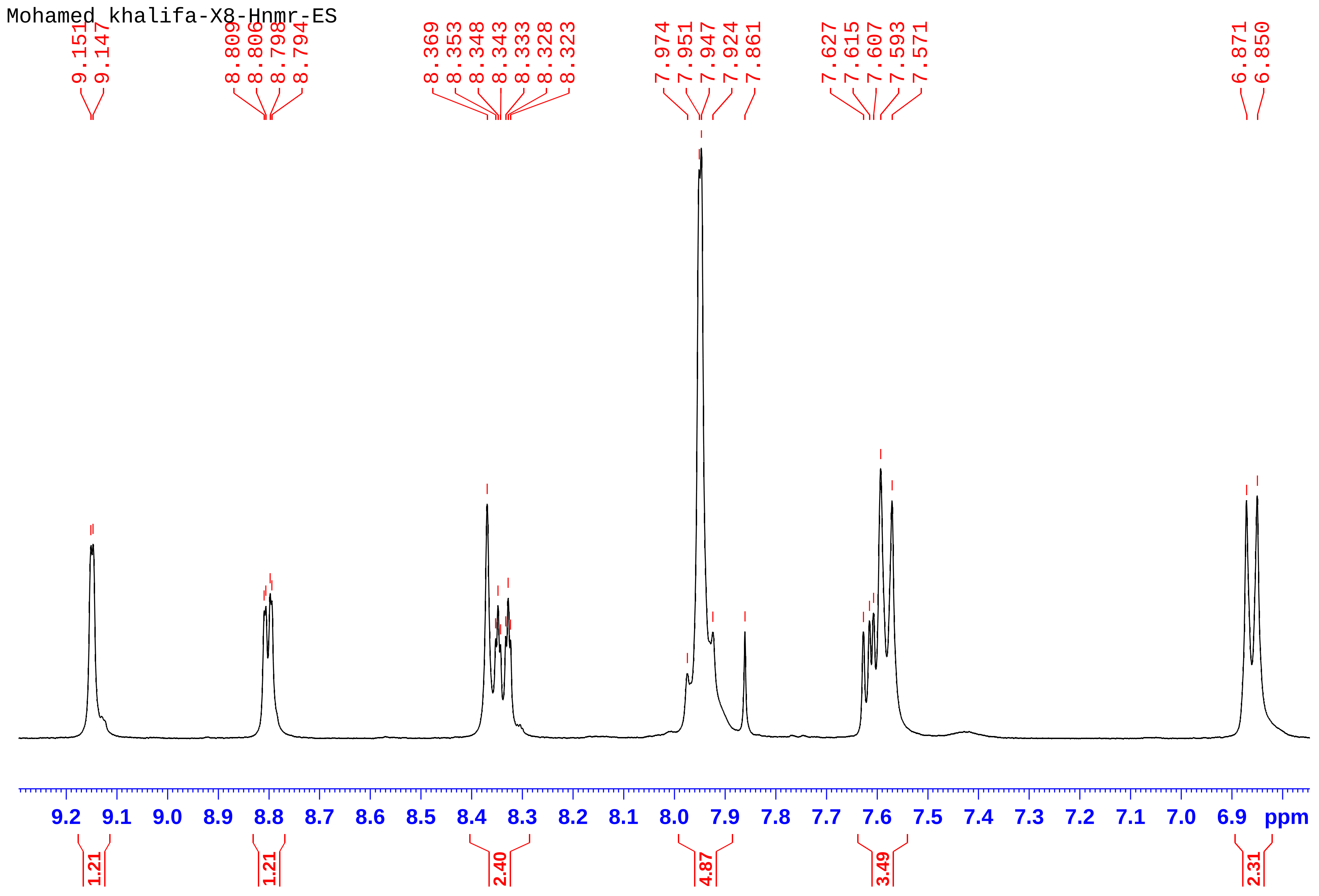
Mohamed khalifa-X8-Hnmr-ES
9.151
9.147
8.809
8.806
8.798
8.794
8.369
8.353
8.348
8.343
8.333
8.328
8.323
7.974
7.951
7.947
7.924
7.861
7.627
7.615
7.607
7.593
7.571
6.871
6.850
9.2
9.1
9.0
8.9
8.8
8.7
8.6
8.5
8.4
8.3
8.2
8.1
8.0
7.9
7.8
7.7
7.6
7.5
7.4
7.3
7.2
7.1
7.0
6.9
ppm
1.21
1.21
2.40
4.87
3.49
2.31

Mohamed khalifa-X8-Hnmr-ES

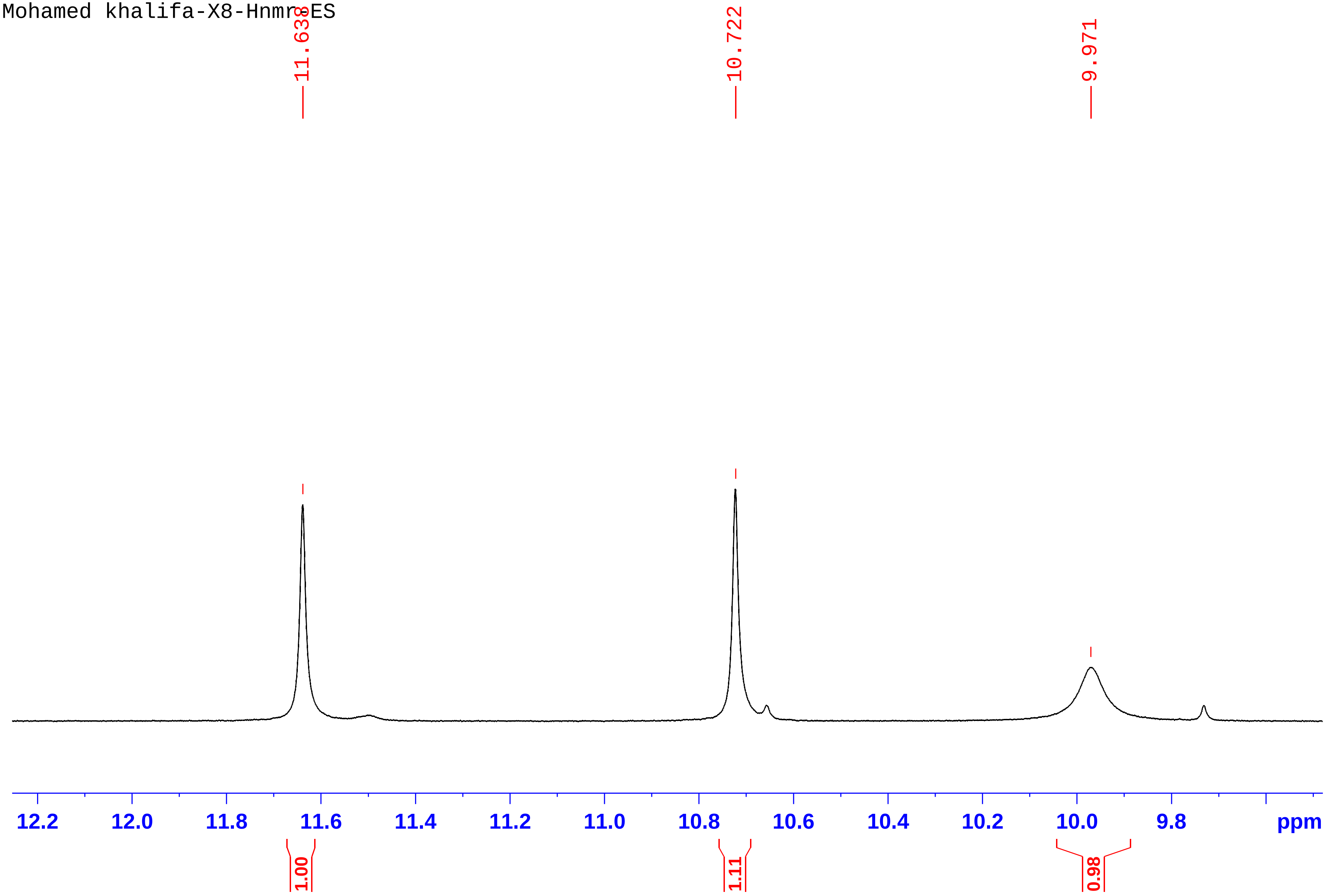

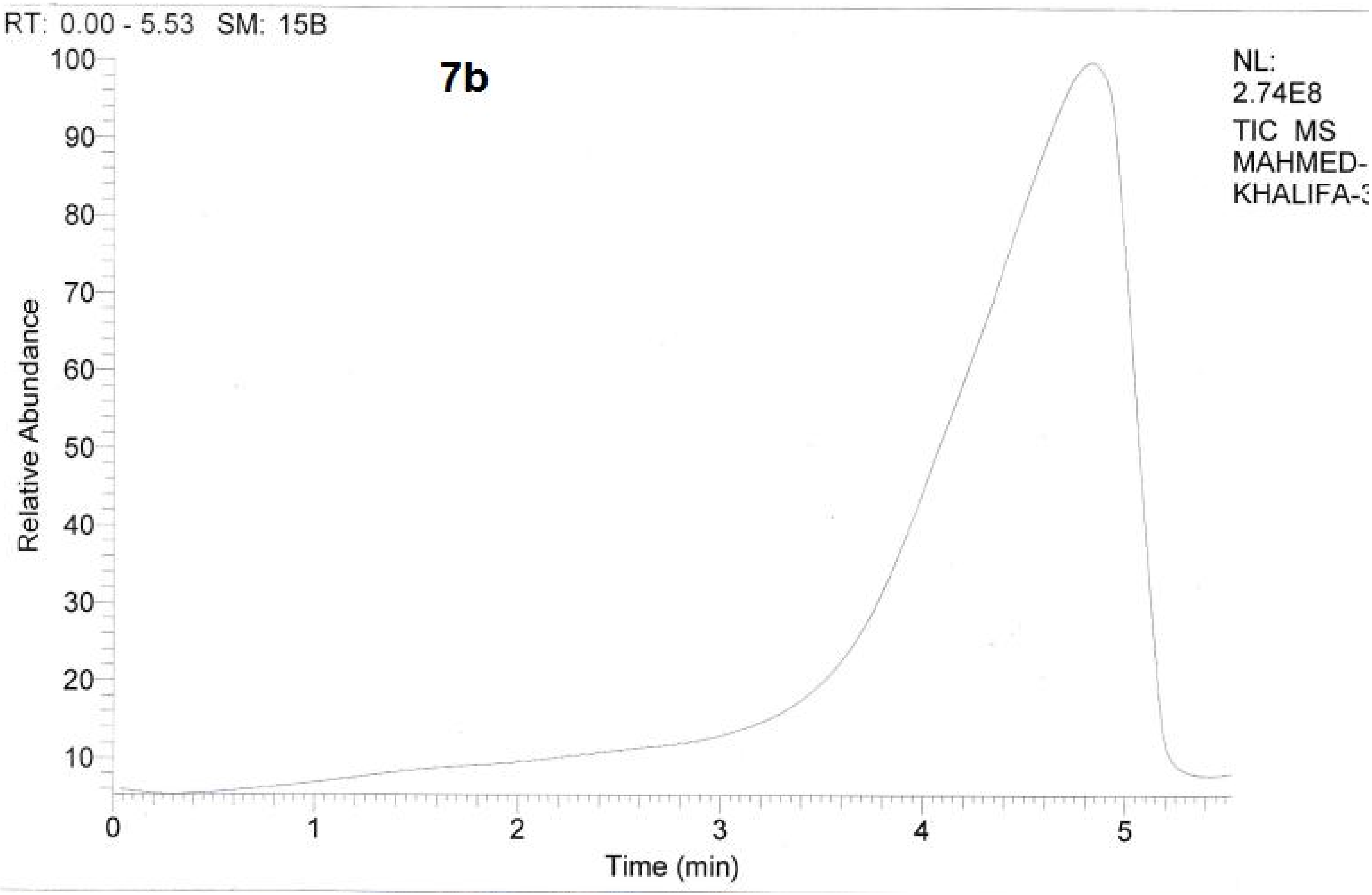
RT: 0.00 - 5.53 SM: 15B
7b
NL:
2.74E8
TIC MS
MAHMED-
KHALIFA-3
Relative Abundance
Time (min)
0
1
2
3
4
5
10
20
30
40
50
60
70
80
90
100

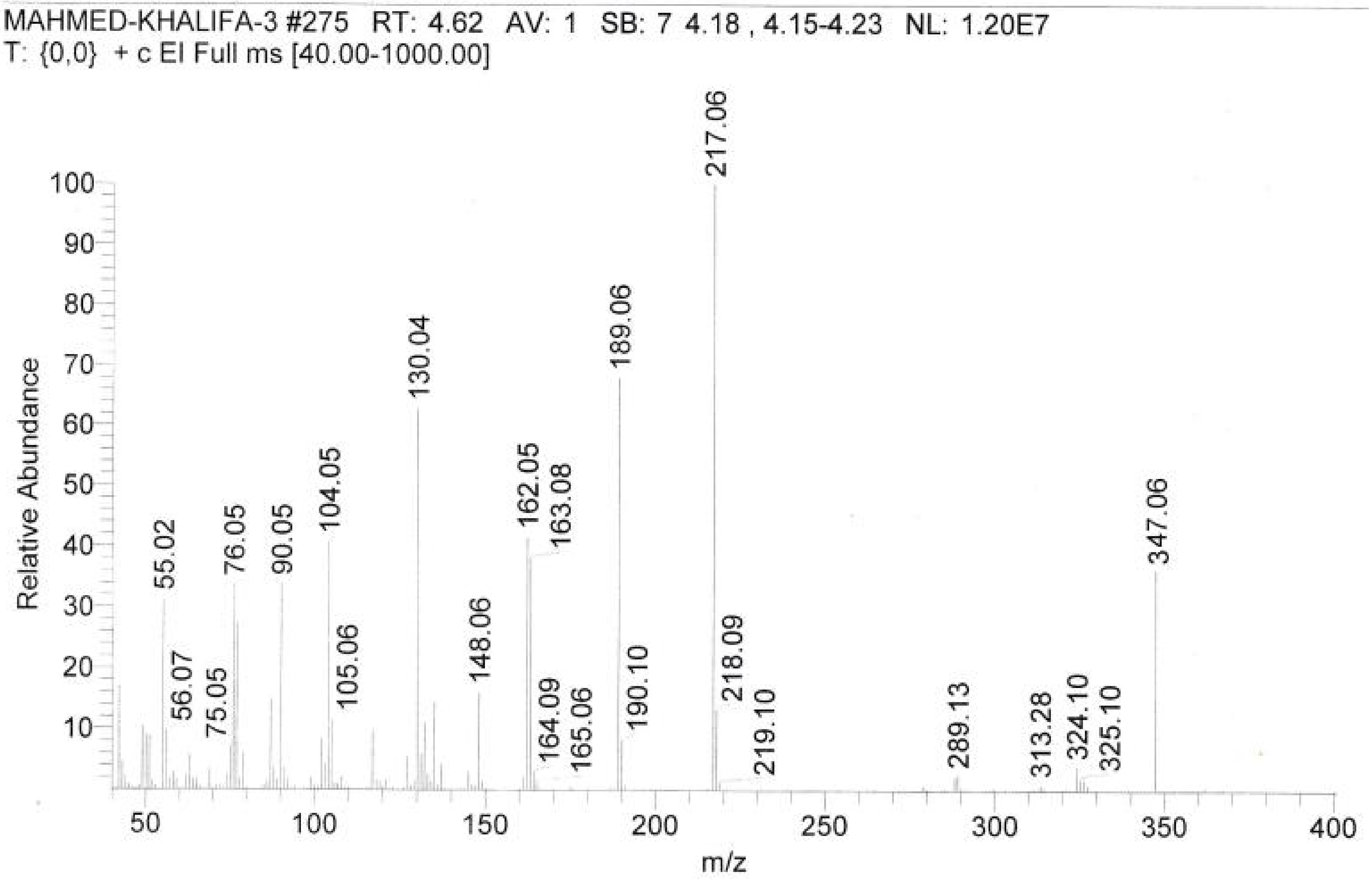

MAHMED-KHALIFA-3 #275 RT: 4.62 AV: 1 SB: 7 4.18 , 4.15-4.23 NL: 1.20E7
T: {0,0} + c EI Full ms [40.00-1000.00]
Relative Abundance
m/z
217.06
189.06
130.04
162.05
163.08
104.05
90.05
76.05
55.02
347.06
56.07
75.05
105.06
148.06
164.09
165.06
190.10
218.09
219.10
289.13
313.28
324.10
325.10

10

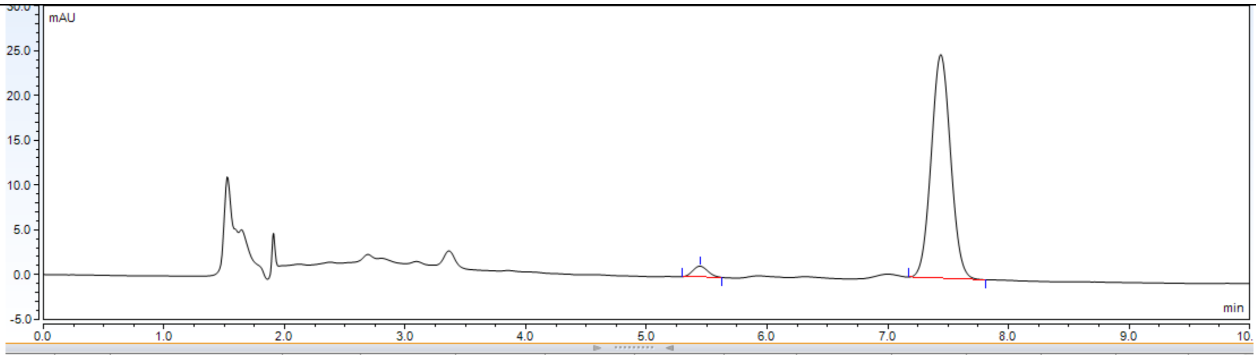

	A	B	C	D	E	F	G	H	I	J	K	L	M	N
1	Peak	Peak Name	Ret.Time	Amount	Rel.Area	Area	Height	Type	Width (50%)	Asym.	Resol.	Plates		
2	No.		min		%	mAU*min	mAU		min	EP	EP	EP		
4	1		5.443	n.a.	3.62	0.1750	1.22	BMB*	0.137	1.11	7.59	8761		
5	2		7.440	5.0674	96.38	4.6607	24.99	BMB*	0.174	1.04	n.a.	10169		
6	Maximum			5.0674	96.38	4.6607	24.99		0.174	1.11	7.59	10169		
7	Minimum			5.0674	3.62	0.1750	1.22		0.137	1.04	7.59	8761		
8	Sum			5.0674	100.00	4.8357	26.21							

18c

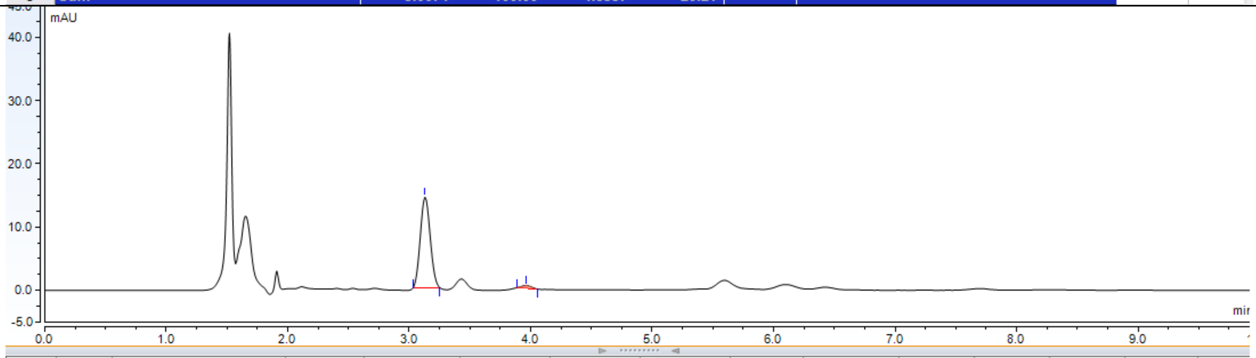

	A	B	C	D	E	F	G	H	I	J	K	L	M	N
1	Peak	Peak Name	Ret.Time	Amount	Rel.Area	Area	Height	Type	Width (50%)	Asym.	Resol.	Plates		
2	No.		min	n.a.	%	mAU*min	mAU		min	EP	EP	EP		
4	1		3.130	n.a.	96.89	1.3539	14.37	BMB*	0.089	1.08	5.36	6790		
5	*2*		*3.963*	*n.a.*	*3.11*	*0.0435*	*0.46*	*BMB**	*0.094*	*1.08*	*n.a.*	*9851*		
6	Maximum			0.0000	96.89	1.3539	14.37		0.094	1.08	5.36	9851		
7	Minimum			0.0000	3.11	0.0435	0.46		0.089	1.08	5.36	6790		
8	Sum			0.0000	100.00	1.3974	14.83							

11b

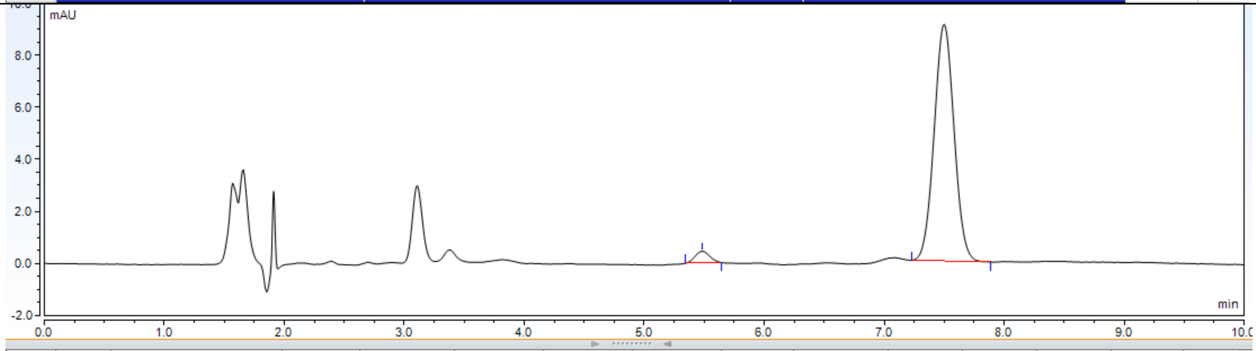

	A	B	C	D	E	F	G	H	I	J	K	L	M	N
1	Peak	Peak Name	Ret.Time	Amount	Rel.Area	Area	Height	Type	Width (50%)	Asym.	Resol.	Plates		
2	No.		min		%	mAU*min	mAU		min	EP	EP	EP		
4	1		5.487	n.a.	3.50	0.0635	0.45	BMB*	0.136	1.07	7.54	9020		
5	2		7.500	1.5622	96.50	1.7489	9.10	BMB*	0.179	1.03	n.a.	9705		
6	Maximum			1.5622	96.50	1.7489	9.10		0.179	1.07	7.54	9705		
7	Minimum			1.5622	3.50	0.0635	0.45		0.136	1.03	7.54	9020		
8	Sum			1.5622	100.00	1.8124	9.55							

11c

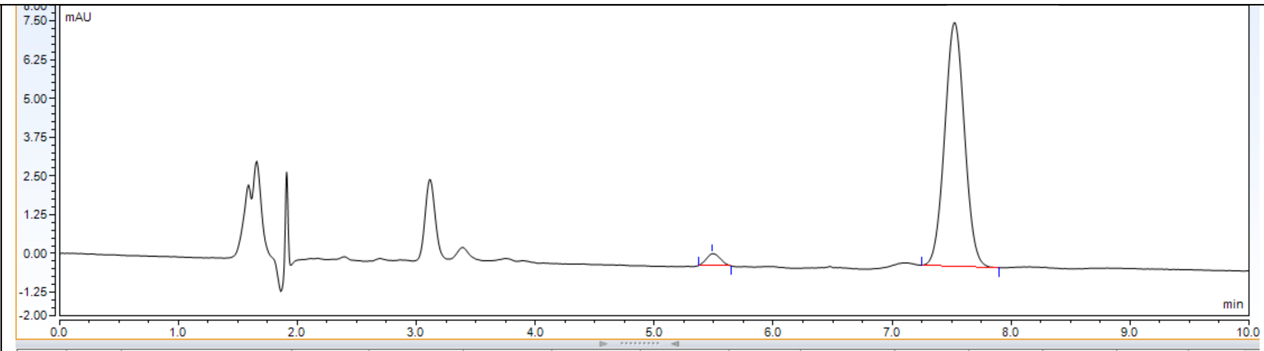

	A	B	C	D	E	F	G	H	I	J	K	L	M	N
1	Peak	Peak Name	Ret.Time	Amount	Rel.Area	Area	Height	Type	Width (50%)	Asym.	Resol.	Plates		
2	No.		min		%	mAU*min	mAU		min	EP	EP	EP		
4	1		5.490	n.a.	3.38	0.0531	0.39	BMB*	0.133	1.13	7.68	9468		
5	2		7.523	1.2827	96.62	1.5167	7.87	BMB*	0.180	1.04	n.a.	9724		
6	Maximum			1.2827	96.62	1.5167	7.87		0.180	1.13	7.68	9724		
7	Minimum			1.2827	3.38	0.0531	0.39		0.133	1.04	7.68	9468		
8	Sum			1.2827	100.00	1.5699	8.26							

11d

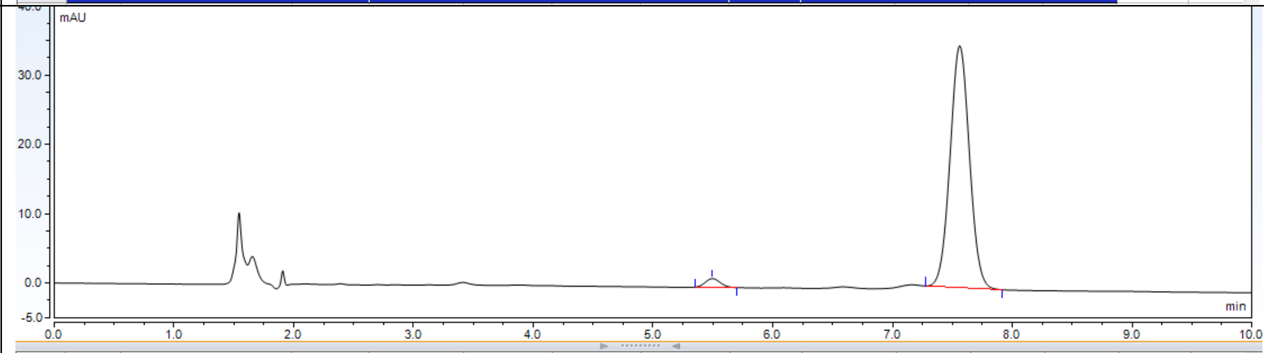

	A	B	C	D	E	F	G	H	I	J	K	L	M	N
1	Peak	Peak Name	Ret.Time	Amount	Rel.Area	Area	Height	Type	Width (50%)	Asym.	Resol.	Plates		
2	No.		min		%	mAU*min	mAU		min	EP	EP	EP		
4	1		5.493	n.a.	2.67	0.1798	1.25	BMB*	0.137	1.15	7.84	8910		
5	2		7.560	7.3421	97.33	6.5503	34.92	BMB*	0.174	1.04	n.a.	10427		
6	Maximum			7.3421	97.33	6.5503	34.92		0.174	1.15	7.84	10427		
7	Minimum			7.3421	2.67	0.1798	1.25		0.137	1.04	7.84	8910		
8	Sum			7.3421	100.00	6.7301	36.17							

11e

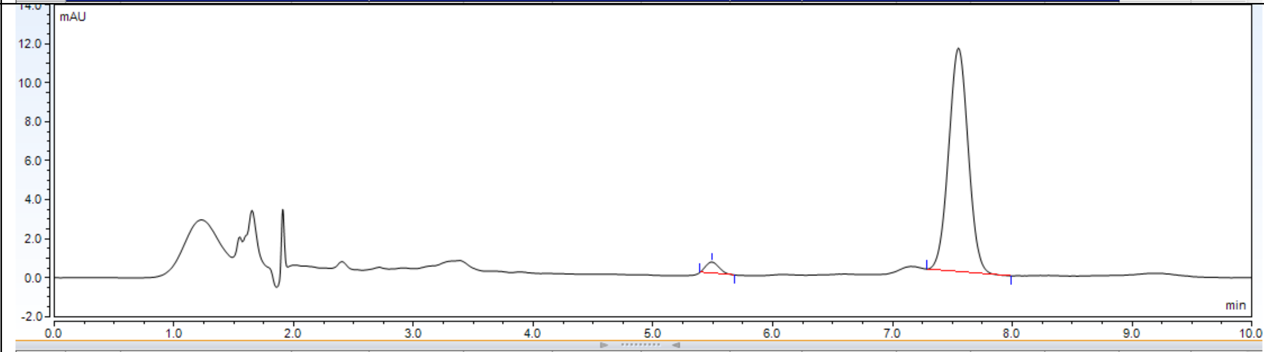

	A	B	C	D	E	F	G	H	I	J	K	L	M	N
1	Peak	Peak Name	Ret.Time	Amount	Rel.Area	Area	Height	Type	Width (50%)	Asym.	Resol.	Plates		
2	No.		min		%	mAU*min	mAU		min	EP	EP	EP		
4	1		5.490	n.a.	3.23	0.0723	0.57	BMB*	0.125	1.24	8.07	10687		
5	2		7.550	2.0684	96.77	2.1694	11.48	BMB*	0.176	1.02	n.a.	10186		
6	Maximum			2.0684	96.77	2.1694	11.48		0.176	1.24	8.07	10687		
7	Minimum			2.0684	3.23	0.0723	0.57		0.125	1.02	8.07	10186		
8	Sum			2.0684	100.00	2.2418	12.05							

11f

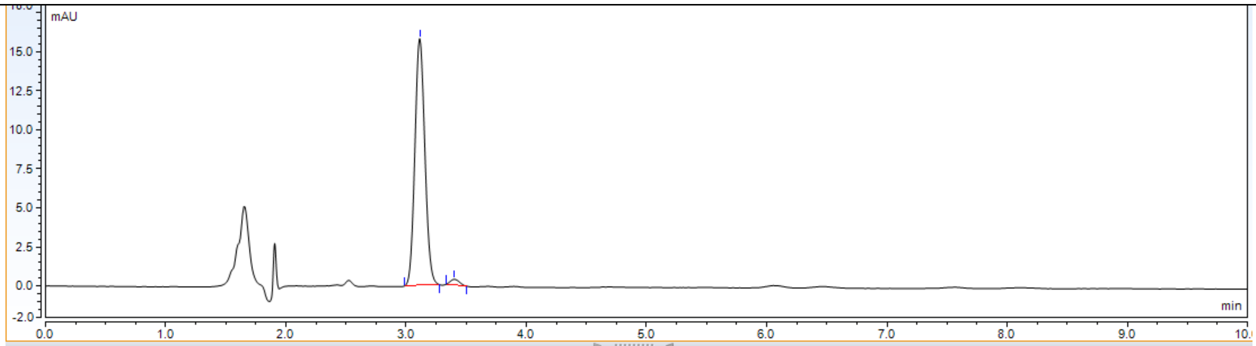

	A	B	C	D	E	F	G	H	I	J	K	L	M	N
1	Peak	Peak Name	Ret.Time	Amount	Rel.Area	Area	Height	Type	Width (50%)	Asym.	Resol.	Plates		
2	No.		min	n.a.	%	mAU*min	mAU		min	EP	EP	EP		
4	1		3.113	n.a.	97.99	1.5110	15.79	BMB*	0.089	1.08	1.94	6763		
5	2		3.400	n.a.	2.01	0.0310	0.36	BMB*	0.085	1.22	n.a.	8868		
6	Maximum			0.0000	97.99	1.5110	15.79		0.089	1.22	1.94	8868		
7	Minimum			0.0000	2.01	0.0310	0.36		0.085	1.08	1.94	6763		
8	Sum			0.0000	100.00	1.5419	16.15							

11g

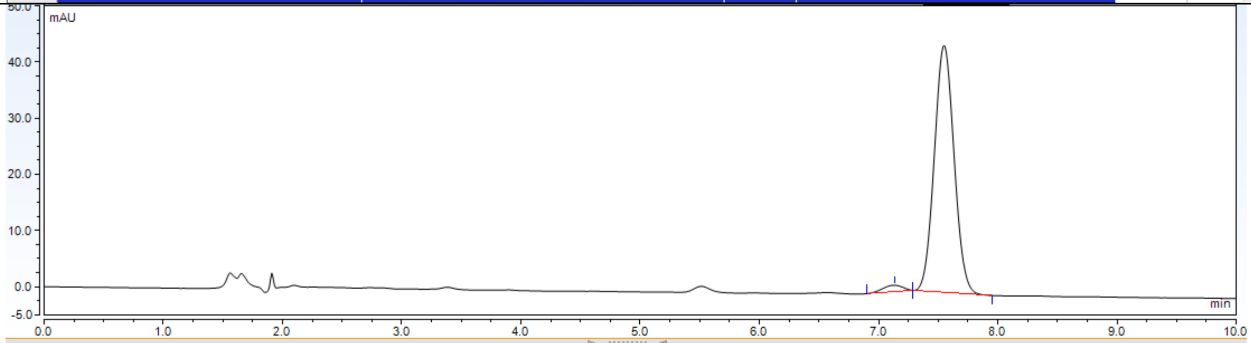

	A	B	C	D	E	F	G	H	I	J	K	L	M	N
1	Peak	Peak Name	Ret.Time	Amount	Rel.Area	Area	Height	Type	Width (50%)	Asym.	Resol.	Plates		
2	No.		min		%	mAU*min	mAU		min	EP	EP	EP		
4	1		7.133	n.a.	2.63	0.2286	1.16	BMb*	0.196	0.85	1.31	7363		
5	2		7.550	9.6382	97.37	8.4577	44.03	bMB*	0.180	1.05	n.a.	9789		
6	Maximum			9.6382	97.37	8.4577	44.03		0.196	1.05	1.31	9789		
7	Minimum			9.6382	2.63	0.2286	1.16		0.180	0.85	1.31	7363		
8	Sum			9.6382	100.00	8.6862	45.18							

11h

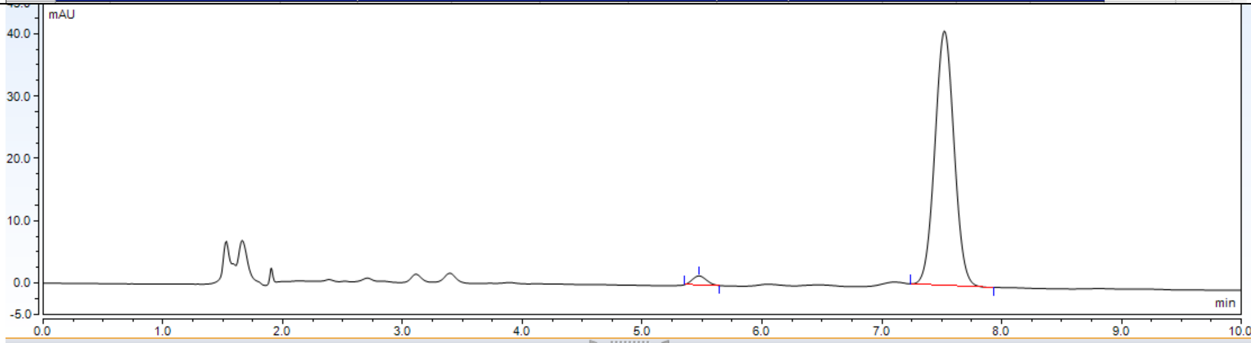

	A	B	C	D	E	F	G	H	I	J	K	L	M	N
1	Peak	Peak Name	Ret.Time	Amount	Rel.Area	Area	Height	Type	Width (50%)	Asym.	Resol.	Plates		
2	No.		min		%	mAU*min	mAU		min	EP	EP	EP		
4	1		5.473	n.a.	2.51	0.1952	1.45	BMB*	0.131	1.16	7.96	9679		
5	2		7.523	8.5932	97.49	7.5896	40.83	BMB*	0.173	1.03	n.a.	10471		
6	Maximum			8.5932	97.49	7.5896	40.83		0.173	1.16	7.96	10471		
7	Minimum			8.5932	2.51	0.1952	1.45		0.131	1.03	7.96	9679		
8	Sum			8.5932	100.00	7.7848	42.28							

7a

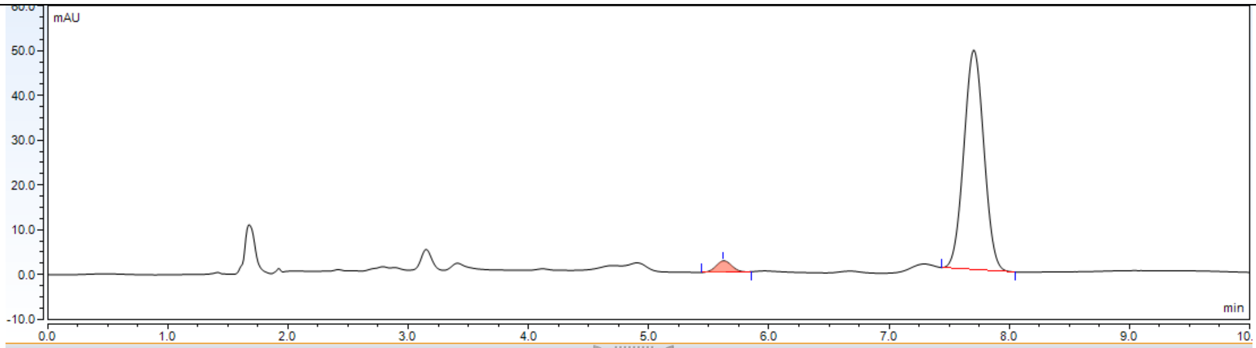

	A	B	C	D	E	F	G	H	I	J	K	L	M	N
1	Peak	Peak Name	Ret.Time	Amount	Rel.Area	Area	Height	Type	Width (50%)	Asym.	Resol.	Plates		
2	No.		min		%	mAU*min	mAU		min	EP	EP	EP		
4	1		5.623	n.a.	3.79	0.3717	2.50	BMB*	0.142	1.03	7.60	8721		
5	2		7.703	10.8303	96.21	9.4479	49.03	BMB*	0.181	1.03	n.a.	10021		
6	Maximum			10.8303	96.21	9.4479	49.03		0.181	1.03	7.60	10021		
7	Minimum			10.8303	3.79	0.3717	2.50		0.142	1.03	7.60	8721		
8	Sum			10.8303	100.00	9.8196	51.53							

7b

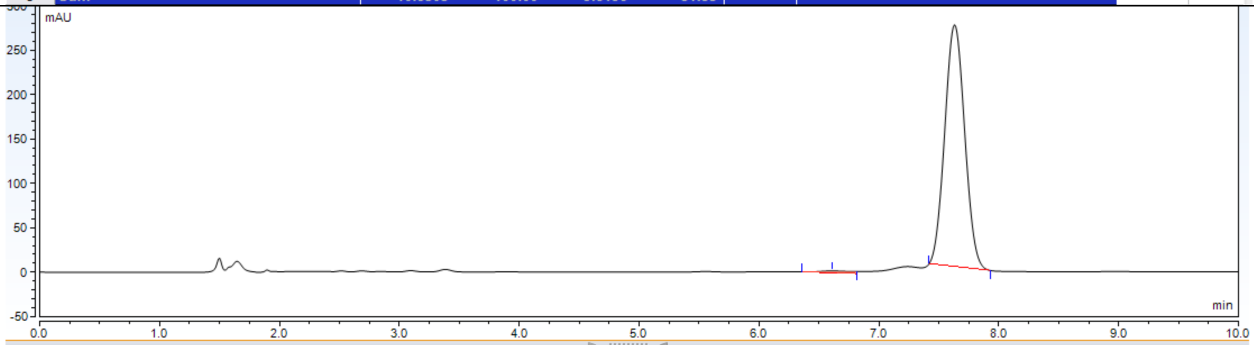

	A	B	C	D	E	F	G	H	I	J	K	L	M	N
1	Peak	Peak Name	Ret.Time	Amount	Rel.Area	Area	Height	Type	Width (50%)	Asym.	Resol.	Plates		
2	No.		min		%	mAU*min	mAU		min	EP	EP	EP		
4	1		6.613	n.a.	1.35	0.7153	2.23	BM *	n.a.	n.a.	n.a.	n.a.		
5	2		7.630	62.5371	98.65	52.4008	272.25	BMB*	0.182	1.06	n.a.	9716		
6	Maximum			62.5371	98.65	52.4008	272.25		0.182	1.06	0.00	9716		
7	Minimum			62.5371	1.35	0.7153	2.23		0.182	1.06	0.00	9716		
8	Sum			62.5371	100.00	53.1161	274.49							

7c

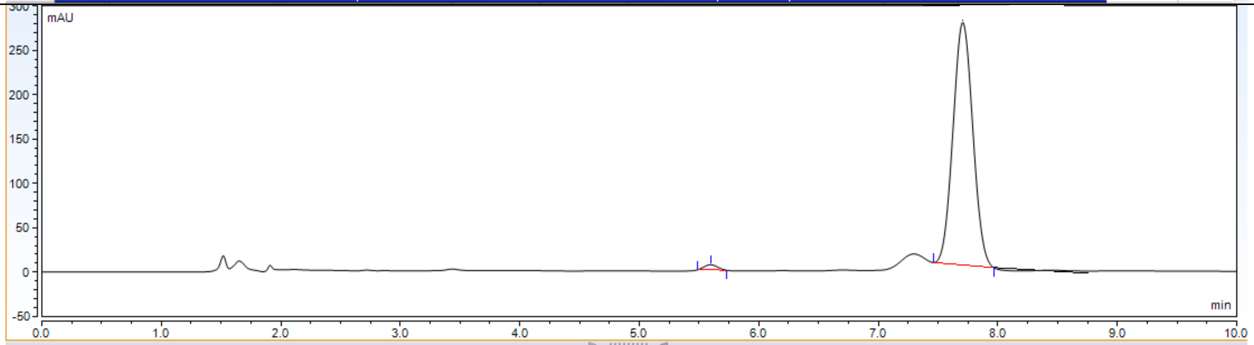

	A	B	C	D	E	F	G	H	I	J	K	L	M	N
1	Peak	Peak Name	Ret.Time	Amount	Rel.Area	Area	Height	Type	Width (50%)	Asym.	Resol.	Plates		
2	No.		min		%	mAU*min	mAU		min	EP	EP	EP		
4	1		5.597	n.a.	1.47	0.7816	6.00	BMB*	0.129	1.06	8.01	10351		
5	2		7.707	62.6906	98.53	52.5283	273.72	BMb*	0.181	1.04	n.a.	10015		
6	Maximum			62.6906	98.53	52.5283	273.72		0.181	1.06	8.01	10351		
7	Minimum			62.6906	1.47	0.7816	6.00		0.129	1.04	8.01	10015		
8	Sum			62.6906	100.00	53.3099	279.72							

7d

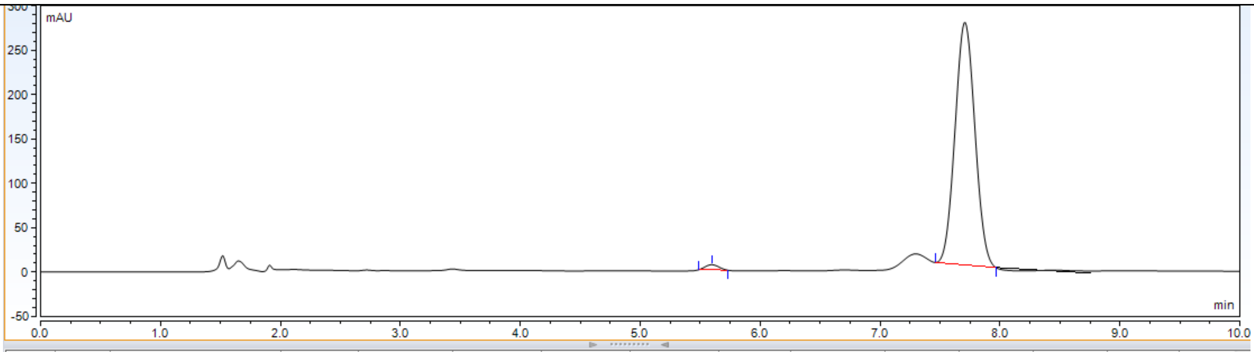

	A	B	C	D	E	F	G	H	I	J	K	L	M	N
1	Peak	Peak Name	Ret.Time	Amount	Rel.Area	Area	Height	Type	Width (50%)	Asym.	Resol.	Plates		
2	No.		min		%	mAU*min	mAU		min	EP	EP	EP		
4	1		5.597	n.a.	1.47	0.7816	6.00	BMB*	0.129	1.06	8.01	10351		
5	2		7.707	62.6906	98.53	52.5283	273.72	BMb*	0.181	1.04	n.a.	10015		
6	Maximum			62.6906	98.53	52.5283	273.72		0.181	1.06	8.01	10351		
7	Minimum			62.6906	1.47	0.7816	6.00		0.129	1.04	8.01	10015		
8	Sum			62.6906	100.00	53.3099	279.72							

6

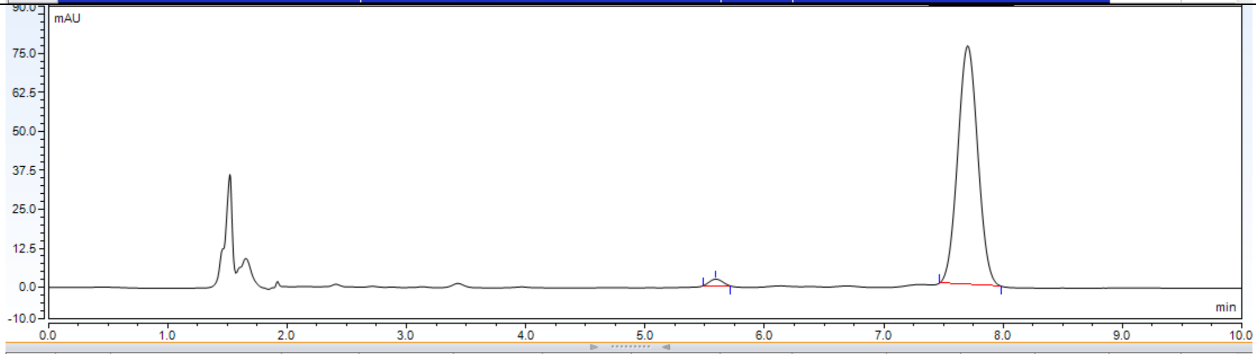

	A	B	C	D	E	F	G	H	I	J	K	L	M	N
1	Peak	Peak Name	Ret.Time	Amount	Rel.Area	Area	Height	Type	Width (50%)	Asym.	Resol.	Plates		
2	No.		min		%	mAU*min	mAU		min	EP	EP	EP		
4	1		5.593	n.a.	1.83	0.2759	2.17	BMB*	0.127	1.05	8.07	10827		
5	2		7.703	17.2657	98.17	14.7938	76.45	BMB*	0.182	1.03	n.a.	9921		
6	Maximum			17.2657	98.17	14.7938	76.45		0.182	1.05	8.07	10827		
7	Minimum			17.2657	1.83	0.2759	2.17		0.127	1.03	8.07	9921		
8	Sum			17.2657	100.00	15.0697	78.62							

7e

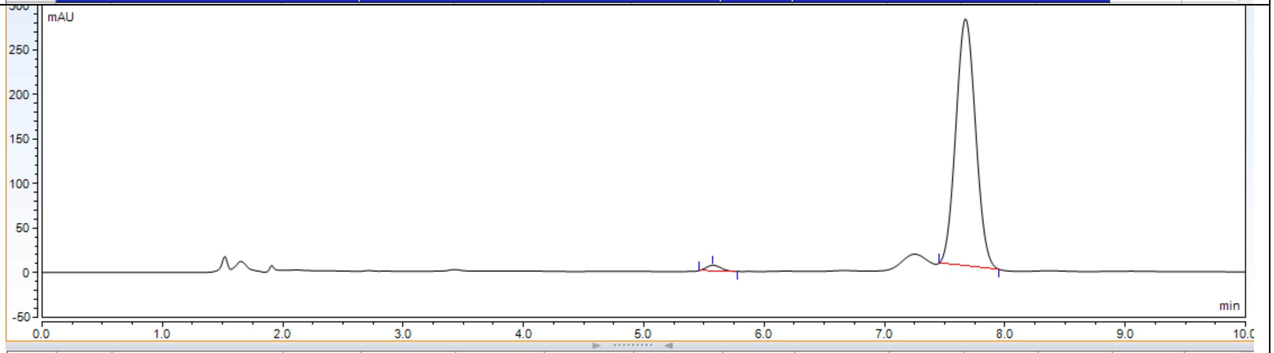

	A	B	C	D	E	F	G	H	I	J	K	L	M	N
1	Peak	Peak Name	Ret.Time	Amount	Rel.Area	Area	Height	Type	Width (50%)	Asym.	Resol.	Plates		
2	No.		min		%	mAU*min	mAU		min	EP	EP	EP		
4	1		5.573	n.a.	1.60	0.8573	6.24	BMB*	0.134	1.21	7.93	9649		
5	2		7.673	62.7559	98.40	52.5826	277.16	BMB*	0.179	1.06	n.a.	10174		
6	Maximum			62.7559	98.40	52.5826	277.16		0.179	1.21	7.93	10174		
7	Minimum			62.7559	1.60	0.8573	6.24		0.134	1.06	7.93	9649		
8	Sum			62.7559	100.00	53.4399	283.40							

7f

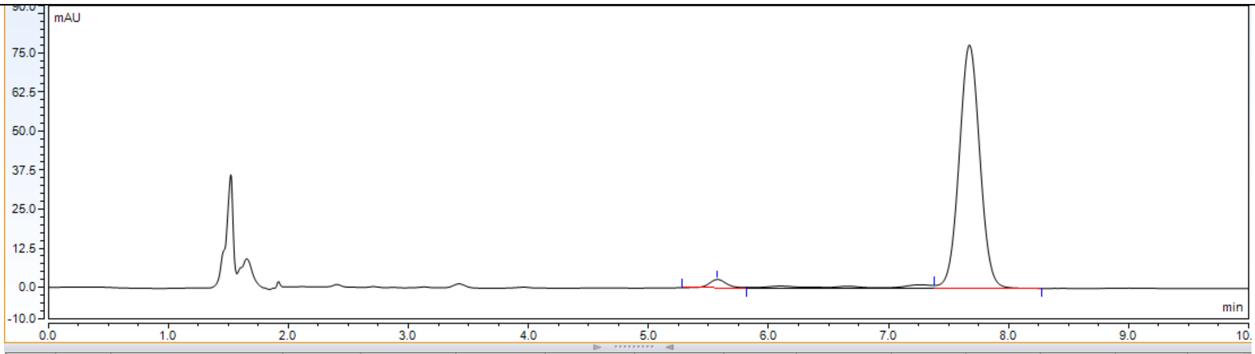

	A	B	C	D	E	F	G	H	I	J	K	L	M	N
1	Peak	Peak Name	Ret.Time	Amount	Rel.Area	Area	Height	Type	Width (50%)	Asym.	Resol.	Plates		
2	No.		min		%	mAU*min	mAU		min	EP	EP	EP		
4	1		5.573	n.a.	2.79	0.4452	2.67	BM	0.148	1.15	7.47	7864		
5	2		7.673	18.1123	97.21	15.4971	77.84	MB	0.184	1.03	n.a.	9666		
6	Maximum			18.1123	97.21	15.4971	77.84		0.184	1.15	7.47	9666		
7	Minimum			18.1123	2.79	0.4452	2.67		0.148	1.03	7.47	7864		
8	Sum			18.1123	100.00	15.9423	80.51							

7g

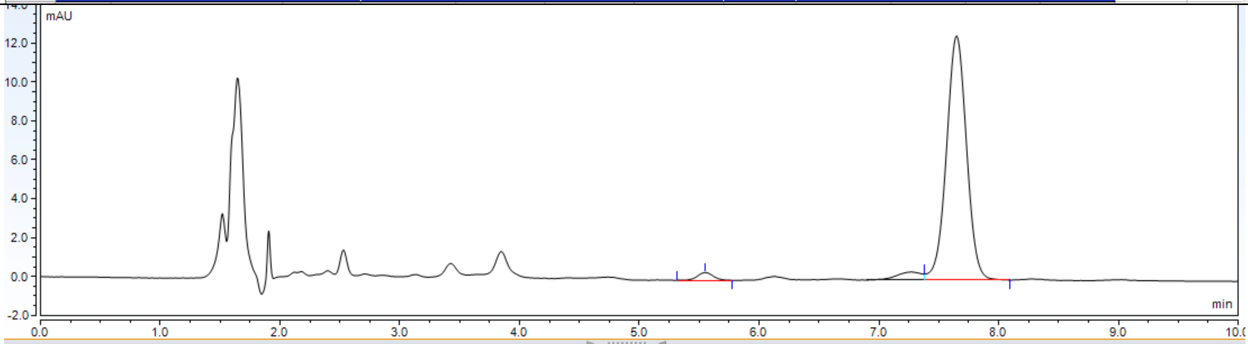

	A	B	C	D	E	F	G	H	I	J	K	L	M	N
1	Peak	Peak Name	Ret.Time	Amount	Rel.Area	Area	Height	Type	Width (50%)	Asym.	Resol.	Plates		
2	No.		min		%	mAU*min	mAU		min	EP	EP	EP		
4	1		5.550	n.a.	2.44	0.0610	0.40	BMB	0.138	1.06	7.84	8978		
5	2		7.650	2.3972	97.56	2.4426	12.57	MB	0.178	0.99	n.a.	10225		
6	Maximum			2.3972	97.56	2.4426	12.57		0.178	1.06	7.84	10225		
7	Minimum			2.3972	2.44	0.0610	0.40		0.138	0.99	7.84	8978		
8	[illegible]			[illegible]	[illegible]	[illegible]	[illegible]							

7h

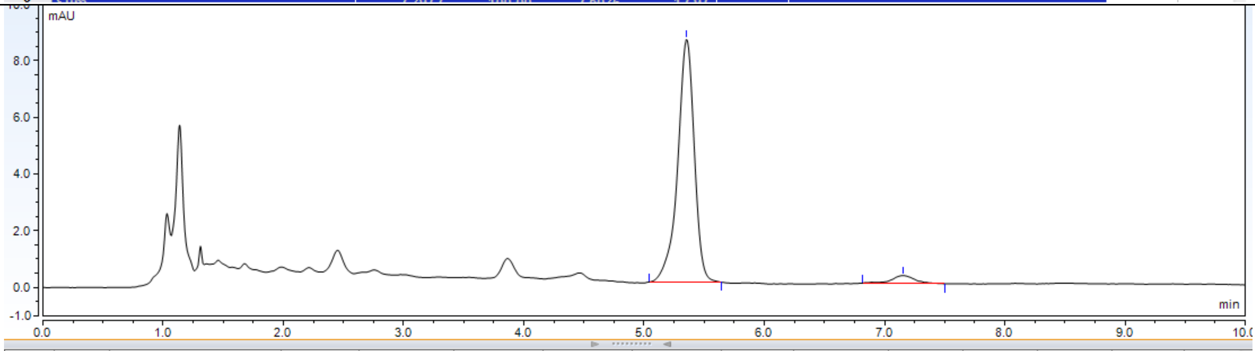

	A	B	C	D	E	F	G	H	I	J	K	L	M	N
1	Peak	Peak Name	Ret.Time	Amount	Rel.Area	Area	Height	Type	Width (50%)	Asym.	Resol.	Plates		
2	No.		min	n.a.	%	mAU*min	mAU		min	EP	EP	EP		
4	1		5.353	n.a.	95.48	1.3732	8.58	BMB*	0.141	0.87	6.45	8025		
5	2		7.153	n.a.	4.52	0.0649	0.28	BMB	0.189	0.94	n.a.	7962		
6	Maximum			0.0000	95.48	1.3732	8.58		0.189	0.94	6.45	8025		
7	Minimum			0.0000	4.52	0.0649	0.28		0.141	0.87	6.45	7962		
8	Sum			0.0000	100.00	1.4381	8.87							

7i

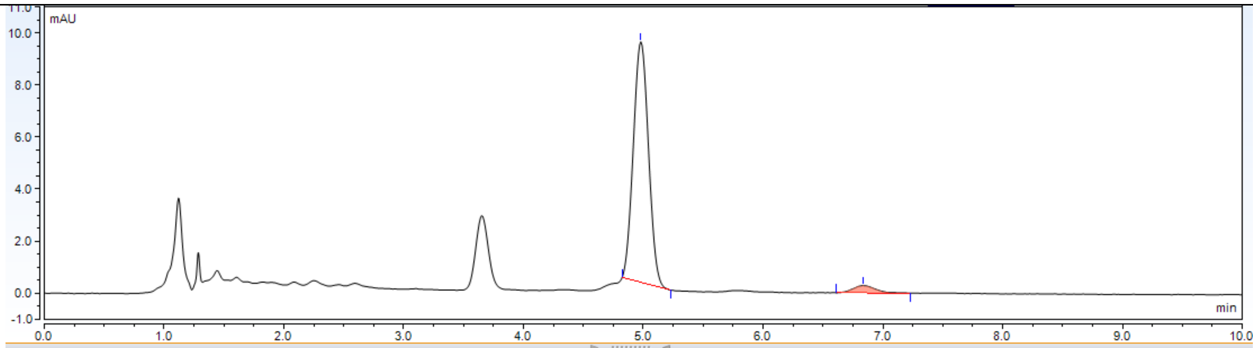

	A	B	C	D	E	F	G	H	I	J	K	L	M	N
1	Peak	Peak Name	Ret.Time	Amount	Rel.Area	Area	Height	Type	Width (50%)	Asym.	Resol.	Plates		
2	No.		min	n.a.	%	mAU*min	mAU		min	EP	EP	EP		
4	1		4.980	n.a.	95.60	1.3559	9.25	BMB*	0.139	1.05	6.43	7079		
5	2		6.833	n.a.	4.40	0.0623	0.28	BMB	0.201	1.27	n.a.	6420		
6	Maximum			0.0000	95.60	1.3559	9.25		0.201	1.27	6.43	7079		
7	Minimum			0.0000	4.40	0.0623	0.28		0.139	1.05	6.43	6420		
8	Sum			0.0000	100.00	1.4183	9.53							

5

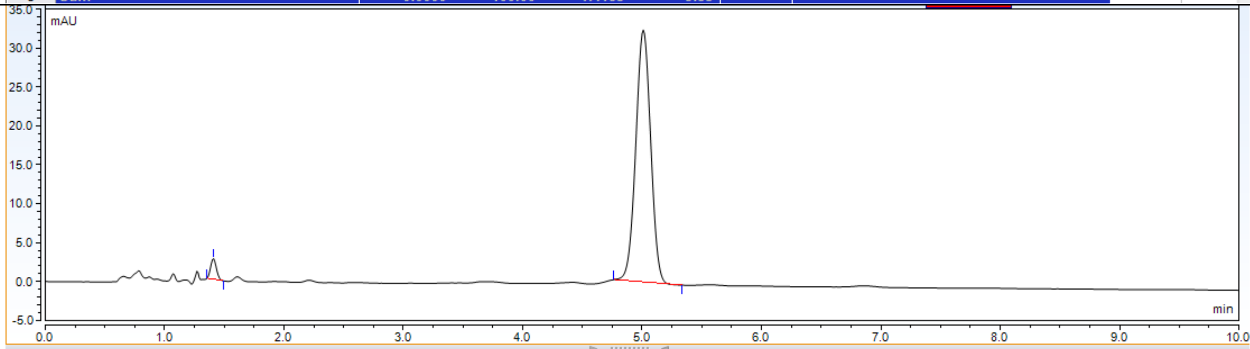

	A	B	C	D	E	F	G	H	I	J	K	L	M	N
1	Peak	Peak Name	Ret.Time	Amount	Rel.Area	Area	Height	Type	Width (50%)	Asym.	Resol.	Plates		
2	No.		min	n.a.	%	mAU*min	mAU		min	EP	EP	EP		
4	1		1.407	n.a.	3.07	0.1482	2.66	BMB*	0.053	1.16	22.60	3868		
5	2		5.010	n.a.	96.93	4.6787	32.41	BMB*	0.135	0.99	n.a.	7641		
6	Maximum			0.0000	96.93	4.6787	32.41		0.135	1.16	22.60	7641		
7	Minimum			0.0000	3.07	0.1482	2.66		0.053	0.99	22.60	3868		
8	Sum			0.0000	100.00	4.8269	35.07							

7j

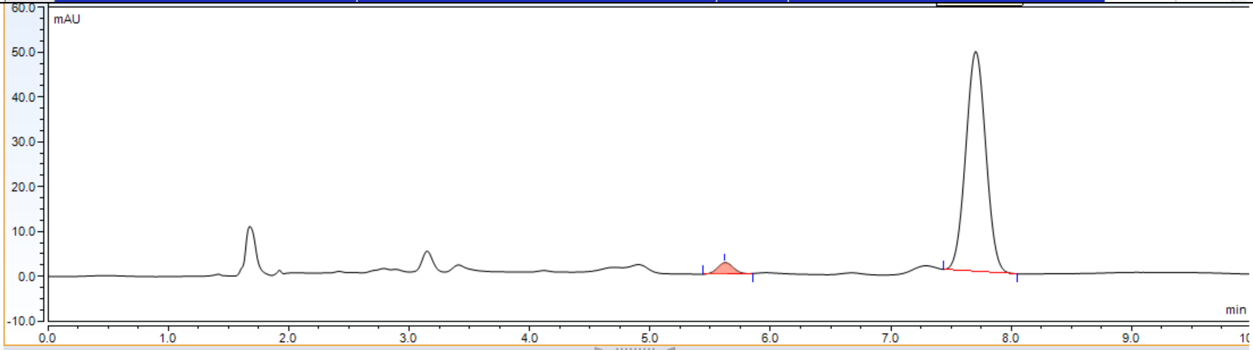

	A	B	C	D	E	F	G	H	I	J	K	L	M	N
1	Peak	Peak Name	Ret.Time	Amount	Rel.Area	Area	Height	Type	Width (50%)	Asym.	Resol.	Plates		
2	No.		min		%	mAU*min	mAU		min	EP	EP	EP		
4	1		5.623	n.a.	3.79	0.3717	2.50	BMB*	0.142	1.03	7.60	8721		
5	2		7.703	10.8303	96.21	9.4479	49.03	BMB*	0.181	1.03	n.a.	10021		
6	Maximum			10.8303	96.21	9.4479	49.03		0.181	1.03	7.60	10021		
7	Minimum			10.8303	3.79	0.3717	2.50		0.142	1.03	7.60	8721		
8	Sum			10.8303	100.00	9.8196	51.53							

7l

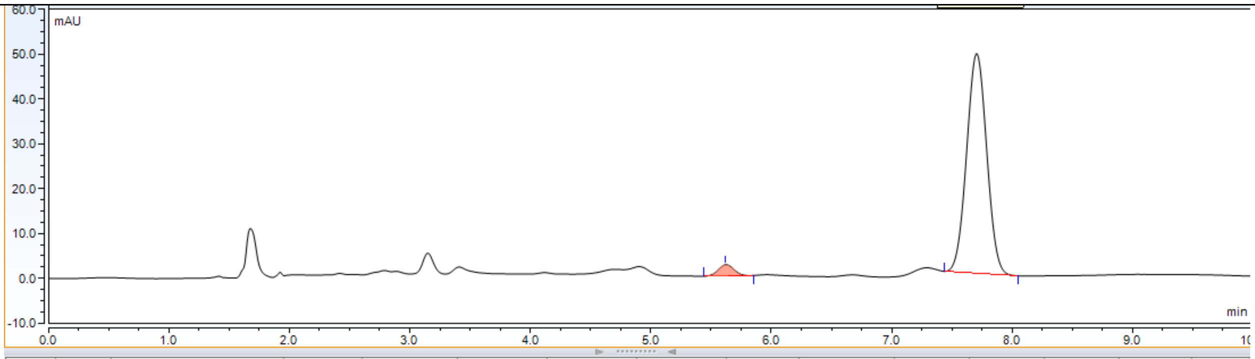

	A	B	C	D	E	F	G	H	I	J	K	L	M	N
1	Peak	Peak Name	Ret.Time	Amount	Rel.Area	Area	Height	Type	Width (50%)	Asym.	Resol.	Plates		
2	No.		min		%	mAU*min	mAU		min	EP	EP	EP		
4	1		5.623	n.a.	3.79	0.3717	2.50	BMB*	0.142	1.03	7.60	8721		
5	2		7.703	10.8303	96.21	9.4479	49.03	BMB*	0.181	1.03	n.a.	10021		
6	Maximum			10.8303	96.21	9.4479	49.03		0.181	1.03	7.60	10021		
7	Minimum			10.8303	3.79	0.3717	2.50		0.142	1.03	7.60	8721		
8	Sum			10.8303	100.00	9.8196	51.53							

4.2. Biological evaluation

4.2.1. *In vitro anti-proliferative activity against MCF-7, HepG-2 and HCT-116*

The cells were cultured in RPMI-1640 medium supplemented with 10% fetal bovine serum (FBS), penicillin (100 unit /mL) and streptomycin sulphate (100µg/mL) at 37 oC in a 5% CO_2 incubator. Then, cells were dropped in 96-well plates at a density of 3-8 × 103 cells/well and incubated for two days in a 5% CO2 incubator at 37 °C. Then, the cells were treated with the synthesized compounds and the cell cultures were continued incubated for 24 h. However, Different concentrations of the compound under test (0.0, 5.0, 12.5, 25.0 and 50 mg/ml) were added to the cell monolayer. Then, (3-[4,5-dimethylthiazol-2-yl]- 2,5-diphenyltetrazolium bromide) MTT solution (20 µl, 5mg/mL) was added to each well and incubated for additional 4 h. The formed purple crystals of MTT-formazan were dissolved in 100 µl DMSO each well; the absorbance of each well was measured at 570 nm using a plate reader (EXL 800, USA). All of the compounds were tested three times. The relative cell viability in percentage was calculated. The results for IC50 values of the active compounds are summarized in **Table 1, 2 and 3**. The data represented the mean of three independent experiments in triplicate and were expressed as means ± SD. The IC_{50} value was defined as the concentration at which 50% of the cells could survive [1, 2].

4.2.2. *In vitro VEGFR-2 enzyme assay inhibition*

The in vitro inhibitory activities of the tested compounds against Human VEGFR-2 were evaluated using ELISA kit (Enzyme-Linked Immunosorbent Assay) with quantitation performed through the Alpha Screen® System (PerkinElmer, USA) according to manufacturer's instructions. The assay employs an anti-phosphotyrosine antibody (specific for VEGFR-2) seeded on a 96-well plate. 100 µL of the standard solution or the tested compound was added into the wells, and incubated over night at 4°C with gentle shaking. After the wells washed, 100µL of the prepared biotin antibody was added, and incubated at room temperature for additional 1h. After washing to move away unbound biotinylated antibody, 100µL of streptavidin solution was added

to the wells then incubated for 45min. at room 48 temperature, followed by washing again. 100µL of TMB Substrate reagent was added and the color produced is proportional to the amount of the of VEGFR-2 bound. The stop solution was added, then the intensity of color is read at 450 nm by ELISA Reader (PerkinElmer) immediately. Percent inhibition was calculated by the comparison of compounds treated to control incubations, the concentration of the test compound causing 50% inhibition (IC50) was calculated from the concentration inhibition response curve (concentrations on the X-axis and the absorbance on the Yaxis) and the data were compared with sorafenib as a standard VEGFR-2 inhibitor [3].

4.2.3. ***Apoptotic markers analysis***

4.2.3.1. Effects on the levels of active caspase-3

HepG-2 cancer cells were treated with the most active compounds. The cells were trypsinized, rinsed with PBS and centrifuged. Subsequently, the cells were harvested, suspended in 1 mL PBS, frozen at < -20 °C and thawed with gentle mixing. This cycle of freeze/thraw was repeated for three times and centrifuged for 10 min to purify from cell debris. Caspase-3 human kit is a solid phase sandwich Enzyme Linked Immuno-Sorbent Assay (ELISA). The wells of the microtiter strips provided were coated with a monoclonal specific antibody for human target caspase. Samples and unknowns were pipetted into these wells and then a rabbit antibody specific for human target caspase were added to the wells. Through the first incubation, the human target caspase-3 bind to the immobilized (capture) antibody and the specific active caspase-3 antibody served as a detection of antibody by binding to the immobilized active caspase-3 proteins. After the first incubation and washing to remove excess protein, a horseradish peroxidase-labeled anti-Rabbit IgG (anti-rabbit IgG HRP) was added. After a third incubation and washing to remove all excess anti-rabbit IgG HRP, a substrate solution was added to produce a colored product as a result of interaction with the bound enzyme. The intensity of the color is directly proportional to the concentration of human active caspase-3 in the original specimen [4].

4.2.3.2. Effects on bcl-2 family proteins

Total RNA was extracted from at least 1 × 106 cells using RNeasy Mini-spin column kit RNeasy RNA extraction kit (Qiagen). A reaction mixture (50 µL) was prepared according to the following recipe: 2X SYBR® Green RT-PCR Reaction Mix (25 µL); Forward primer (10 µM) (1.5 µL); Reverse primer (10 µM) (1.5 µL); Nuclease-free H2O; RNA template (1 pg to 100 ng total RNA); iScript Reverse Transcriptase for One-Step RT-PCR (1 µL). Amplification was performed using a real-time thermal detection system (Rotorgene) as follows: cDNA synthesis: 10 min at 50 °C; iScript Reverse transcriptase inactivation: 95 °C (5 min); PCR cycling and detection 45 cycles: 95 °C (10 sec); 55 °C (30 sec) then data collection step and Melt curve analysis. The gene expression fold values were calculated using 2(-delta delta CT) method for data analysis of quantitative real time polymerase chain reaction [5].

4.2.3.3. Cell cycle analysis

HepG-2 cells were grown in six-well plates (each one contains 2 x 105 cells per well) containing 10% foetal bovine serum and incubated for 24 h at 37ºC and 5% CO2. The medium was replaced with (DMSO 1% v/v) containing the 3.1 µM of compound **7$_a$**, then incubated for 48 h, collected and washed with cold phosphate buffered saline (PBS). After fixation of the collected cells with ice-cold absolute ethanol (70%), the cells were rinsed with PBS then stained with the DNA fluorochrome PI, kept for 15 min at 37ºC. Then samples were analyzed with a FACS Caliber flow cytometer [6].

4.2.3.4. Annexin V-FITC apoptosis assay

Apoptotic activity of compound **7$_a$** was evaluated using Annexin V-FITC/PI apoptosis detection kit. HepG-2 cells (2 x 105) were seeded and incubated with compound **7$_a$** for 48 h, trypsinized, collected and washed with phosphate-buffered saline (PBS) several times; HepG-2 cells were stained with Annexin V fluorescein isothiocyanate (FITC) and counterstained with propidium iodide (PI) for 15 min at 37ºC in the dark using the apoptosis detection kit (BD Biosciences, San Jose, CA)

according to the manufacturer's protocol. Then, Annexin V-FITC and PI binding were analyzed by a FACS Caliber flow cytometer [7].

4.2.5. Vascular smooth muscle cell culture

VSMCs were isolated from thoracic aortas of 2- 3-monthold rats as described previously. VSMCs (2 × 104 cells) were seeded in 24-well plates, and inoculated into RPMI 1640 containing 10% FBS with for 24h then VSMCs were treated with various concentrations of drug, and incubated for 48. Cells in RPMI 1640 plus 10% FBS alone served as a control. After treatment, cells were obtained by trypsinization, and cell numbers were counted and cell proliferation was observed. from the results we can indicate that treatment with compound caused inhibitory effects on cell proliferation [8].

4.3. Molecular docking

VEGFR-2 crystal structure of was downloaded from the Protein Data Bank, http://www.rcsb.org/pdb (PDB ID: 4ASD, resolution: 2.05 Å) using Discovery Studio 2.5 software. Water molecules were removed from the downloaded protein. Alternate conformations and valence monitor options were used to correct crystallographic disorders and unfilled valence atoms. The energy of the 4ASD protein was minimized using CHARMm and MMFF94 force fields for charge and partial charge, respectively. The active binding site of the protein was identified and prepared for docking. Structures of the designed compounds and reference ligand, sorafenib, were sketched using ChemBioDraw Ultra 14.0. Structures were saved in MDL-SD file format. Next, the SD file was opened followed by protonation of the 3D structures Then, and energy was minimized by applying CHARMm and MMFF94 force fields. Energy minimization was carried out as described in the Supporting Information. The structures were then prepared for docking by optimization of the parameters. Docking was carried out using CDOCKER-CHARMm-based technique in the interface of Accelry's Discovery Studio 2.5. A maximum of 10 conformers was measured for each molecule in the docking analysis. Subsequently, the docking scores (CDOCKER

interaction energy) of the most ideal pose of each of the docked molecules with the amino acids at the VEGFR-2 binding pocket were recorded [9].

1. Mosmann, T., *Rapid colorimetric assay for cellular growth and survival: application to proliferation and cytotoxicity assays.* Journal of Immunological Methods, 1983. **65**(1-2): p. 55-63.
2. Denizot, F. and R. Lang, *Rapid colorimetric assay for cell growth and survival: modifications to the tetrazolium dye procedure giving improved sensitivity and reliability.* Journal of Immunological Methods, 1986. **89**(2): p. 271-277.
3. Eldehna, W.M., et al., *Increasing the binding affinity of VEGFR-2 inhibitors by extending their hydrophobic interaction with the active site: Design, synthesis and biological evaluation of 1-substituted-4-(4-methoxybenzyl) phthalazine derivatives.* European journal of medicinal chemistry, 2016. **113**: p. 50-62.
4. Andersson, M., et al., *Caspase and proteasome activity during staurosporin-induced apoptosis in lens epithelial cells.* Investigative Ophthalmology and Visual Science, 2000. **41**(9): p. 2623-2632.
5. LIVAK, K. and T. Schmittgen, *Analysis of relative gene expression data using real-time quanti tative PCR and the 2 (-MC (T)) Method.* Methods, 2001. **25**: p. 402-408.
6. Wang, J. and M.J. Lenardo, *Roles of caspases in apoptosis, development, and cytokine maturation revealed by homozygous gene deficiencies.* Journal of Cell Science, 2000. **113**(5): p. 753-757.
7. Lo, K.K.-W., et al., *Luminescent biological probes derived from ruthenium (II) estradiol polypyridine complexes.* Inorganic Chemistry, 2008. **47**(1): p. 200-208.
8. Seo, H.-H., et al., *7-cyclopentyl-5-(4-phenoxyphenyl)– 7H-pyrrolo [2, 3-d] pyrimidin-4-ylamine inhibits the proliferation and migration of vascular smooth muscle cells by suppressing ERK and Akt pathways.* European Journal of Pharmacology, 2017. **798**: p. 35-42.
9. Murcko, M.A., *Computational methods to predict binding free energy in ligand-receptor complexes.* Journal of Medicinal Chemistry, 1995. **38**(26): p. 4953-4967.
